# Liquid-Phase Peptide Synthesis of Tropolone–Peptide Hybrid Antimalarials

**DOI:** 10.1021/acs.orglett.6c00703

**Published:** 2026-03-26

**Authors:** Goh Sennari, Asuka Nakajima, Hiroki Nakahara, Ryo Saito, Aki Ishiyama, Rei Hokari, Masato Iwatsuki, Tomoyasu Hirose, Toshiaki Sunazuka

**Affiliations:** O̅mura Satoshi Memorial Institute and Graduate School of Infection Control Sciences, 12877 Kitasato University, 5-9-1 Shirokane, Minato-ku, Tokyo 108-8641, Japan

## Abstract

Tropolones are non-benzenoid aromatics with broad bioactivity, yet their inherent planarity and metal-chelating properties pose challenges to a medicinal chemistry campaign. We developed a liquid-phase peptide synthesis strategy that leveraged a hydrophobic TAG carrier to streamline condensation, deprotection, and purification steps, overcoming challenges that hinder traditional solution-phase derivatization of tropolones. Among the synthesized tropolone–peptide hybrid derivatives, the lead analogue exceeded antimalarial potency of puberulic acid and artemisinin, highlighting the advantage of three-dimensional peptide hybridization.

## Graphical abstract

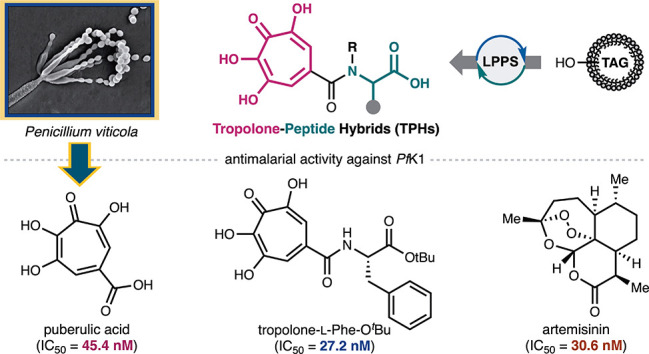

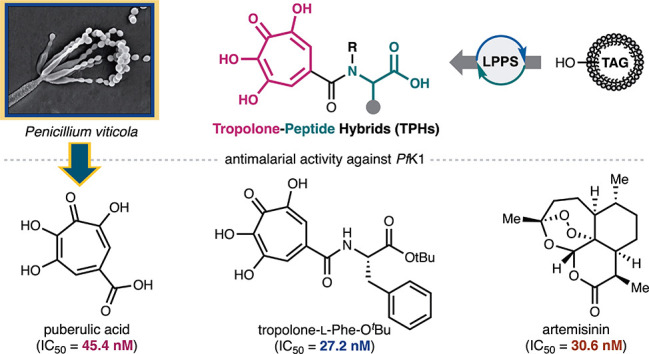

Non-benzenoid seven-membered aromatics, tropones and tropolones, exhibit atypical π-electron delocalization along with a polarized carbon–oxygen double bond that confer a strong dipole moment, enhanced Brønsted acidity (for α-tropolones), and high affinity with divalent metals.^1^ These physicochemical features have been correlated with broad bioactivity profiles across plant, fungal, and microbial secondary metabolites.^2^ To date, more than 200 naturally occurring tropolones have been identified, spanning antibacterial, antifungal, antiviral, antitumor, and enzyme-inhibitory properties.^3^ These scaffolds appear in diverse natural products (e.g., thujaplicins/hinokitiol, colchicine, and benzotropolones), and a rich synthetic toolbox exists for elaborating the seven-membered ring, via ring expansions, oxidative cycloadditions, and related strategies, facilitating medicinal exploration.^4^

As part of our ongoing efforts to explore antimalarial candidates, puberulic acid (**1**)^5^ has emerged as a potency benchmark among oxygenated troponoids (Figure 1).^6^ Isolated from *Penicillium* spp.,^7^ **1** inhibited the activity against the chloroquine-resistant *Plasmodium falciparum* K1 strain with an IC_50_ value of 50.5 nM *in vitro*. It also demonstrated *in vivo* efficacy in a *Plasmodium berghei* mouse model through subcutaneous (s.c.) administration (not shown); however, it proved to be ineffective through oral (p.o.) administration, and acute toxicity constrains its actual use as a medication. Motivated by this preliminary biological evaluation,^6^ we implemented a structure–activity relationship (SAR) campaign leveraging a concise total synthesis route of **1** ^8^ to access several congeners (e.g., viticolins and *iso*-stipitatic acid) and derivatives.^9^ As a result, it was revealed that C4 carboxylic acid is amenable to functionalization, whereas the array of four contiguous oxygen atoms on the tropone ring is largely intolerant of its modification. Although we have identified that several derivatives, such as cyclohexyl ester (**2**) and isobutyl ketone (**3**), showed promising *in vivo* efficacy at 15 mg/kg (p.o.) without toxicity,^10^ they were not effective at the dose of 5 mg/kg. We attributed this remarkable loss of the activity to the inherent planar structure that would have a competitive affinity with plasma proteins in blood.^11^ Therefore, we sought to synthesize a complementary class of derivatives that possess three-dimensionally complex structures.

**Figure 1:**
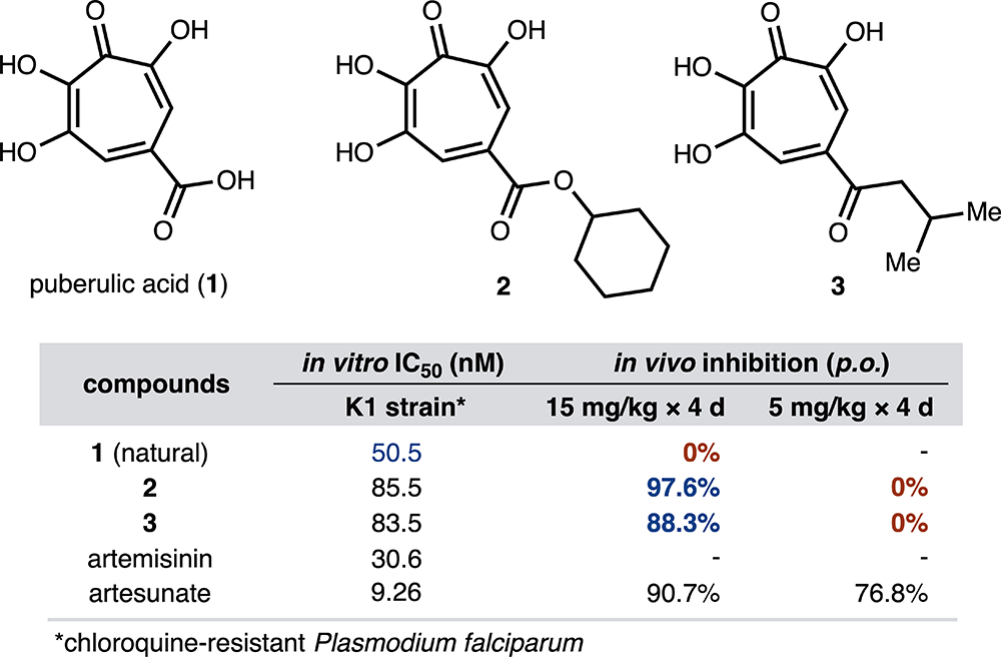
Structures and antimalarial activities of puberulic acid and related tropolones.

While many precedented methods focus on the construction of the seven-membered aromatic framework,^3,12^ subsequent derivatization of tropolones remains intrinsically challenging. The α-tropolone nucleus combines a polarized enol–ketone array, enhanced acidity, and strong chelation ability, a constellation that narrows chemoselectivity windows and promotes side reactions under conditions that would be routinely utilized for benzenoids. These intrinsic features complicate both bond-forming events and workup/purification operations, as silica or trace metals can be chelated, necessitating protecting group manipulations and/or skillful purification protocols.^13^

To circumvent these issues and with our recent success for the systematic manipulation of peptide natural products,^14^ we envisioned liquid-phase peptide synthesis (LPPS)^15^ leveraging a soluble hydrophobic tag auxiliary to enable practical tropolone derivatization. Peptides, with their inherently three-dimensional, conformationally rich architectures, represent a fundamentally different chemical space from planar tropolone scaffolds and, thus, hold considerable promise as next-generation antimalarial leads.^16^ Their structural complexity offers high target selectivity and tunable physicochemical properties that would be advantageous to address current challenges surrounding biological limitations of tropolones through modular diversification in residue-level editing.^17^ Herein, we report the synthesis of tropolone–peptide hybrid (TPH) analogues through LPPS and evaluation of their antimalarial activities.

At the outset, our investigation for the synthesis of TPH derivatives commenced with the preparation of puberulic acid (**1**) following our established route (Scheme 1A).^8^ Although the direct amidation of **1** using the method reported by Murelli and co-workers^18^ was not effective in our substrate because of its challenging purification, we found an alternative approach for this purpose. Akin to our previous synthesis of amide derivatives,^10^ **1** was first subjected to the benzylation conditions, which was followed by hydrolysis of the corresponding benzyl ester, providing carboxylic acid **5** on a multigram scale. Condensation of **5** with H-Gly-OBn using PyBOP in the presence of Hünig’s base gave the corresponding amide in 70% yield. Global deprotection using a membrane-supported palladium catalyst (Pd/iO-brane) under hydrogenolysis conditions^19^ that had worked previously provided only a trace amount of desired **6** after purification by a reverse-phase (RP) column due to non-specific decomposition. We assumed that the mono-*N*-acyl amino acid functionality in **6**, in combination with the palladium metal, caused undesired reactivities in this case.^20^ To gain more insights into preparing TPHs, we thought to synthesize tertiary amide derivatives.

**Scheme 1:**
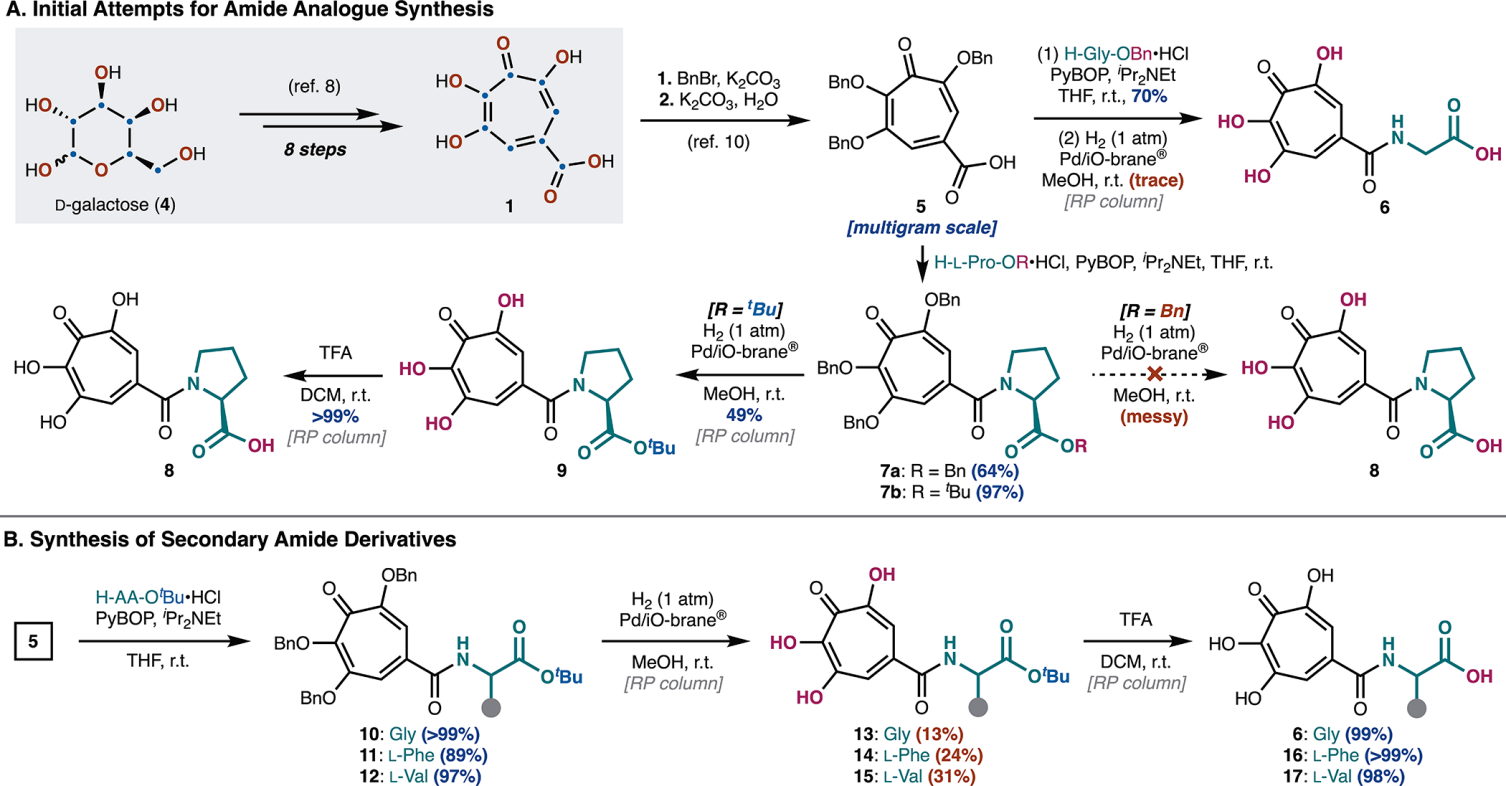
Synthetic Challenges toward Tropolone–Peptides

In this regard, carboxylic acid **5** was subjected to the condensation conditions with C-protected prolines affording benzyl ester **7a** in 64% yield and *tert*-butyl ester **7b** in 97% yield, respectively. While global removal of the benzyl groups in **7a** resulted in a complex mixture, we were pleased to find that stepwise deprotection of **7b** proved to be effective. Thus, hydrogenolysis using Pd/iO-brane worked in an acceptable yield to provide **9** (49% by RP column), and subsequent treatment with TFA gave rise to desired **8** quantitatively. Encouraged by this result, we next aimed at preparing secondary amide derivatives using an analogous sequence (Scheme 1B). After condensation of **5** with Gly, l-Phe, and l-Val (from 89 to >99% yields), cleavage of the benzyl groups in amides **10**–**12** by the established hydrogenolysis afforded tropolones **13**–**15** in unsatisfactory but synthetically useful yields (13–31%). Ultimately, the *tert*-butyl groups in **13**–**15** were removed by treatment with TFA, furnishing TPHs **6**, **16**, and **17** in excellent yields. As shown, although we were able to manipulate several tropolone–peptides, the process represented intrinsic challenges associated with the chelate ability that resulted in low-yielding deprotection and required purification by a time-consuming RP column. In order to facilitate a SAR study, we turned our attention to the LPPS protocol^21^ to enable the practical and rapid generation of TPH derivatives.

Soluble hydrophobic tagging strategies provide an effective means of modulating molecular polarity to enable streamlined liquid-phase synthesis and purification. Hydrophobic tag carriers bearing long-chain alkyl substituents promote solubility in low-polarity solvents while inducing aggregation in polar media, thereby facilitating separation through solidification and/or decantation. A representative anchor molecule (TAG–OH), reported by Tamiaki and co-workers,^22^ served as a dual-purpose protecting group and soluble tag for peptide synthesis (Figure 2). The electron-rich aromatic framework allows mild acid-mediated cleavage, while the hydrophobic chains impart the phase behavior necessary for efficient reaction/purification cycles. We envisioned that this strategy could mitigate concerns related to the inherent physicochemical properties of tropolones, thus enabling systematic manipulations of peptide hybrid compounds through LPPS.

**Figure 2:**
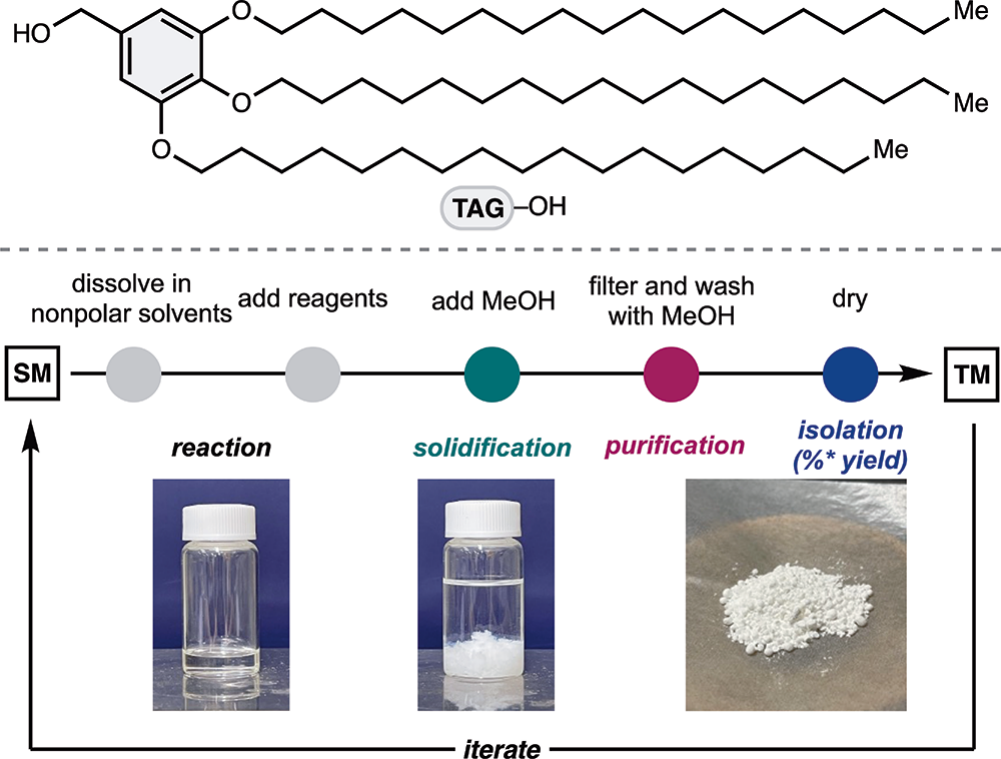
Soluble hydrophobic tagging synthetic protocol. SM, starting material; TM, targeting material.

To implement the planned LPPS approach, we prepared the carrier molecule by the modified procedure.^23^ As a result of our investigation en route to TPH analogues, the general sequence is outlined in Scheme 2A. First, TAG–OH was condensed with a Fmoc amino acid (AA), which was followed by deprotection of the N terminus, providing the corresponding TAG-supported amines (i.e., **18**, step 1). Next, the condensation of amine **18** with carboxylic acid **5** afforded the coupled products (i.e., **19**, step 2). These products after steps 1 and 2 were purified by our solidification protocol in LPPS (see the Supporting Information for details; an asterisk after the percentage shows the isolated yield by the solidification protocol). Finally, hydrogenolysis of the benzyl groups using the Pearlman’s catalyst,^24^ in which the products were briefly purified by a solidification/decantation procedure, and, subsequently, the TAG carrier was cleaved by treating with TFA. After the reaction, the TAG residue was removed by solidification and filtration to furnish the desired tropolone–peptides (**20**–**32**, step 3).

**Scheme 2:**
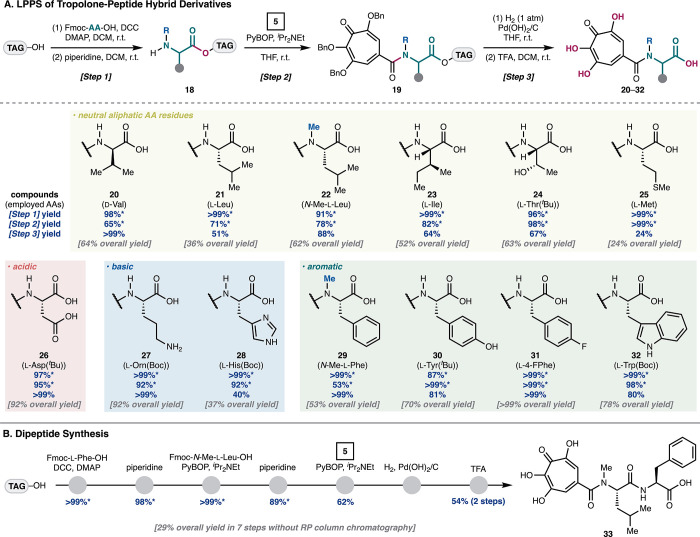
Synthesis of Tropolone–Peptide Hybrid Analogues^a^

a An asterisk “∗” indicates the yield after purification by the solidification protocol described in Figure 2.

Having established a method for the LPPS of TPHs, we explored the scope with respect to the AA residue. In direct comparison to our solution-phase synthesis of l-Val bearing tropolone **17** (described in Scheme 1B, 29% overall yield), d-Val was employed for the LPPS sequence, producing **20** in overall 64% yield (over five chemical steps) without RP column chromatography. Conventional aliphatic AA residues, such as Leu and Ile, including a *N*-methyl amino acid participated faithfully in this method to give TPHs **21**–**23** in a 36–62% overall yield. The polar Thr residue was tolerant of the process with a *tert*-butyl protecting group, which was removed under acidic conditions with the TAG carrier, providing **24** in an excellent yield. While we observed non-specific degradation in the hydrogenolysis conditions when using Met, it delivered TPH **25** in synthetically useful yield (24% over five steps). Acidic and basic AA residues, such as Asp, Orn, and His, performed well with the protecting group to give **26**–**28** in good yields. Substrates possessing aromatics, such as *N*-Me-Phe, Tyr, 4-F-Phe, and Trp, also smoothly afforded the desired products **29**–**32** (from 53 to >99% overall yields).

To test further utility of the approach, we next sought to synthesize a dipeptide analogue (Scheme 2B). In this regard, condensation of the TAG carrier with Phe and subsequent deprotection provided TAG-supported amine. Iterative condensation and deprotection, followed by amidation with tropolone **5** afforded the corresponding coupled products. Lastly, global cleavage of the benzyl groups and TAG removal gave rise to desired TPH **33** in 29% overall yield through the seven-step sequence without RP column chromatography. These results demonstrated the power of a LPPS strategy for the rapid and systematic synthesis of tropolone-containing peptidic derivatives.

The LPPS strategy described above enabled a comprehensive investigation of SARs of TPH analogues. A library of 22 synthesized derivatives was evaluated for their *in vitro* antimalarial activity against the *Pf*K1 strain (Table 1).^25^ Analogues incorporating neutral amino acid residues (highlighted in yellow) exhibited moderate IC_50_ values. Notably, a comparison of **17** (l-Val) and **20** (d-Val) revealed that the absolute configuration of the isopropyl substituent significantly influences potency, suggesting that the 3D conformation of these derivatives is recognized by the biological target. N-Methylated Leu analogue **22** displayed an order-of-magnitude improvement in potency compared to corresponding secondary amide **21**, underscoring the contribution of conformational restriction on activity. In contrast, introduction of acidic (highlighted in red) or basic (lowlighted in blue) polar residues (**26**–**28**) attenuated antimalarial activity, indicating that hydrophobic AA residues are critical for function. Within the hydrophobic aromatic series (highlighted in green), tropolone-l-Phe-O^*t*^Bu (**14**) exhibited remarkable potency with an IC_50_ of 27.2 nM, surpassing both synthetic puberulic acid (**1**) and the clinically used antimalarial drug artemisinin. Interestingly, N-methylation of the related derivative diminished potency (**16** vs **29**), further emphasizing the importance of stereochemical and conformational features in ligand recognition. Dipeptide analogue **33** showed only weak activity, suggesting steric limitations in the corresponding affinity pocket that restrict derivatization at this position.

**Table 1:**
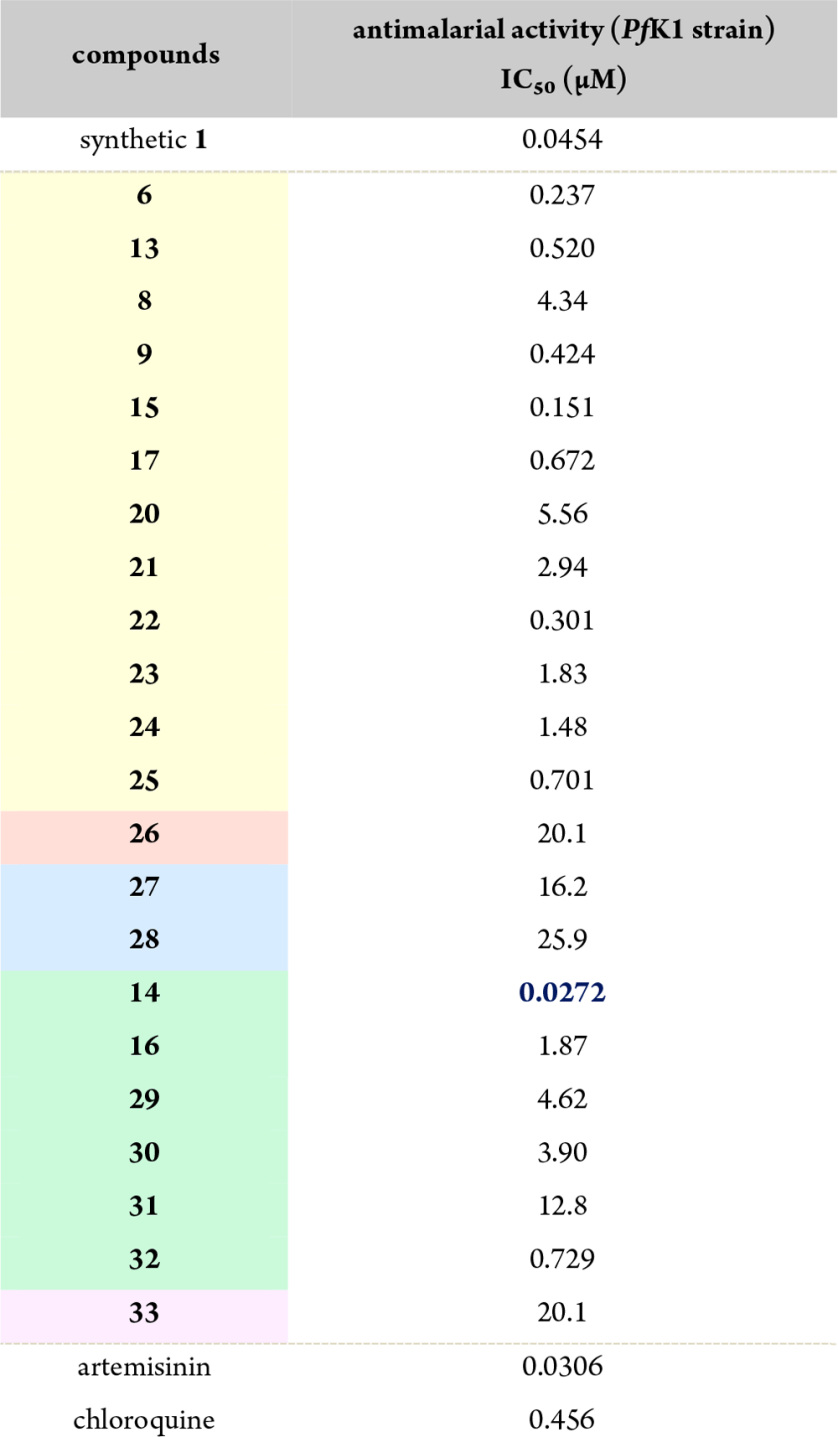
*In Vitro* Antimalarial Activity of TPHs

To further probe the influence of three-dimensional structural elements on tropolone-based scaffolds, we evaluated several TPHs against the chloroquine-sensitive *P. falciparum* FCR-3 strain in the presence and absence of human serum albumin (HSA).^26^ As a benchmark, planar ketone analogue **3** had an IC_50_ of 0.0504 μM, but its potency decreased by more than 2 orders of magnitude upon HSA addition (IC_50_ = 5.50 μM; Table S1 of the Supporting Information). In striking contrast, the most potent TPH derivative **14** retained sub-micromolar potency regardless of the HSA additive (IC_50_ = 0.130 μM). These findings support our hypothesis that introducing stereochemical and conformational complexity into the tropolone core mitigates non-specific plasma protein binding inherent to the planar framework, thereby demonstrating the utility of peptide hybrid derivatization for improving antimalarial candidate profiles.

In summary, we have developed a LPPS platform that enables the rapid, modular, and systematic construction of TPH analogues, overcoming long-standing challenges associated with the intrinsic reactivity and chelation properties of α-tropolones. This strategy streamlined access to a diverse library of derivatives without reliance on chromatographic purification, providing a robust route for comprehensive SAR interrogation. Biological evaluation highlighted the crucial role of hydrophobic and conformationally constrained amino acid residues in achieving potent *in vitro* antimalarial activity. Notably, tropolone-l-Phe-O^*t*^Bu emerged as a standout lead, surpassing the potency of both synthetic puberulic acid and artemisinin and exhibiting remarkable activity in protein-rich media. These results validate the design principle that three-dimensional peptide architectures effectively mitigate the detrimental plasma protein binding observed for planar troponoids. Collectively, this work establishes LPPS-enabled peptide hybridization as a powerful, generalizable approach for expanding the chemical space of tropolones and identifies TPH motifs as promising leads for next-generation antimalarial agent development.

## Supplementary Material



## Data Availability

The data underlying this study are available in the published article and its Supporting Information.

## Notes

Experimental procedures, spectroscopic data, and NMR spectra (PDF)
