# Supplementary material for: Liquid-Phase Peptide Synthesis of Tropolone–Peptide Hybrid Antimalarials
Source: Org Lett. 2026 Mar 26;28(14):4462–7. doi: 10.1021/acs.orglett.6c00703 (PMC13077680; doi:10.1021/acs.orglett.6c00703)
Supplement: Supplementary file 1 [file ol6c00703_si_001.pdf]

Supporting Information

Liquid-Phase Peptide Synthesis of Tropolone–Peptide Hybrid Antimalarials

Goh Sennari, Asuka Nakajima, Hiroki Nakahara, Ryo Saito, Aki Ishiyama, Rei Hokari, Masato Iwatsuki, Tomoyasu Hirose*, Toshiaki Sunazuka*

Ōmura Satoshi Memorial Institute and Graduate School of Infection Control Sciences
Kitasato University, 5-9-1 Shirokane, Minato-ku, Tokyo 108-8641, Japan

Corresponding Authors

*thirose@lisci.kitasato-u.ac.jp
*sunazuka@lisci.kitasato-u.ac.jp

1. General Considerations**.....S1**

2. Biological Evaluation**.....S2**

2-1. *In vitro* antimalarial assays in the presence and absence of HSA (Table S1).....S3

2-1. *In vitro* antimalarial activity and cytotoxicity (Table S2).....S4

3. Experimental Procedures and Characterization Data**.....S5**

3-1. Solution-phase synthesis of TPHs.....S5

3-2. Liquid-phase synthesis of TPHs.....S13

3-3. Liquid-phase synthesis of the dipeptide derivative.....S47

4. NMR and LC-UV Spectra Charts**.....S52**

1. General Considerations

• Solvents and Reagents

Unless otherwise noted, commercial reagents were purchased from Sigma Aldrich, Combi-blocks, TCI, Strem Chemicals, BLDPharm, FUJIFILM Wako Pure Chemical Co., and/or Kanto Chemical Co., and used without additional purification. Solvents were purchased from Sigma Aldrich, TCI, and/or Kanto Chemical Co., and used without additional purification (stored over molecular sieves). THF and DCM were sparged with argon and dried by passing through alumina columns using argon in a Glass Contour solvent purification system.

• Experimental Procedures

Unless otherwise noted in the experimental procedures, reactions were carried out in flame- or oven-dried glassware under a positive pressure of N_2 in anhydrous solvents using standard Schlenk techniques. Reaction temperatures above room temperature (20–25 °C) were controlled by an IKA® temperature modulator or AS ONE Co. Oil Bath and monitored using liquid-in-glass thermometers. Reaction progress was monitored by thin-layer chromatography (TLC) on Sigma Aldrich/Millipore silica gel (60 Å, F254 indicator). TLC plates were visualized by exposure to ultraviolet light (254 nm), and/or stained by submersion in *p*-anisaldehyde, ninhydrin, or phosphomolybdic acid stain and heating with a heat gun or heating plate. Organic solutions were concentrated under reduced pressure on an EYELA temperature-controlled rotary evaporator equipped with a cooling condenser. Flash column chromatography was performed with either glass columns using Kanto Chemical silica gel (60 N, spherical neutral, 40–50 μm particle size) or with a Yamazen Smart Flash EPCLC W-Prep 2XY (dual channel) automated flash chromatography system on prefilled, premium, universal columns using ACS grade solvents. All yields refer to chromatographically and spectroscopically (^{1}H and ^{13}C NMR) pure material.

• Analytical Instrumentation

^{1}H NMR and ^{13}C NMR data were recorded on JEOL JNM-ECA-500 (500 MHz for ^{1}H NMR and 125 MHz for ^{13}C NMR) spectrometers, typically at 20–23 °C. Chemical shifts (δ) are reported in ppm relative to the residual solvent signal (δ 7.26 for ^{1}H NMR & δ 77.2 or 77.0 for ^{13}C NMR in $CDCl_3$, δ 3.31 or 3.30 for ^{1}H NMR & δ 49.0 for ^{13}C NMR in CD_3OD, δ 2.05 for ^{1}H NMR & δ 206.3 for ^{13}C NMR in $(CD_3)_2CO$ and δ 2.50 for ^{1}H NMR & δ 39.5 for ^{13}C NMR in $(CD_3)_2SO$). Data for ^{1}H and ^{13}C spectroscopy are reported as follows: chemical shift (δ ppm), multiplicity (s = singlet, d = doublet, t = triplet, q = quartet, m = multiplet, br = broad, app = apparent), coupling constant (Hz), integration. High-resolution mass spectra (HRMS) were measured on a JEOL JMS-AX505HA, JEOL JMS-700 MStation and/or JEOL JMS-T100LP (analyzer type: quadrupole). LC/UV analysis was performed using an Agilent 1260 Infinity LC (Agilent Technologies inc.) with a Waters symmetry C18 Column (4.6 i.d. × 150 mm, Waters Corporation). Optical rotations were measured on a JASCO P-2020 polarimeter. The calculated HRMS values were determined using the accurate mass calculator available at https://huggingface.co/spaces/Matchball/accurate_mass, following the procedure described in the referenced article (Christmann, M. *Org. Lett.* **2025**, *27*, 4–7).

2. Biological Evaluation

In vitro cultivation of *Plasmodium falciparum* and antimalarial assay

In vitro cultivation and antimalarial activity against the *Plasmodium falciparum* K1 (chloroquine-resistant) parasite strain were measured using the method described previously.[1] Briefly, the *P. falciparum* K1 strain was cultured in human erythrocytes in RPMI medium supplemented with 10% human plasma at 37°C, under 93% N_2, 4% CO_2, and 3% O_2. Asynchronous parasites (2% hematocrit and 0.5 or 1% parasitemia) were seeded in a 96-well micro-titer plate, and serially diluted test compounds were added. Positive controls such as chloroquine and artemisinin were added in a similar fashion. After 72-hours incubation, parasite lactate dehydrogenase (p-LDH) was assayed using a slight modification of the procedure reported by Makler *et al*[2] and Vivas *et al*.[3] The 50% inhibitory concentration (IC_{50}) value was calculated from a dose response curve. This study was approved by "Kitasato Institute Hospital Research Ethics Committee" on the donation of human erythrocytes from volunteers.

Cytotoxic assay against MRC-5 cells

Measurement of cytotoxicity against human fetal lung fibroblast MRC-5 cells was carried out as described previously.[4]

[1] Otoguro, K.; Kohana, A.; Manabe, C.; Ishiyama, A.; Ui, H.; Shiomi, K.; Yamada, H.; Ōmura, S. Potent antimalarial activities of polyether antibiotic, X-206. *J. Antibiot*. **2001**, *54*, 658–663.

[2] Makler, M. T.; Ries, J. M.; Williams, J. A.; Bancroft, J. E.; Piper, R. C.; Gibbins, B. L.; Hinrichs, D. J. Parasite lactate dehydrogenase as an assay for Plasmodium falciparum drug sensitivity. *Am. J. Med. Hyg*. **1993**, *48*, 739–741.

[3] Vivas, L.; Easton, A.; Kendrick, H.; Cameron, A.; Lavandera, J.-L.; Barros, D.; de las Heras, F. G.; Brady, R. L.; Croft, S. L. Plasmodium falciparum: Stage specific effects of a selective inhibitor of lactate dehydrogenase. *Exper. Parasitol*. **2005**, *111*, 105–114.

[4] Otoguro, K.; Ui, H.; Ishiyama, A.; Arai, N.; Kobayashi, M.; Takahashi, Y.; Masuma, R.; Shiomi, K,; Yamada, H.; Ōmura, S. *In vitro* antimalarial activities of the microbial metabolites. *J. Antibiot*. **2003**, *56*, 322-324.

2-1. *In vitro* antimalarial assays in the presence and absence of HSA (Table S1)

compounds	antimalarial activity IC50 (μM)		index of HAS influence
	FCR-3	FCR-3 + HSA	FCR3 + HAS/FCR-3
synthetic **1**	0..0505	-	-
**3**	0.0504	**5.50**	**109**
**6**	0.427	1.92	4.5
**13**	0.639	0.999	1.6
**8**	4.72	10.6	2.2
**9**	0.410	0.731	1.8
**14**	**0.0623**	**0.130**	**2.1**
**16**	3.74	4.05	***1.1***
**15**	0.190	0.297	1.6
**17**	0.861	3.00	3.5
**22**	0.424	1.93	4.6
chloroquine	0.119	0.147	1.2

FCR-3: chloroquine-sensitive *Pf* strain, HAS: human serum albumin.

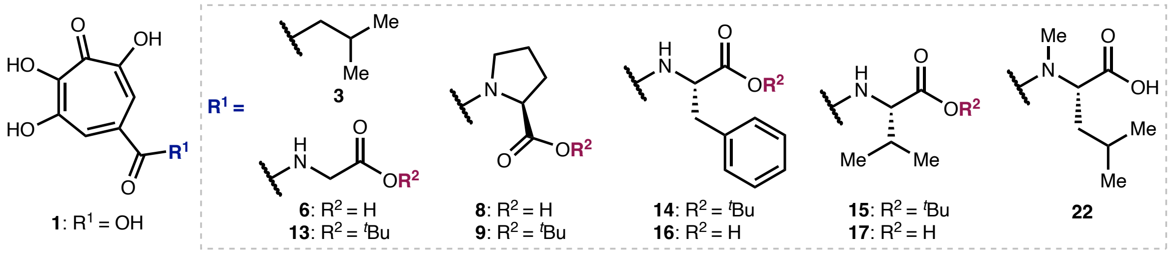

2-1. *In vitro* antimalarial activity and cytotoxicity (Table S2)

compound	IC_{50} (μM)	
	antimalarial activity (K1 strain)	cytotoxicity (MRC-5)
synthetic **1**	0.0454	21.1
**6**	0.237	38.3
**13**	0.520	1.30
**8**	4.34	119
**9**	0.424	1.67
**15**	0.151	0.00934
**17**	0.672	7.21
**20**	5.56	83.5
**21**	2.94	35.2
**22**	0.301	2.76
**23**	1.83	38.0
**24**	1.48	63.4
**25**	0.701	74.0
**26**	20.1	77.2
**27**	16.2	70.2
**28**	25.9	21.2
**14**	**0.0272**	**0.00249**
**16**	1.87	1.61
**29**	4.62	92.0
**30**	3.90	42.8
**31**	12.8	38.7
**32**	0.729	30.7
**33**	20.1	17.9
artemisinin	0.0306	>25,000
chloroquine	0.456	>25,000

3. Experimental Procedures and Characterization Data

3-1. Solution-phase synthesis of TPHs

General Procedure A: Solution-phase condensation of **5** with H-AA-O^{t}Bu

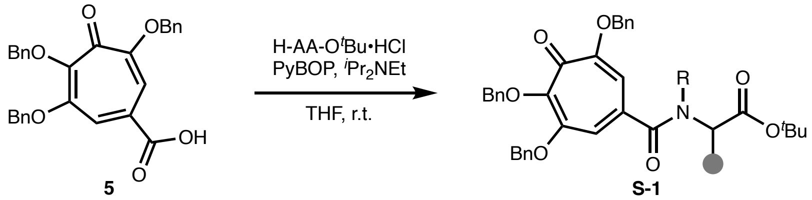

To a solution of **5** (1.0 equiv) in THF (10 mM) was added PyBOP (2.0 equiv), ^{i}Pr$_2$NEt (6.0 equiv), and H-AA-O^{t}Bu•HCl (3.0 equiv) at room temperature. After stirring for 1–3 h, the reaction mixture was quenched with 1 M HCl (ca. 5 mM) and extracted with DCM (ca. 5 mM × 3). The combined organic phase was dried over Na_2SO_4, filtered and concentrated in vacuo. The resulting residue was purified by silica gel column chromatography, yielding **S-1**.

General Procedure B: Solution-phase hydrogenolysis of **S-1**

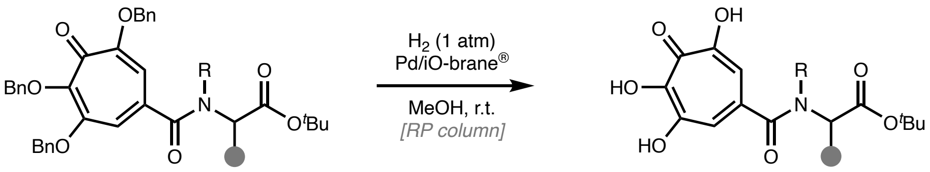

To a solution of **S-1** (1.0 equiv) in MeOH (20 mM) was added Pd/iO-brane®[5] (10 wt%) at room temperature under air. The resulting mixture was carefully evacuated by an aspirator and backfilled with H_2 × 3. After stirring for 5–10 h under a H_2 atmosphere, Pd/iO-brane® was removed by cotton-filtration. The filtrate was concentrated *in vacuo* and the resulting residue was recrystallized from MeOH/H_2O. The resulting residue was purified by reverse-phase Sep-Pak® Plus C18 Short Cartridge column chromatography, yielding **S-2**.

[5] Liguori, F.; Barbaro, P.; Giordano, C.; Sawa, H. Partial Hydrogenation Reactions over Pd-Containing Hybrid Inorganic/Polymeric Catalytic Membranes. *Appl. Catl. A: Gen.* **2013**, *459*, 81–88.

General Procedure C: Solution-phase cleavage of the ^{t}Bu group in **S-2**

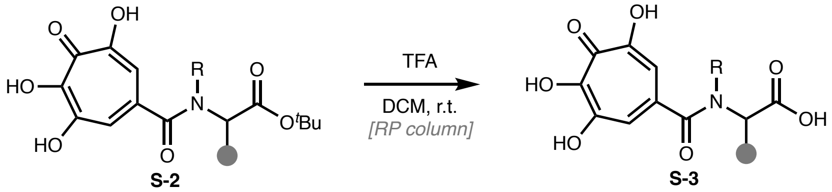

To a reaction vessel containing **S-2** (1.0 equiv) was added 50% TFA/DCM (20 mM) at room temperature under air. After stirring for 5 h, the reaction mixture was diluted with PhMe (ca. 10 mM) and the resulting solution was concentrated in vacuo. The residual acid was removed by azeotropic with PhMe three times. The resulting residue was purified by reverse-phase Sep-Pak® Plus C18 Short Cartridge column chromatography, yielding **S-3**.

• BnO-tropolone-L-Pro-O^{t}Bu (**7b**)

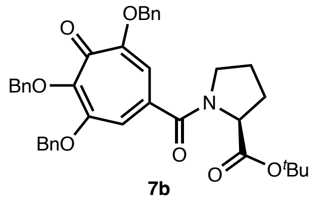

7b

Following the **General Procedure A**, **7b** (28.1 mg, 97%) as a yellow oil was obtained from **5** (21.8 mg, 46.5 μmol). The analytically pure material (^{1}H NMR) was obtained by the General Procedure.

***Rf*-value**: 0.80 (hexane/EtOAc = 2:3, stained with *p*-anisaldehyde)

HRMS (m/z): ESI $[M+Na]^+$ calculated for $C_{38}H_{39}NO_7Na$: 644.2619, found: 644.2624.

$[\alpha]_D^{23}$: –77.5 (c = 0.10, $CHCl_3$)

^{1}H NMR (500 MHz, CD_3OD): δ 7.48 – 7.42 (m, 2H), 7.41 – 7.30 (m, 10H), 7.26 – 7.24 (m, 3H), 7.15 (s, 0.7H), 7.06 (s, 0.3H), 6.92 (s, 0.7H), 6.85 (s, 0.3H), 5.36 –5.17 (m, 4H), 5.10 – 5.03 (m, 2H), 4.39 – 4.32 (m, 1H), 3.66 – 3.57 (m, 1H), 2.95 – 2.83 (m, 1H), 2.30 – 2.20 (m, 1H), 1.93 – 1.68 (m, 3H), 1.50 (s, 7H, *^{t}Bu), 1.21 (s, 2H, *^{t}Bu).

^{13}C NMR (125 MHz, CD_3OD): δ 175.5, 172.6, 171.6, 170.8, 164.5, 164.3, 160.8, 160.6, 154.3, 138.3, 137.6, 137.4, 136.8, 129.9, 129.8, 129.7, 129.4, 129.2, 129.2, 128.9, 128.7, 128.6, 119.9, 112.8, 112.3, 83.3, 83.0, 79.5, 74.7, 73.8, 73.7, 72.2, 62.7, 61.3, 50.5, 32.6, 30.3, 28.3, 28.1, 25.9, 23.7.

• BnO-tropolone-Gly-O^{t}Bu (**10**)

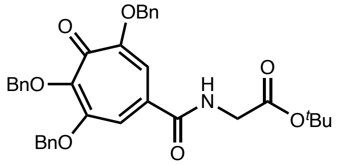

10

Following the **General Procedure A**, **10** (51.1 mg, >99%) as a yellow amorphous solid was obtained from **5** (41.2 mg, 87.9 μmol). The analytically pure material (^{1}H NMR) was obtained by the General Procedure.

R*f*-value: 0.46 (hexane/EtOAc = 2:3, stained with *p*-anisaldehyde)

HRMS (m/z): ESI $[M+Na]^+$ calculated for $C_{35}H_{35}NO_7Na$: 604.2306, found: 604.2305.

^{1}H NMR (500 MHz, CD_3OD): δ 7.62 (s, 1H), 7.51 –7.42 (m, 2H), 7.45 – 7.42 (m, 2H), 7.41 – 7.33 (m, 9H), 7.26 – 7.21 (m, 3H), 5.31 (s, 2H), 5.26 (s, 2H), 5.09 (s, 2H), 3.99 (s, 2H), 1.49 (s, 9H).

^{13}C NMR (125 MHz, CD_3OD): δ 176.0, 168.7, 163.4, 160.1, 155.6, 138.3, 137.4, 136.8, 129.9, 129.7, 129.5, 129.4, 129.3, 129.2, 129.0, 123.8, 112.8, 108.2, 75.0, 73.8, 72.2, 70.3, 30.8, 28.6, 28.3, 14.5.

• BnO-tropolone-L-Phe-O^{t}Bu (**11**)

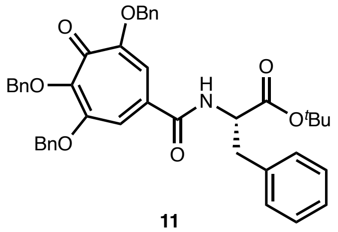

11

Following the **General Procedure A**, **11** (29.7 mg, 89%) as a yellow amorphous solid was obtained from **5** (23.3 mg, 49.7 μmol). The analytically pure material (^{1}H NMR) was obtained by the General Procedure.

R*f*-value: 0.71 (hexane/EtOAc = 1:1, stained with *p*-anisaldehyde)

HRMS (m/z): ESI $[M+Na]^+$ calculated for $C_{42}H_{41}NO_7Na$: 694.2775, found: 694.2768.

$[\alpha]_D^{24}$: +104 (c = 0.10, $CHCl_3$)

^{1}H NMR (500 MHz, $(CD_3)_2CO$): δ 7.99 (d, *J* = 8.0 Hz, 1H), 7.55 – 7.19 (m, 22H), 5.22 – 5.09 (m, 6H), 4.75 (dd, *J* = 9.2, 5.4 Hz, 1H), 3.27 (dd, *J* = 14.0, 5.4 Hz, 1H), 3.10 (dd, *J* = 14.0, 9.2 Hz, 1H), 1.45 (s, 9H).

^{13}**C NMR** (125 MHz, $(CD_3)_2CO$): δ 174.5, 171.3, 168.9, 163.3, 158.1, 154.7, 138.5, 137.5, 136.9, 134.1, 130.2, 129.3, 129.2, 128.9, 128.9, 128.6, 127.5, 121.3, 111.3, 82.1, 73.7, 71.4, 56.2, 38.0, 28.1.

• BnO-tropolone-L-Val-O^{t}Bu (**12**)

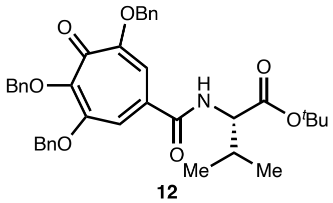

12

Following the **General Procedure A**, **12** (77.7 mg, 97%) as a yellow amorphous solid was obtained from **5** (60.5 mg, 0.129 mmol). The analytically pure material (^{1}H NMR) was obtained by the General Procedure.

R*f*-value: 0.36 (hexane/EtOAc = 1:1, stained with *p*-anisaldehyde)

HRMS (m/z): ESI $[M+Na]^+$ calculated for $C_{38}H_{41}NO_7Na$: 646.2775, found: 646.2772.

$[\alpha]_D^{24}$: +11.8 (c = 0.23, $CHCl_3$)

^{1}**H NMR** (500 MHz, $(CD_3)_2CO$): δ 7.99 (d, *J* = 8.0 Hz, 1H), 7.56 – 7.28 (m, 17H), 5.26 (s, 2H), 5.25 (s, 2H), 5.14 (s, 2H), 4.42 (m, 1H), 2.24 (m, 1H), 1.49 (s, 9H), 1.01 (d, *J* = 2.9 Hz, 3H), 1.00 (d, *J* = 2.9 Hz, 3H).

^{13}**C NMR** (125 MHz, $(CD_3)_2CO$): δ 174.6, 171.3, 169.4, 163.2, 158.1, 154.7, 138.6, 137.6, 137.1, 134.5, 129.3, 129.3, 129.3, 128.9, 128.8, 128.6, 121.6, 111.7, 81.9, 73.7, 71.3, 60.1, 31.5, 28.2, 19.6, 18.7.

• Tropolone-L-Pro-O^{t}Bu (**9**)

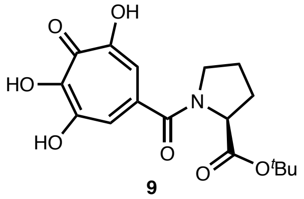

9

Following the **General Procedure B**, **9** (1.30 mg, 49%) as a brown oil was obtained from **7b** (4.70 mg, 7.57 μmol). The analytically pure material (^{1}H NMR) was obtained by the General Procedure.

HRMS (m/z): ESI $[M+Na]^+$ calculated for $C_{17}H_{21}NO_7Na$: 374.1210, found: 374.1206.

[α]$_D$^{21}: –31.6 (c = 0.10, MeOH)

^{1}H NMR (500 MHz, CD_3OD): δ 7.11 (s, 1.4H), 7.00 (s, 0.6H), 4.44 (dd, *J* = 8.5, 5.0 Hz, 0.6H, *α-proton), 4.30 – 4.27 (br, 0.4H, *α-proton), 3.72 – 3.61 (m , 0.8H), 3.55 – 3.43 (m, 1.2H), 2.39 – 2.30 (m, 1H), 2.05 – 1.87 (m, 3H), 1.50 (s, 6H, *^{t}Bu), 1.30 (s, 3H, *^{t}Bu).

^{13}C NMR (125 MHz, CD_3OD): δ 172.7, 171.4, 158.9, 158.1, 158.0, 136.5, 117.5, 117.3, 117.2, 83.5, 83.0, 63.5, 61.5, 60.6, 51.2, 32.4, 30.6, 30.5, 28.2, 28.0, 26.1, 23.6.

• Tropolone-Gly-O^{t}Bu (**13**)

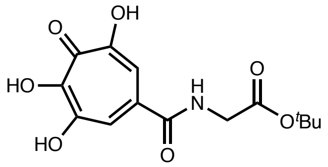

13

Following the **General Procedure B**, **13** (3.80 mg, 13%) as a yellow amorphous solid was obtained from **10** (55.1 mg, 94.8 μmol). The analytically pure material (^{1}H NMR) was obtained by the General Procedure.

HRMS (m/z): ESI $[M+Na]^+$ calculated for $C_{14}H_{17}NO_7Na$: 334.0897, found: 334.0903.

^{1}H NMR (500 MHz, CD_3OD): δ 9.00 (t, *J* = 6.0 Hz, 1H), 7.43 (s, 2H), 3.88 (d, *J* = 5.7 Hz, 2H).

^{13}C NMR (125 MHz, CD_3OD): δ 171.3, 170.4, 159.4, 157.3, 134.2, 117.9, 83.0, 43.6, 28.3.

• Tropolone-L-Phe-O^{t}Bu (**14**)

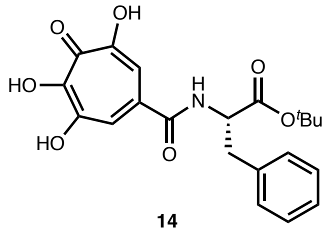

14

Following the **General Procedure B**, **14** (11.2 mg, 24%) as a brown amorphous solid was obtained from **11** (75.0 mg, 0.117 mmol). The analytically pure material (^{1}H NMR) was obtained by the General Procedure.

HRMS (m/z): ESI $[M+Na]^+$ calculated for $C_{21}H_{23}NO_7Na$: 424.1367, found: 424.1390.

[α]$_D$^{23}: +74.5 (c = 0.38, MeOH)

^{1}H NMR (500 MHz, CD_3OD): δ 7.33 – 7.22 (m, 7H), 4.68 – 4.63 (dd, *J* = 14.0, 9.2 Hz, 1H), 3.25 – 3.17 (m, 1H), 3.09 – 3.01 (m, 1H), 1.44 (s, 9H).

^{13}C NMR (125 MHz, CD_3OD): δ 172.2, 171.1, 157.2, 138.5, 130.3, 129.5, 127.9, 117.9, 83.1, 79.5, 57.0, 38.2, 28.2.

• Tropolone-L-Val-O^{t}Bu (**15**)

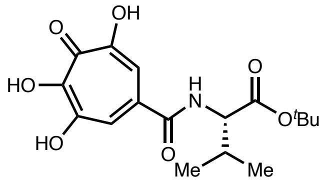

15

Following the **General Procedure B**, **15** (5.00 mg, 31%) as a brown amorphous solid was obtained from **12** (28.1 mg, 45.1 μmol). The analytically pure material (^{1}H NMR) was obtained by the General Procedure.

HRMS (m/z): ESI $[M+Na]^+$ calculated for $C_{17}H_{23}NO_7Na$: 376.1367, found: 376.1367.

$[\alpha]_D^{24}$: +48.5 (c = 0.72, MeOH)

^{1}H NMR (500 MHz, (CD_3OD): δ 8.62 (d, *J* = 7.0 Hz, 1H), 7.44 (s, 2H), 4.31 – 4.28 (m, 1H), 2.26 – 2.20 (m, 1H), 1.50 (s, 9H), 1.04 (d, *J* = 6.5 Hz, 6H).

^{13}C NMR (125 MHz, CD_3OD): δ 159.8, 157.3, 134.2, 118.0, 83.0, 61.3, 31.6, 28.3, 19.6, 19.0

• Tropolone-L-Pro-OH (**8**)

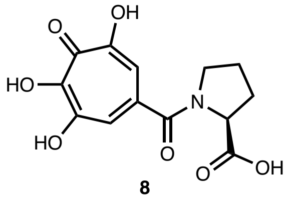

8

Following the **General Procedure C**, **8** (5.05 mg, >99%) as a brown oil was obtained from **9** (6.00 mg, 17.1 μmol). The analytically pure material (^{1}H NMR) was obtained by the General Procedure.

HRMS (m/z): ESI $[M+Na]^+$ calculated for $C_{13}H_{13}NO_7Na$: 318.0584, found: 318.0597.

$[\alpha]_D^{22}$: –38.8 (c = 0.10, MeOH with 0.1% TFA)

^{1}H NMR (500 MHz, CD_3OD): δ 7.18 – 7.02 (br, 2H), 4.59 – 4,33 (br, 1H), 3.75 – 3.45 (br, 2H), 2.45 – 1.87 (br, 4H)

^{13}C NMR (125 MHz, CD_3OD): δ 175.3, 172.2, 158.1, 146.1, 133.7, 117.3, 60.6, 51.2, 30.6, 26.1.

• Tropolone-Gly-OH (**6**)

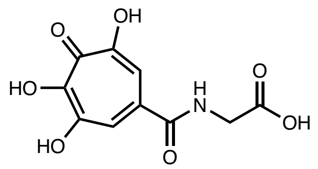

6

Following the **General Procedure C**, **6** (8.20 mg, 99%) as a brown amorphous solid was obtained from **13** (10.1 mg, 32.5 μmol). The analytically pure material (^{1}H NMR) was obtained by the General Procedure.

HRMS (m/z): ESI $[M+Na]^+$ calculated for $C_{10}H_9NO_7Na$: 278.0271, found: 278.0285.

^{1}H NMR (500 MHz, $(CD_3)_2SO$): δ 9.00 (t, *J* = 6.0 Hz, 1H), 7.43 (s, 2H), 3.88 (d, *J* = 5.7 Hz, 2H).

^{13}C NMR (125 MHz, $(CD_3)_2SO$): δ 171.1, 167.7, 158.1, 155.2, 131.4, 116.6, 41.7.

• Tropolone-L-Phe-OH (**16**)

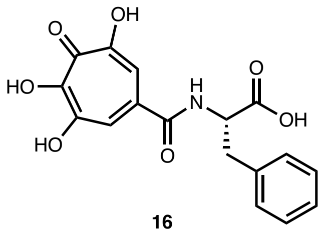

16

Following the **General Procedure C**, **16** (4.73 mg, >99%) as a brown oil was obtained from **14** (5.50 mg, 13.7 μmol). The analytically pure material (^{1}H NMR) was obtained by the General Procedure.

HRMS (m/z): ESI $[M+Na]^+$ calculated for $C_{17}H_{15}NO_7Na$: 368.0741, found: 368.0757.

$[\alpha]_D^{21}$: –75.8 (c = 0.075, MeOH with 0.1% TFA)

^{1}H NMR (500 MHz, CD_3OD): δ 7.32 – 7.18 (m, 7H), 4.82 – 4.77 (m, 1H), 3.34 (dd, *J* = 14.0, 5.0 Hz, 1H), 3.06 (dd, *J* = 14.0, 10.5 Hz, 1H).

^{13}C NMR (125 MHz, CD_3OD): δ 174.6, 171.1, 157.3, 138.7, 130.2, 129.5, 127.8, 117.9, 79.5, 56.1, 49.3, 38.1.

• Tropolone-L-Val-OH (**17**)

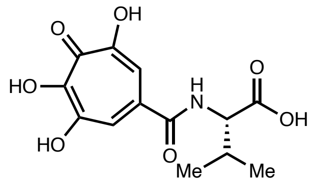

17

Following the **General Procedure C**, **17** (8.90 mg, 98%) as a brown oil was obtained from **15** (10.8 mg, 30.6 μmol). The analytically pure material (^{1}H NMR) was obtained by the General Procedure.

HRMS (m/z): ESI $[M+Na]^+$ calculated for $C_{13}H_{15}NO_7Na$: 320.0741, found: 320.0749.

$[\alpha]_D^{22}$: +16.8 (c = 0.10, MeOH with 0.1% TFA)

^{1}H NMR (500 MHz, CD_3OD): δ 7.55 (s, 2H), 4.44 (d, J = 6.5 Hz, 1H), 2.31 – 2.24 (m, 1H), 1.05 (d, J = 3.0 Hz, 3H), 1.03 (d, J = 3.0 Hz, 3H).

^{13}C NMR (125 MHz, CD_3OD): δ 174.7, 171.6, 159.4, 157.3, 134.7, 118.1, 60.3, 31.6, 19.7, 18.8.

3-2. Liquid-phase synthesis of TPHs

General Procedure D: Condensation of TAG-OH with Fmoc-AA-OH

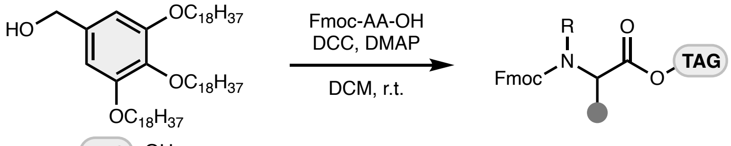

S-4

3,4,5-Tris(octadecyloxy)benzyl alcohol (TAG-OH) was prepared according to the literature procedure.[6] To a solution of TAG-OH (1.0 equiv) and Fmoc-AA-OH (1.3 equiv) in DCM (20 mM) was added DMAP (13 mol%) and a solution of DCC (2.0 equiv) in DCM (0.5 M) slowly at room temperature. After stirring for 1–3 h, the reaction mixture was cooled down to 0 °C and MeOH (4 mM) was added. The resulting suspension was stirred for 10 min at 0 °C before the solid was collected by vacuum filtration. The resulting solid residue was washed with excess MeOH, yielding **S-4** as a white powder.

General Procedure E: Removal of the Fmoc group in **S-4**

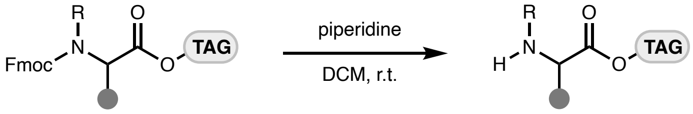

S-4 **18**

To a reaction vessel containing **S-4** (1.0 equiv) was added 10% piperidine/DCM (20 mM) at room temperature. After stirring for 1–3 h, the reaction mixture was cooled down to 0 °C and MeOH (4 mM) was added. The resulting suspension was stirred for 10 min at 0 °C before the solid was collected by vacuum filtration. The resulting solid residue was washed with excess MeOH, yielding **18** as a white powder.

[6] Hirose, T.; Kasai, T.; Akimoto, T.; Endo, A.; Sugawara, A.; Nagasawa, K.; Shiomi, K.; Ōmura, S.; Sunazuka, T. Solution-Phase Total Synthesis of the Hydrophilic Natural Product Argifin Using 3,4,5-Tris(Octadecyloxy)Benzyl Tag. *Tetrahedron* **2011**, *67*, 6633–6643.

General Procedure F: Condensation of **18** with **5**

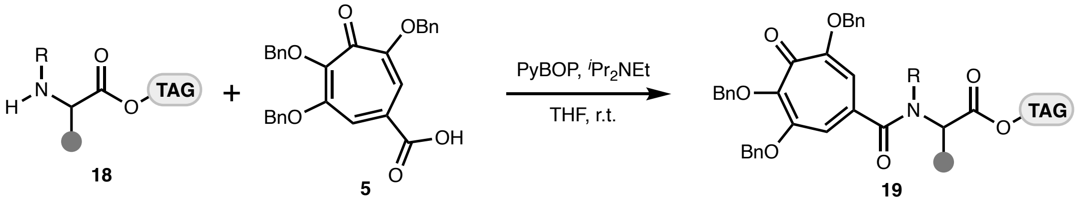

To a solution of **5** (1.1 equiv) in THF (10 mM) was added PyBOP (2.0 equiv), ^{i}Pr$_2$NEt (6.0 equiv), and **18** (1.0 equiv) at room temperature. After stirring for 1–10 h, the reaction mixture was cooled down to 0 °C and MeOH (2 mM) was added. The resulting suspension was stirred for 10 min at 0 °C before the solid was collected by vacuum filtration. The resulting solid residue was washed with excess MeOH, yielding **19** as a white powder

General Procedure G: Deprotection and TAG-removal of **19**

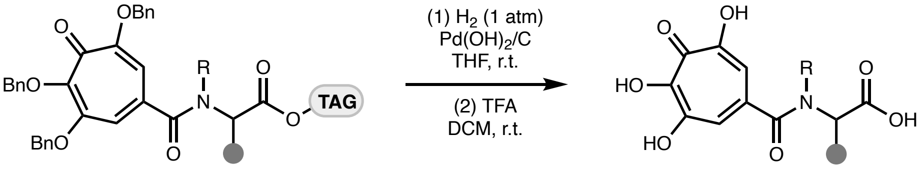

To a solution of **19** (1.0 equiv) in THF (10 mM) was added $Pd(OH)_2/C$ (10 wt%) at room temperature under air. The resulting mixture was carefully evacuated by an aspirator and backfilled with $H_2 \times 3$. After stirring for 5–8 h under a H_2 atmosphere, the reaction mixture was cooled down to 0 °C and MeOH (2 mM) was added. The resulting suspension was stirred for 10 min at 0 °C and centrifuged at 40×10^3 rpm for 7 min before the supernatant was removed by decantation. The resulting solid residue was washed with MeOH (2 mM) by repeating the centrifuge separation described above (a total of three washes). The resulting residue was dissolved in DCM (10 mM) before the resulting mixture was centrifuged at 40×10^3 rpm for 7 min. After removing the resulting sediment by filtration, the filtrate was concentrated *in vacuo*. This crude material was used in the next reaction without further purification.

To a reaction vessel containing the crude product (1.0 equiv) was added 33% TFA/DCM (10 mM) at room temperature under air. After stirring for 3 h, the reaction mixture was diluted with DCM (5 mM) and concentrated in vacuo. To the resulting residue was added MeOH (5 mM) and the suspension was centrifuged at 40×10^3 rpm for 7 min before the sediment was removed by decantation and the supernatant was concentrated *in vacuo*. After repeating this cycle three times, the supernatant was filtered through a pad of Celite®. The filtrate was concentrated in vacuo and dried under high vacuum, yielding **S-5**.

• Fmoc-D-Val-OTAG (**S-4a**)

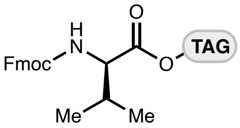

S-4a

Following the **General Procedure D**, **S-4a** (138 mg, 98%) as a white powder was obtained from TAG-OH (104 mg, 0.114 mmol). The analytically pure material (^{1}H NMR) was obtained by the General Procedure.

R*f*-value: 0.68 (hexane/EtOAc = 3:1, stained with phosphomolybdic acid)

HRMS (m/z): FAB $[M+Na]^+$ calculated for $C_{81}H_{135}NO_7Na$: 1257.0131, found: 1257.0149.

$[\alpha]_D^{25}$: +2.90 (c = 0.10, $CHCl_3$)

^{1}H NMR (500 MHz, $CDCl_3$): δ 7.77 (d, *J* = 7.5 Hz, 2H), 7.60 (d, *J* = 7.5 Hz, 2H), 7.39 (t, *J* = 7.5 Hz, 2H), 7.31 (t, *J* = 7.5 Hz, 2H), 6.53 (s, 2H), 5.30 (d, *J* = 9.2 Hz, 1H), 5.11 (d, *J* = 12.0 Hz, 1H), 5.04 (d, *J* = 12.0 Hz, 1H), 4.40 – 4.34 (m, 2H), 4.24 (t, *J* = 6.3 Hz, 1H), 3.93 (m, 6H), 2.17 (br, 1H), 1.79 – 1.71 (m, 6H), 1.46 – 1.42 (m, 6H), 1.25 (br, 85H), 0.96 – 0.87 (m, 15H).

^{13}C NMR (125 MHz, $CDCl_3$): δ 172.3, 156.5, 153.4, 144.1, 144.0, 141.5, 130.5, 128.0, 127.3, 135.3, 120.2, 107.3, 73.6, 69.4, 67.7, 67.3, 59.2, 47.4, 32.2, 31.6, 30.6, 26.3, 19.2, 17.8, 14.4.

• Fmoc-L-Leu-OTAG (**S-4b**)

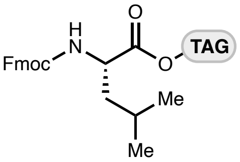

S-4b

Following the **General Procedure D**, **S-4b** (133 mg, >99%) as a white powder was obtained from TAG-OH (96.6 mg, 0.106 mmol). The characterization data of **S-4b** have been reported previously (*Angew. Chem. Int. Ed.* **2025**, *64*, e202416770.).

• Fmoc-*N*-Me-L-Leu-OTAG (**S-4c**)

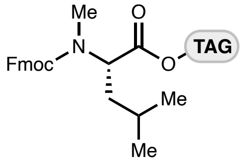

S-4c

Following the **General Procedure D**, **S-4c** (428 mg, >99%) as a white powder was obtained from TAG-OH (300 mg, 0.328 mmol). The analytically pure material (^{1}H NMR) was obtained by the General Procedure.

R*f*-value: 0.64 (hexane/EtOAc = 3:1, stained with phosphomolybdic acid)

HRMS (m/z): FAB $[M+Na]^+$ calculated for $C_{83}H_{139}NO_7Na$: 1285.0444, found: 1285.0466.

$[\alpha]_D^{23}$: –7.12 (c = 0.10, $CHCl_3$)

^{1}H NMR (500 MHz, $CDCl_3$): δ 7.79 – 77.1 (m, 4H), 7.60 – 7.51 (m, 2H), 7.42 – 7.33 (m, 2H), 7.32 – 7.28 (m, 1H), 7.25 – 7.19 (m, 1H), 6.52 – 6.46 (m 2H), 5.00 – 4.95 (m, 2H), 4.65 – 4.14 (m, 3H), 3.95 – 3.85 (m, 6H), 2.89 – 2.84 (m, 3H, **N*-Me), 1.78 – 1.62 (m, 8H), 1.53 – 1.36 (m, 7H), 1.36 – 1.10 (m, 84H), 0.98 – 0.78 (m, 15H).

^{13}C NMR (125 MHz, $CDCl_3$): δ 172.1, 171.7, 157.0, 156.5, 153.2, 144.2, 144.0, 141.3, 138.2, 130.5, 127.8, 127.2, 125.2, 125.1, 125.0, 120.1, 106.7, 73.5, 69.2, 67.8, 67.7, 67.2, 56.9, 53.5, 47.4, 37.9, 37.6, 32.1, 30.6-29.5, 26.4, 25.2, 22.8, 21.7, 14.2.

• Fmoc-L-Ile-OTAG (**S-4d**)

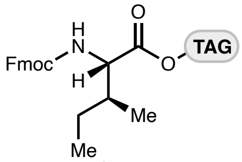

S-4d

Following the **General Procedure D**, **S-4d** (133 mg, >99%) as a white powder was obtained from TAG-OH (95.5 mg, 0.105 mmol). The analytically pure material (^{1}H NMR) was obtained by the General Procedure.

R*f*-value: 0.46 (hexane/EtOAc = 6:1, stained with phosphomolybdic acid)

HRMS (m/z): FAB $[M+Na]^+$ calculated for $C_{82}H_{137}NO_7Na$: 1271.0287, found: 1271.0302.

$[\alpha]_D^{25}$: –2.90 (c = 0.10, $CHCl_3$)

^{1}H NMR (500 MHz, $CDCl_3$): δ 7.76 (d, *J* = 7.4 Hz, 2H), 7.59 (d, *J* = 7.4 Hz, 2H), 7.40 (t, *J* = 7.4 Hz, 2H), 7.31 (t, *J* = 7.4 Hz, 2H), 6.53 (s, 2H), 5.31 (d, *J* = 9.2 Hz, 1H), 5.12 (d, *J* = 12.0 Hz, 1H), 5.03 (d, *J* = 12.0 Hz, 1H), 4.39 (d, *J* = 7.4 Hz, 2H), 4.23 (t, *J* = 7.4 Hz, 1H), 3.97 – 3.91 (m, 6H), 1.91 (br, 1H), 1.79 – 1.71 (m, 6H), 1.46 – 1.43 (br, 6H), 1.26 (br, 85H), 0.93 – 0.86 (m, 17H).

^{13}C NMR (125 MHz, $CDCl_3$): δ 172.1, 156.2, 153.3, 144.0, 141.4, 138.4, 130.4, 127.8, 127.2, 125.2, 120.1, 107.2, 73.6, 69.3, 67.3, 58.5, 47.3, 38.3, 32.1, 30.5, 29.7, 26.3, 25.1, 22.8, 15.6, 14.3, 11.8.

• Fmoc-L-Thr(^{t}Bu)-OTAG (**S-4e**)

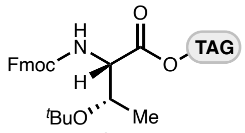

S-4e

Following the **General Procedure D**, **S-4e** (548 mg, >99%) as a white powder was obtained from TAG-OH (347 mg, 0.380 mmol). The analytically pure material (^{1}H NMR) was obtained by the General Procedure.

R*f*-value: 0.75 (hexane/EtOAc = 3:1, stained with phosphomolybdic acid)

HRMS (m/z): FAB $[M+Na]^+$ calculated for $C_{84}H_{141}NO_8Na$: 1315.0549, found: 1315.0544.

$[\alpha]_D^{23}$: +1.24 (c = 0.10, $CHCl_3$)

^{1}H NMR (500 MHz, $CDCl_3$): δ 7.76 (d, *J* = 7.4 Hz, 2H), 7.63 (t, *J* = 7.4 Hz, 2H), 7.40 (t, *J* = 7.4 Hz, 2H), 7.31 (t, *J* = 7.4 Hz, 2H), 6.54 (s, 2H), 5.64 (dd, *J* = 12.0, 12.0 Hz, 1H), 5.09 (d, *J* = 12.0 Hz, 1H), 4.97 (d, *J* = 12.0 Hz, 1H), 4.43 – 4.23 (m, 4H), 3.95 – 3.90 (m, 6H), 1.78 – 1.70 (m, 6H), 1.47 – 1.41 (m, 6H), 1.25 (br, 85H), 1.10 (s, 9H), 0.88 (m, 12H).

^{13}C NMR (125 MHz, $CDCl_3$): δ 171.2, 157.0, 153.3, 144.2, 143.9, 141.4, 138.5, 130.2, 127.8, 127.2, 125.4, 125.3, 120.1, 107.5, 74.2, 73.6, 69.3, 67.9, 67.5, 67.4, 60.1, 47.3, 32.1, 30.5, 29.9, 29.8, 29.6, 29.5, 28.5, 26.3, 22.8, 21.1, 14.3.

• Fmoc-L-Met-OTAG (**S-4f**)

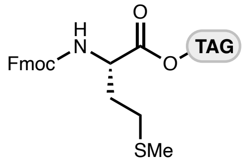

S-4f

Following the **General Procedure D**, **S-4f** (181 mg, >99%) as a white powder was obtained from TAG-OH (130 mg, 0.143 mmol). The analytically pure material (^{1}H NMR) was obtained by the General Procedure.

R*f*-value: 0.48 (hexane/EtOAc/AcOH = 4:1:0.1, stained with phosphomolybdic acid)

HRMS (m/z): FAB $[M+Na]^+$ calculated for $C_{81}H_{135}NO_7SNa$: 1288.9851, found: 1288.9874.

$[\alpha]_D^{25}$: +1.84 (c = 0.10, $CHCl_3$)

^{1}H NMR (500 MHz, $CDCl_3$): δ 7.76 (d, *J* = 7.4 Hz, 2H), 7.59 (d, *J* = 7.4 Hz, 2H), 7.40 (t, *J* = 7.4 Hz, 2H), 7.31 (t, *J* = 7.4 Hz, 2H), 6.52 (s, 2H), 5.12 – 5.05 (d, *J* = 12.2 Hz, 2H), 4.54 (m 1H), 4.41 (d, *J* = 6.9 Hz, 2H), 4.23 (t, *J* = 6.9 Hz, 1H), 3.95 – 3.92 (m, 6H), 2.48 (m, 2H), 2.04 (s, 3H), 1.80 – 1.71 (m, 6H), 1.46 – 1.45 (m, 6H), 1.25 (br, 87H), 0.88 (t, *J* = 6.9 Hz, 9H).

^{13}C NMR (125 MHz, $CDCl_3$): δ 172.0, 155.9, 153.4, 143.8, 141.4, 138.5, 130.1, 127.9, 127.2, 125.2, 120.2, 107.1, 73.6, 69.3, 67.9, 67.2, 53.4, 47.3, 32.1, 30.5, 29.9, 29.8, 29.6, 29.6, 29.5, 26.3, 22.8, 15.6, 14.3.

• Fmoc-L-Asp(^{t}Bu)-OTAG (**S-4g**)

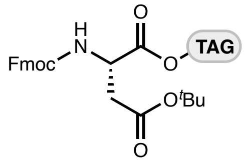

S-4g

Following the **General Procedure D**, **S-4g** (138 mg, 97%) as a white powder was obtained from TAG-OH (99.5 mg, 0.109 mmol). The analytically pure material (^{1}H NMR) was obtained by the General Procedure.

R*f*-value: 0.33 (hexane/EtOAc = 3:1, stained with phosphomolybdic acid)

HRMS (m/z): FAB $[M+Na]^+$ calculated for $C_{84}H_{139}NO_9Na$: 1329.0342, found: 1329.0372.

$[\alpha]_D^{23}$: +7.78 (c = 0.10, $CHCl_3$)

^{1}H NMR (500 MHz, $CDCl_3$): δ 7.76 (d, *J* = 7.4 Hz, 2H), 7.59 (d, *J* = 7.4 Hz, 2H), 7.39 (t, *J* = 7.4 Hz, 2H), 7.30 (t, *J* = 7.4 Hz, 2H), 6.54 (s, 2H), 5.77 (d, *J* = 16.6 Hz, 1H), 5.07 (d, *J* = 12.0 Hz, 1H), 4.99 (d, *J* = 12.0 Hz, 1H), 4.55 (br, 1H), 4.36 (m, 2H), 4.23 (t, *J* = 6.9 Hz, 1H), 3.96 – 3.90 (m, 6H), 3.01 (dd, *J* = 16.6, 4.6 Hz, 1H), 2.91 (dd, *J* = 16.6, 4.6 Hz, 1H), 1.75 (m, 6H), 1.42 (br, 15H), 1.25 (br, 84H), 0.88 – 0.83 (m, 9H).

^{13}C NMR (125 MHz, $CDCl_3$): δ 170.9, 169.7, 156.1, 153.4, 144.0, 143.9, 141.4, 138.5, 130.4, 127.8, 127.2, 125.3, 120.1, 107.2, 82.8, 73.6, 69.3, 67.4, 51.0, 47.2, 37.1, 32.1, 30.5, 29.9, 29.8, 29.6, 29.6, 29.5, 28.0, 26.3, 22.8, 14.3.

• Fmoc-L-Orn(Boc)-OTAG (**S-4h**)

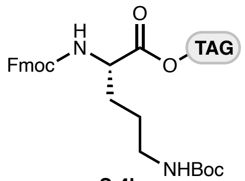

S-4h

Following the **General Procedure D**, **S-4h** (162 mg, >99%) as a white powder was obtained from TAG-OH (110 mg, 0.120 mmol). The analytically pure material (^{1}H NMR) was obtained by the General Procedure.

R*f*-value: 0.33 (hexane/EtOAc = 3:1, stained with phosphomolybdic acid)

HRMS (m/z): FAB $[M+Na]^+$ calculated for $C_{86}H_{144}N_2O_9Na$: 1372.0764, found: 1372.0774.

$[\alpha]_D^{23}$: +2.14 (c = 0.10, $CHCl_3$)

^{1}H NMR (500 MHz, $CDCl_3$): δ 7.75 (d, *J* = 7.4 Hz, 2H), 7.59 (d, *J* = 7.4 Hz, 2H), 7.40 (t, *J* = 7.4 Hz, 2H), 7.31 (t, *J* = 7.4 Hz, 2H), 6.52 (s, 2H), 5.44 (br, 1H), 5.06 (dd, *J* = 13.0, 12.0 Hz, 2H), 4.40 (d, *J* = 7.4 Hz, 2H), 4.21 (t, *J* = 7.2 Hz, 1H), 3.95 – 3.91 (m, 6H), 3.11 (s, 2H), 1.90 (m, 2H), 1.79 – 1.70 (m, 8H), 1.44 (br, 6H), 1.25 (br, 95H), 0.88 (t, *J* = 7.2 Hz, 9H).

^{13}C NMR (125 MHz, $CDCl_3$): δ 172.4, 156.1, 153.4, 144.0, 141.5, 138.5, 130.2, 127.9, 127.2, 125.2, 120.1, 107.2, 73.6, 69.3, 67.1, 47.3, 32.1, 30.5, 29.9, 29.8, 29.6, 29.6, 29.5, 28.5, 26.3, 22.8, 14.3.

• Fmoc-L-His(Boc)-OTAG (**S-4i**)

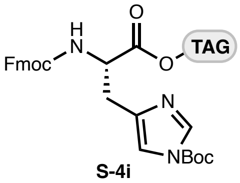

S-4i

Following the **General Procedure D**, **S-4i** (504 mg, >99%) as a white powder was obtained from TAG-OH (317 mg, 0.347 mmol). The analytically pure material (^{1}H NMR) was obtained by the General Procedure.

R*f*-value: 0.50 (hexane/EtOAc = 2:1, stained with phosphomolybdic acid)

$[\alpha]_D^{22}$: +1.62 (c = 0.10, $CHCl_3$)

^{1}H NMR (500 MHz, $CDCl_3$): δ 8.18 (s, 1H), 7.75 (d, *J* = 7.5 Hz, 2H), 7.60 (dd, *J* = 13.2, 7.5 Hz, 2H), 7.38 (t, *J* = 7.5 Hz, 2H), 7.29 (dd, *J* = 13.2, 7.5 Hz, 2H), 7.20 (s, 1H), 6.54 (s, 2H), 5.09 (br s, 2H), 4.71 (br, 1H), 4.34 – 4.27 (m, 2H), 4.21 (m, 1H), 3.94 – 3.89 (m, 6H), 3.22 (br, 2H), 1.78 – 1.71 (m, 6H), 1.61 (s, 9H), 1.45 – 1.42 (m, 6H), 1.25 (m, 85H), 0.87 (t, *J* = 6.9 Hz, 9H).

^{13}C NMR (125 MHz, $CDCl_3$): δ 171.3, 156.2, 153.3, 146.4, 144.0, 141.4, 138.4, 136.6, 129.5-130.3, 127.8, 127.2, 125.4, 120.1, 115.1, 107.1, 73.5, 69.3, 67.6, 53.8, 47.2, 32.1, 28.0, 26.3, 22.8, 14.3.

Note: HRMS using any routine conditions including Fast Atom Bombardment (FAB) could not be conducted due to its physicochemical property.

• Fmoc-*N*-Me-L-Phe-OTAG (**S-4j**)

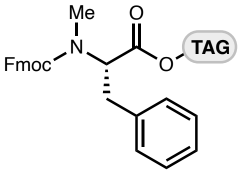

S-4j

Following the **General Procedure D**, **S-4j** (484 mg, >99%) as a white powder was obtained from TAG-OH (332 mg, 0.363 mmol). The analytically pure material (^{1}H NMR) was obtained by the General Procedure.

R*f*-value: 0.59 (hexane/EtOAc = 3:1, stained with phosphomolybdic acid)

HRMS (m/z): FAB $[M+Na]^+$ calculated for $C_{86}H_{137}NO_7Na$: 1319.0287, found: 1319.0282.

$[\alpha]_D^{23}$: –12.2 (c = 0.10, $CHCl_3$)

^{1}H NMR (500 MHz, $CDCl_3$): δ 7.79 – 7.72 (br, 2H), 7.54 – 7.45 (m, 2H), 7.44 – 7.33 (m, 2H), 7.30 – 7.27 (m, 5H), 7.24 – 7.18 (br, 2H), 7.04 – 6.99 (br, 1H), 6.54 – 6.47 (m, 2H), 5.12 – 4.95 (m, 2H), 4.75 – 4.68 (m, 0.5H, *α-proton), 4.52 – 4.46 (m, 0.5H, *α-proton), 4.38 – 4.09 (m, 3H), 3.98 – 3.86 (m, 6H), 3.44 – 3.37 (m, 1H), 3.23 – 3.04 (m, 1H), 2.87 – 2.78 (m, 3H, **N*-Me), 1.80 – 1.70 (m, 6H), 1.50 – 1.39 (br, 6H), 1.39 – 1.19 (br, 84H), 0.92 – 0.84 (m, 9H).

^{13}C NMR (125 MHz, $CDCl_3$): δ 171.0, 156.7, 153.3, 144.0, 141.5, 138.3, 137.2, 130.6, 129.0, 128.7, 127.8, 125.1, 123.1, 120.1, 107.0, 100.1, 69.3, 67.9, 67.5, 60.5, 47.2, 35.0, 32.1, 30.5, 29.9, 29.8, 29.6, 29.5, 26.3, 22.8, 14.3.

• Fmoc-L-Tyr(^{t}Bu)-OTAG (**S-4k**)

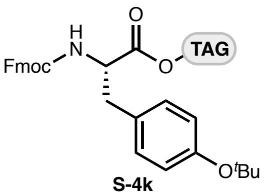

S-4k

Following the **General Procedure D**, **S-4k** (508 mg, >99%) as a white powder was obtained from TAG-OH (329 mg, 0.360 mmol). The analytically pure material (^{1}H NMR) was obtained by the General Procedure.

R*f*-value: 0.74 (hexane/EtOAc = 3:1, stained with phosphomolybdic acid)

HRMS (m/z): FAB $[M+Na]^+$ calculated for $C_{89}H_{143}NO_8Na$: 1377.0706, found: 1377.0695.

$[\alpha]_D^{24}$: –5.60 (c = 0.10, $CHCl_3$)

^{1}H NMR (500 MHz, $CDCl_3$): δ 7.76 (d, *J* = 7.4 Hz, 2H), 7.56 (d, *J* = 7.4 Hz, 2H), 7.40 (t, *J* = 7.4 Hz, 2H), 7.30 (t, *J* = 7.4 Hz, 2H), 6.86 (d, *J* = 8.6 Hz, 4H), 6.52 (s, 2H), 5.04 (dd, *J* = 11.8, 8.0 Hz, 2H), 4.67 (m, 1H), 4.41 (m, 1H), 4.34 (m, 1H), 4.21 (d, *J* = 7.4 Hz, 1H), 3.94 (m, 6H), 3.06 (m, 2H), 1.81 – 1.72 (m, 6H), 1.46 (m, 6H), 1.30 (s, 9H), 1.28 (br, 85H), 0.86 (t, *J* = 6.9 Hz, 9H).

^{13}C NMR (125 MHz, $CDCl_3$): δ 171.5, 155.7, 154.6, 153.4, 144.0, 143.8, 141.4, 138.5, 130.3, 130.1, 130.0, 127.9, 127.2, 125.2, 124.3, 120.1, 78.5, 73.6, 69.3, 67.8, 67.1, 54.9, 47.3, 37.7, 32.1, 30.5, 29.9, 29.8, 29.6, 29.6, 29.5, 29.0, 26.3, 22.8, 14.3.

• Fmoc-L-4-F-Phe-OTAG (**S-4l**)

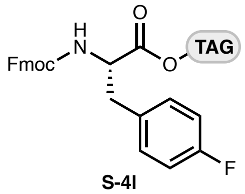

S-4l

Following the **General Procedure D**, **S-4l** (475 mg, >99%) as a white powder was obtained from TAG-OH (331 mg, 0.362 mmol). The analytically pure material (^{1}H NMR) was obtained by the General Procedure.

R*f*-value: 0.72 (hexane/EtOAc = 3:1, stained with phosphomolybdic acid)

HRMS (m/z): FAB [M+Na]$^+$ calculated for $C_{85}H_{134}NO_7FNa$: 1323.0037, found: 1323.0063.

[α]$_D$^{21}: –3.84 (c = 0.10, $CHCl_3$)

^{1}H NMR (500 MHz, $CDCl_3$): δ 7.77 (d, *J* = 7.4 Hz, 2H), 7.56 (d, *J* = 7.4 Hz, 2H), 7.40 (t, *J* = 7.4 Hz, 2H), 7.30 (t, *J* = 7.4 Hz, 2H), 6.87 (d, *J* = 6.9 Hz, 4H), 6.51 (s, 2H), 5.09 (d, *J* = 12.6 Hz, 1H), 4.99 (d, *J* = 12.6 Hz, 1H), 4.66 (m, 1H), 4.46 (m, 1H), 4.37 (m, 1H), 4.20 (m, 1H), 3.94 (m, 6H), 3.06 (m, 2H), 1.76 (m, 6H), 1.45 (m, 6H), 1.25 (br, 85H), 0.88 (t, *J* = 6.9 Hz, 9H).

^{13}C NMR (125 MHz, $CDCl_3$): δ 171.3, 163.1, 161.1, 155.6, 153.4, 143.9, 143.8, 141.5, 138.6, 131.1, 131.0, 129.9, 127.9, 127.2, 125.2, 125.1, 120.2, 115.6, 115.4, 73.6, 69.3, 68.0, 67.0, 54.8, 47.3, 37.5, 32.1, 30.5, 29.9, 29.8, 29.6, 29.5, 26.3, 22.8, 14.3.

• Fmoc-L-Trp(Boc)-OTAG (**S-4m**)

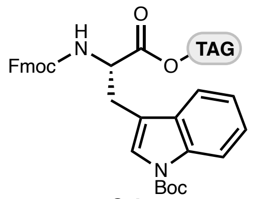

S-4m

Following the **General Procedure D**, **S-4m** (548 mg, >99%) as a white powder was obtained from TAG-OH (315 mg, 0.345 mmol). The analytically pure material (^{1}H NMR) was obtained by the General Procedure.

R*f*-value: 0.73 (hexane/EtOAc = 3:1, stained with phosphomolybdic acid)

HRMS (m/z): FAB [M+Na]$^+$ calculated for $C_{92}H_{144}N_2O_9Na$: 1444.0764, found: 1444.0781.

[α]$_D$^{22}: +3.94 (c = 0.10, $CHCl_3$)

^{1}H NMR (500 MHz, $CDCl_3$): δ 8.11 (s, 1H), 7.75 (d, J = 7.4 Hz, 2H), 7.55 – 7.26 (m, 8H), 7.20 (t, J = 7.4 Hz, 2H), 6.46 (s, 2H), 5.07 (d, J = 12.0 Hz, 1H), 4.94 (d, J = 12.0 Hz, 1H), 4.79 (dd, J = 13.7, 5.7 Hz, 1H), 4.37 – 4.32 (m, 2H), 4.20 (t, J = 7.2 Hz, 1H), 3.91 (m, 6H), 3.27 (dd, J = 7.2 Hz, 2H), 1.78 – 1.71 (m, 6H), 1.64 (s, 9H), 1.44 (br, 6H), 1.25 (br, 85H), 0.88 (t, J = 6.9 Hz, 9H).

^{13}C NMR (125 MHz, $CDCl_3$): δ 171.7, 155.8, 153.4, 149.7, 143.9, 143.8, 141.4, 138.5, 130.6, 129.9, 127.8, 127.2, 125.3, 124.7, 124.4, 122.8, 120.1, 118.9, 115.5, 114.9, 107.1, 73.5, 69.3, 68.1, 67.4, 54.3, 47.2, 32.1, 30.5, 29.9, 29.8, 29.6, 29.6, 29.5, 28.3, 28.1, 26.3, 22.8, 14.3.

• H-D-Val-OTAG (**18a**)

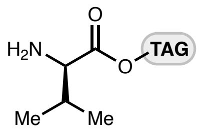

18a

Following the **General Procedure E**, **18a** (113 mg, >99%) as a white powder was obtained from **S-4a** (138 mg, 0.112 mmol). The analytically pure material (^{1}H NMR) was obtained by the General Procedure.

R*f*-value: 0.22 (hexane/EtOAc = 3:1, stained with phosphomolybdic acid)

HRMS (m/z): ESI $[M+Na]^+$ calculated for $C_{66}H_{125}NO_5Na$: 1034.9450, found: 1034.9471.

$[\alpha]_D^{23}$: –2.82 (c = 0.10, $CHCl_3$)

^{1}H NMR (500 MHz, $CDCl_3$): δ 6.53 (s, 2H), 5.09 (d, J = 12.0 Hz, 1H), 5.03 (d, J = 12.0 Hz, 1H), 3.97 – 3.91 (m, 6H), 3.41 – 3.37 (br m, 1H), 2.12 – 2.02 (br m, 1H), 1.82 – 1.69 (m, 6H), 1.50 – 1.41 (m, 6H), 1.38 – 1.20 (m, 84H), 0.99 – 0.85 (m, 15H).

^{13}C NMR (125 MHz, $CDCl_3$): δ 175.5, 153.2, 138.3, 130.7, 107.1, 73.4, 69.2, 66.8, 60.0, 32.2, 31.9, 30.5, 29.7, 29.7, 29.4, 29.4, 29.4, 29.3, 26.1, 22.7, 19.3, 17.1, 14.1.

• H-L-Leu-OTAG (**18b**)

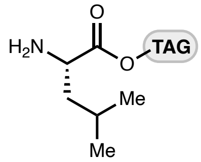

18b

Following the **General Procedure E**, **18b** (432 mg, >99%) as a white powder was obtained from **S-4b** (470 mg, 0.363 mmol). The characterization data of **18b** have been reported previously (*Angew. Chem. Int. Ed.* **2025**, *64*, e202416770.).

.

• H-*N*-Me-L-Leu-OTAG (**18c**)

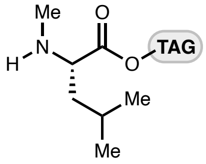

18c

Following the **General Procedure E**, **18c** (319 mg, 91%) as a white powder was obtained from **S-4c** (428 mg, 0.329 mmol). The analytically pure material (^{1}H NMR) was obtained by the General Procedure.

R*f*-value: 0.29 (hexane/EtOAc = 6:1, stained with phosphomolybdic acid)

HRMS (m/z): ESI $[M+Na]^+$ calculated for $C_{68}H_{129}NO_5Na$: 1062.9763, found: 1062.9757.

$[\alpha]_D^{21}$: –3.32 (c = 0.10, $CHCl_3$)

^{1}H NMR (500 MHz, $CDCl_3$): δ 6.53 (s, 2H), 5.07 (s, 2H), 3.94 (m, 6H), 3.24 (t, *J* = 7.2 Hz, 1H), 2.36 (s, 3H), 1.82 – 1.65 (m, 8H), 1.52 – 1.43 (m, 6H), 1.25 (br m, 86H), 0.92 – 0.86 (m, 15H).

^{13}C NMR (125 MHz, $CDCl_3$): δ 175.3, 153.3, 138.3, 130.9, 106.9, 73.5, 69.2, 66.8, 61.8, 53.6, 42.5, 34.6, 32.1, 30.5, 29.7, 26.2, 25.1, 22.8, 22.6, 14.2.

• H-L-Ile-OTAG (**18d**)

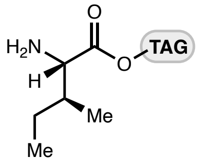

18d

Following the **General Procedure E**, **18d** (179 mg, >99%) as a white powder was obtained from **S-4d** (206 mg, 0.165 mmol). The analytically pure material (^{1}H NMR) was obtained by the General Procedure.

R*f*-value: 0.53 (hexane/EtOAc = 3:1, stained with phosphomolybdic acid)

HRMS (m/z): ESI $[M+Na]^+$ calculated for $C_{67}H_{127}NO_5Na$: 1048.9606, found: 1048.9624.

$[\alpha]_D^{23}$: –6.74 (c = 0.10, $CHCl_3$)

^{1}H NMR (500 MHz, $CDCl_3$): δ 6.53 (s, 2H), 5.18 – 5.07 (m, 2H), 5.02 (d, J = 12.0 Hz, 1H), 3.97 – 3.92 (m, 6H), 1.95 (br s, 1H), 1.81 – 1.70 (m, 6H), 1.45 (m, 6H), 1.25 (br, 86H), 0.98 (m, 2H), 0.90 – 0.84 (m, 15H).

^{13}C NMR (125 MHz, $CDCl_3$): δ 175.2, 153.3, 138.4, 130.8, 107.2, 73.5, 69.3, 67.0, 59.1, 39.2, 32.1, 30.5, 26.2, 24.8, 22.8, 15.8, 14.3, 11.8.

• H-L-Thr(^{t}Bu)-OTAG (**18e**)

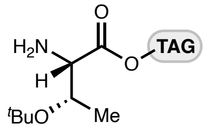

18e

Following the **General Procedure E**, **18e** (418 mg, 96%) as a white powder was obtained from **S-4e** (524 mg, 0.406 mmol). The analytically pure material (^{1}H NMR) was obtained by the General Procedure.

R*f*-value: 0.43 (hexane/EtOAc = 1:1, stained with phosphomolybdic acid)

HRMS (m/z): FAB $[M+Na]^+$ calculated for $C_{69}H_{131}NO_6Na$: 1092.9869, found: 1092.9885.

$[\alpha]_D^{22}$: –9.98 (c = 0.10, $CHCl_3$)

^{1}H NMR (500 MHz, $CDCl_3$): δ 6.55 (s, 2H), 5.09 (d, J = 12.0 Hz, 1H), 4.95 (d, J = 12.0 Hz, 1H), 4.00 (qd, J = 6.3 Hz, 3.4 Hz, 1H), 3.97 – 3.92 (m, 6H), 3.31 (d, J = 3.4 Hz, 1H), 1.82 – 1.70 (m, 6H), 1.46 – 1.43 (m, 6H), 1.25 (br, 86H), 1.11 (s, 9H), 0.88 – 0.83 (m, 12H).

^{13}C NMR (125 MHz, $CDCl_3$): δ 174.6, 153.3, 138.4, 130.6, 107.4, 77.5, 77.3, 77.0, 73.7, 73.5, 69.2, 68.6, 67.3, 60.8, 32.1, 30.5, 29.9, 29.9, 29.8, 29.8, 29.6, 29.6, 28.6, 26.3, 26.3, 22.9, 20.9, 14.3.

• H-L-Met-OTAG (**18f**)

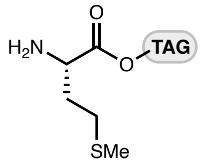

18f

Following the **General Procedure E**, **18f** (424 mg, >99%) as a white powder was obtained from **S-4f** (433 mg, 0.342 mmol). The analytically pure material (^{1}H NMR) was obtained by the General Procedure.

R*f*-value: 0.48 (hexane/EtOAc = 1:1, stained with phosphomolybdic acid)

HRMS (m/z): ESI $[M+Na]^+$ calculated for $C_{66}H_{125}NO_5SNa$: 1066.9171, found: 1066.9177.

$[\alpha]_D^{22}$: –7.94 (c = 0.10, $CHCl_3$)

^{1}H NMR (500 MHz, $CDCl_3$): δ 6.54 (s, 2H), 5.19 – 5.08 (m, 2H), 4.28 (m, 1H), 3.97 – 3.92 (m, 6H), 2.74 – 2.57 (m, 2H), 2.38 – 2.35 (m, 1H), 2.06 (s, 3H), 1.80 – 1.71 (m, 6H), 1.48 – 1.43 (m, 6H), 1.25 (br, 87H), 0.88 (t, J = 6.9 Hz, 9H).

^{13}C NMR (125 MHz, $CDCl_3$): δ 174.4, 153.4, 138.4, 130.4, 107.1, 73.6, 69.3, 67.6, 53.3, 32.1, 30.5, 30.3, 29.9, 29.8, 29.6, 29.5, 26.3, 22.8, 15.5, 14.3.

• H-L-Asp(^{t}Bu)-OTAG (**18g**)

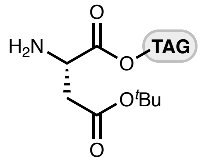

18g

Following the **General Procedure E**, **18g** (115 mg, >99%) as a white powder was obtained from **S-4g** (138 mg, 0.106 mmol). The analytically pure material (^{1}H NMR) was obtained by the General Procedure.

R*f*-value: 0.17 (hexane/EtOAc = 3:1, stained with phosphomolybdic acid)

HRMS (m/z): ESI $[M+Na]^+$ calculated for $C_{69}H_{129}NO_7Na$: 1106.9661, found: 1106.9658.

$[\alpha]_D^{23}$: –14.6 (c = 0.10, $CHCl_3$)

^{1}H NMR (500 MHz, $CDCl_3$): δ 6.54 (s, 2H), 5.07 – 4.99 (m, 2H), 3.97 – 3.91 (m, 6H), 3.85 – 3.90 (m, 1H), 3.00 – 2.83 (m, 2H), 1.82 – 1.70 (m, 6H), 1.49 – 1.43 (br, 15H), 1.36 – 1.25 (br, 86H), 0.88 (t, *J* = 7.2 Hz, 9H).

^{13}C NMR (125 MHz, $CDCl_3$): δ 173.2, 171.3, 153.3, 138.3, 130.6, 107.1, 81.9, 73.5, 69.2, 67.1, 51.8, 39.0, 32.1, 30.5, 29.7, 28.0, 26.2, 22.8, 14.3.

• H-L-Orn(Boc)-OTAG (**18h**)

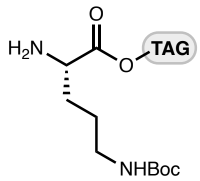

18h

Following the **General Procedure E**, **18h** (144 mg, >99%) as a white powder was obtained from **S-4h** (162 mg, 0.120 mmol). The analytically pure material (^{1}H NMR) was obtained by the General Procedure.

R*f*-value: 0.40 (hexane/EtOAc = 2:3, stained with phosphomolybdic acid)

HRMS (m/z): ESI $[M+H]^+$ calculated for $C_{71}H_{135}N_2O_7$: 1128.0264, found: 1128.0274.

$[\alpha]_D^{23}$: –5.88 (c = 0.10, $CHCl_3$)

^{1}H NMR (500 MHz, $CDCl_3$): δ 6.53 (s, 2H), 5.07 (d, *J* = 12.0 Hz, 1H), 5.02 (d, *J* = 12.0Hz, 1H), 4.67 (br, 1H), 3.97 – 3.92 (m, 6H), 3.56 (br, 1H), 3.13 (br, 1H), 1.84 – 1.70 (m, 8H), 1.49 – 1.42 (m, 15H), 1.25 (br, 89H), 0.88 (t, *J* = 6.9 Hz, 9H).

^{13}C NMR (125 MHz, $CDCl_3$): δ 158.1, 155.4, 140.5, 132.4, 109.3, 81.3, 75.6, 71.3, 69.5, 56.1, 42.4, 34.1, 32.5, 31.8, 31.6, 30.5, 28.3, 24.8, 16.3.

• H-L-His(Boc)-OTAG (**18i**)

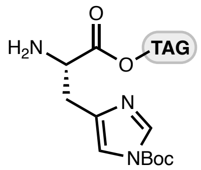

18i

Following the **General Procedure E**, **18i** (489 mg, >99%) as a white powder was obtained from **S-4i** (483 mg, 0.352 mmol). The analytically pure material (^{1}H NMR) was obtained by the General Procedure.

R*f*-value: 0.10 (hexane/EtOAc = 1:1, stained with phosphomolybdic acid)

HRMS (m/z): ESI $[M+H]^+$ calculated for $C_{72}H_{132}N_3O_7$: 1151.0060, found: 1151.0056.

$[\alpha]_D^{23}$: –11.3 (c = 0.10, $CHCl_3$)

^{1}H NMR (500 MHz, $CDCl_3$): δ 7.96 (s, 1H), 7.16 (s, 1H), 6.53 (s, 2H), 5.06 (s, 2H), 4.00 (br, 1H), 3.96 – 3.93 (m, 6H), 3.14 (dd, *J* = 14.9, 4.6 Hz, 1H), 2.92 (dd, *J* = 14.9, 4,6 Hz, 1H), 1.81 – 1.71 (m, 6H), 1.60 (s, 9H), 1.45 (br, 6H), 1.25 (br, 86H), 0.87 (t, *J* = 6.9 Hz, 9H).

^{13}C NMR (125 MHz, $CDCl_3$): δ 177.5, 174.8, 153.3, 147.0, 139.5, 138.3, 137.0, 130.6, 114.8, 107.1, 85.6, 73.5, 69.2, 67.4, 54.1, 33.0, 32.1, 30.5, 28.0, 26.2, 22.8, 14.2.

• H-*N*-Me-L-Phe-OTAG (**18j**)

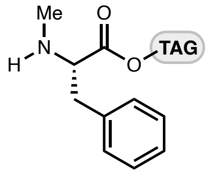

18j

Following the **General Procedure E**, **18j** (432 mg, >99%) as a white powder was obtained from **S-4j** (470 mg, 0.363 mmol). The analytically pure material (^{1}H NMR) was obtained by the General Procedure.

R*f*-value: 0.40 (hexane/EtOAc = 3:1, stained with phosphomolybdic acid)

HRMS (m/z): FAB $[M+Na]^+$ calculated for $C_{71}H_{127}NO_5Na$: 1096.9606, found: 1096.9629.

$[\alpha]_D^{23}$: –7.10 (c = 0.10, $CHCl_3$)

^{1}**H NMR** (500 MHz, $CDCl_3$): δ 7.32 – 7.28 (m, 0.5H, *Ph), 7.25 –7.10 (m, 4.5H, *Ph), 6.51 (s, 0.5H), 6.45 (s, 1.5H), 5.15 – 4.94 (m, 2H), 3.97 – 3.88 (m, 6H), 3.72 – 3.62 (br, 0.8H, *β-proton), 3.42 – 3.37 (m, 0.2H, *β-proton), 3.24 – 3.10 (br, 1H, *β-proton), 2.92 (s, 0.7H, **N*-Me), 2.58 – 2.47 (br, 2.30H, **N*-Me), 1.82 – 1.68 (m, 6H), 1.50 – 1.41 (m, 6H), 1.37 – 1.16 (m, 84H), 0.88 (t, J = 7.0 Hz, 9H).

^{13}**C NMR** (125 MHz, $CDCl_3$): δ 174.3, 153.4, 138.5, 137.1, 130.7, 129.4, 128.6, 126.9, 107.4, 73.6, 69.3, 67.1, 64.8, 39.7, 34.9, 32.2, 30.6, 30.0, 29.9, 29.7, 29.6, 26.4, 22.9, 14.4.

• H-L-Tyr(^{t}Bu)-OTAG (**18k**)

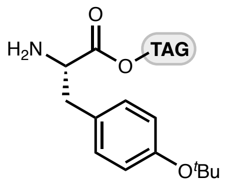

18k

Following the **General Procedure E**, **18k** (423 mg, 87%) as a white powder was obtained from **S-4k** (485 mg, 0.428 mmol). The analytically pure material (^{1}H NMR) was obtained by the General Procedure.

R*f*-value: 0.19 (hexane/EtOAc = 3:1, stained with phosphomolybdic acid)

HRMS (m/z): FAB $[M+Na]^+$ calculated for $C_{74}H_{133}NO_6Na$: 1155.0025, found: 1155.0005.

$[\alpha]_D^{23}$: –9.90 (c = 0.10, $CHCl_3$)

^{1}**H NMR** (500 MHz, $CDCl_3$): δ 7.02 (m, 2H), 6.88 (m, 2H), 6.53 (s, 2H), 5.16 – 4.99 (m, 2H), 4.38 (br, 1H), 3.98 – 3.94 (m, 6H), 3.38 (m, 2H), 1.79 – 1.73 (m, 6H), 1.47 (br, 6H), 1.29 (s, 9H), 1.25 (br, 86H), 0.88 (t, J = 6.9 Hz, 9H).

^{13}**C NMR** (125 MHz, $CDCl_3$): δ 174.9, 154.3, 153.3, 138.3, 131.7, 130.5, 129.8, 124.3, 107.2, 78.3, 73.5, 69.2, 67.2, 55.9, 40.4, 32.0, 30.4, 29.8, 29.8, 29.7, 29.5, 29.5, 28.9, 26.2, 22.8, 14.2.

• H-L-4-F-Phe-OTAG (**18l**)

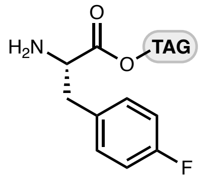

18l

Following the **General Procedure E**, **18l** (397 mg, >99%) as a white powder was obtained from **S-4l** (453 mg, 0.348 mmol). The analytically pure material (^{1}H NMR) was obtained by the General Procedure.

R*f*-value: 0.33 (hexane/EtOAc = 1:1, stained with phosphomolybdic acid)

HRMS (m/z): FAB [M+Na]$^+$ calculated for $C_{70}H_{124}NO_5FNa$: 1100.9356, found: 1100.9363.

[α]$_D$^{22}: –5.16 (c = 0.10, $CHCl_3$)

^{1}H NMR (500 MHz, $CDCl_3$): δ 7.06 (m, 2H), 6.93 (m, 2H), 6.50 (s, 2H), 5.00 (s, 2H), 3.94 (m, 6H), 3.7 (m, 1H), 3.03 (m, 1H), 2.87 (m, 1H), 1.79 – 1.73 (m, 6H), 1.46 (br, 6H), 1.25 (br, 86H), 0.87 (m, 9H).

^{13}C NMR (125 MHz, $CDCl_3$): δ 174.7, 163.0, 161.0, 153.4, 138.5, 132.5, 131.0, 130.3, 115.6, 115.4, 107.5, 73.6, 69.3, 67.5, 55.8, 39.9, 32.2, 32.1, 30.5, 30.0, 29.9, 29.6, 29.5, 26.4, 26.3, 23.0, 22.8, 14.4, 14.3.

• H-L-Trp(Boc)-OTAG (**18m**)

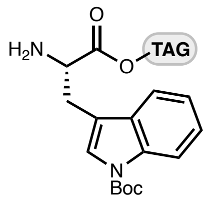

18m

Following the **General Procedure E**, **18m** (518 mg, >99%) as a white powder was obtained from **S-4m** (521 mg, 0.386 mmol). The analytically pure material (^{1}H NMR) was obtained by the General Procedure.

R*f*-value: 0.23 (hexane/EtOAc = 3:1, stained with phosphomolybdic acid)

HRMS (m/z): FAB [M+Na]$^+$ calculated for $C_{77}H_{134}N_2O_7Na$: 1222.0083, found: 1222.0034.

[α]$_D$^{22}: +4.08 (c = 0.10, $CHCl_3$)

^{1}H NMR (500 MHz, $CDCl_3$): δ 8.09 (br, 1H), 7.60 (br, 1H), 7.52 (app d, J = 7.5Hz, 1H), 7.31 – 7.18 (m, 2H), 6.47 (s, 2H), 5.04 – 4.95 (d, J = 12.0 Hz, 2H), 4.07 (br, 1H), 3.95 – 3.91 (m, 6H), 3.34 – 3.15 (m, 2H), 1.81 – 1.70 (m, 6H), 1.63 (s, 9H), 1.45 (m, 6H), 1.25 (br, 86H), 0.88 (t, J = 6.9Hz, 9H).

^{13}C NMR (125 MHz, $CDCl_3$): δ 174.9, 153.3, 149.6, 138.3, 135.6, 130.4, 124.6, 124.3, 122.6, 119.0, 115.9, 115.4, 107.1, 83.6, 73.5, 69.2, 67.4, 54.6, 32.0, 30.6, 30.4, 29.8, 29.8, 29.5, 29.5, 28.2, 26.2, 22.8, 14.2.

• BnO-tropolone-D-Val-OTAG (**19a**)

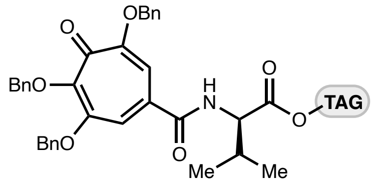

19a

Following the **General Procedure F**, **19a** (68.0 mg, 65%) as a white powder was obtained from **18a** (73.0 mg, 72.1 μmol). The analytically pure material (^{1}H NMR) was obtained by the General Procedure.

R*f*-value: 0.39 (hexane/EtOAc/AcOH = 3:1:0.1, stained with phosphomolybdic acid)

$[\alpha]_D^{22}$: +1.12 (c = 1.0, $CHCl_3$)

^{1}H NMR (500 MHz, $CDCl_3$): δ 7.48 (m, 4H), 7.38 – 7.26 (m, 11H), 7.24 (s, 1H), 7.15 (s, 1H), 6.55 (s, 2H), 6.23 (br s, 1H), 5.23 – 5.05 (m, 8H), 4.69 (br s, 1H), 3.96 – 3.92 (m, 6H), 2.23 (br, 1H), 1.81 – 1.70 (m, 6H), 1.46 (m, 6H), 1.25 (br, 84H), 0.92 – 0.85 (m, 15H).

^{13}C NMR (125 MHz, $CDCl_3$): δ 174.5, 171.7, 168.4, 162.5, 157.5, 154.8, 153.4, 138.5, 137.2, 136.9, 136.3, 135.3, 133.0, 130.2, 128.9, 128.9, 128.8, 128.4, 128.1, 127.6, 121.4, 110.6, 107.2, 73.7, 71.2, 69.3, 67.7, 58.1, 32.0, 31.4, 30.4, 29.8, 29.5, 26.2, 22.8, 19.1, 18.0, 14.2.

Note: HRMS using any routine conditions including Fast Atom Bombardment (FAB) could not be conducted due to its physicochemical property.

• BnO-tropolone-L-Leu-OTAG (**19b**)

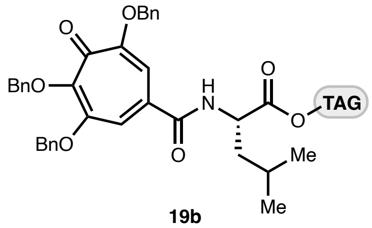

19b

Following the **General Procedure F**, **19b** (45.2 mg, 71%) as a white powder was obtained from **18b** (44.1 mg, 43.0 μmol). The analytically pure material (^{1}H NMR) was obtained by the General Procedure.

R*f*-value: 0.32 (hexane/EtOAc/AcOH = 3:1:0.1, stained with phosphomolybdic acid)

HRMS (m/z): FAB $[M+Na]^+$ calculated for $C_{96}H_{149}NO_{10}Na$: 1499.1074, found: 1499.1074.

$[\alpha]_D^{22}$: –5.39 (c = 0.10, $CHCl_3$)

^{1}H NMR (500 MHz, $CDCl_3$): δ 7.49 – 7.45 (m, 3H), 7.38 – 7.30 (m, 12H), 7.24 (s, 1H), 7.15 (s, 1H), 6.54 (s, 2H), 6.12 (br s, 1H), 5.24 (d, *J* = 10.9 Hz, 4H), 5.12 (d, *J* = 10.9 Hz, 3H), 5.16 (d, *J* = 10.9 Hz, 1H), 4.72 (m, 1H), 3.96 – 3.92 (m, 6H), 1.81 – 1.68 (m, 8H), 1.57 (m, 1H), 1.46 – 1.42, (m, 6H), 1.25 (br, 84H), 0.94 (d, *J* = 6.3 Hz, 6H), 0.88 (t, *J* = 6.9 Hz, 9H).

^{13}C NMR (125 MHz, $CDCl_3$): δ 174.4, 172.6, 168.4, 162.5, 157.7, 154.5, 153.4, 138.4, 137.2, 136.2, 135.3, 133.2, 130.3, 128.9, 128.8, 128.7, 128.4, 128.1, 127.6, 121.1, 110.7, 106.9, 73.7, 71.1, 69.3, 67.6, 52.0, 41.2, 32.0, 30.5, 29.8, 29.8, 29.5, 26.2, 25.1, 22.9, 22.1, 14.2.

• BnO-tropolone-*N*-Me-L-Leu-OTAG (**19c**)

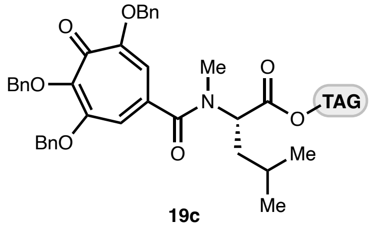

19c

Following the **General Procedure F**, **19c** (52.0 mg, 78%) as a white powder was obtained from **18c** (46.8 mg, 45.0 μmol). The analytically pure material (^{1}H NMR) was obtained by the General Procedure.

R*f*-value: 0.41 (hexane/EtOAc/AcOH = 3:1:0.1, stained with phosphomolybdic acid)

HRMS (m/z): FAB $[M+Na]^+$ calculated for $C_{97}H_{151}NO_{10}Na$: 1513.1230, found: 1513.1230.

$[\alpha]_D^{21}$: –3.28 (c = 1.0, $CHCl_3$)

^{1}H NMR (500 MHz, $CDCl_3$): δ 7.50 – 7.27 (m, 15H), 7.02 – 6.96 (br m, 0.3H), 6.79 (s, 1H), 6.56 (s, 0.7H), 6.52 – 6.43 (br m, 2H), 5.26 – 4.95 (m, 9H), 3.97 – 3.83 (m, 6H), 2.92 (s, 1H, **N*-Me), 2.43 (s, 2H, **N*-Me), 1.82 – 1.68 (m, 8H), 1.50 – 1.38 (m, 7H), 1.37 – 1.10 (m, 84H), 1.02 – 0.78 (m, 15H).

^{13}C NMR (125 MHz, $CDCl_3$): δ 163.1, 158.2, 153.5, 129.0, 128.8, 128.5, 128.1, 127.5, 196.9, 74.0, 73.7, 69.4, 67.6, 32.2, 26.4, 22.9, 14.4.

• BnO-tropolone-L-Ile-OTAG (**19d**)

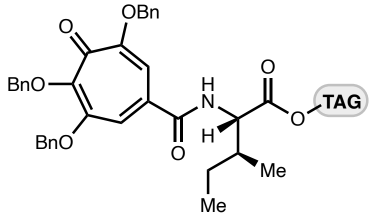

19d

Following the **General Procedure F**, **19d** (51.7 mg, 82%) as a white powder was obtained from **18d** (44.0 mg, 42.9 μmol). The analytically pure material (^{1}H NMR) was obtained by the General Procedure.

R*f*-value: 0.43 (hexane/EtOAc/AcOH = 3:1:0.1, stained with phosphomolybdic acid)

HRMS (m/z): FAB $[M+Na]^+$ calculated for $C_{96}H_{149}NO_{10}Na$: 1499.1074, found: 1499.1086.

$[\alpha]_D^{22}$: –2.78 (c = 0.45, $CHCl_3$)

^{1}H NMR (500 MHz, $CDCl_3$): δ 7.51 – 7.27 (m, 15H), 7.23 (s, 1H), 7.14 (s, 1H), 6.55 – 6.52 (m, 2H), 6.30 – 6.22 (br m, 1H), 5.27 – 5.03 (m, 8H), 4.71 (br, 1H), 3.96 – 3.92 (m, 6H), 1.94 (app br s, 1H) 1.81 – 1.69 (m, 6H), 1.50 – 1.40 (m, 8H), 1.40 – 1.07 (m, 84H), 0.92 – 0.83 (m, 15H).

^{13}C NMR (125 MHz, $CDCl_3$): δ 174.5, 171.7, 168.1, 162.5, 157.4, 155.0, 153.4, 138.6, 137.2, 136.3, 135.3, 132.8, 130.2, 129.0, 128.9, 128.8, 128.4, 128.1, 127.6, 121.6, 110.6, 107.2, 73.9, 73.6, 71.3, 69.3, 67.8, 57.5, 38.2, 32.1, 30.5, 29.9, 29.5, 26.3, 25.4, 22.8, 15.7, 14.3, 11.8.

• BnO-tropolone-L-Thr(^{t}Bu)-OTAG (**19e**)

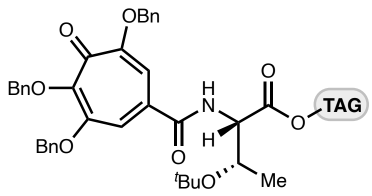

19e

Following the **General Procedure F**, **19e** (141 mg, 98%) as a white powder was obtained from **18e** (101 mg, 94.5 μmol). The analytically pure material (^{1}H NMR) was obtained by the General Procedure.

R*f*-value: 0.63 (hexane/EtOAc = 1:1, stained with phosphomolybdic acid)

HRMS (m/z): FAB $[M+Na]^+$ calculated for $C_{98}H_{153}NO_{11}Na$: 1543.1336, found: 1543.1335.

$[\alpha]_D^{23}$: +11.5 (c = 0.075, $CHCl_3$)

^{1}H NMR (500 MHz, $CDCl_3$): δ 7.48 – 2.30 (m, 15H), 6.54 (s, 2H), 5.23 – 5.13 (m, 6H), 5.09 (d, J = 12.0 Hz, 1H), 5.00 (d, J = 12.0 Hz, 1H), 4.64 (d, J = 8.0 Hz, 1H), 4.28 (d, J = 8.0, 6.3 Hz, 1H), 3.94 (m, 6H), 1.81 – 1.70 (m, 6H), 1.45 (br, 6H), 1.25 (br, 87H), 1.17 (d, J = 6.3 Hz, 3H), 1.10 (s, 9H), 0.88 (t, J = 6.9 Hz, 9H).

^{13}C NMR (125 MHz, $CDCl_3$): δ 174.5, 170.4, 168.7, 162.6, 157.4, 155.0, 153.4, 138.7, 137.2, 136.2, 135.3, 132.9, 129.9, 128.9, 128.8, 128.7, 128.4, 128.4, 128.2, 128.1, 127.7, 121.5, 110.6, 107.6, 74.4, 74.0, 73.8, 73.5, 71.2, 69.3, 68.1, 67.5, 58.8, 32.0, 30.4, 29.8, 29.8, 29.5, 29.5, 28.5, 26.2, 22.8, 21.4, 14.2.

• BnO-tropolone-L-Met-OTAG (**19f**)

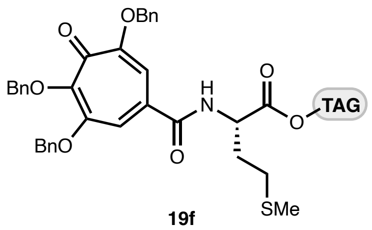

19f

Following the **General Procedure F**, **19f** (210 mg, >99%) as a white powder was obtained from **18f** (140 mg, 0.135 mmol). The analytically pure material (^{1}H NMR) was obtained by the General Procedure.

R*f*-value: 0.75 (hexane/EtOAc = 2:1, stained with phosphomolybdic acid)

HRMS (m/z): FAB $[M+Na]^+$ calculated for $C_{95}H_{147}NO_{10}SNa$: 1517.0638, found: 1517.0657.

[α]D²²: –3.62 (c = 0.10, $CHCl_3$)

^{1}H NMR (500 MHz, $CDCl_3$): δ 7.49 – 7.27 (m, 15H), 7.25 – 7.20 (m, 2H), 6.85 (app br s, 1H, *NH), 6.57 – 6.51 (br m, 2H), 5.31 – 5.13 (m, 8H), 4.83 (app br s, 1H), 3.97 – 3.88 (m, 6H), 2.51 – 2.44 (br m, 2H), 2.27 – 2.19 (br m, 1H), 2,12 – 2.00 (m, 4H), 1.82 – 1.69 (m, 6H), 1.49 – 1.39 (m, 6H), 1.39 – 1.14 (m, 84H), 0.89 – 0.82 (m, 9H).

^{13}C NMR (125 MHz, $CDCl_3$): δ 174.4, 171.6, 168.2, 162.5, 157.7, 153.4, 138.6, 137.1, 136.1, 135.3, 128.9, 128.7, 128.4, 128.4, 128.2, 128.1, 127.7, 110.6, 107.1, 73.7, 71.2, 69.3, 68.0, 52.9, 32.0, 26.3, 22.8, 15.6, 14.2.

• BnO-tropolone-L-Asp(^{t}Bu)-OTAG (**19g**)

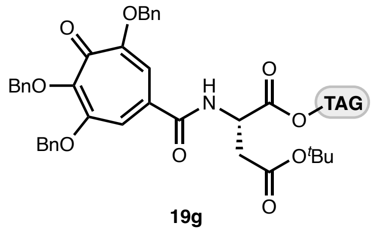

19g

Following the **General Procedure F**, **19g** (60.7 mg, 95%) as a white powder was obtained from **18g** (45.4 mg, 41.9 μmol). The analytically pure material (^{1}H NMR) was obtained by the General Procedure.

R*f*-value: 0.55 (hexane/EtOAc = 2:1, stained with phosphomolybdic acid)

HRMS (m/z): FAB $[M+Na]^+$ calculated for $C_{98}H_{151}NO_{12}Na$: 1557.1129, found: 1557.1140.

[α]D²⁵: +11.0 (c = 0.10, $CHCl_3$)

^{1}H NMR (500 MHz, $CDCl_3$): δ 7.50 – 7.46 (m, 4H), 7.39 – 7.27 (m, 12H), 7.17 (s, 1H), 6.93 (d, *J* = 7.5 Hz, 1H), 6.53 (s, 2H), 5.25 (s, 2H), 5.22 (s, 2H), 5.14 (s, 2H), 5.09 (d, *J* = 12.0 Hz, 1H), 4.95 (d, *J* = 12.0 Hz, 1H), 4.82 – 4.77 (m, 1H), 3.96 – 3.88 (m, 6H), 3.06 (dd, *J* = 17.5, 4.5 Hz, 1H), 2.92 (dd, *J* = 17.5, 4.5 Hz, 1H), 1.80 – 1.67 (m, 6H), 1.48 – 1.37 (m, 15H), 1.37 – 1.17 (m, 84H), 0.88 (t, *J* = 6.8 Hz, 9H).

^{13}C NMR (125 MHz, $CDCl_3$): δ 174.5, 171.0, 169.3, 167.8, 162.5, 157.5, 154.9, 153.4, 138.5, 137.2, 136.2, 135.3, 132.6, 130.2, 128.9, 128.9, 128.8, 128.5, 128.4, 128.4, 128.2, 128.2, 127.7, 121.5, 110.4, 107.2, 83.1, 74.0, 73.8, 73.5, 71.2, 69.2, 67.4, 50.1, 36.4, 32.0, 30.5, 29.8, 29.8, 29.6, 29.5, 27.9, 26.2, 22.8, 14.2.

• BnO-tropolone-L-Orn(Boc)-OTAG (**19h**)

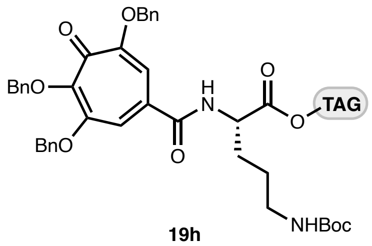

19h

Following the **General Procedure F**, **19h** (55.4 mg, 92%) as a white powder was obtained from **18h** (42.9 mg, 38.1 μmol). The analytically pure material (^{1}H NMR) was obtained by the General Procedure.

R*f*-value: 0.45 (hexane/EtOAc = 4:3, stained with phosphomolybdic acid)

HRMS (m/z): FAB $[M+Na]^+$ calculated for $C_{100}H_{156}N_2O_{12}Na$: 1600.1550, found: 1600.1547.

$[\alpha]_D^{22}$: –0.54 (c = 0.10, $CHCl_3$)

^{1}H NMR (500 MHz, $CDCl_3$): δ 7.47 – 7.29 (m, 16H), 7.19 (s, 1H), 6.88 (br s, 1H), 6.54 (s, 2H), 5.23 (d, *J* = 14.3 Hz, 3H), 5.11 (d, *J* = 14.3 Hz, 3H), 4.72 – 4.67 (m, 2H), 4.60 (br, 1H), 3.96 – 3.91 (m, 6H), 3.14 – 3.06 (m, 2H), 1.93 (m, 2H), 1.81 – 1.70 (m, 8H), 1.45 (br, 6H), 1.39 (s, 9H), 1.25 (br, 85H), 0.88 (t, *J* = 6.9 Hz, 9H).

^{13}C NMR (125 MHz, $CDCl_3$): δ 174.3, 171.9, 168.9, 162.6, 158.3, 156.4, 154.0, 153.4, 138.5, 137.1, 136.0, 135.2, 130.3, 128.8, 128.6, 128.5, 128.3, 128.1, 127.8, 110.5, 107.1, 79.2, 73.7, 73.5, 73.0, 71.2, 69.3, 67.7, 53.5, 40.0, 32.0, 30.5, 29.8, 29.8, 29.6, 29.5, 28.5, 26.4, 26.2, 22.8, 14.2.

• BnO-tropolone-L-His(Boc)-OTAG (**19i**)

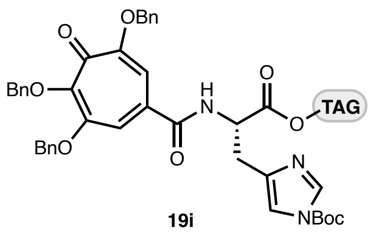

19i

Following the **General Procedure F**, **19i** (210 mg, 92%) as a white powder was obtained from **18i** (163 mg, 0.142 mmol). The analytically pure material (^{1}H NMR) was obtained by the General Procedure.

R*f*-value: 0.59 (hexane/EtOAc = 1:1, stained with phosphomolybdic acid)

HRMS (m/z): FAB $[M+Na]^+$ calculated for $C_{101}H_{153}N_3O_{12}Na$: 1623.1346, found: 1623.1318.

[α]$_D$^{22}: +27.1 (c = 0.10, $CHCl_3$)

^{1}H NMR (500 MHz, $CDCl_3$): δ 7.64 – 7.60 (m, 1H), 7,52 – 7.46 (m, 3H), 7.41 – 7.27 (m, 15H), 6.53 (br, 2H), 5.29 –5.16 (m, 6H), 5.08 – 4.94 (m, 2H), 4.06 – 4.00 (br, 1H), 3.99 – 3.89 (m, 6H), 3.38 – 3.14 (br, 2H), 1.80 – 1.70 (m, 6H), 1.61 (s, 9H), 1.49 – 1.40 (m, 6H), 1.38 – 1.18 (m, 84H), 0.90 – 0.83 (m, 9H).

^{13}C NMR (125 MHz, $CDCl_3$): δ 174.4, 170.7, 167.6, 162.6, 157.7, 154.7, 153.3, 146.5, 138.5, 138.4, 137.2, 136.9, 136.2, 135.4, 132.6, 130.2, 128.8, 128.7, 128.6, 128.3, 128.2, 128.0, 127.7, 121.2, 114.9, 110.4, 106.9, 86.0, 73.9, 73.5, 73.4, 71.2, 69.2, 67.7, 53.2, 32.0, 30.4, 29.8, 29.7, 29.5, 29.4, 29.0, 27.8, 26.2, 22.7, 14.2.

• BnO-tropolone-*N*-Me-L-Phe-OTAG (**19j**)

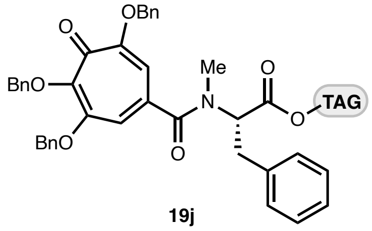

19j

Following the **General Procedure F**, **19j** (80.7 mg, 53%) as a white powder was obtained from **18j** (107 mg, 0.100 mmol). The analytically pure material (^{1}H NMR) was obtained by the General Procedure.

R*f*-value: 0.37 (hexane/EtOAc/AcOH = 3:1:0.1, stained with phosphomolybdic acid)

HRMS (m/z): FAB $[M+Na]^+$ calculated for $C_{100}H_{149}NO_{10}Na$: 1547.1074, found: 1547.1079.

[α]$_D$^{23}: –7.48 (c = 0.10, $CHCl_3$)

^{1}H NMR (500 MHz, $CDCl_3$): δ 7.46 – 7.13 (overlap m, 21H), 6.96 – 6.88 (br, 1H), 6.56 – 6.46 (m, 2H), 5.38 – 5.33 (m, 0.5H, *Bn × 4 + α-proton), 5.18 – 4.72 (m, 8.5H, *Bn × 4 + α-proton), 3.96 – 3.83 (m, 6H), 3.55 – 3.48 (m, 1H), 3.17 – 3.12 (m, 1H), 3.02 (s, 1H, **N*–Me), 2.44 (s, 2H, **N*–Me), 1.80 – 1.67 (m, 6H), 1.49 – 1.38 (m, 6H), 1.38 – 1.10 (m, 84H), 0.88 (t, J = 6.8 Hz, 9H).

^{13}C NMR (125 MHz, $CDCl_3$): δ 174.2, 171.8, 170.0, 163.1, 158.0, 153.5, 138.6, 137.3, 136.7, 136.1, 135.2, 134.4, 128.9, 128.8, 128.7, 128.4, 128.3, 128.1, 127.7, 120.2, 110.6, 107.1, 73.9, 73.6, 71.2, 69.4, 67.8, 58.1, 34.9, 34.5, 32.0, 30.5, 29.8, 29.8, 29.6, 29.6, 29.5, 26.3, 22.8, 14.2, 1.1.

• BnO-tropolone-L-Tyr(^{t}Bu)-OTAG (**19k**)

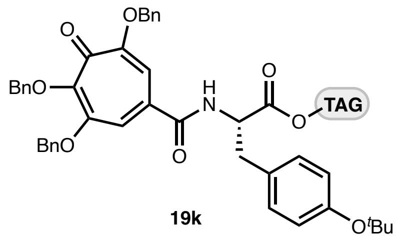

19k

Following the **General Procedure F**, **19k** (139 mg, >99%) as a white powder was obtained from **18k** (92.9 mg, 82.1 μmol). The analytically pure material (^{1}H NMR) was obtained by the General Procedure.

R*f*-value: 0.34 (hexane/EtOAc = 3:1, stained with phosphomolybdic acid)

HRMS (m/z): FAB $[M+Na]^+$ calculated for $C_{103}H_{155}NO_{11}Na$: 1605.1492, found: 1605.1500.

$[\alpha]_D^{23}$: +6.16 (c = 0.10, $CHCl_3$)

^{1}H NMR (500 MHz, $CDCl_3$): δ 7.48 – 7.26 (m, 15H), 7.14 (s, 1H), 7.06 (s, 1H), 6.84 (d, *J* = 8.6 Hz, 4H), 6.57 (s, 2H), 6.23 (m, 1H), 5.20 – 5.14 (m, 6H), 5.06 (s, 2H), 4.95 (dd, *J* = 14.0, 6.0 Hz, 1H), 3.95 (m, 6H), 3.20 (dd, *J* = 14.0, 6.0 Hz, 1H), 3.09 (dd, *J* = 14.3, 5.2 Hz, 1H), 1.82 – 1.17 (m, 6H), 1.45 (m, 6H), 1.28 (s, 9H), 1.25 (br, 84H), 0.88 (t, *J* = 6.9 Hz, 9H).

^{13}C NMR (125 MHz, $CDCl_3$): δ 174.4, 171.3, 168.1, 162.6, 157.7, 154.9, 154.6, 153.5, 138.7, 137.3, 136.1, 135.3, 130.5, 130.1, 130.0, 128.9, 128.9, 128.8, 128.5, 128.5, 128.4, 128.2, 127.8, 124.3, 120.9, 110.5, 107.6, 78.5, 74.0, 73.6, 73.5, 71.2, 69.4, 68.0, 54.4, 37.0, 32.1, 30.6, 29.9, 29.9, 29.7, 29.6, 29.0, 26.3, 22.9, 14.3.

• BnO-tropolone-L-4-F-Phe-OTAG (**19l**)

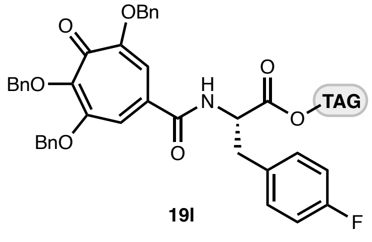

19l

Following the **General Procedure F**, **19l** (39.3 mg, >99%) as a white powder was obtained from **19l** (26.5 mg, 24.6 μmol). The analytically pure material (^{1}H NMR) was obtained by the General Procedure.

R*f*-value: 0.41 (hexane/EtOAc = 3:1, stained with phosphomolybdic acid)

HRMS (m/z): FAB $[M+Na]^+$ calculated for $C_{99}H_{146}NO_{10}FNa$: 1551.0823, found: 1551.0864.

$[\alpha]_D^{22}$: +3.48 (c = 0.10, $CHCl_3$)

^{1}H NMR (500 MHz, $CDCl_3$): δ 7.47 – 7.29 (m, 15H), 7.21 (d, *J* = 8.6 Hz, 2H), 6.97 (br s, 1H), 6.90 (d, *J* = 8.6 Hz, 2H), 6.56 (s, 2H), 5.24 – 5.17 (m, 6H), 5.12 – 5.02 (m, 2H), 4.99 – 4.92 (m, 1H), 3.98 – 3.93 (m, 6H), 3.25-3.14 (m, 2H), 1.82 – 1.72 (m, 6H), 1.47 – 1.43 (m, 6H), 1.25 (br, 84H), 0.88 (t, *J* = 6.9 Hz, 9H).

^{13}C NMR (125 MHz, $CDCl_3$): δ 174.3, 171.1, 167.9, 162.5, 157.6, 154.8, 153.4, 138.7, 137.1, 136.0, 135.2, 131.4, 131.1, 131.0, 129.8, 128.9, 128.9, 128.8, 128.6, 128.5, 128.4, 128.3, 128.1, 127.7, 121.4, 115.7, 115.5, 110.6, 107.7, 74.0, 73.8, 73.6, 71.3, 69.3, 68.2, 54.2, 36.8, 32.1, 30.5, 29.9, 29.8, 29.6, 29.5, 29.5, 26.3, 22.8, 14.3.

• BnO-tropolone-L-Trp(Boc)-OTAG (**19m**)

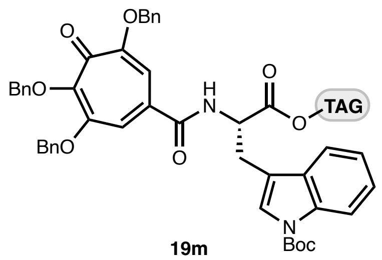

19m

Following the **General Procedure F**, **19m** (215 mg, 98%) as a white powder was obtained from **18m** (170 mg, 0.142 µmol). The analytically pure material (^{1}H NMR) was obtained by the General Procedure.

R*f*-value: 0.23 (hexane/EtOAc = 3:1, stained with phosphomolybdic acid)

HRMS (m/z): FAB $[M+Na]^+$ calculated for $C_{106}H_{156}N_2O_{12}Na$: 1672.1550, found: 1672.1550.

$[\alpha]_D^{22}$: +17.6 (c = 0.10, $CHCl_3$)

^{1}H NMR (500 MHz, $CDCl_3$): δ 8.08 (br s, 1H), 7.44 – 7.22 (m, 18H), 7.13 (t, *J* = 6.6 Hz, 1H), 7.08 (s, 1H), 6.98 (s, 1H), 6.53 (s, 2H), 6.33 (br m, 1H), 5.18 – 4.88 (m, 9H), 3.94 (m, 6H), 3.43 (d, *J* = 12,0 Hz, 1H), 3.28 (d, *J* = 12.0 Hz, 1H), 1.80 – 1.72 (m, 6H), 1.62 (s, 9H), 1.45 (br, 6H), 1.25 (br, 84H), 0.87 (t, *J* = 6.9 Hz, 9H).

^{13}C NMR (125 MHz, $CDCl_3$): δ 174.3, 171.4, 168.4, 162.6, 157.8, 154.5, 153.5, 149.6, 138.6, 137.3, 136.1, 135.3, 135.3, 133.1, 130.8, 129.9, 128.8, 128.6, 128.3, 128.1, 127.7, 124.8, 124.4, 122.9, 120.8, 118.9, 115.6, 115.3, 110.4, 107.1, 84.0, 73.8, 73.5, 73.2, 71.0, 69.3, 68.1, 54.1, 32.1, 30.5, 29.9, 29.8, 29.6, 29.5, 28.3, 27.0, 26.3, 22.8, 14.3.

• Tropolone-D-Val-OH (**20**)

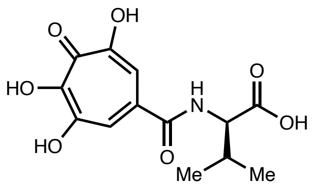

20

Following the **General Procedure G**, **20** (10.5 mg, >99%) as a brown oil was obtained from **19a** (46.7 mg, 31.9 μmol). The analytically pure material (^{1}H NMR) was obtained by the General Procedure.

HRMS (m/z): ESI $[M+H]^+$ calculated for $C_{13}H_{16}NO_7$: 298.0921, found: 298.095.

$[\alpha]_D^{23}$: –16.5 (c = 0.10, MeOH with 0.1% TFA)

^{1}H NMR (500 MHz, CD_3OD): δ 7.45 (s, 2H), 4.43 (d, *J* = 6.0 Hz, 1H), 2.31 – 2.24 (m 1H), 1.06 (d, *J* = 2.0 Hz, 3H), 1.04 (d, *J* = 6.9 Hz, 3H).

^{13}C NMR (125 MHz, CD_3OD): δ 174.7, 171.7, 159.4, 157.3, 134.8, 118.1, 60.4, 31.6, 19.7, 18.8.

• Tropolone-L-Leu-OH (**21**)

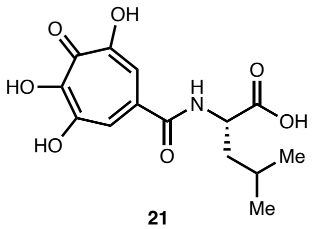

21

Following the **General Procedure G**, **21** (11.9 mg, 51%) as a brown oil was obtained from **19b** (108 mg, 73.2 μmol). The analytically pure material (^{1}H NMR) was obtained by the General Procedure.

HRMS (m/z): ESI $[M+H]^+$ calculated for $C_{14}H_{18}NO_7$: 312.1078, found: 312.1081.

$[\alpha]_D^{22}$: +4.24 (c = 0.10, MeOH with 0.1% TFA)

^{1}H NMR (500 MHz, CD_3OD): δ 7.46 (s, 2H), 4.59 (dd, *J* = 10.3, 10.3 Hz, 1H), 1.82 – 1.69 (m, 3H), 0.99 (d, *J* = 6.3 Hz, 3H), 0.97 (d, *J* = 6.3 Hz, 3H).

^{13}C NMR (125 MHz, CD_3OD): δ 175.9, 171.3, 159.4, 157.3, 134.5, 118.0, 53.1, 41.2, 26.3, 23.4, 21.7.

• Tropolone-*N*-Me-L-Leu-OH (**22**)

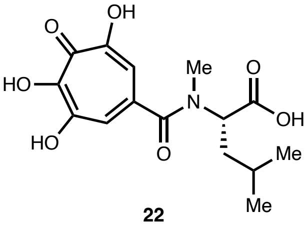

22

Following the **General Procedure G**, **21** (14.0 mg, 88%) as a brown oil was obtained from **19c** (73.1 mg, 49.1 μmol). The analytically pure material (^{1}H NMR) was obtained by the General Procedure.

HRMS (m/z): ESI $[M+H]^+$ calculated for $C_{15}H_{20}NO_7$: 326.1234, found: 326.1226.

$[\alpha]_D^{22}$: –21.9 (c = 0.10, MeOH with 0.1% TFA)

^{1}H NMR (500 MHz, CD_3OD): δ 6.99 – 6.94 (br, 2H), 5.21 – 5.16 (m, 0.6H, *α-proton), 4.28 – 4.23 (m, 0.4H, *α-proton), 2.99 – 2.97 (br, 1H, **N*-Me), 2.91 – 2.87 (br, 2H, **N*-Me), 1.95 – 1.72 (m, 2H), 1.68 – 1.48 (m, 1H), 1.03 (app dd, *J* = 10.0, 7.0 Hz, 4H), 0.90 (app d, *J* = 6.5 Hz, 1H), 0.75 (app d, *J* = 6.5 Hz, 1H).

^{13}C NMR (125 MHz, CD_3OD): δ 174.2, 173.6, 158.3, 158.2, 136.4, 116.8, 61.4, 56.4, 39.0, 38.0, 34.5, 29.8, 26.5, 25.8, 23.6, 23.3, 21.9, 21.6

• Tropolone-L-Ile-OH (**23**)

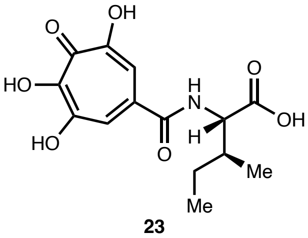

23

Following the **General Procedure G**, **23** (19.9 mg, 64%) as a brown oil was obtained from **19d** (123 mg, 83.3 μmol). The analytically pure material (^{1}H NMR) was obtained by the General Procedure.

HRMS (m/z): ESI $[M+H]^+$ calculated for $C_{14}H_{18}NO_7$: 312.1078, found: 312.1080.

$[\alpha]_D^{22}$: +20.0 (c = 0.10, MeOH with 0.1% TFA)

^{1}H NMR (500 MHz, CD_3OD): δ 7.44 (s, 2H), 4.49 (d, *J* = 6.0 Hz, 1H), 2.05 – 1.97 (m, 1H), 1.65 – 1.55 (m, 1H), 1.38 – 1.27 (m, 1H), 1.02 (d, *J* = 7.0 Hz, 3H), 0.97 (t, *J* = 7.5 Hz, 3H).

^{13}C NMR (125 MHz, CD_3OD): δ 174.8, 171.5, 159.4, 157.3, 134.8, 118.1, 59.4, 38.0, 26.6, 16.1, 11.7.

• Tropolone-L-Thr-OH (**24**)

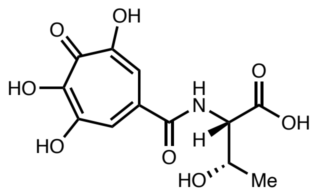

24

Following the **General Procedure G**, **24** (18.1 mg, 67%) as a brown oil was obtained from **19e** (138 mg, 90.8 μmol). The analytically pure material (^{1}H NMR) was obtained by the General Procedure.

HRMS (m/z): ESI $[M+H]^+$ calculated for $C_{12}H_{14}NO_8$: 300.0714, found: 300.0723.

$[\alpha]_D^{22}$: +48.8 (c = 0.10, MeOH with 0.1% TFA)

^{1}H NMR (500 MHz, CD_3OD): δ 7.54 (s, 2H), 4.58 (s, 1H), 4.42 – 4.37 (br, 1H), 1.26 (d, *J* = 6.5 Hz, 3H).

^{13}C NMR (125 MHz, CD_3OD): δ 173.6, 159.5, 157.3, 137.4, 134.3, 118.0, 68.5, 60.3, 30.8, 20.6.

• Tropolone-L-Met-OH (**25**)

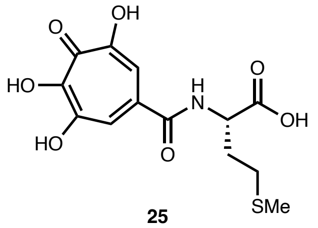

25

Following the **General Procedure G**, **25** (11.1 mg, 24%) as a brown oil was obtained from **19f** (210 mg, 0.141 mmol). The analytically pure material (^{1}H NMR) was obtained by the General Procedure.

HRMS (m/z): ESI $[M+H]^+$ calculated for $C_{13}H_{16}NO_7S$: 330.0642, found: 330.0645.

$[\alpha]_D^{22}$: +20.0 (c = 0.10, MeOH with 0.1% TFA)

^{1}H NMR (500 MHz, CD_3OD): δ 7.49 (s, 2H), 4.72 – 4.68 (m, 1H), 2.68 – 2.55 (m, 2H), 2.28 – 2.19 (m, 1H), 2.14 – 2.04 (m, 4H).

^{13}C NMR (125 MHz, CD_3OD): δ 175.1, 171.4, 159.4, 157.3, 134.4, 118.1, 53.7, 31.7, 31.6, 15.2.

• Tropolone-L-Asp-OH (**26**)

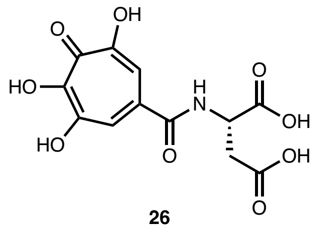

26

Following the **General Procedure G**, **26** (12.4 mg, >99%) as a brown oil was obtained from **19g** (60.7 mg, 39.6 μmol). The analytically pure material (^{1}H NMR) was obtained by the General Procedure.

HRMS (m/z): ESI $[M+H]^+$ calculated for $C_{12}H_{12}NO_9$: 314.0507, found: 314.0512.

$[\alpha]_D^{22}$: –13.7 (c = 0.10, MeOH with 0.1% TFA)

^{1}H NMR (500 MHz, CD_3OD): δ 7.47 (s, 2H), 2.99 (dd, J = 16.5, 5.0 Hz, 1H), 2.88 (dd, J = 16.5, 8.0 Hz, 1H).

^{13}C NMR (125 MHz, CD_3OD): δ 174.1, 173.9, 170.9, 157.3, 134.2, 118.0, 51.3, 36.6.

• Tropolone-L-Orn-OH (**27**)

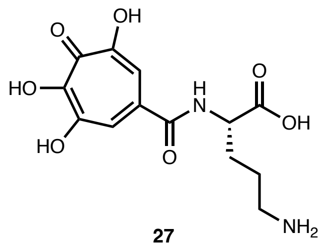

27

Following the **General Procedure G**, **27** (26.5 mg, >99%) as a brown oil was obtained from **19h** (125 mg, 79.3 μmol). The analytically pure material (^{1}H NMR) was obtained by the General Procedure.

HRMS (m/z): ESI $[M+H]^+$ calculated for $C_{13}H_{17}N_2O_7$: 313.1030, found: 313.1035.

$[\alpha]_D^{23}$: –19.6 (c = 0.10, MeOH with 0.1% TFA)

^{1}H NMR (500 MHz, CD_3OD): δ 7.51 (s, 2H), 4.60 (dd, J = 9.0, 4.5 Hz, 1H), 3.03 – 2.93 (m, 2H), 2.15 – 2.05 (m, 1H), 1.92 – 1.77 (m, 3H).

^{13}C NMR (125 MHz, CD_3OD): δ 174.7, 171.3, 163.1, 162.9, 159.5, 157.3, 134.1, 118.0, 54.0, 40.2, 29.4, 25.4

• Tropolone-L-His-OH (**28**)

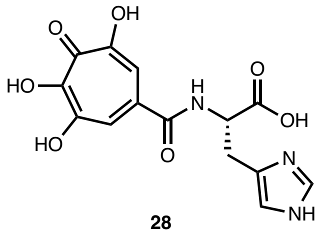

28

Following the **General Procedure G**, **28** (16.8 mg, 40%) as a brown oil was obtained from **19i** (204 mg, 0.127 mmol). The analytically pure material (^{1}H NMR) was obtained by the General Procedure.

HRMS (m/z): ESI $[M+H]^+$ calculated for $C_{14}H_{14}N_3O_7$: 336.0826, found: 336.0821.

$[\alpha]_D^{20}$: –50.3 (c = 0.10, MeOH with 0.1% TFA)

^{1}H NMR (500 MHz, CD_3OD): δ 8.83 (s, 1H), 7.40 (s, 2H), 7.36 (s, 1H), 4.94 (dd, *J* = 9.5, 5.0 Hz, 1H), 3.47 – 3.42 (m, 1H), 3.25 – 3.20 (m, 1H).

^{13}C NMR (125 MHz, CD_3OD): δ 173.1, 170.9, 159.6, 157.2, 135.1, 133.7, 131.5, 118.4, 117.9, 53.6, 27.7.

• Tropolone-*N*-Me-L-Phe-OH (**29**)

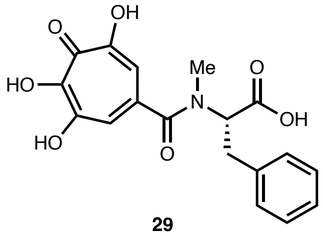

29

Following the **General Procedure G**, **29** (13.3 mg, >99%) as a brown oil was obtained from **19j** (49.3 mg, 32.3 μmol). The analytically pure material (^{1}H NMR) was obtained by the General Procedure.

HRMS (m/z): ESI $[M+H]^+$ calculated for $C_{18}H_{18}NO_7$: 360.1078, found: 360.1077.

$[\alpha]_D^{22}$: –69.0 (c = 0.10, MeOH with 0.1% TFA)

^{1}H NMR (500 MHz, CD_3OD): δ 7.39 – 7.27 (m, 5H), 7.07 – 7.03 (m, 1H), 6.58 (s, 1H), 5.26 (dd, *J* = 12.0, 5.0 Hz, 0.5 H, *α-proton), 4.54 (dd, *J* = 11.5, 4.0 Hz, 0.5 H, *α-proton), 3.49 – 3.42 (m, 0.5 H, *β-proton), 3.27 – 3.23 (m, 1.5 H, *β-proton), 3.05 (s, 1.5 H, **N*-Me), 2.74 (s, 1.5 H, **N*-Me).

^{13}C NMR (125 MHz, $(CD_3)_2CO$): δ 171.6, 171.4, 159.0, 158.6, 158.3, 158.0, 138.6, 137.9, 130.2, 129.9, 129.5, 129.4, 127.9, 127.7, 119.3, 117.0, 116.7, 114.7, 112.5, 79.2, 64.3, 59.6, 36.0, 35.2, 34.9.

• Tropolone-L-Tyr-OH (**30**)

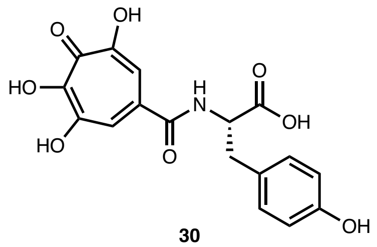

30

Following the **General Procedure G**, **30** (25.7 mg, 81%) as a brown oil was obtained from **19k** (135 mg, 85.3 µmol). The analytically pure material (^{1}H NMR) was obtained by the General Procedure.

HRMS (m/z): ESI $[M+Na]^+$ calculated for $C_{17}H_{15}NO_8Na$: 384.0690, found: 384.0689.

$[\alpha]_D^{21}$: –93.0 (c = 0.10, MeOH with 0.1% TFA)

^{1}H NMR (500 MHz, CD_3OD): δ 7.33 (s, 2H), 7.09 (d, *J* = 6.0 Hz, 2H), 6.72 (d, *J* = 6.0 Hz, 2H), 4.74 – 4.68 (m, 1H), 3.26 – 3.21 (m, 1H), 3.00 – 2.93 (m, 1H).

^{13}C NMR (125 MHz, CD_3OD): δ 174.7, 171.0, 159.4, 157.3, 157.2, 134.6, 131.2, 129.3, 118.0, 116.3, 56.3, 37.4.

• Tropolone-L-4-F-Phe-OH (**31**)

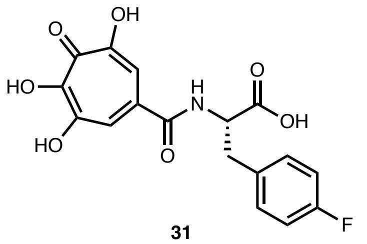

31

Following the **General Procedure G**, **31** (4.90 mg, >99%) as a brown oil was obtained from **19l** (16.9 mg, 11.1 µmol). The analytically pure material (^{1}H NMR) was obtained by the General Procedure.

HRMS (m/z): ESI $[M+H]^+$ calculated for $C_{17}H_{15}NO_7F$: 364.0827, found: 364.0850.

$[\alpha]_D^{27}$: +15.9 (c = 0.10, MeOH with 0.1% TFA)

^{1}H NMR (500 MHz, CD_3OD): δ 7.34 – 7.15 (br, 4H), 7.05 – 6.93 (br, 2H), 4.82 – 4.73 (br, 1H), 3.09 –3.00 (br, 1H, *β-proton).

Note: The remaining β-proton signal is likely overlapped with the solvent peak of CD_3OD (δ 3.30).

^{13}C NMR (125 MHz, CD_3OD): δ 174.7, 171.1, 164.3, 162.3, 157.3, 134.8, 132.0, 123.0, 117.8, 116.2, 116.0, 56.2, 37.4.

• Tropolone-L-Trp-OH (**32**)

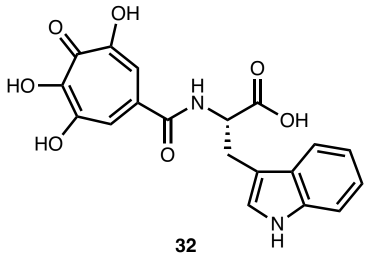

32

Following the **General Procedure G**, **32** (38.8 mg, 80%) as a brown oil was obtained from **19m** (210 mg, 0.127 mmol). The analytically pure material (^{1}H NMR) was obtained by the General Procedure.

HRMS (m/z): ESI [M+Na]$^+$ calculated for $C_{19}H_{16}N_2O_7Na$: 407.0850, found: 407.0859.

[α]$_D$^{22}: –63.4 (c = 0.10, MeOH with 0.1% TFA)

^{1}H NMR (500 MHz, $(CD_3)_2CO$ containing with 1% TFA): δ 8.16 (g, *J* = 8.0 Hz, 1H), 7.72 (d, *J* = 8.0 Hz, 1H), 7.66 (d, *J* = 6.5 Hz, 1H), 7.53 (d, *J* = 5.5 Hz, 1H), 7.38 – 7.23 (m, 2H), 7.10 – 6.98 (m, 1H), 5.04 – 4.91 (m, 1H), 3.54 – 3.46 (m, 1H), 3.37 (dd, *J* = 14.0, 8.5 Hz, 1H).

^{13}C NMR (125 MHz, $(CD_3)_2CO$ containing with 1% TFA): δ 173.7, 173.6, 137.8, 137.7, 128.7, 124.7, 124.5, 122.6, 120.0, 119.3, 118.9, 116.9, 114.6, 112.5, 112.4, 111.0, 55.1, 28.0.

3-3. Liquid-phase synthesis of the dipeptide derivative

• Fmoc-L-Phe-OTAG (**S-6**)

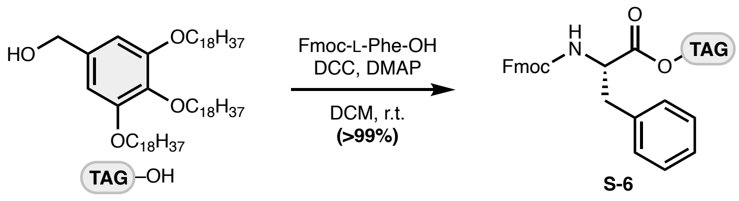

Following the **General Procedure D**, **S-6** (432 mg, >99%) as a white powder was obtained from TAG-OH (309 mg, 0.338 mmol). The analytically pure material (^{1}H NMR) was obtained by the General Procedure.

R*f*-value: 0.59 (hexane/EtOAc = 3:1, stained with phosphomolybdic acid)

HRMS (m/z): FAB $[M+Na]^+$ calculated for $C_{85}H_{135}NO_7Na$: 1305.0131, found: 1305.0140.

$[\alpha]_D^{23}$: +1.40 (c = 0.10, $CHCl_3$)

^{1}H NMR (500 MHz, $CDCl_3$): δ 7.77 (d, *J* = 7.5 Hz, 2H), 7.58 – 7.52 (m, 2H), 7.40 (t, *J* = 7.5 Hz, 2H), 7.30 (t, *J* = 7.5 Hz, 2H), 7.24 – 7.18 (m, 3H), 6.99 – 6.95 (br, 2H), 6.51 (s, 2H), 5.25 (d, *J* = 8.5 Hz, *NH), 5.09 (d, *J* = 12.0 Hz, 1H), 5.01 (d, *J* = 12.0 Hz, 1H), 4.74 – 4.68 (m, 1H, *α-proton), 4.44 (dd, *J* = 10.0, 6.5 Hz, 1H), 4.33 (dd, *J* = 10.0, 6.5 Hz, 1H), 4.20 (t, *J* = 6.5 Hz, 1H), 3.96 – 3.87 (m, 6H), 3.16 – 3.06 (m, 2H), 1.82 – 1.70 (m, 6H), 1.50 – 1.41 (m, 6H), 1.38 – 1.15 (m, 84H), 0.88 (t, *J* = 7.0 Hz, 9H).

^{13}C NMR (125 MHz, $CDCl_3$): δ 171.5, 155.6, 153.4, 144.0, 143.8, 141.4, 138.5, 135.6, 130.1, 129.6, 128.7, 127.9, 127.2, 125.3, 125.2, 120.1, 107.5, 73.6, 69.3, 67.9, 67.1, 54.8, 47.3, 38.3, 32.1, 30.5, 29.9, 29.8, 29.6, 29.6, 29.5, 26.3, 22.8, 14.3.

• H-L-Phe-OTAG (**S-7**)

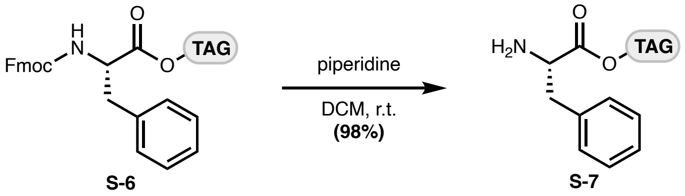

Following the **General Procedure E**, **S-7** (326 mg, 98%) as a white powder was obtained from **S-6** (402 mg, 0.314 mmol). The analytically pure material (^{1}H NMR) was obtained by the General Procedure.

R*f*-value: 0.27 (hexane/EtOAc = 1:1, stained with phosphomolybdic acid)

HRMS (m/z): FAB $[M+Na]^+$ calculated for $C_{70}H_{125}NO_5Na$: 1082.9450, found: 1082.9447.

$[\alpha]_D^{22}$: –4.74 (c = 0.10, $CHCl_3$)

^{1}H NMR (500 MHz, $CDCl_3$): δ 8.74 (br s, 2H, *NH_2), 7.24 – 7.16 (m, 3H), 7.16 – 7.09 (m, 2H), 6.54 – 6.47 (m, 2H), 5.19 – 4.95 (m, 2H), 4.43 – 4.36 (br m, 1H), 3.97 – 3.88 (m, 6H), 3.43 – 3.35 (br m, 2H), 1.82 – 1.71 (m, 6H), 1.51 – 1.41 (br, 6H), 1.41 – 1.10 (br, 84H), 0.88 (t, J = 6.8 Hz, 9H).

^{13}C NMR (125 MHz, $CDCl_3$): δ 174.8, 153.3, 138.4, 137.0, 130.5, 129.5, 128.6, 126.9, 107.3, 73.5, 69.2, 67.3, 55.9, 41.0, 32.1, 30.5, 29.9, 29.8, 29.6, 29.5, 26.3, 22.8, 14.3.

• Fmoc-*N*-Me-L-Leu-L-Phe-OTAG (**S-8**)

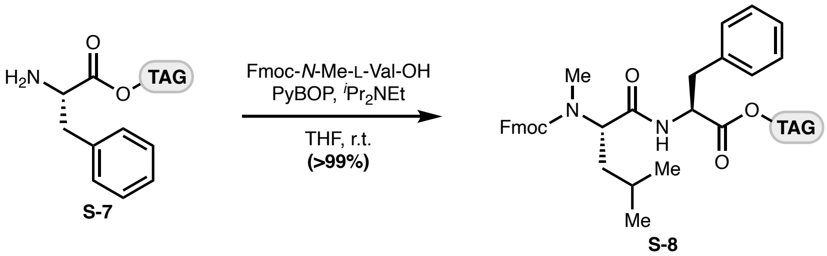

To a solution of Fmoc-*N*-Me-L-Val-OH (70.7 mg, 0.525 mmol, 3.0 equiv) in THF (3.50 mL, 50 mM) was added PyBOP (163 mg, 0.350 mmol, 2.0 equiv), $^i Pr_2NEt$ (0.183 mL, 1.05 mmol, 6.0 equiv), and **S-7** (185 mg, 0.175 mmol) at room temperature. After stirring for 2 h, the reaction mixture was cooled down to 0 °C and MeOH (17.5 mL, 4 mM) was added. The resulting suspension was stirred for 10 min at 0 °C before the solid was collected by vacuum filtration. The resulting solid residue was washed with excess MeOH, yielding **S-8** (263 mg, >99%) as a white powder.

R*f*-value: 0.54 (hexane/EtOAc = 3:1, stained with phosphomolybdic acid)

HRMS (m/z): FAB $[M+Na]^+$ calculated for $C_{92}H_{148}N_2O_8Na$: 1432.9450, found: 1432.1132.

$[\alpha]_D^{23}$: +22.5 (c = 0.10, $CHCl_3$)

^{1}H NMR (500 MHz, $CDCl_3$): δ 7.81 – 7.28 (m, 8H), 7.19 – 6.85 (m, 5H), 6.51 (br s, 2H), 6.40 (br d, *J* = 7.0 Hz, 1H, *NH) 5.11 – 4.99 (m, 2H), 4.88 – 4.65 (m, 2H), 4.53 – 4.47 (m, 1H), 4.43 – 4.15 (m, 2H), 3.94 – 3.91 (m, 6H), 3.18 – 3.12 (br m, 1H), 2.99 – 2.91 (br m, 1H), 2.60 – 2.51 (br m, 3H, **N*-Me), 1.81 – 1.70 (m, 8H), 1.49 – 1.40 (m, 7H), 1.39 – 1.21 (m, 84H), 0.91 – 0.84 (m, 15H).

^{13}C NMR (125 MHz, $CDCl_3$): δ 171.3, 170.6, 157.4, 153.4, 144.0, 143.9, 141.5, 138.5, 135.8, 130.2, 129.2, 128.6, 127.9, 127.2, 127.1, 125.2, 125.1, 120.2, 107.3, 73.6, 69.3, 67.9, 67.8, 56.7, 53.0, 47.4, 38.0, 36.3, 32.1, 30.5, 29.9, 29.8, 29.6, 29.6, 29.5, 26.3, 24.8, 23.2, 22.8, 22.0, 14.3.

• H-*N*-Me-L-Leu-L-Phe-OTAG (**S-9**)

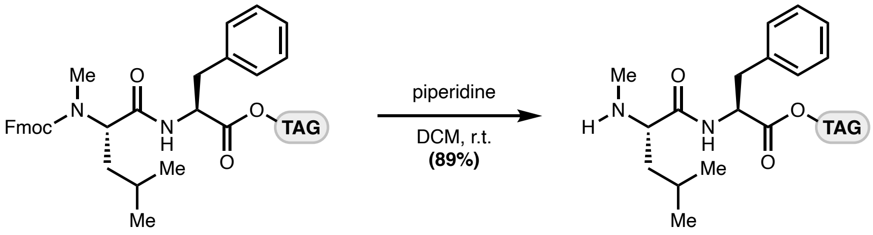

To a reaction vessel containing **S-8** (233 mg, 0.165 mmol) was added 10% piperidine/DCM (8.25 mL, 20 mM) at room temperature. After stirring for 1 h, the reaction mixture was cooled down to 0 °C and MeOH (41.3 mL, 4 mM) was added. The resulting suspension was stirred for 10 min at 0 °C before the solid was collected by vacuum filtration. The resulting solid residue was washed with excess MeOH, yielding **S-9** (174 mg, 89%) as a white powder.

***Rf*-value**: 0.50 (only EtOAc, stained with phosphomolybdic acid)

HRMS (m/z): FAB $[M+Na]^+$ calculated for $C_{77}H_{138}N_2O_6Na$: 1210.0447, found: 1210.0448.

$[\alpha]_D^{22}$: –6.60 (c = 0.10, $CHCl_3$)

^{1}H NMR (500 MHz, $CDCl_3$): δ δ 7.34 – 7.15 (m, 5H), 7.01 (d, *J* = 8.5 Hz, 1H, *NH), 6.53 – 6.47 (m, 2H), 5.08 – 5.02 (m, 2H), 4.91 – 4.78 (m, 1H), 3.97 – 3.90 (m, 6H), 3.72 (app br s, 1H), 3.37 – 3.33 (m, 1H), 3.09 – 3.02 (m, 1H), 2.59 – 2.52 (app br m, 3H, **N*-Me), 1.87 – 1.70 (m, 8H), 1.50 – 1.40 (m, 8H), 1.40 – 1.10 (m, 84H), 0.92 – 0.84 (m, 15H).

^{13}C NMR (125 MHz, $CDCl_3$): δ 171.5, 153.3, 138.4, 136.2, 130.2, 129.5, 128.6, 127.1, 73.6, 69.3, 67.8, 63.3, 52.9, 41.9, 38.0, 34.2, 32.1, 30.5, 29.9, 29.8, 29.6, 29.5, 26.3, 25.1, 22.8, 22.8, 22.4, 14.3.

• BnO-tropolone-*N*-Me-L-Leu-L-Phe-OTAG (**S-10**)

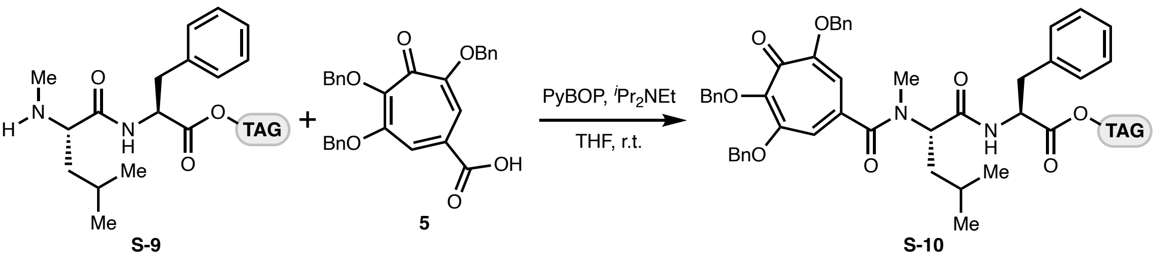

To a solution of **5** (12.1 mg, 25.8 µmol, 0.9 equiv) in THF (2.84 mL, 10 mM) was added PyBOP (26.8 mg, 56.8 µmol, 2.0 equiv), $^{i}Pr_2NEt$ (27.1 µL, 0.170 mmol, 6.0 equiv), and **S-9** (33.7 mg, 28.4 µmol) at room temperature. After stirring for 8 h, the reaction mixture was cooled down to 0 °C and MeOH (14.2 mL, 4 mM) was added. The resulting suspension was stirred for 10 min at 0 °C before the solid was collected by vacuum filtration. The resulting solid residue was washed with excess MeOH. The resulting residue was purified by preparative TLC (hexane/EtOAc = 1:1), yielding **S-10** (28.8 mg, 62%) as a yellow solid.

R*f*-value: 0.71 (hexane/EtOAc = 1:1, stained with phosphomolybdic acid)

HRMS (m/z): FAB $[M+Na]^+$ calculated for $C_{106}H_{160}N_2O_{11}Na$: 1660.1914, found: 1661.1927.

$[\alpha]_D^{21}$: –10.3 (c = 0.10, $CHCl_3$)

^{1}H NMR (500 MHz, $CDCl_3$): δ 7.51 – 7.28 (m, 16H), 7.09 (br s, 1H), 6.71 – 6.68 (m, 1H), 6.58 – 6.49 (m, 2H), 5.26 – 5.01 (m, 8H), 4.92 – 4.82 (m, 2H), 3.96 – 3.90 (m, 6H), 3.23 – 3.16 (br m, 1H), 3.00 – 2.92 (br m, 1H), 2.19 – 2.16 (br m, 3H, **N*-Me), 1.81 – 1.71 (m, 8H), 1.50 – 1.41 (m, 7H), 1.39 – 1.21 (m, 84H), 0.96 – 0.83 (m, 15H).

^{13}C NMR (125 MHz, $CDCl_3$): δ 174.6, 172.6, 171.4, 169.9, 157.9, 154.5, 153.5, 138.6, 137.3, 136.4, 135.5, 133.8, 129.3, 129.1, 128.9, 128.7, 128.6, 128.5, 128.4, 128.0, 127.3, 122.1, 111.7, 107.4, 74.0, 73.7, 71.4, 69.4, 68.0, 55.6, 53.3, 38.0, 36.1, 33.0, 32.2, 30.6, 30.0, 29.9, 29.7, 29.7, 29.6, 26.4, 25.2, 23.4, 22.9, 22.1.

• Tropolone-*N*-Me-L-Leu-L-Phe-OH (**33**)

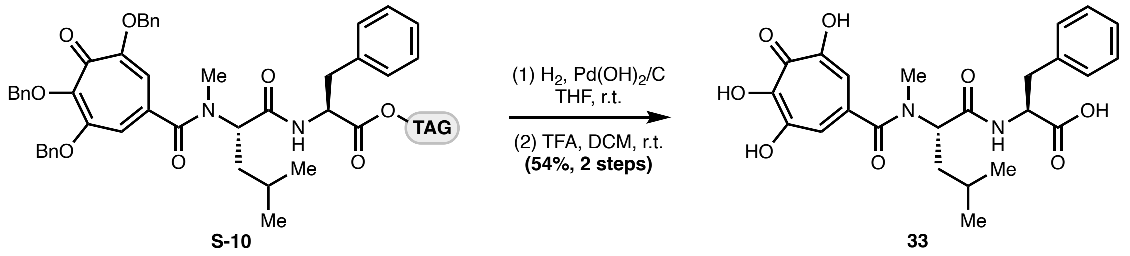

To a solution of **S-10** (19.1 mg, 11.7 μmo) in THF (1.17 mL, 10 mM) was added $Pd(OH)_2$/C (1.91 mg, 10 wt%) at room temperature under air. The resulting mixture was carefully evacuated by an aspirator and backfilled with H_2 × 3. After stirring for 8 h under a H_2 atmosphere, the reaction mixture was cooled down to 0 °C and MeOH (5.85 mL, 2 mM) was added. The resulting suspension was stirred for 10 min at 0 °C and centrifuged at 40 rpm for 7 min before the supernatant was removed by decantation. The resulting solid residue was washed with MeOH (2 mM) by repeating the centrifuge separation described above (a total of three washes). The resulting residue was dissolved in DCM (1.17 mL, 10 mM) before the resulting mixture was centrifuged at 40 rpm for 7 min. After removing the resulting sediment by filtration, the filtrate was concentrated in vacuo. This crude material was used in the next reaction without further purification.

To a reaction vessel containing the crude product was added 33% TFA/DCM (1.17 mL, 10 mM) at room temperature under air. After stirring for 3 h, the reaction mixture was diluted with DCM (2.34 mL, 5 mM) and concentrated in vacuo. To the resulting residue was added MeOH (2.34 mL, 5 mM) and the suspension was centrifuged at 40 rpm for 7 min before the sediment was removed by decantation and the supernatant was concentrated in vacuo. After repeating this cycle three times, the supernatant was filtered through a pad of Celite®. The filtrate was concentrated in vacuo and dried under high vacuum, yielding **33** (3.00 mg, 54%) as a brown oil.

HRMS (m/z): ESI $[M+Na]^+$ calculated for $C_{24}H_{28}N_2O_8Na$: 495.1738, found: 495.1751.

$[\alpha]_D^{22}$: –57.1 (c = 0.10, MeOH with 0.1% TFA)

^{1}H NMR (500 MHz, CD_3OD): δ 7.32 – 7.14 (m, 6H), 6.99 – 6.85 (br, 1H), 5.16 (t, *J* = 7.8 Hz, 0.8H, *α-proton), 4.77 – 4.60 (br, 1.20H, *α-proton), 3.07 – 2.88 (br, 2H, *β-proton of Phe), 2.50 (s, 3H, **N*-Me), 1.71 – 1.51 (m, 3H, *β & γ -proton of Leu), 1.01 – 0.83 (m, 6H).

^{13}C NMR (125 MHz, CD_3OD): δ 175.0, 173.9, 172.0, 164.4, 158.5, 138.7, 130.2, 129.6, 129.5, 127.9, 127.7, 117.0, 57.5, 56.2, 55.0, 38.3, 37.7, 33.5, 30.8, 26.2, 23.4, 22.3

4. NMR and LC-UV Spectra Charts

• BnO-tropolone-L-Pro-O^{t}Bu (**7b**): ^{1}H NMR (500 MHz, CD_3OD), ^{13}C NMR (125 MHz, CD_3OD)

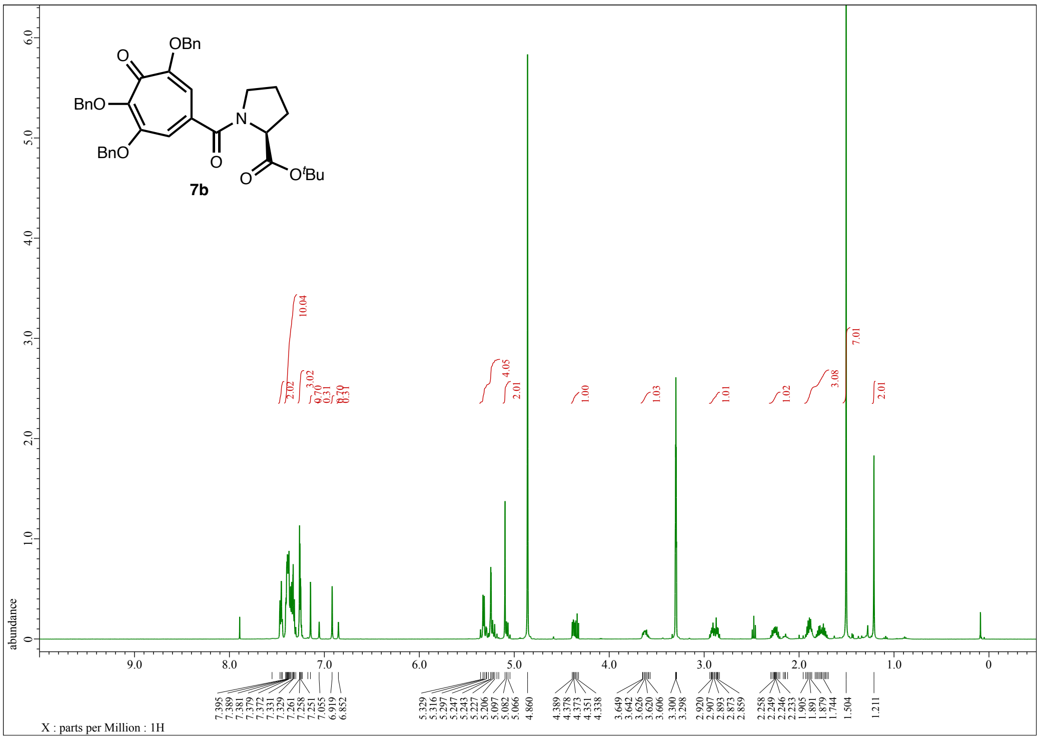

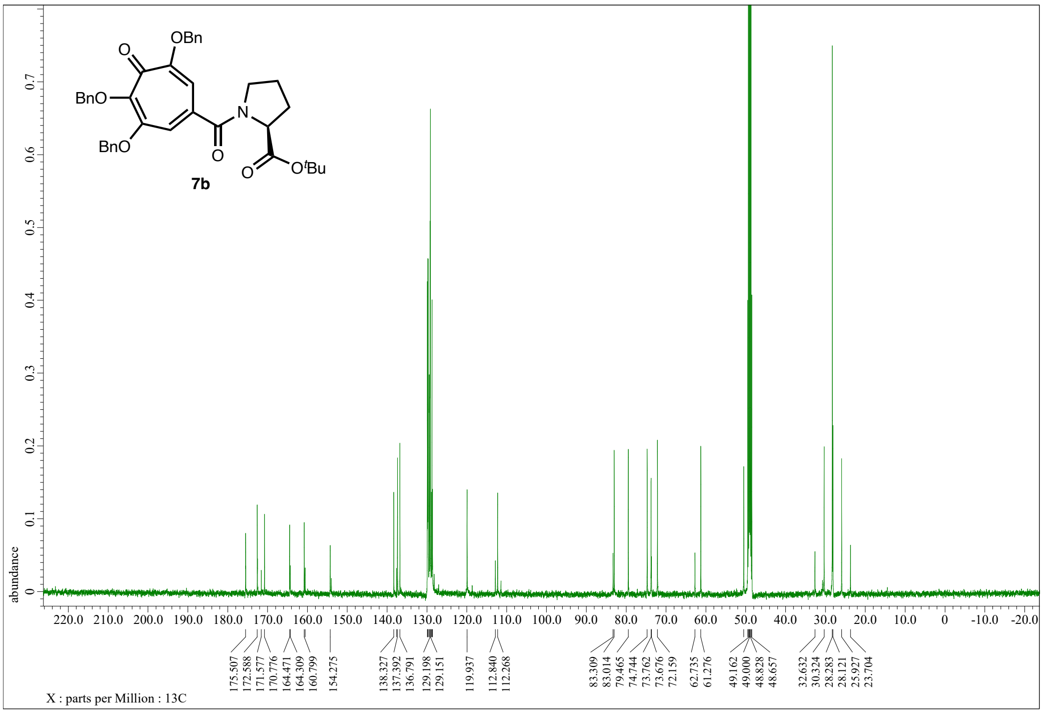

• BnO-tropolone-Gly-O^{t}Bu (**10**): ^{1}H NMR (500 MHz, CD_3OD), ^{13}C NMR (125 MHz, CD_3OD)

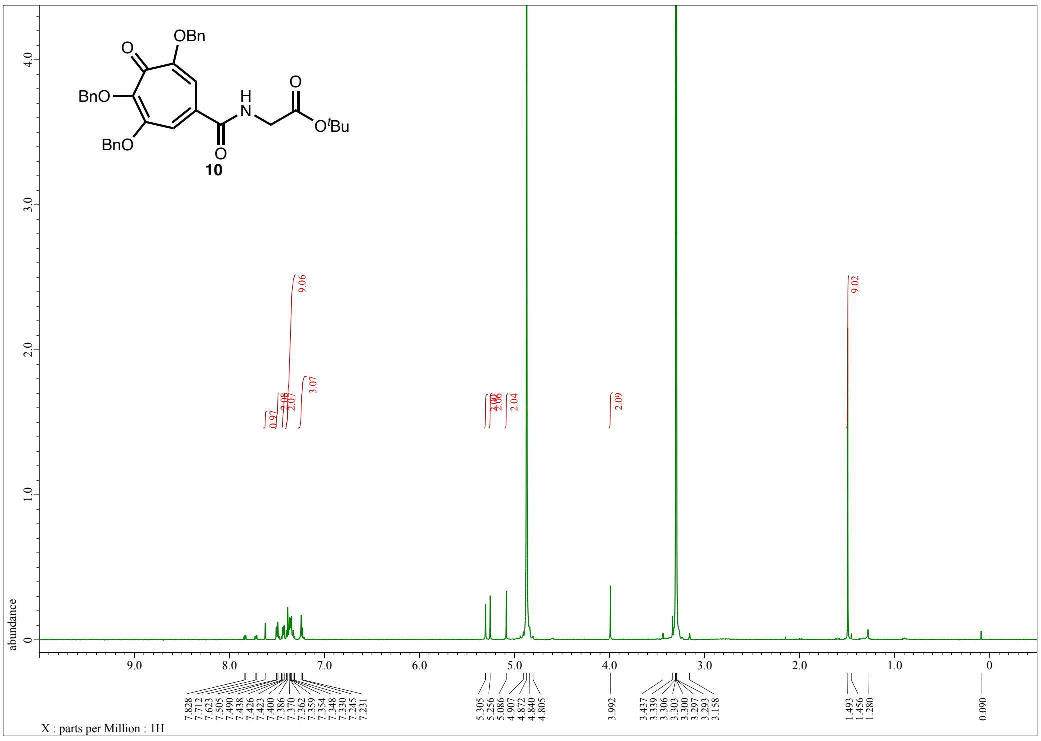

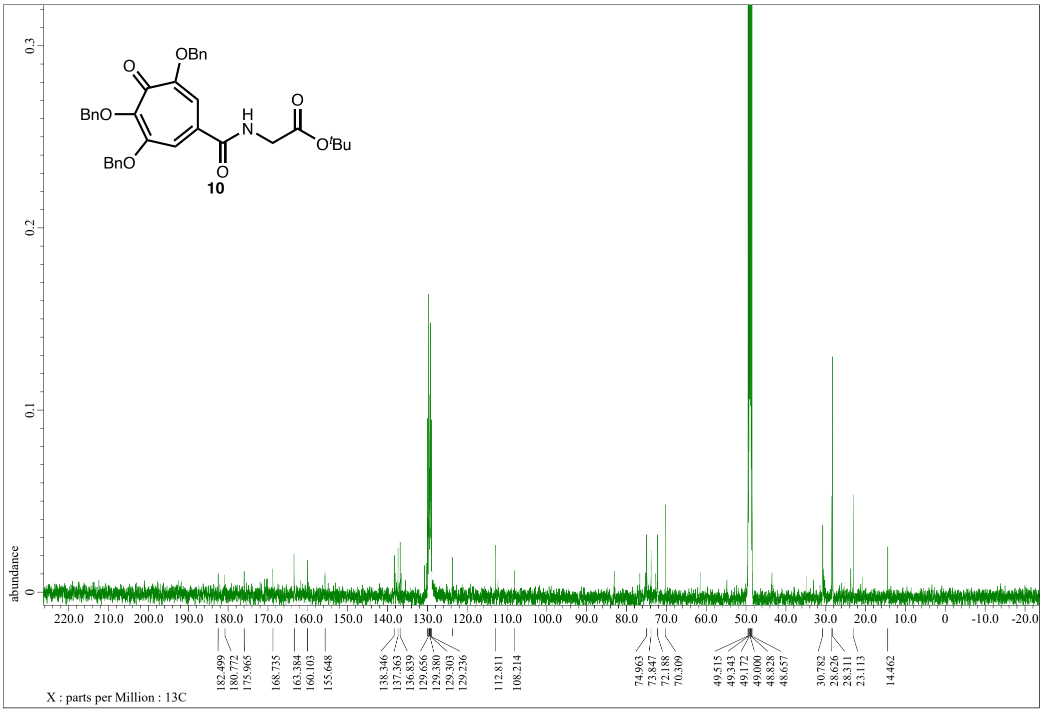

• BnO-tropolone-L-Phe-O^{t}Bu (**11**): ^{1}H NMR (500 MHz, $(CD_3)_2CO$), ^{13}C NMR (125 MHz, $(CD_3)_2CO$)

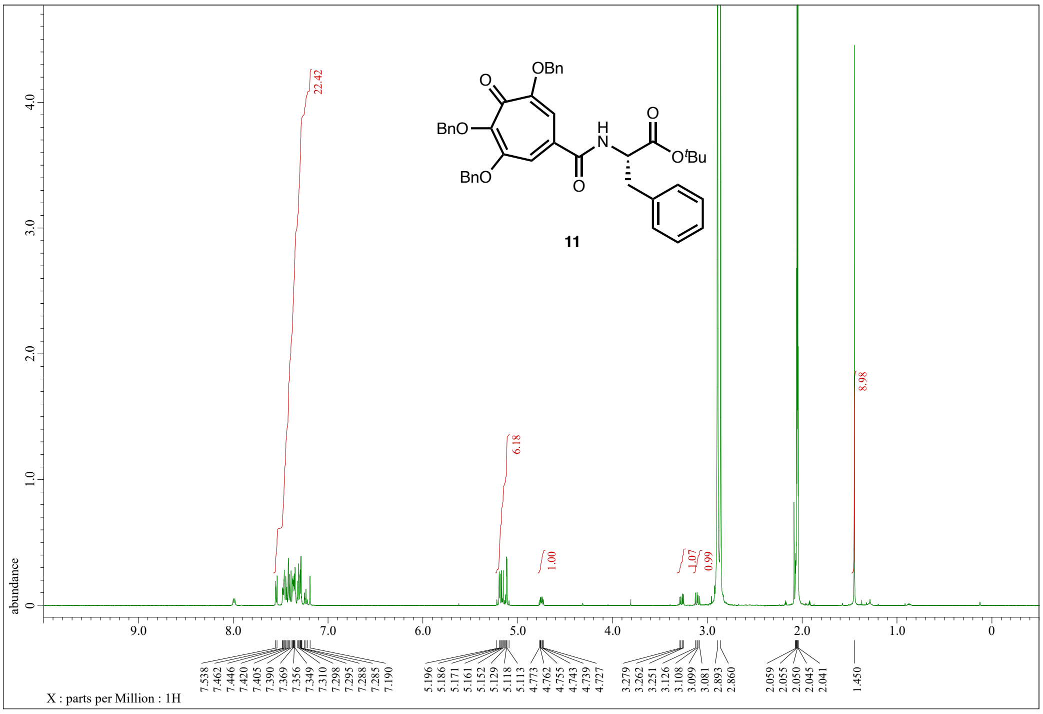

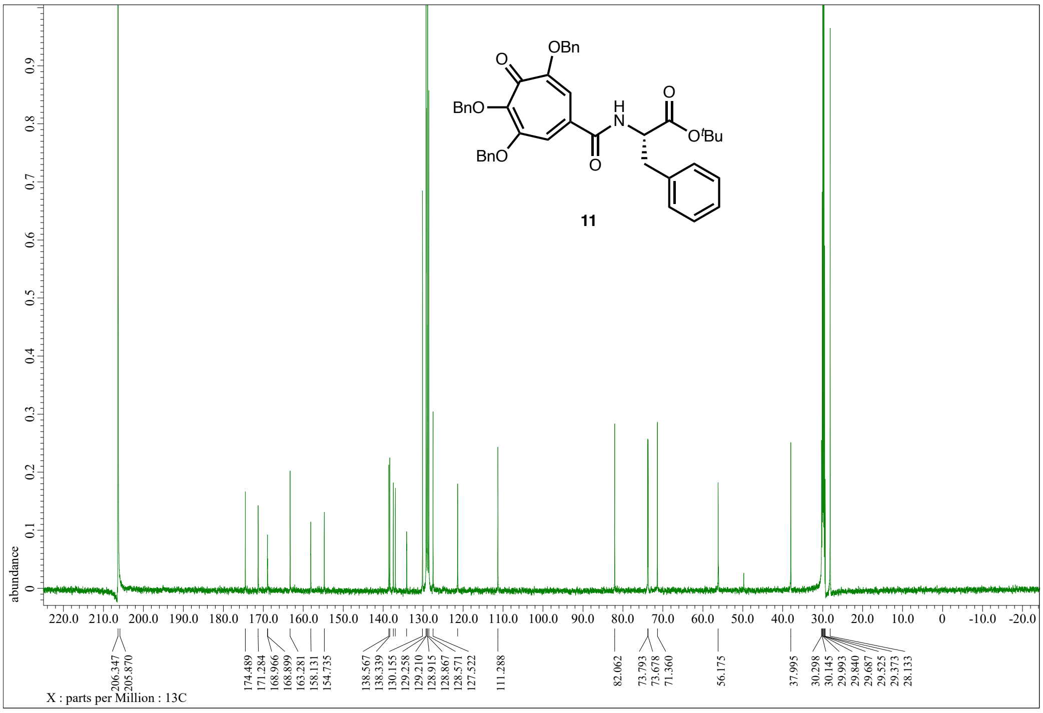

• BnO-tropolone-L-Val-O^{t}Bu (**12**): ^{1}H NMR (500 MHz, $(CD_3)_2CO$), ^{13}C NMR (125 MHz, $(CD_3)_2CO$)

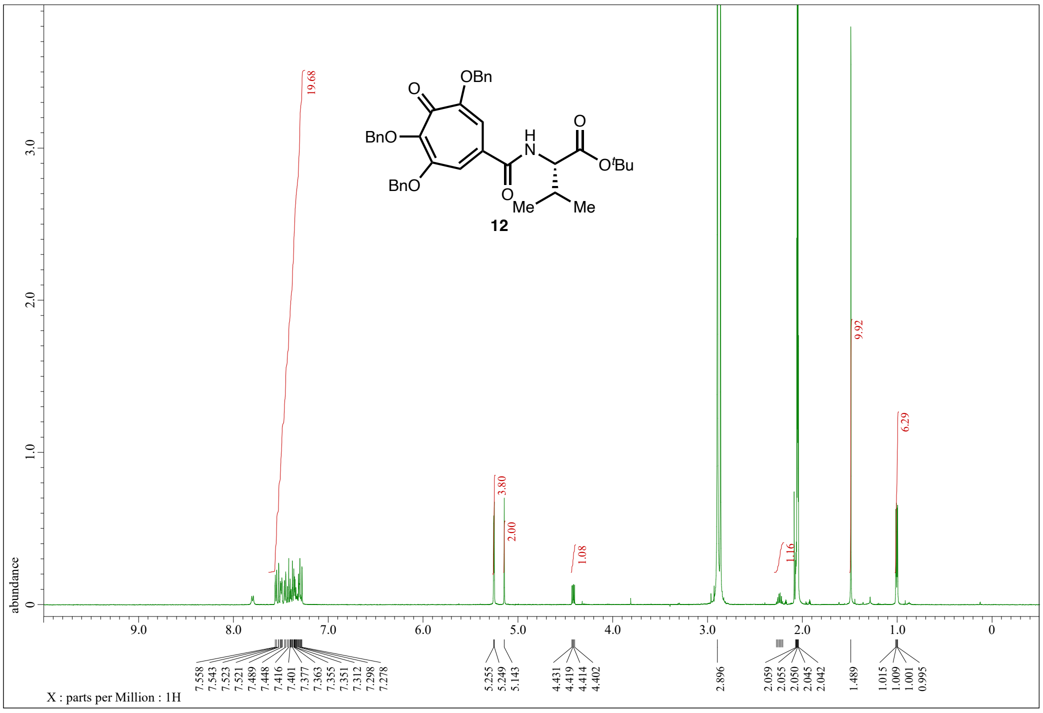

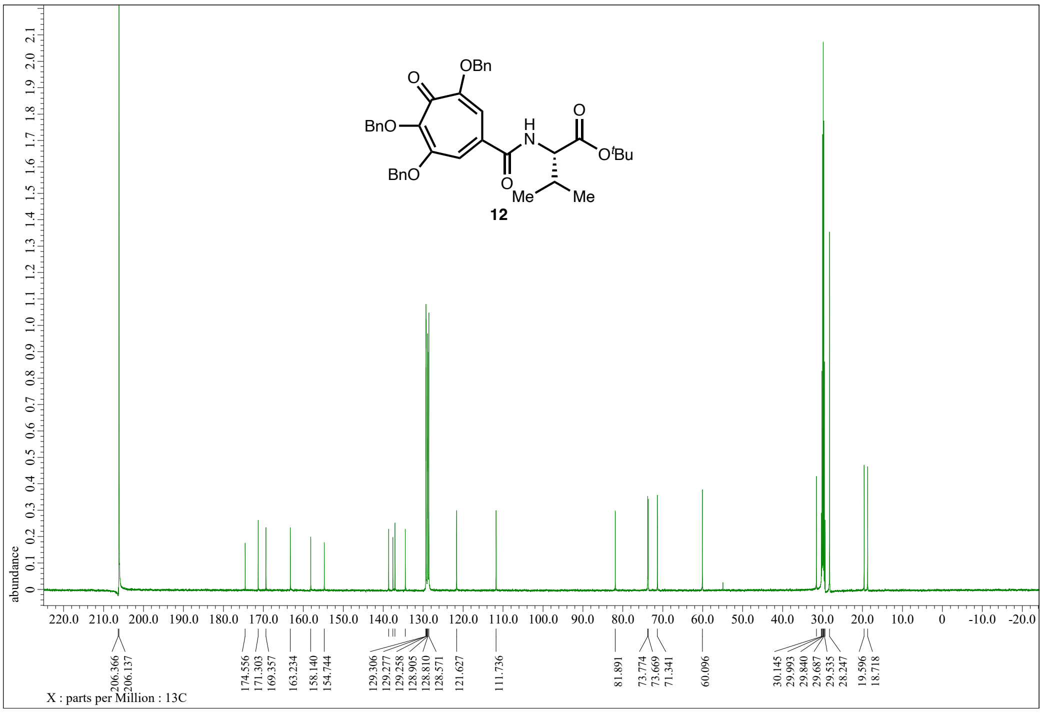

• Tropolone-L-Pro-O^{t}Bu (**9**): ^{1}H NMR (500 MHz, CD_3OD), ^{13}C NMR (125 MHz, CD_3OD)

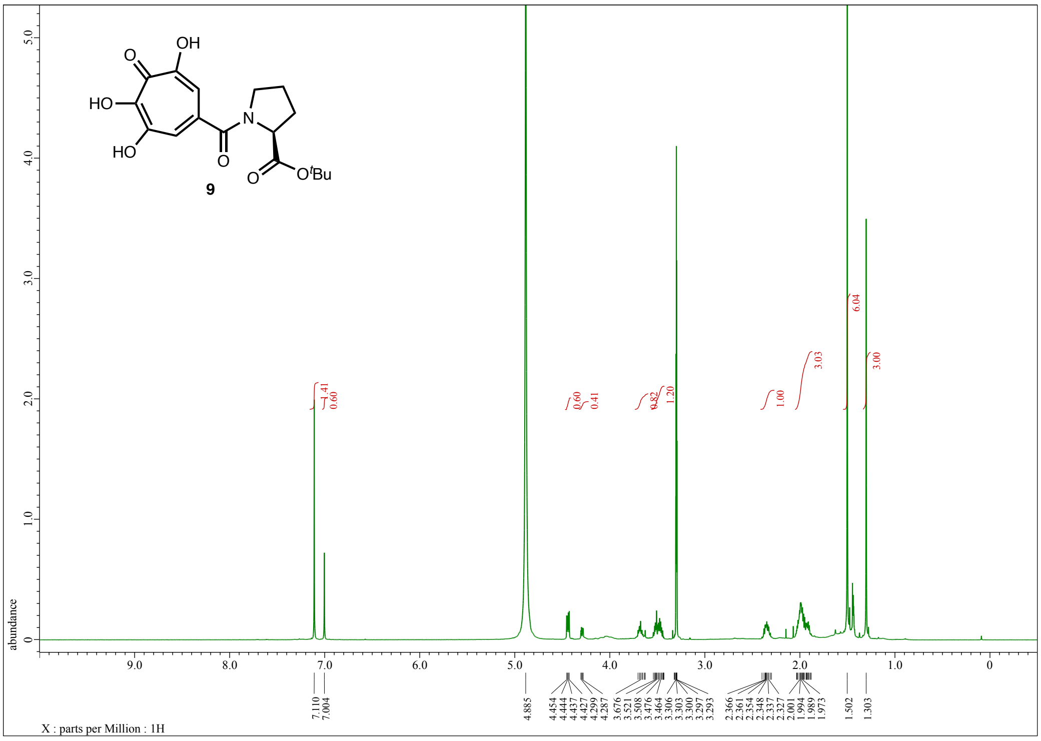

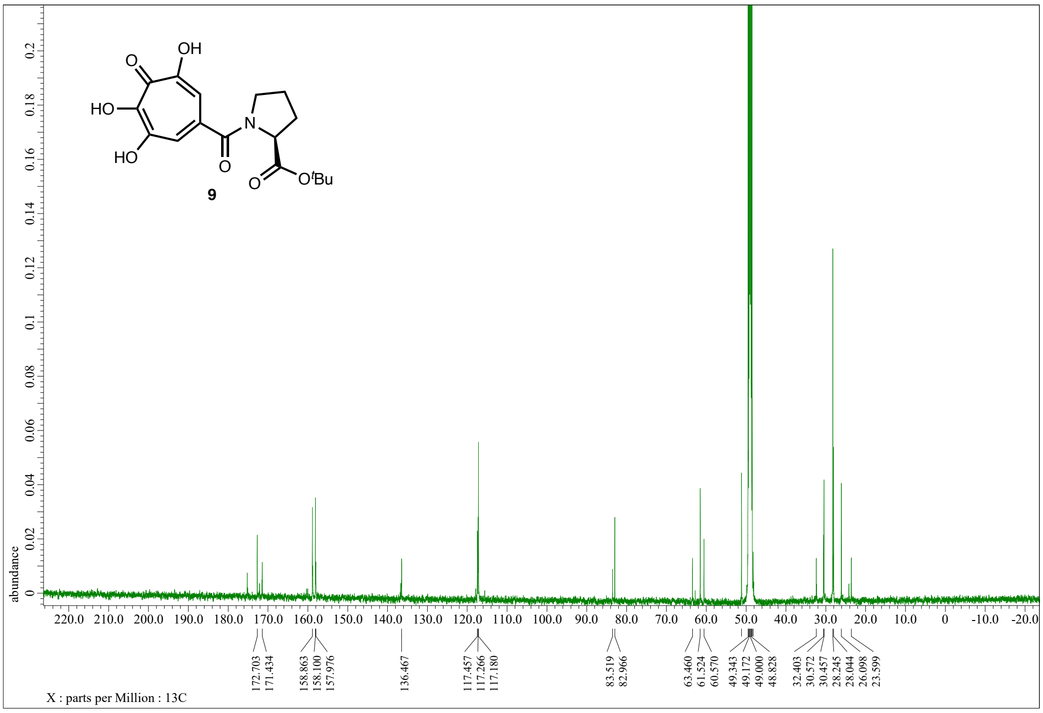

• Tropolone-Gly-O^{t}Bu (**13**): ^{1}H NMR (500 MHz, CD_3OD), ^{13}C NMR (125 MHz, CD_3OD)

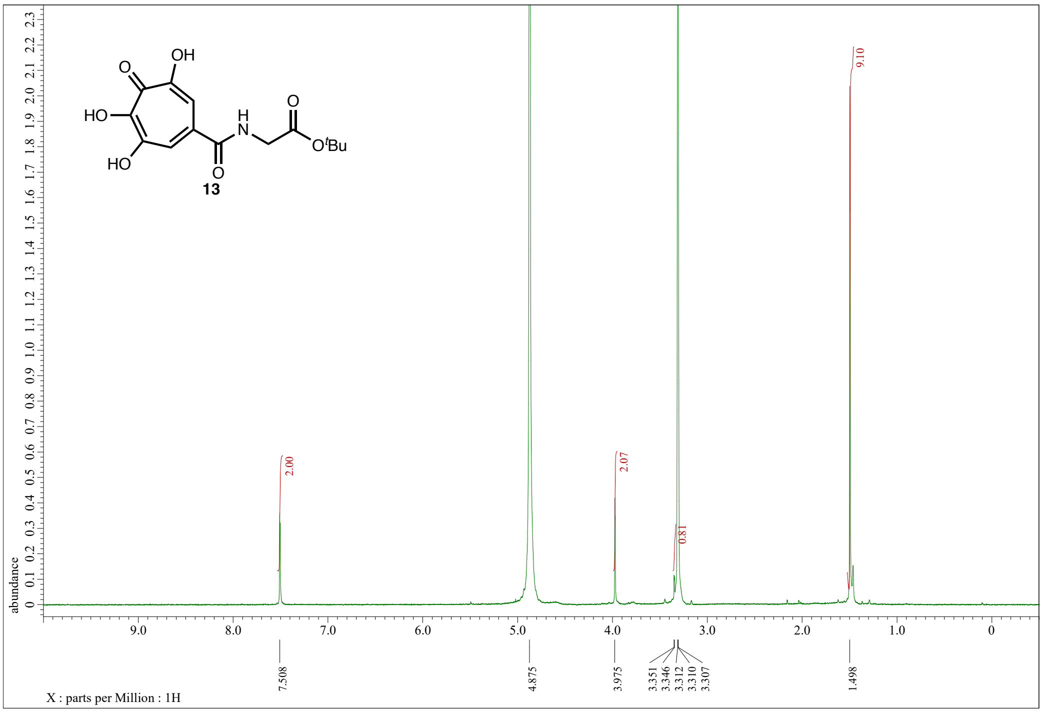

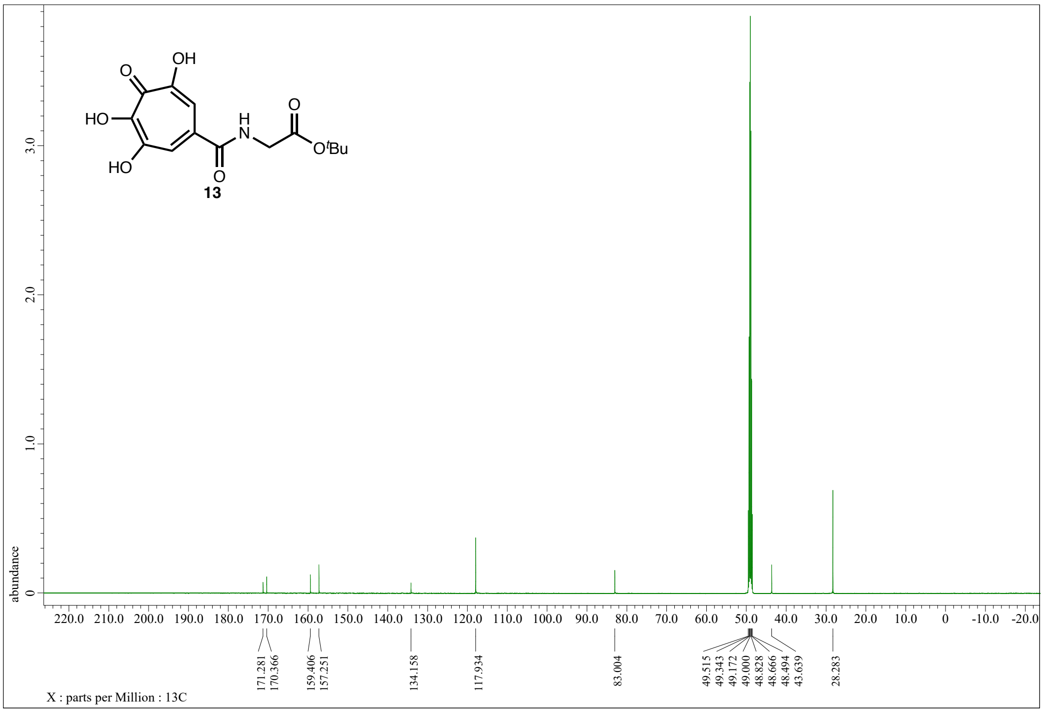

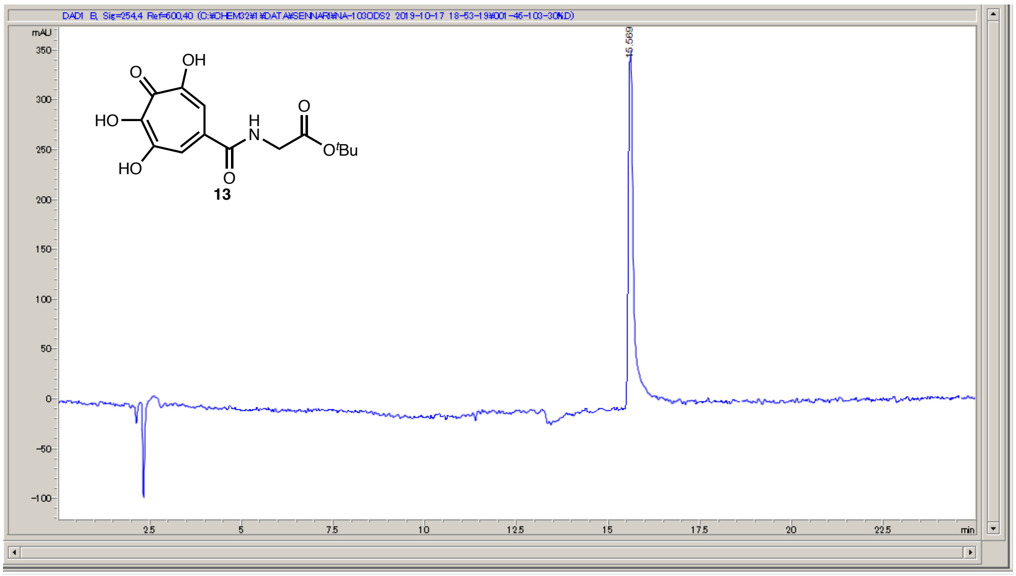

Retention time: 16.589 min

LC/UV method

Measuring equipment: Agilent 1260 Infinity LC

Column: Waters symmetry C18 Column (3.5 μm, 2.1 × 150 mm)

Mobile phase A: H_2O (containing 0.05% H_3PO_4)

Mobile phase B: MeCN (containing 0.05% H_3PO_4)

Linear gradient: A:B = 95:5 to 0:100 (0 – 25 min)

Flow late: 0.2 mL/min

Detect: UV 254 nm

Temperature: 40 ˚C

• Tropolone-L-Phe-O^{t}Bu (**14**): ^{1}H NMR (500 MHz, CD_3OD), ^{13}C NMR (125 MHz, CD_3OD)

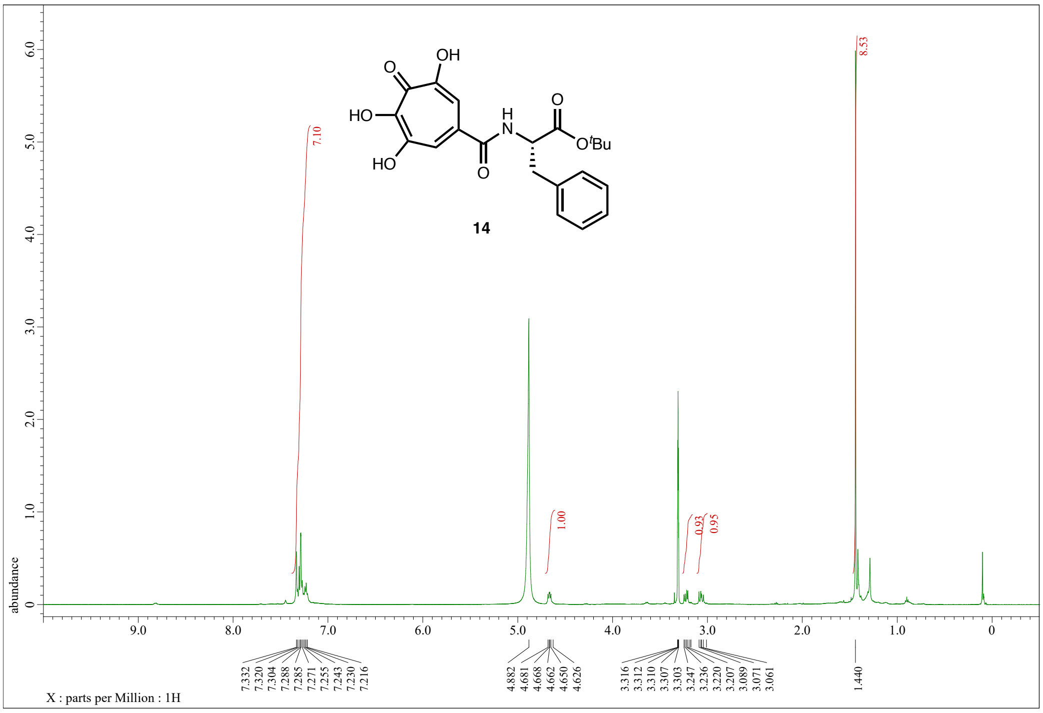

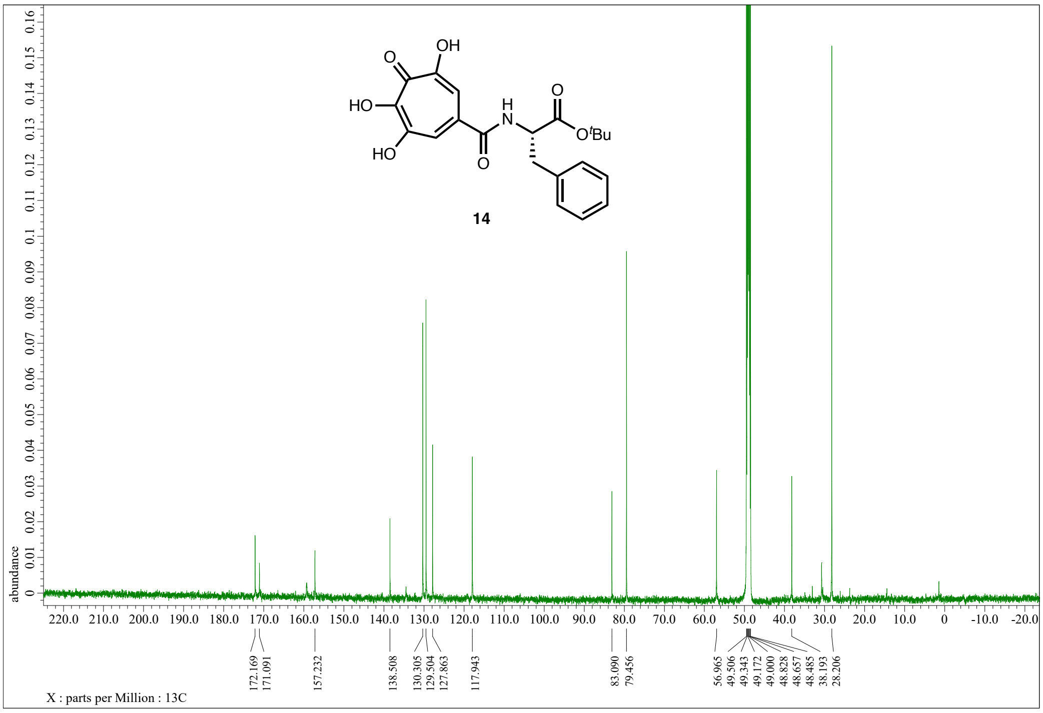

• Tropolone-L-Val-O^{t}Bu (**15**): ^{1}H NMR (500 MHz, CD_3OD), ^{13}C NMR (125 MHz, CD_3OD)

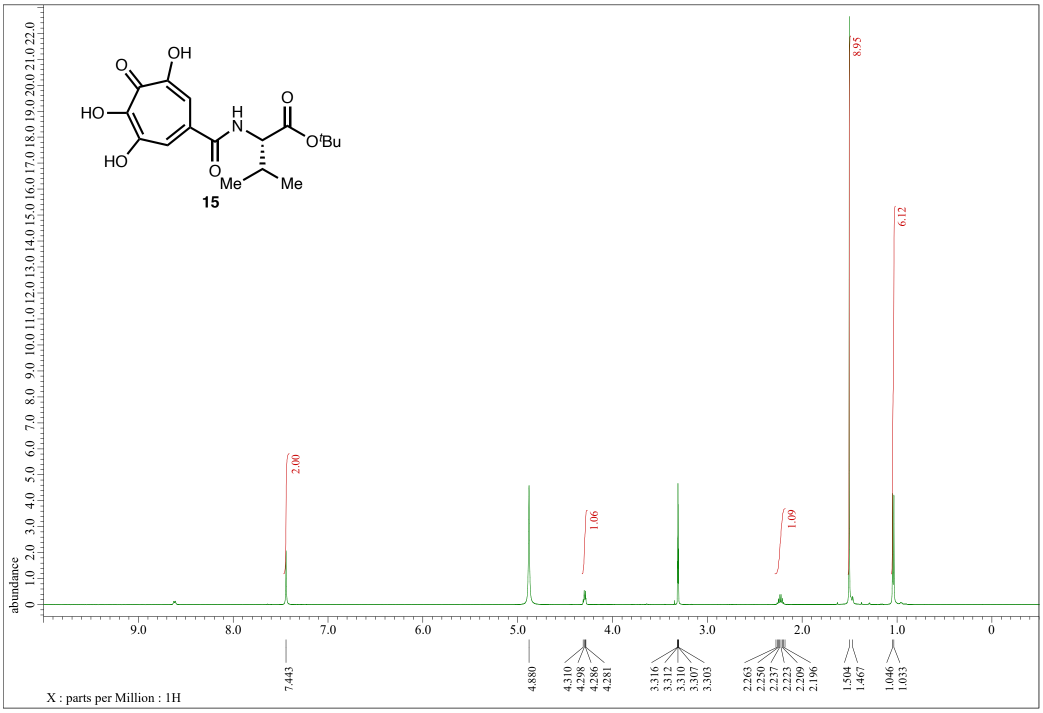

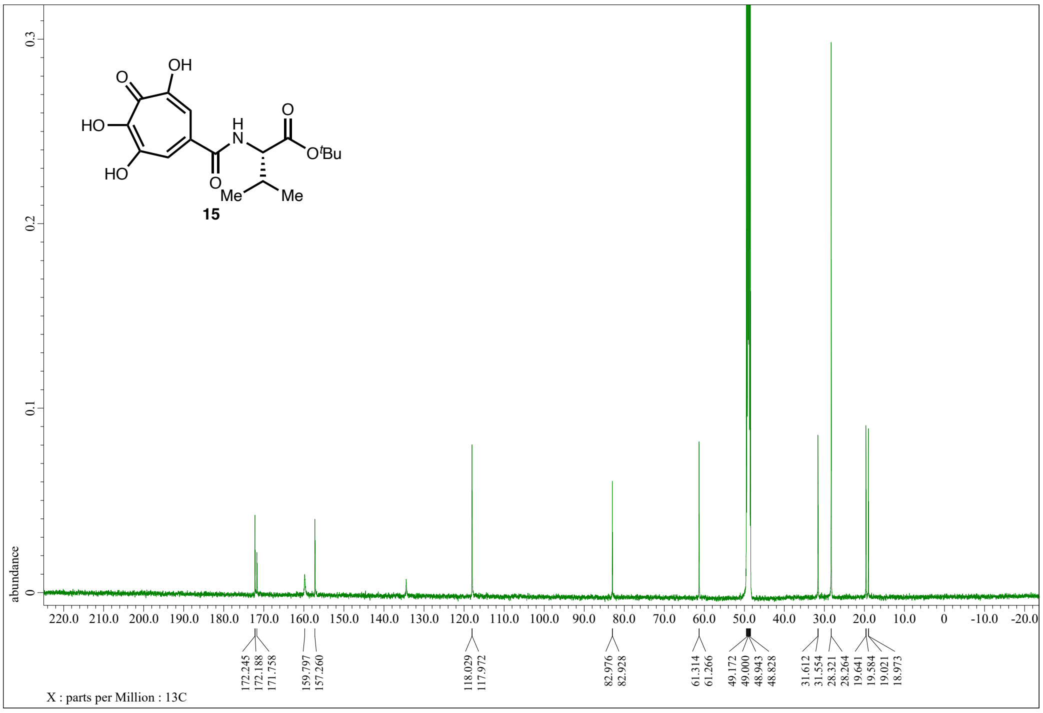

• Tropolone-L-Pro-OH (**8**): ^{1}H NMR (500 MHz, CD_3OD), ^{13}C NMR (125 MHz, CD_3OD)

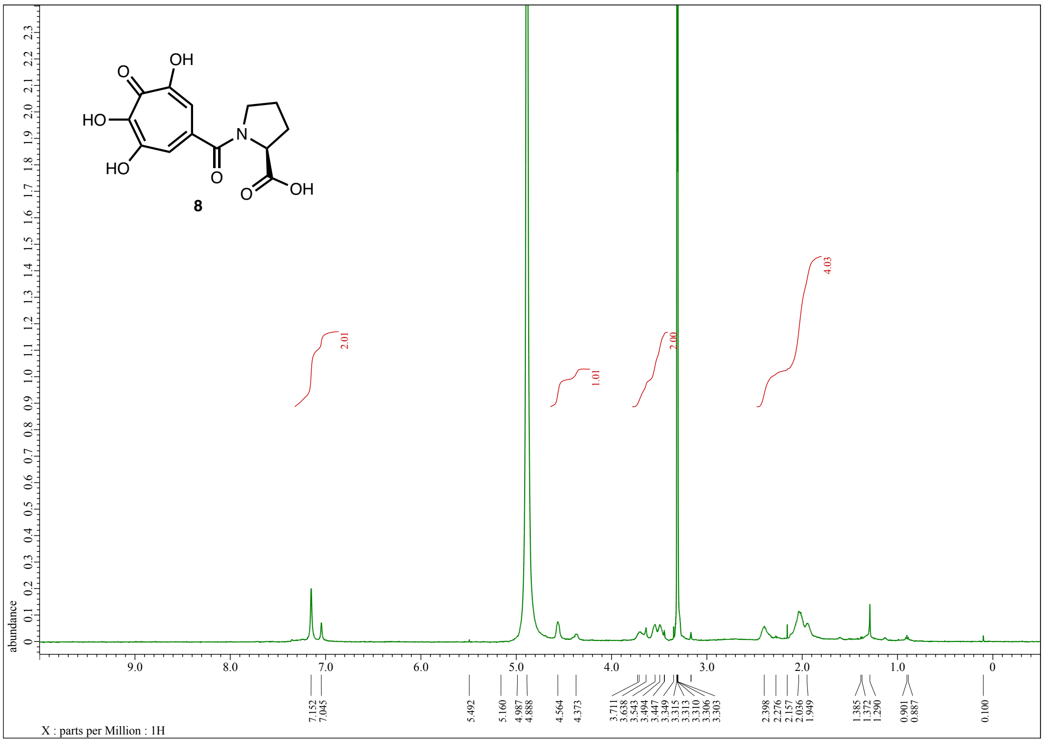

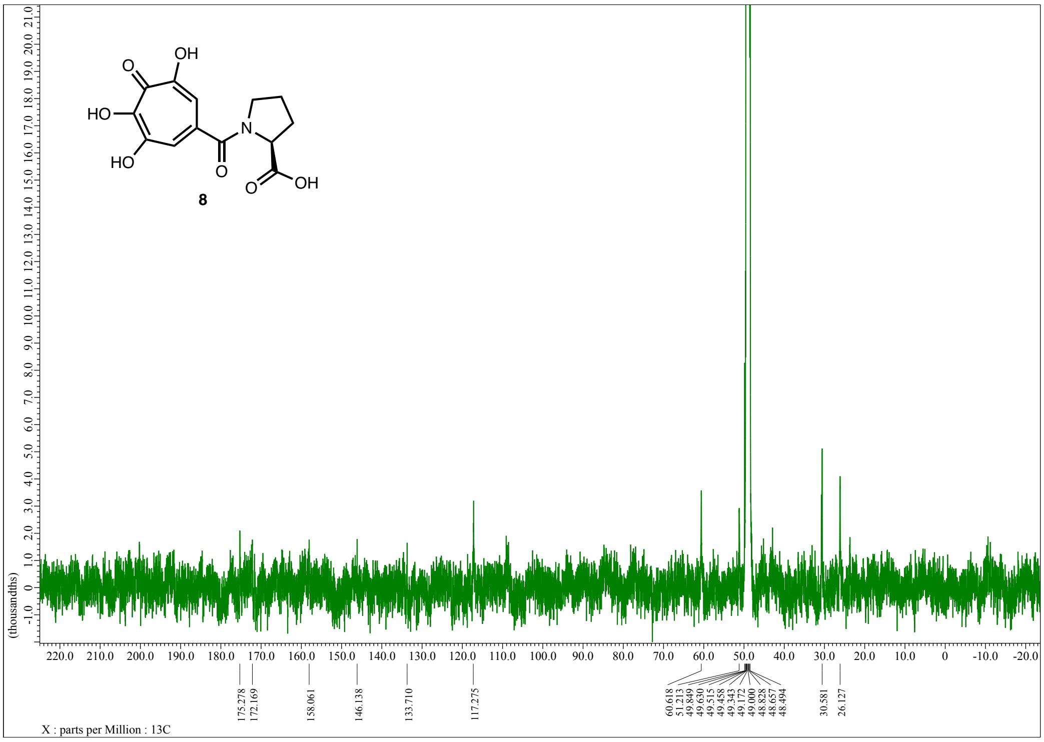

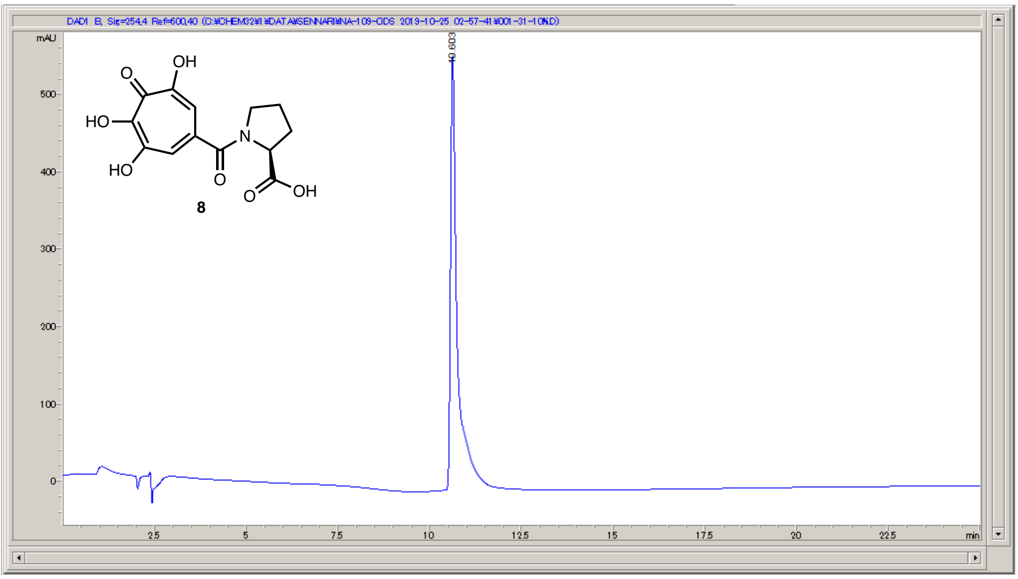

Retention time: 10.603 min

<u>**LC/UV method**</u>

Measuring equipment: Agilent 1260 Infinity LC

Column: Waters symmetry C18 Column (3.5 µm, 2.1 × 150 mm)

Mobile phase A: H_2O (containing 0.05% H_3PO_4)

Mobile phase B: MeCN (containing 0.05% H_3PO_4)

Linear gradient: A:B = 95:5 to 0:100 (0 – 25 min)

Flow late: 0.2 mL/min

Detect: UV 254 nm

Temperature: 40 ˚C

• Tropolone-Gly-OH (**6**): ^{1}H NMR (500 MHz, $(CD_3)_2SO$), ^{13}C NMR (125 MHz, $(CD_3)_2SO$)

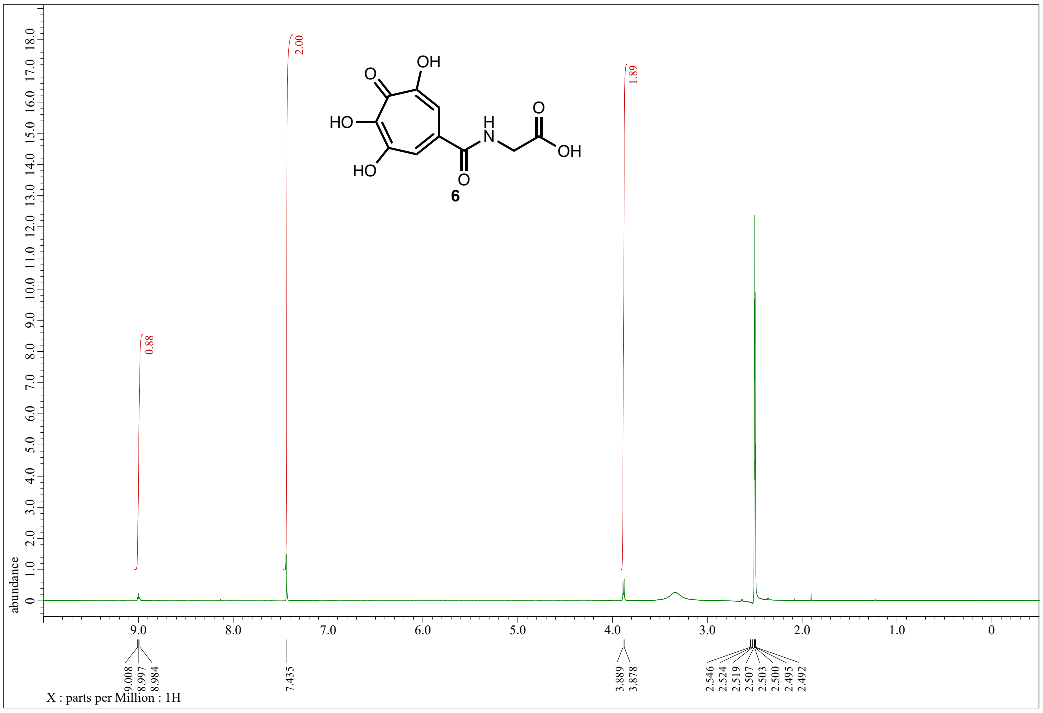

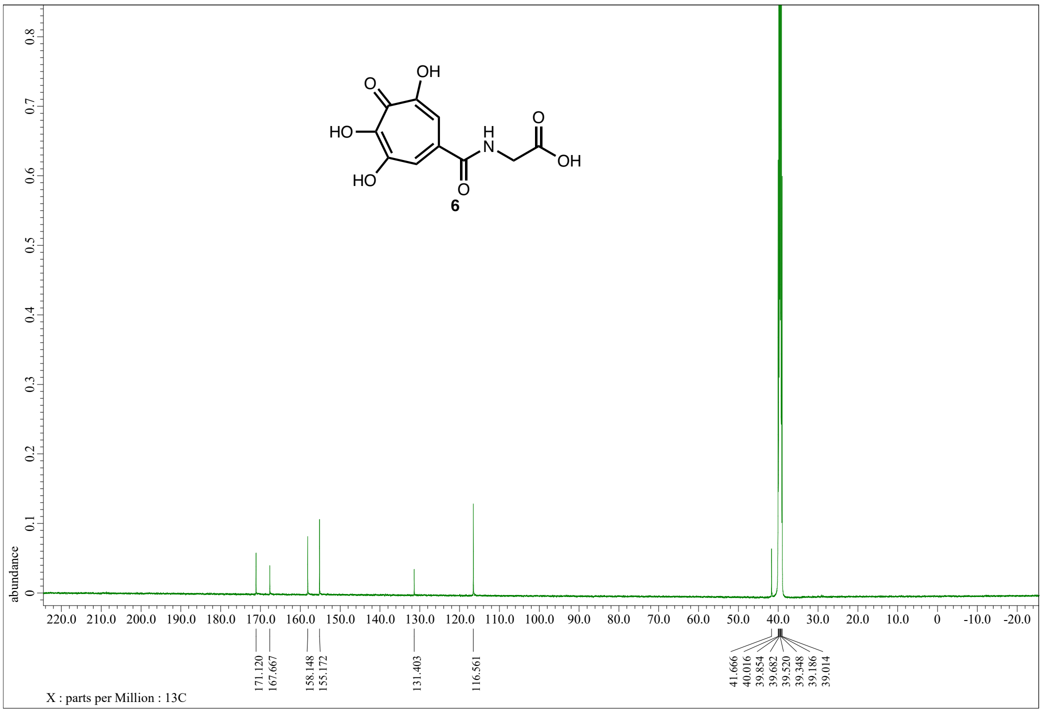

• Tropolone-L-Phe-OH (**16**): ^{1}H NMR (500 MHz, CD_3OD), ^{13}C NMR (125 MHz, CD_3OD)

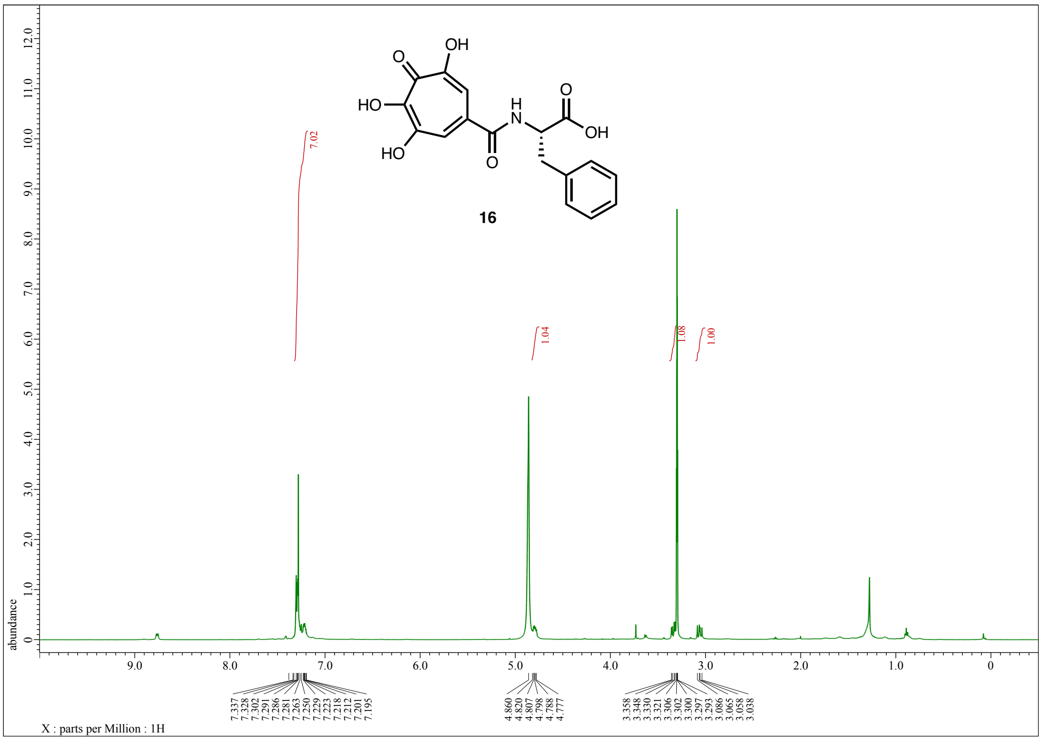

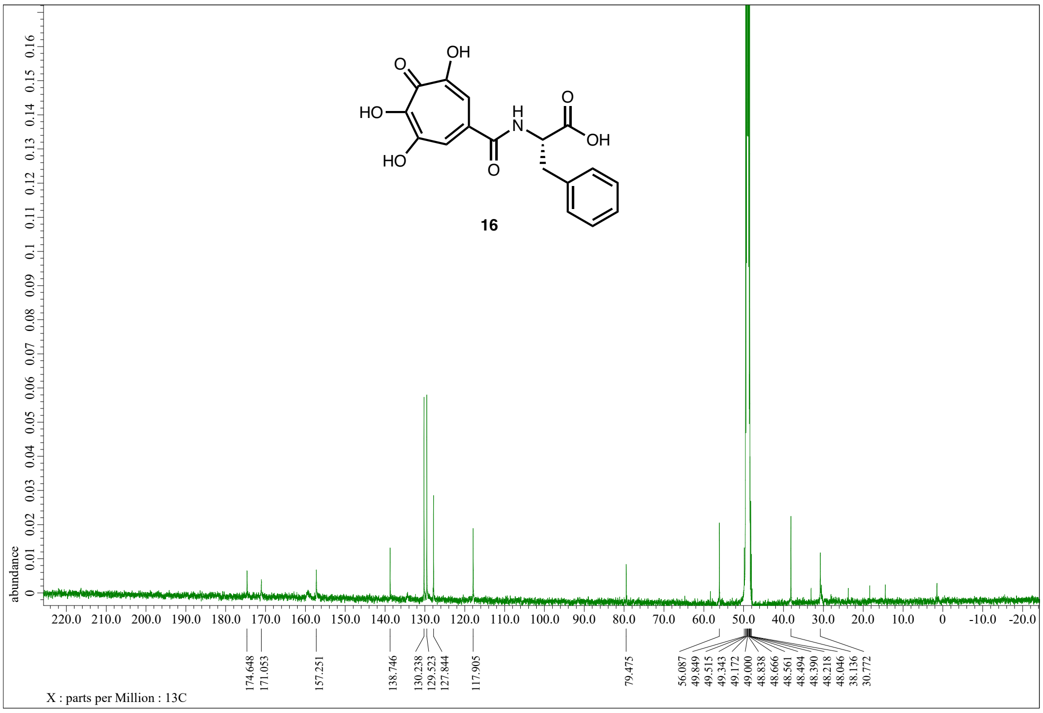

• Tropolone-L-Val-OH (**17**): ^{1}H NMR (500 MHz, CD_3OD), ^{13}C NMR (125 MHz, CD_3OD)

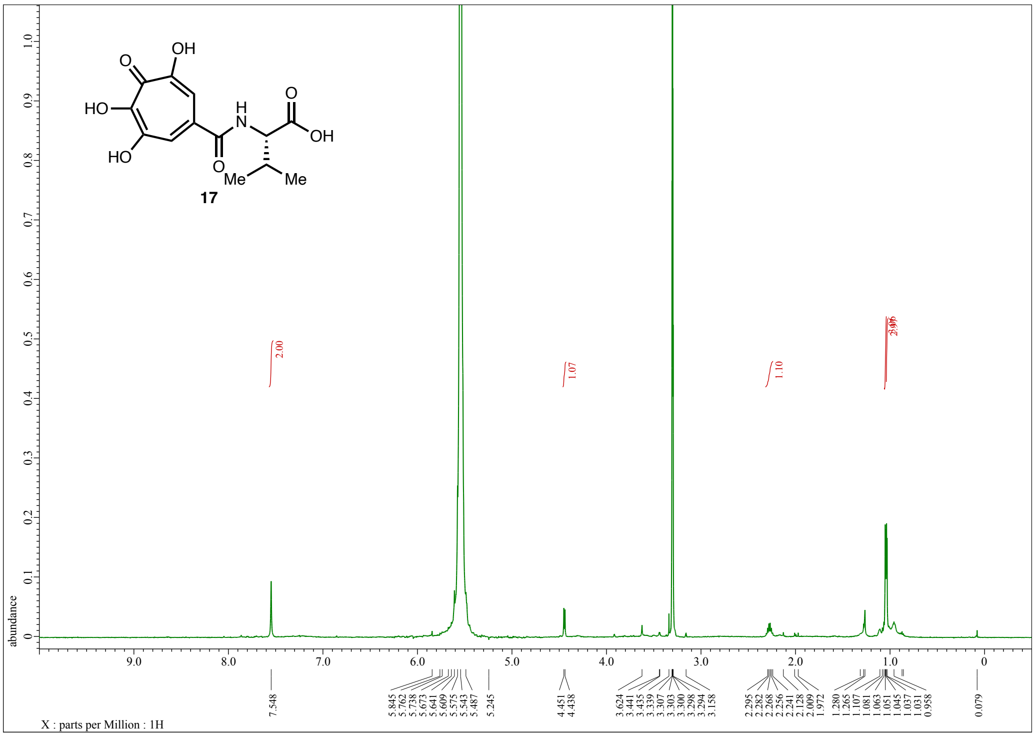

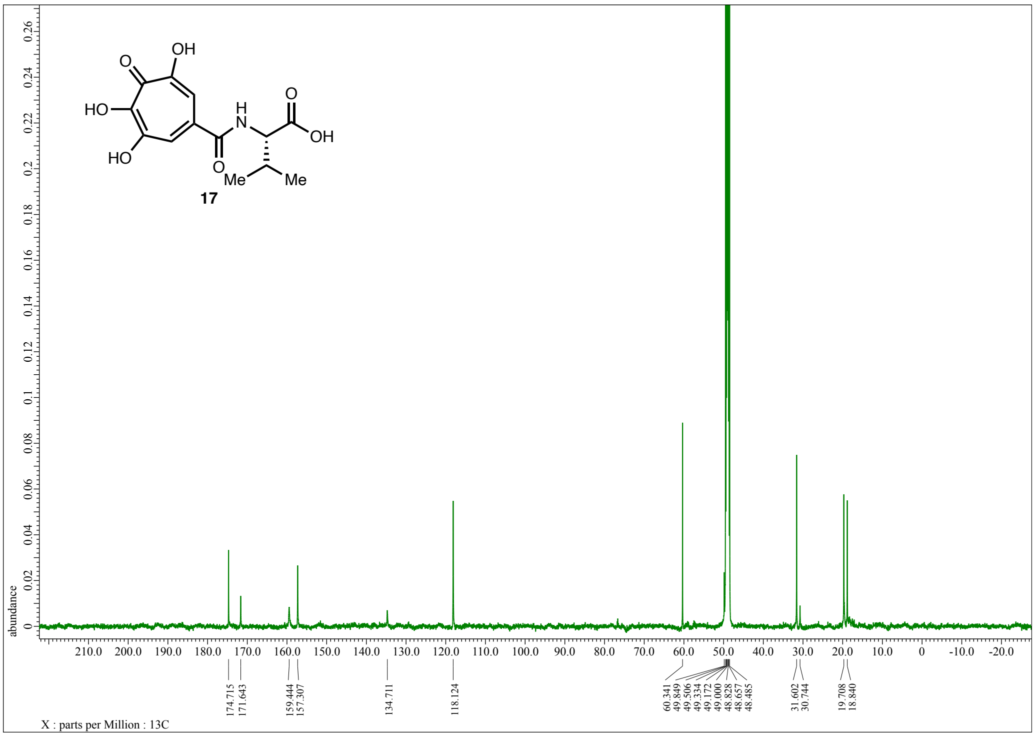

• Fmoc-D-Val-OTAG (**S-4a**): ^{1}H NMR (500 MHz, $CDCl_3$), ^{13}C NMR (125 MHz, $CDCl_3$)

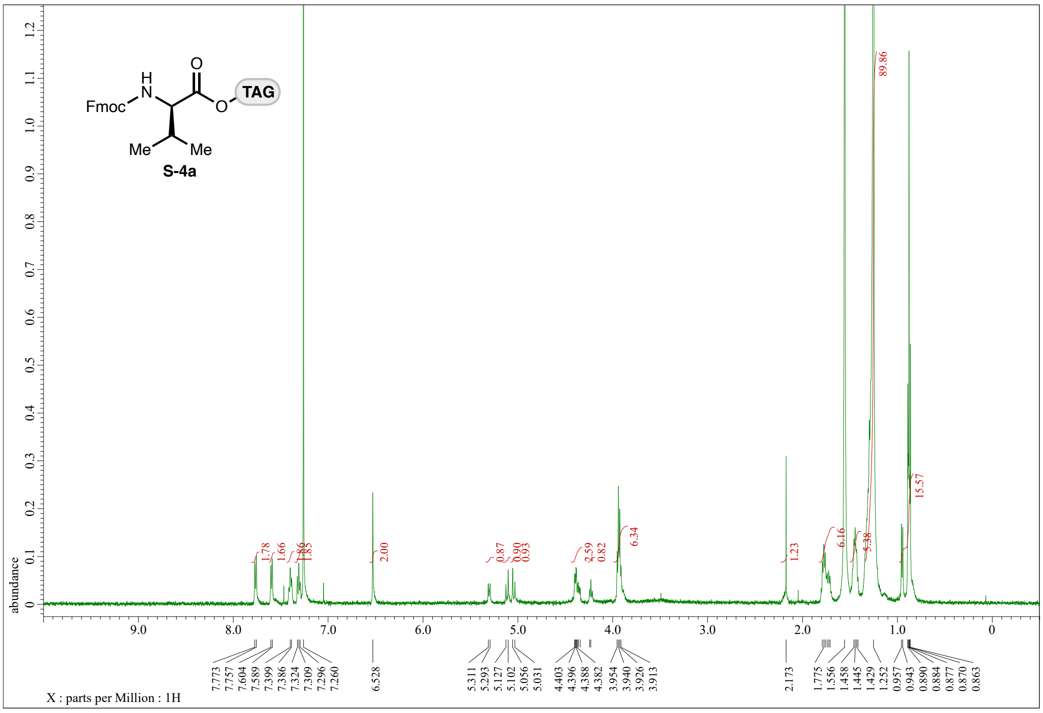

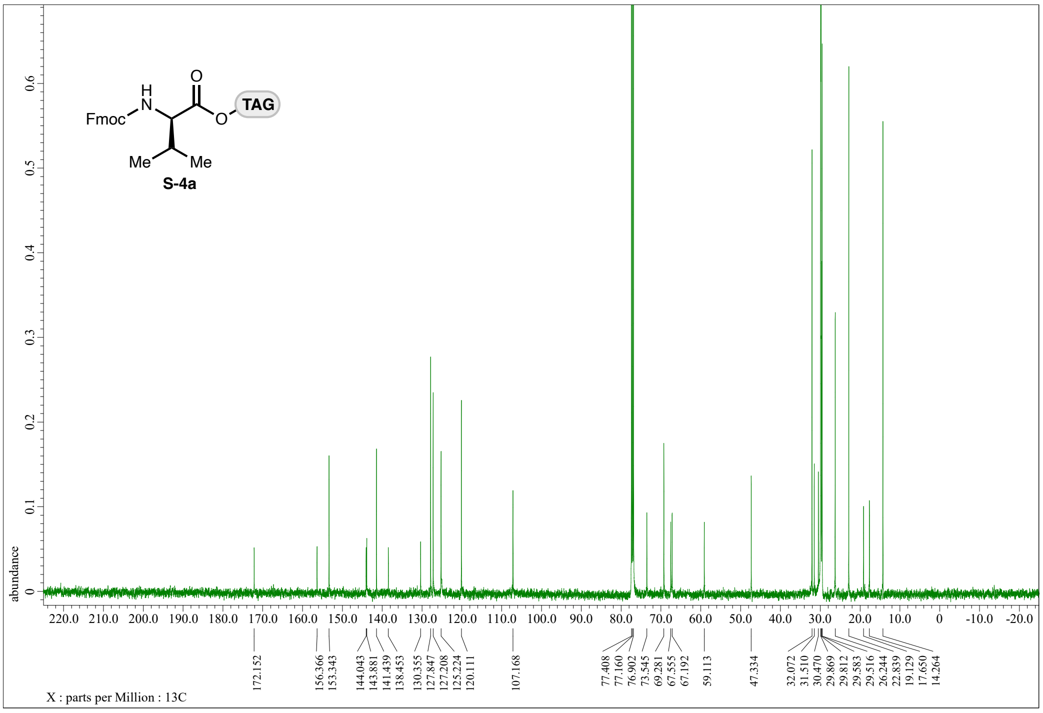

• Fmoc-*N*-Me-L-leu-OTAG (**S-4c**): ^{1}H NMR (500 MHz, $CDCl_3$), ^{13}C NMR (125 MHz, $CDCl_3$)

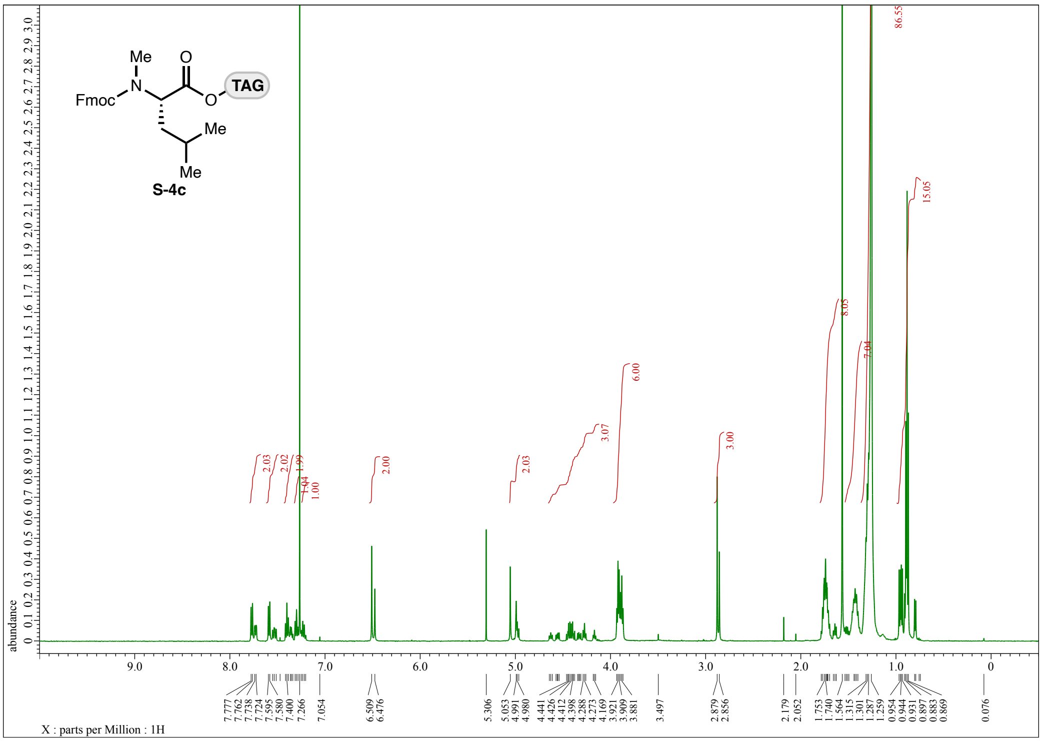

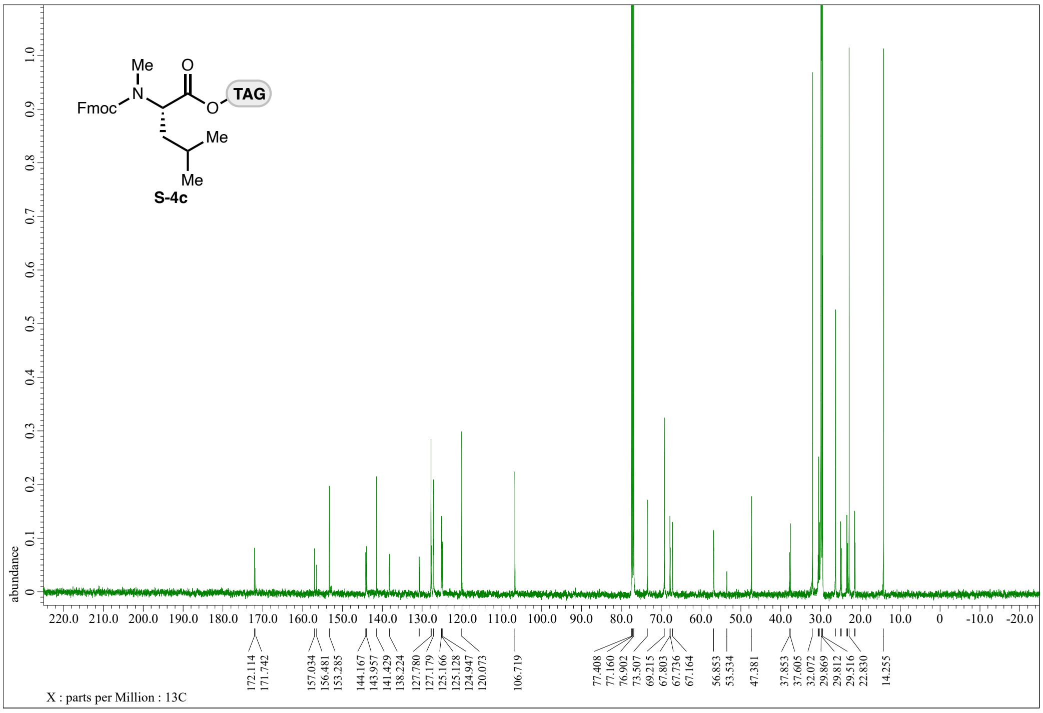

• Fmoc-L-Ile-OTAG (**S-4d**): ^{1}H NMR (500 MHz, $CDCl_3$), ^{13}C NMR (125 MHz, $CDCl_3$)

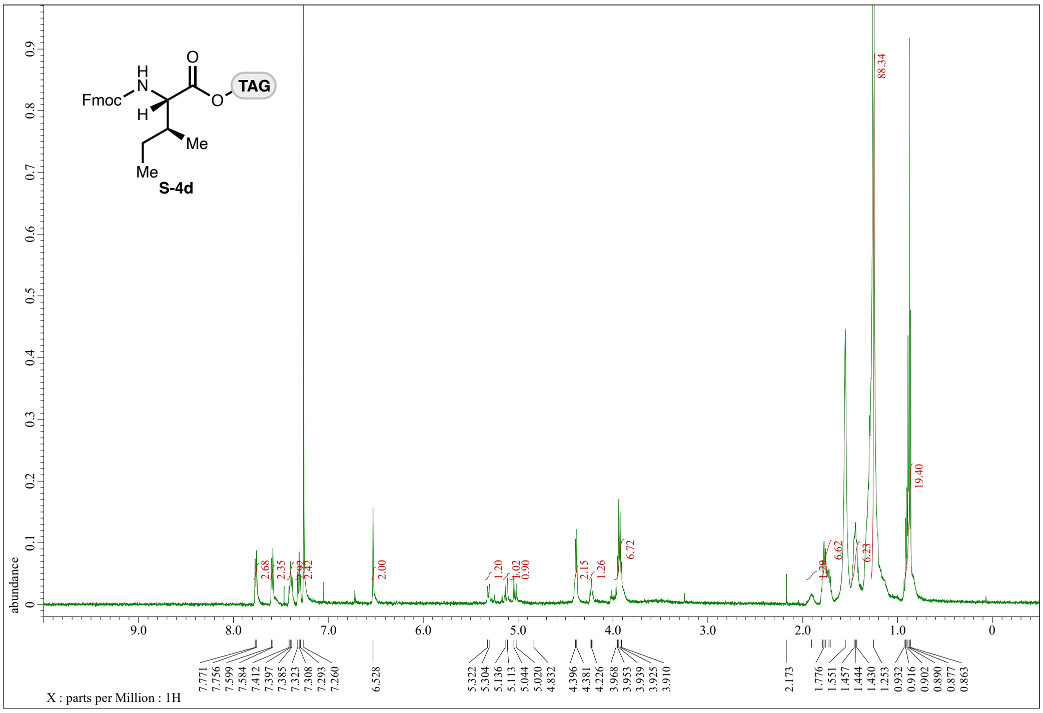

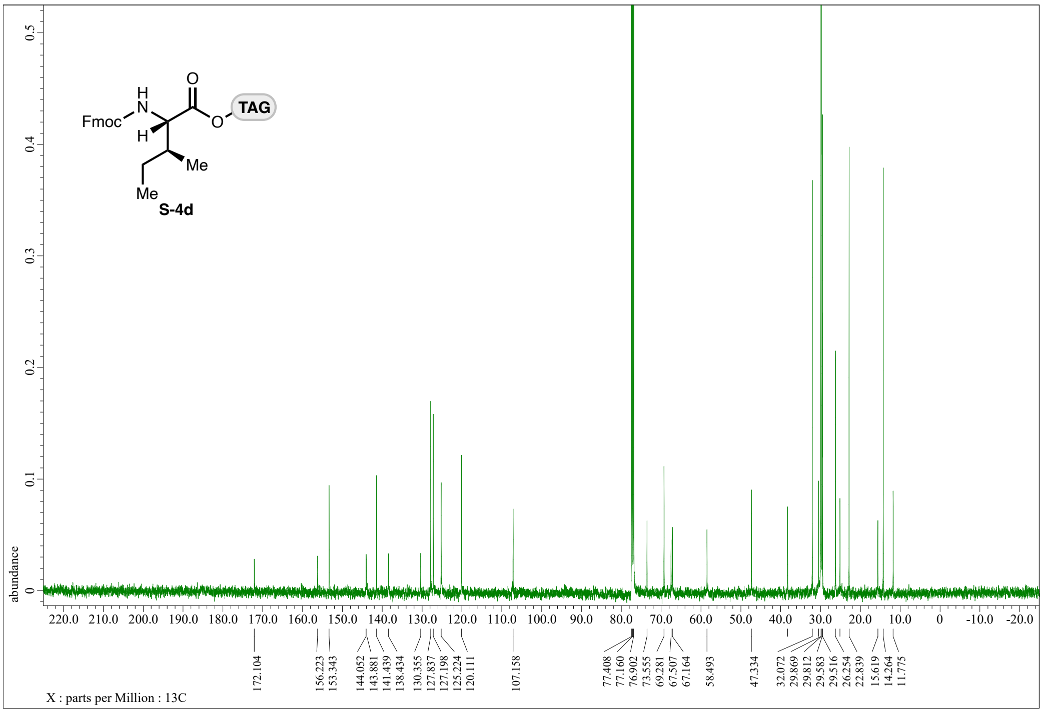

• Fmoc-L-Thr(^{t}Bu)-OTAG (**S-4e**): ^{1}H NMR (500 MHz, $CDCl_3$), ^{13}C NMR (125 MHz, $CDCl_3$)

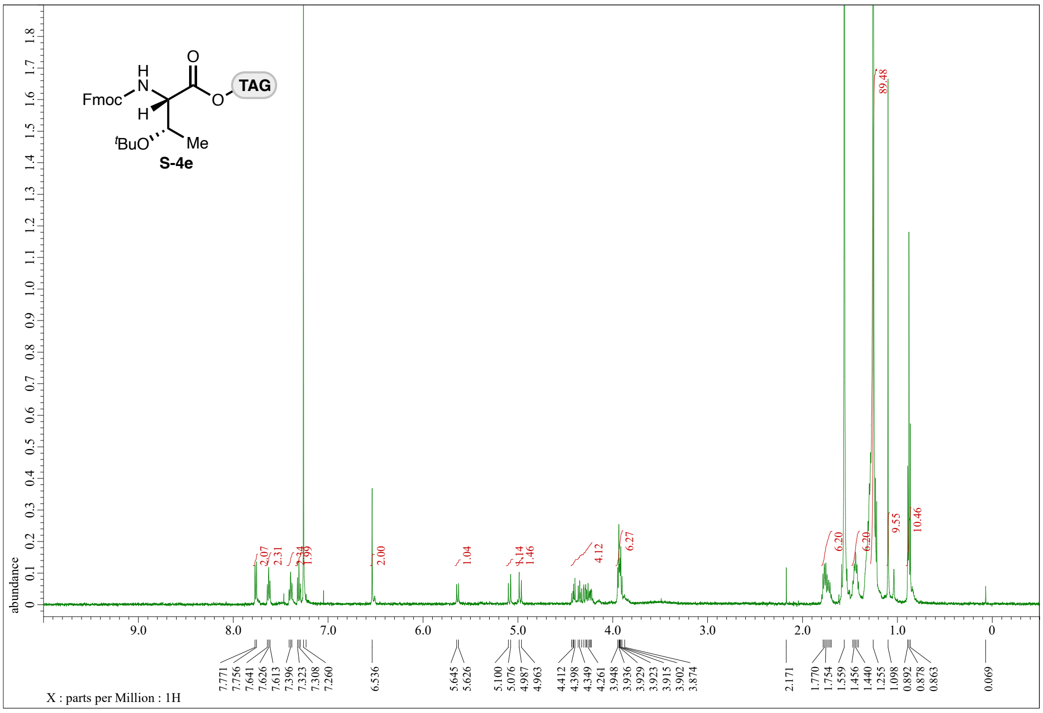

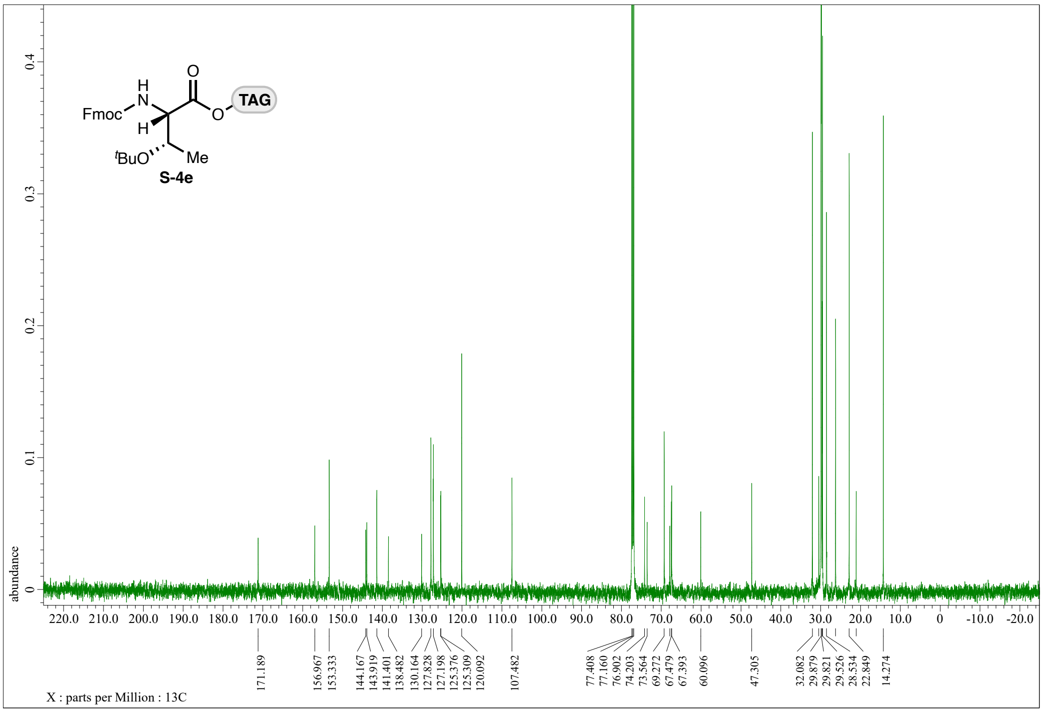

• Fmoc-L-Met-OTAG (**S-4f**): ^{1}H NMR (500 MHz, $CDCl_3$), ^{13}C NMR (125 MHz, $CDCl_3$)

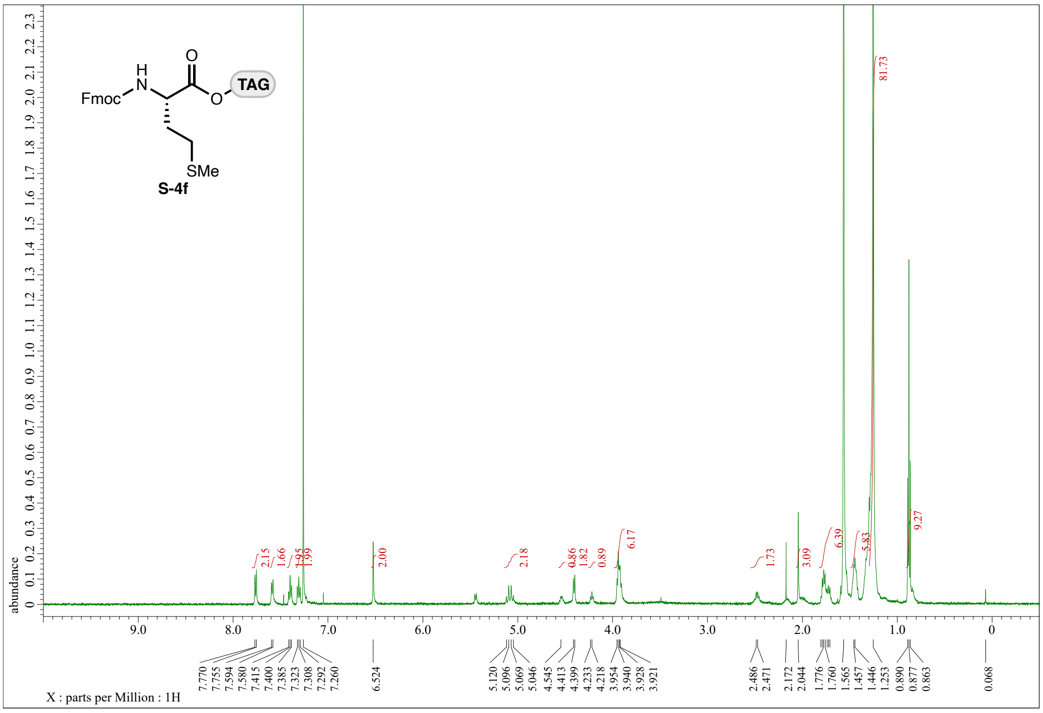

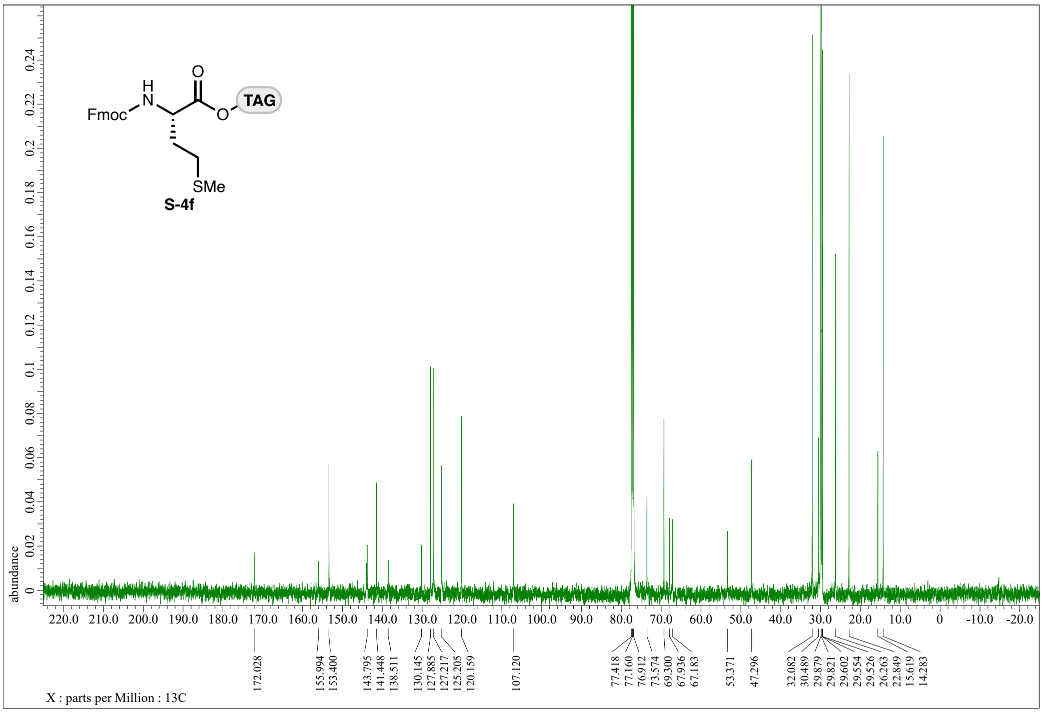

• Fmoc-L-Asp(^{t}Bu)-OTAG (**S-4g**)

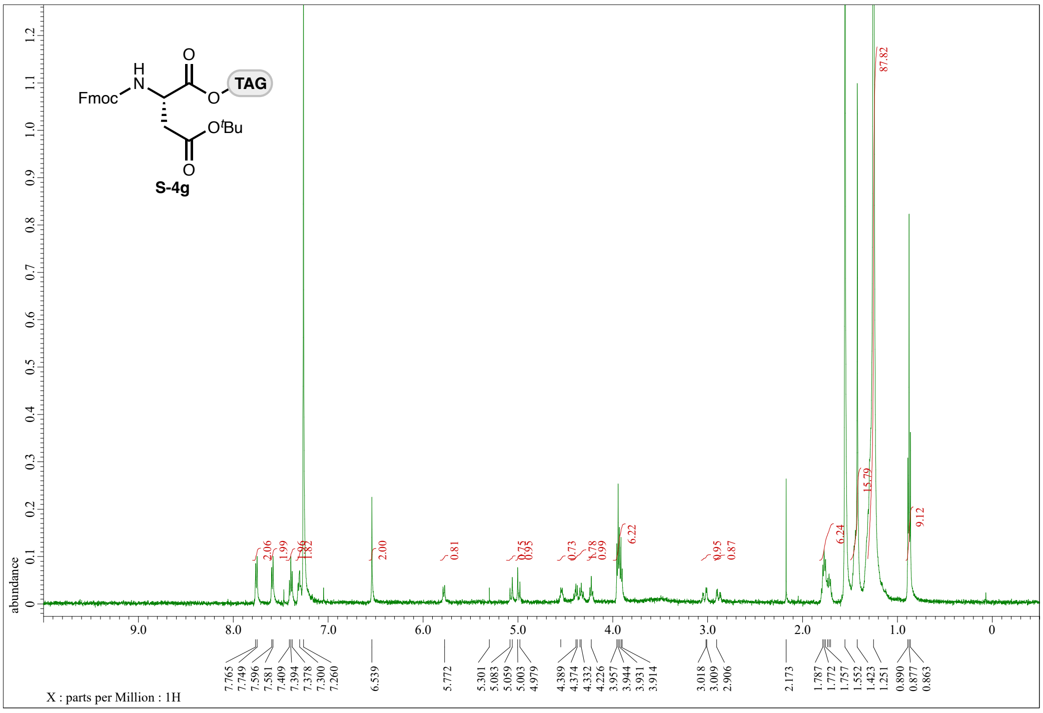

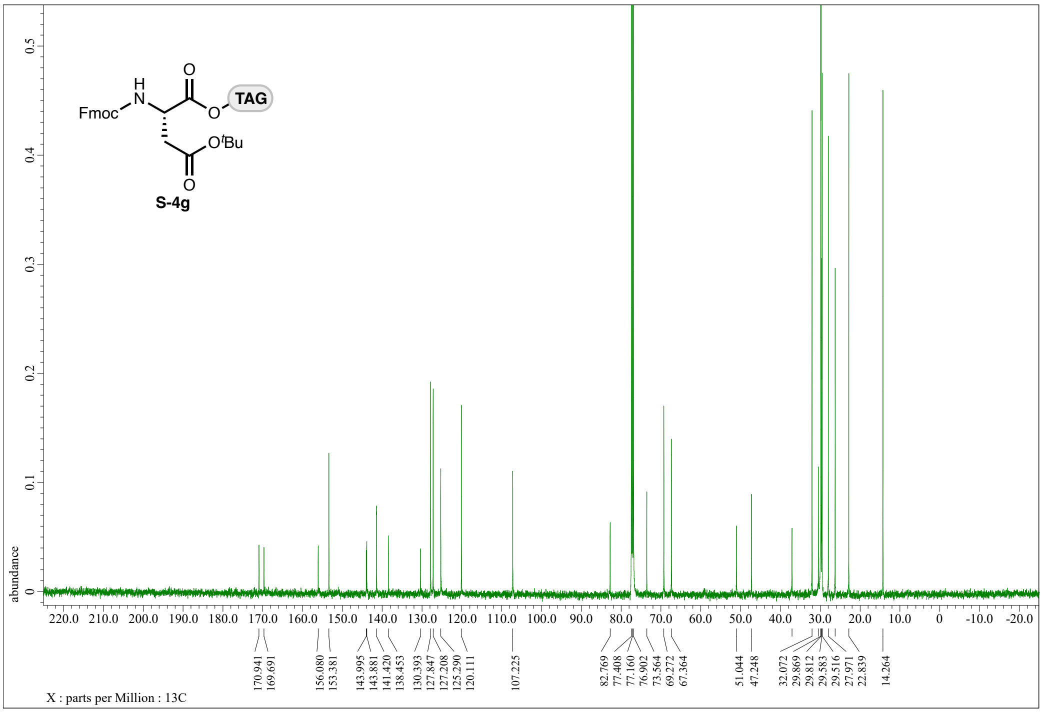

• Fmoc-L-Orn(Boc)-OTAG (**S-4h**): ^{1}H NMR (500 MHz, $CDCl_3$), ^{13}C NMR (125 MHz, $CDCl_3$)

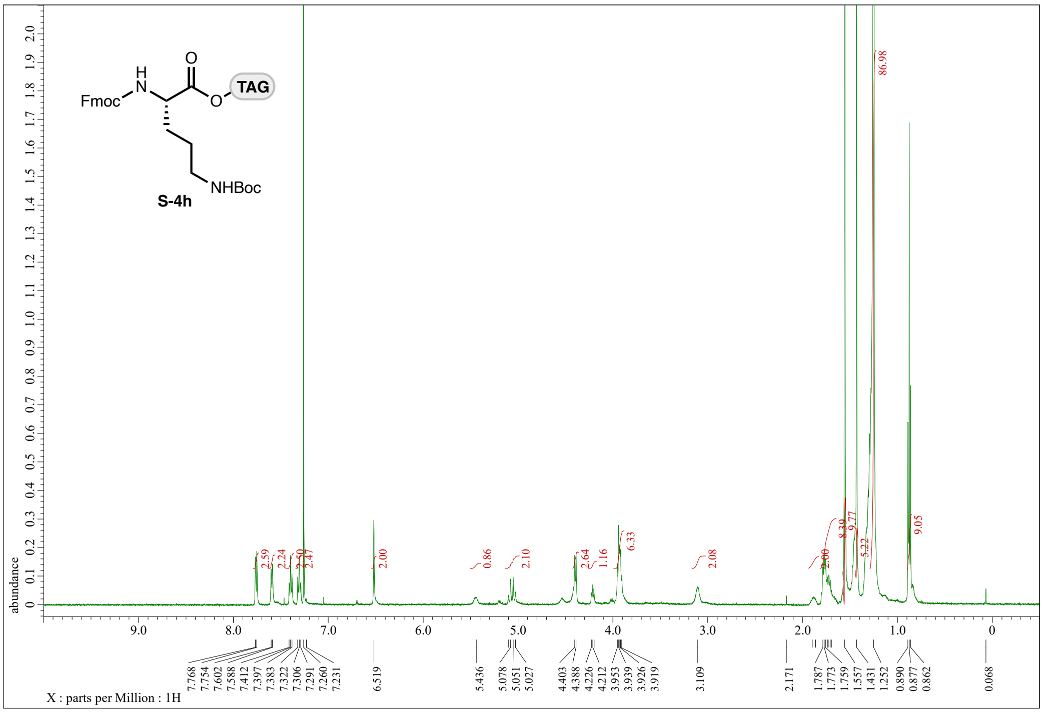

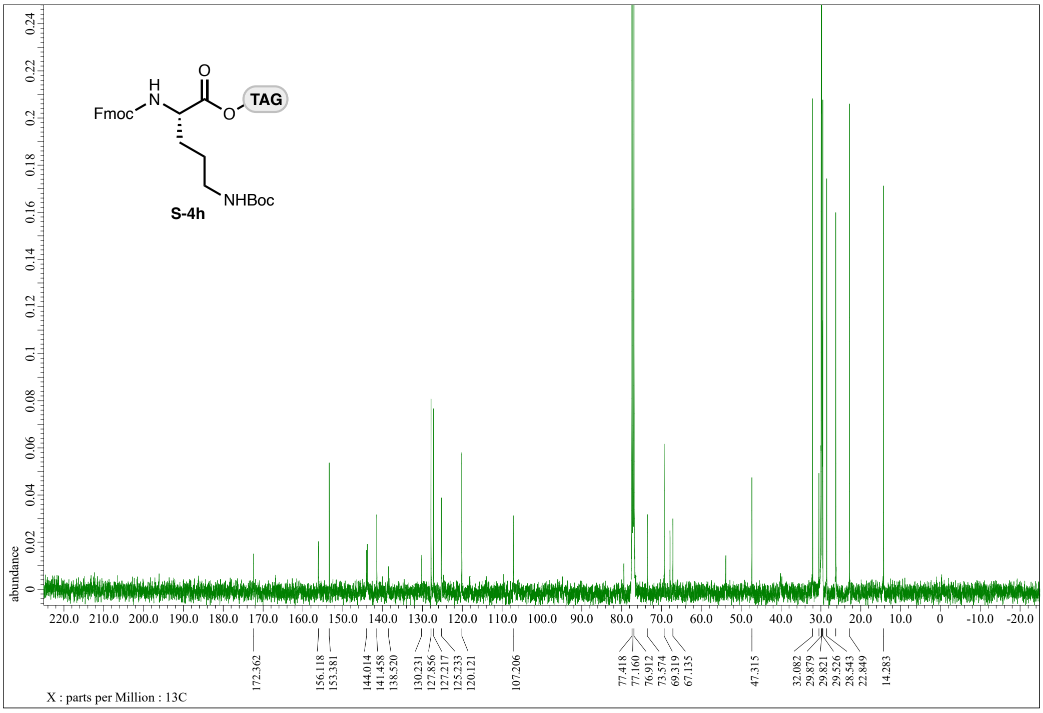

• Fmoc-L-His(Boc)-OTAG (**S-4i**): ^{1}H NMR (500 MHz, $CDCl_3$), ^{13}C NMR (125 MHz, $CDCl_3$)

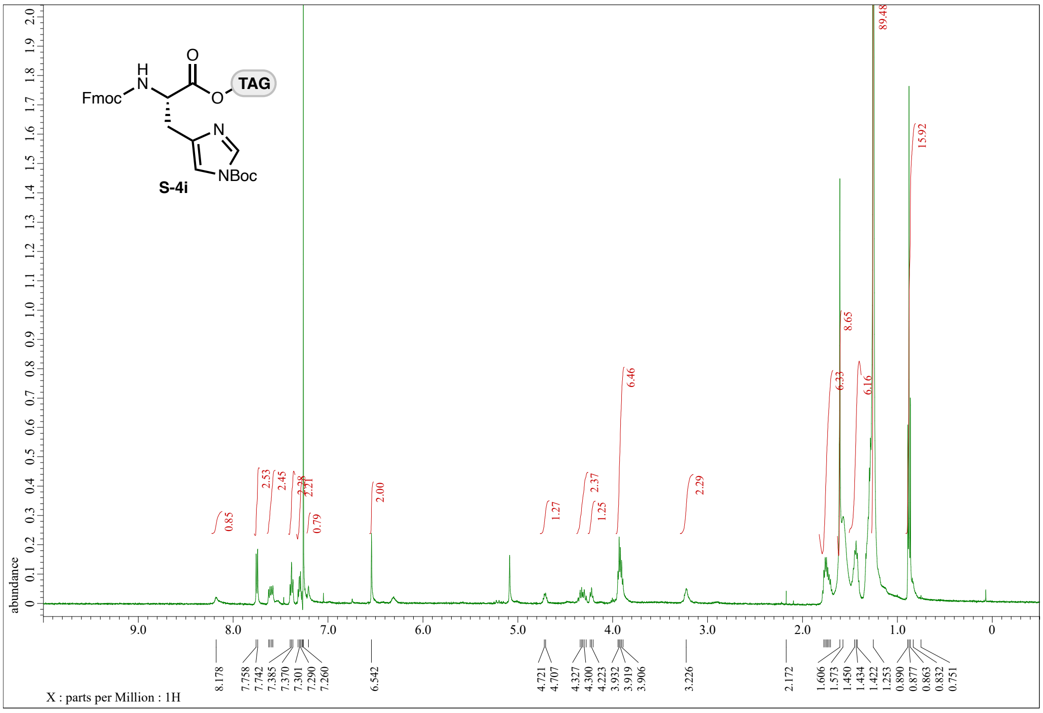

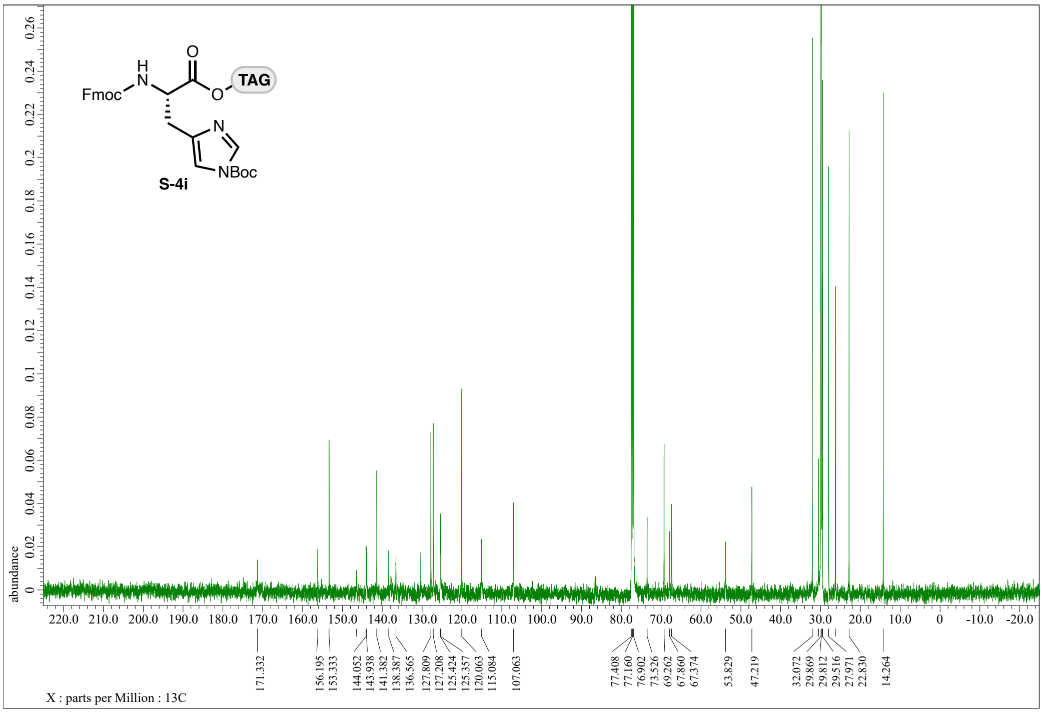

• Fmoc-*N*-Me-L-Phe-OTAG (**S-4j**): ^{1}H NMR (500 MHz, $CDCl_3$), ^{13}C NMR (125 MHz, $CDCl_3$)

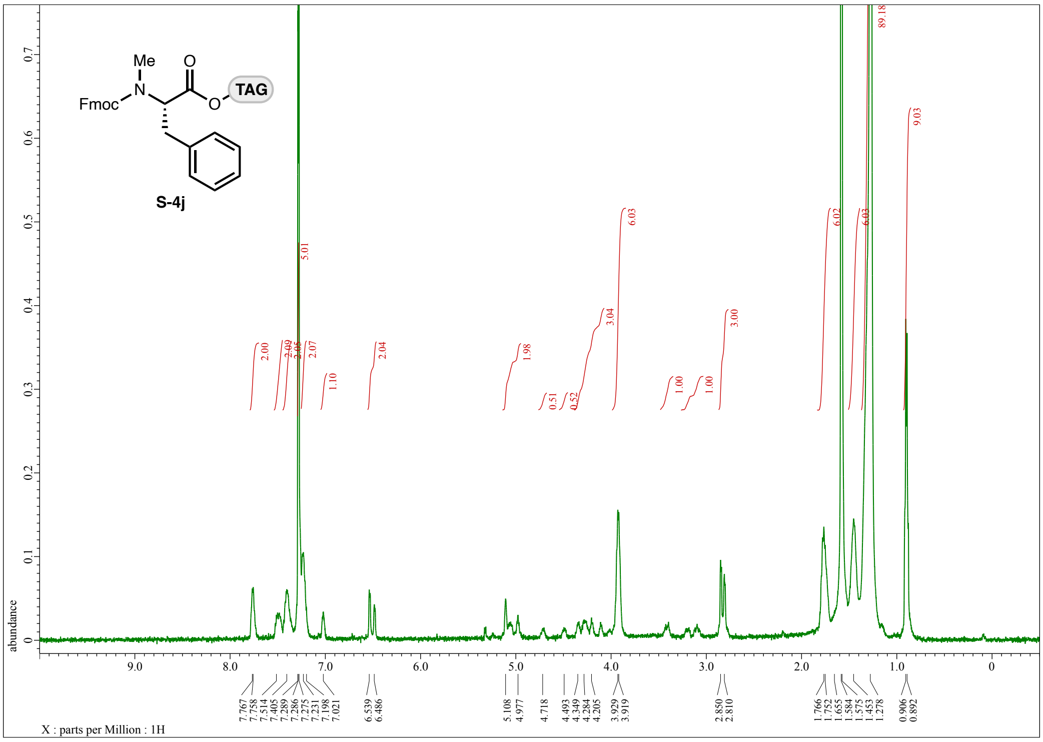

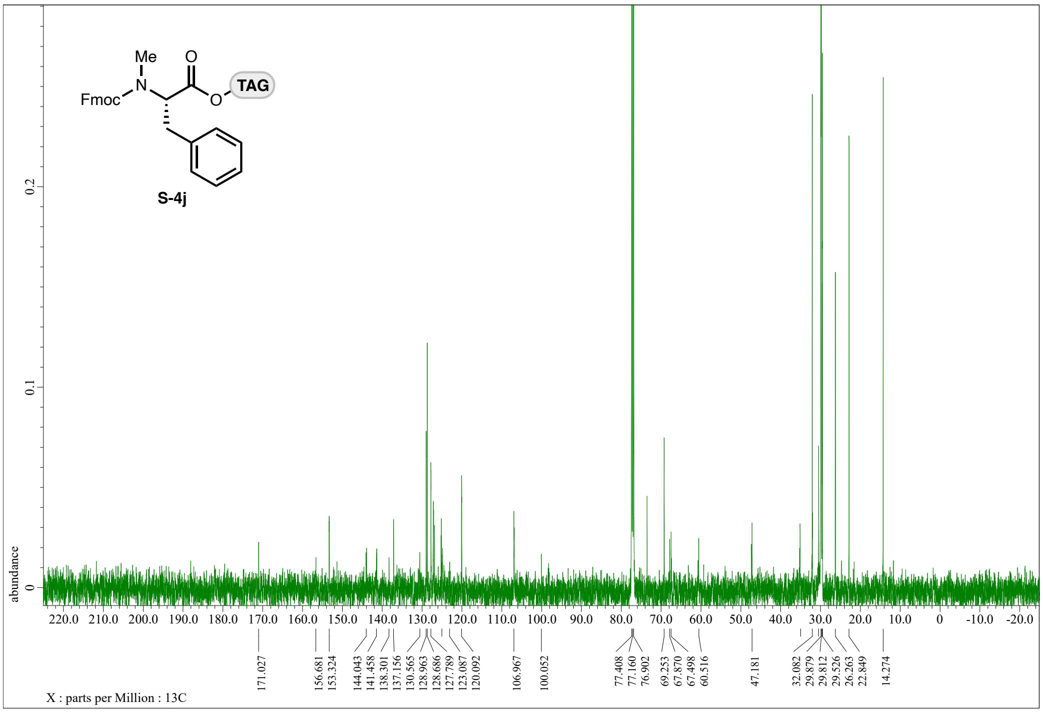

• Fmoc-L-Tyr(^{t}Bu)-OTAG (**S-4k**): ^{1}H NMR (500 MHz, $CDCl_3$), ^{13}C NMR (125 MHz, $CDCl_3$)

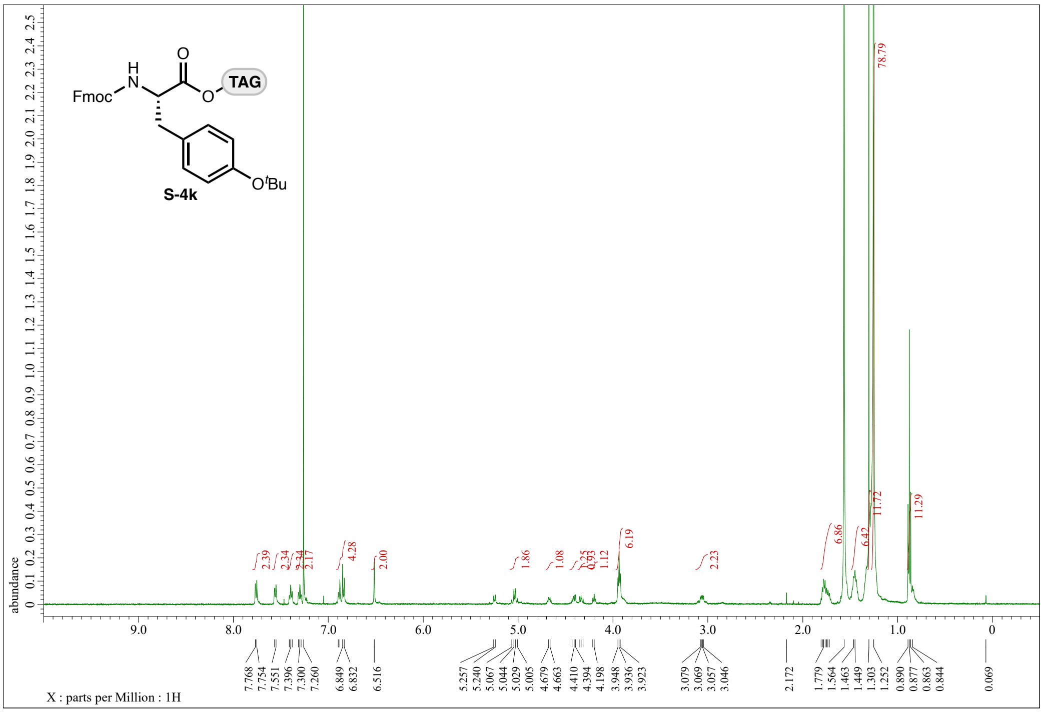

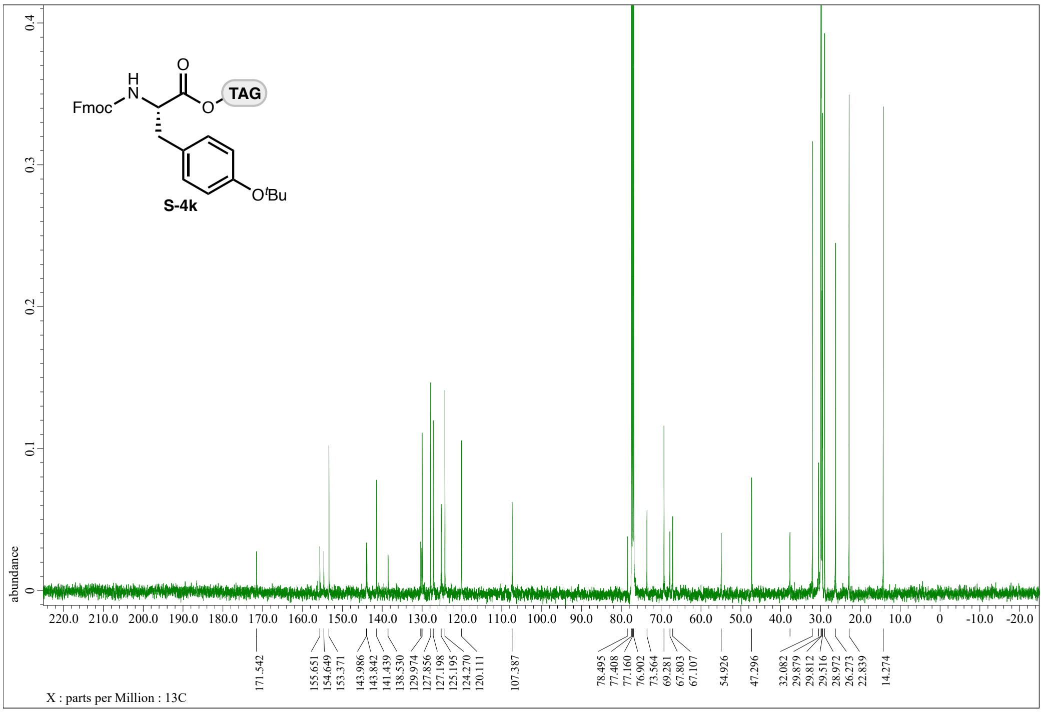

• Fmoc-L-4-F-Phe-OTAG (**S-4l**): ^{1}H NMR (500 MHz, $CDCl_3$), ^{13}C NMR (125 MHz, $CDCl_3$)

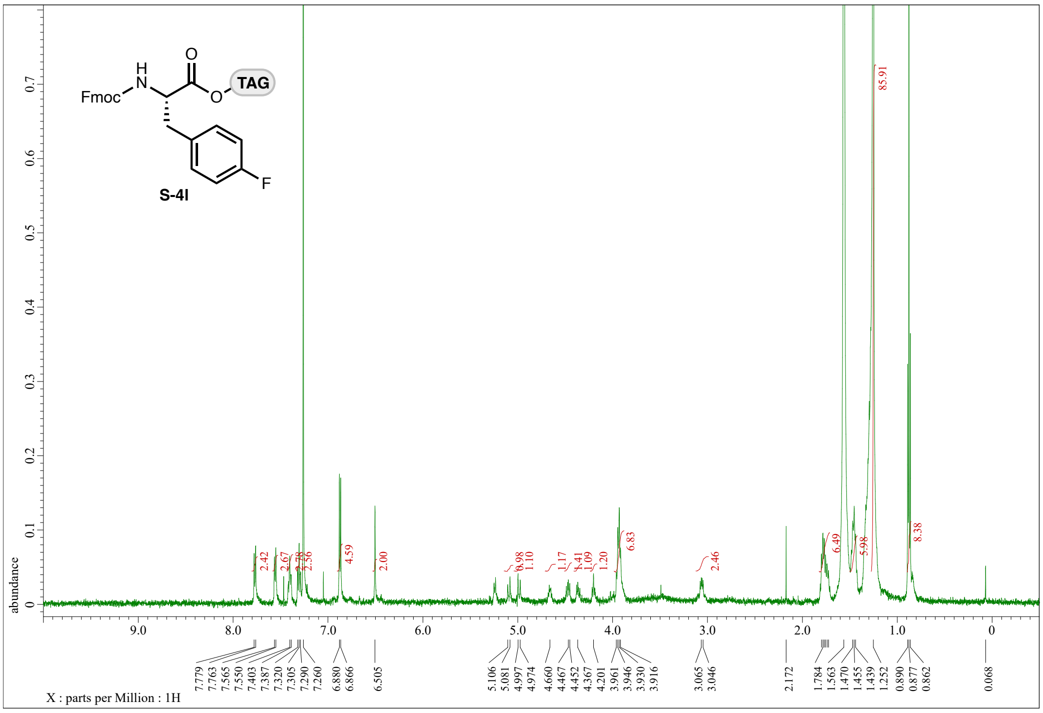

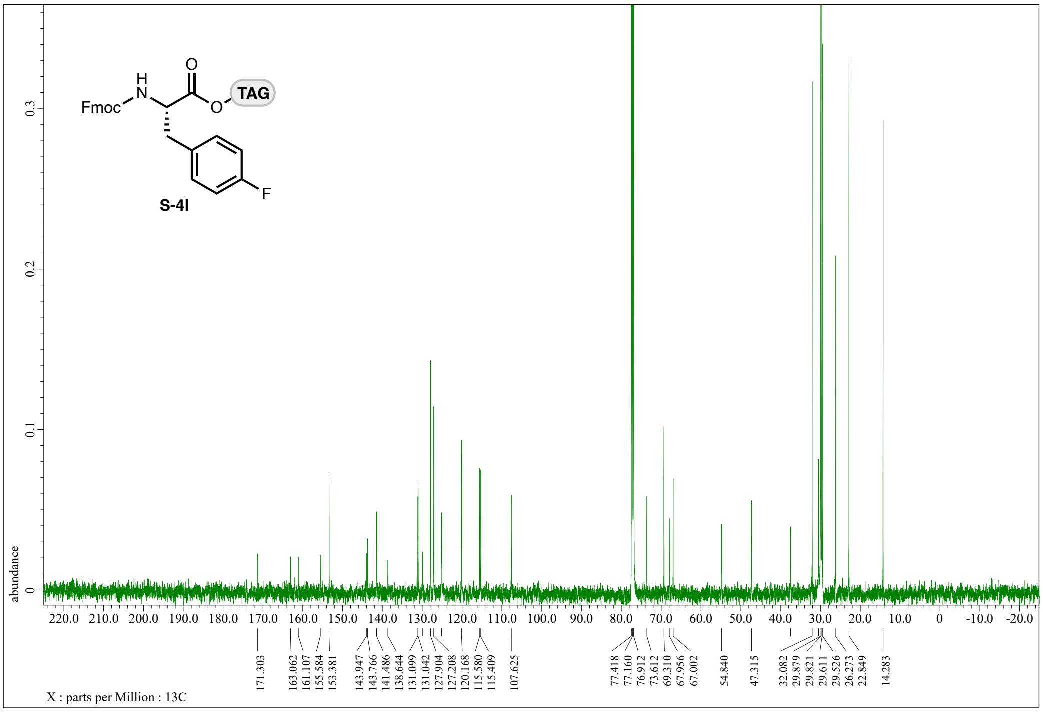

• Fmoc-L-Trp(Boc)-OTAG (**S-4m**): ^{1}H NMR (500 MHz, $CDCl_3$), ^{13}C NMR (125 MHz, $CDCl_3$)

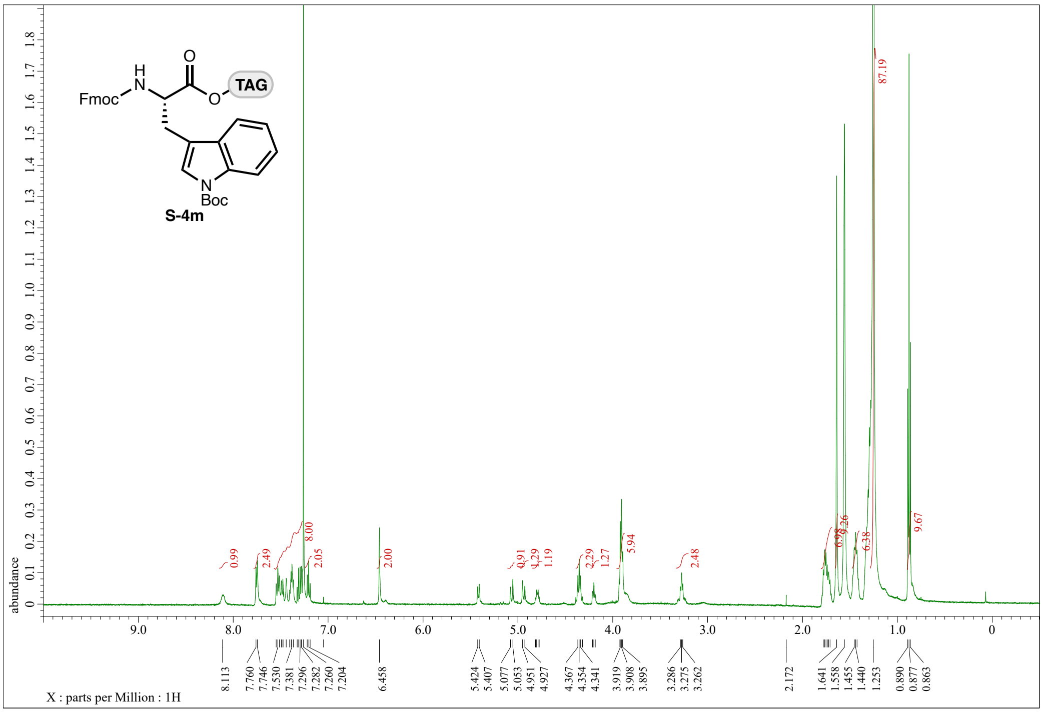

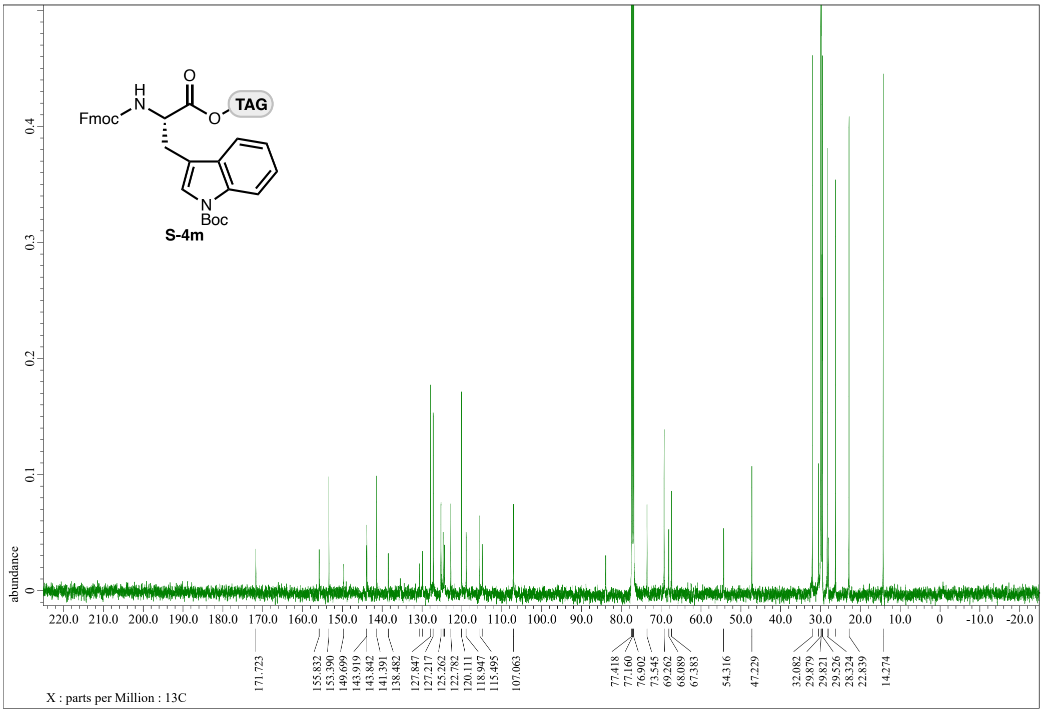

• H-D-Val-OTAG (**18a**): ^{1}H NMR (500 MHz, $CDCl_3$), ^{13}C NMR (125 MHz, $CDCl_3$)

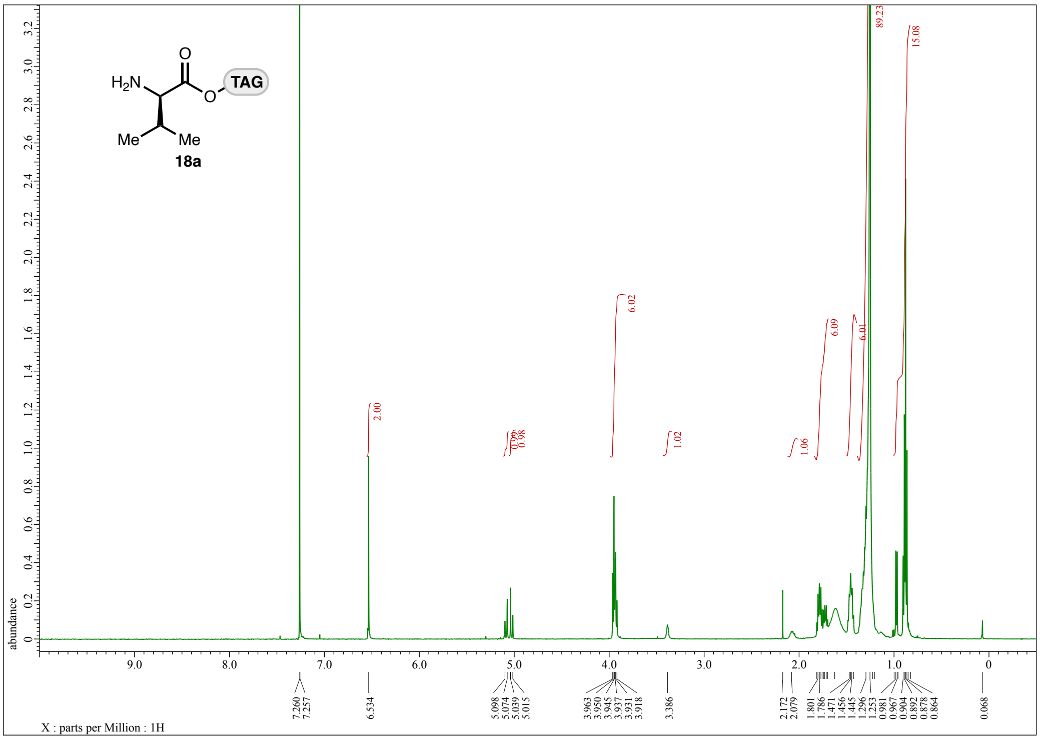

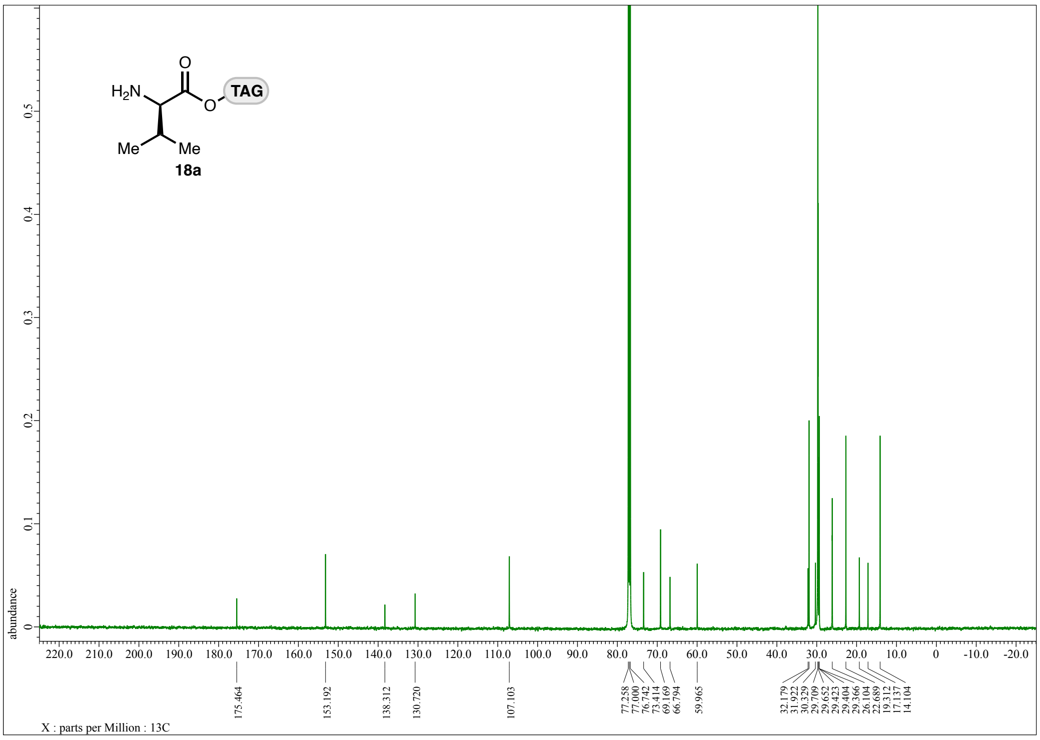

• H-*N*-Me-L-Leu-OTAG (**18c**): ^{1}H NMR (500 MHz, $CDCl_3$), ^{13}C NMR (125 MHz, $CDCl_3$)

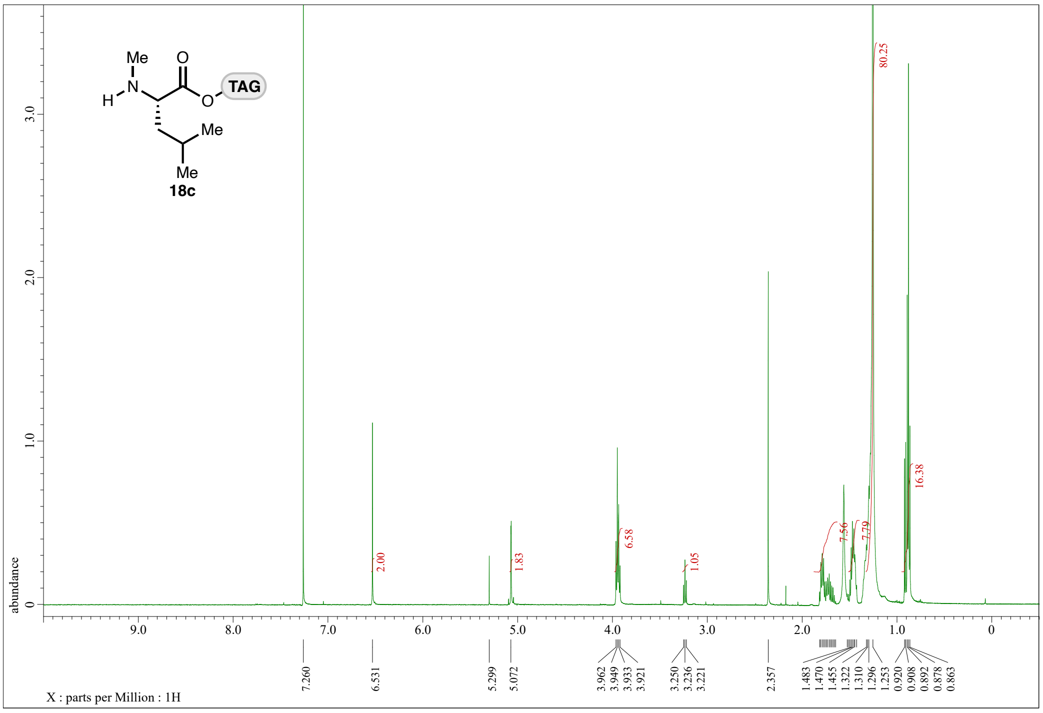

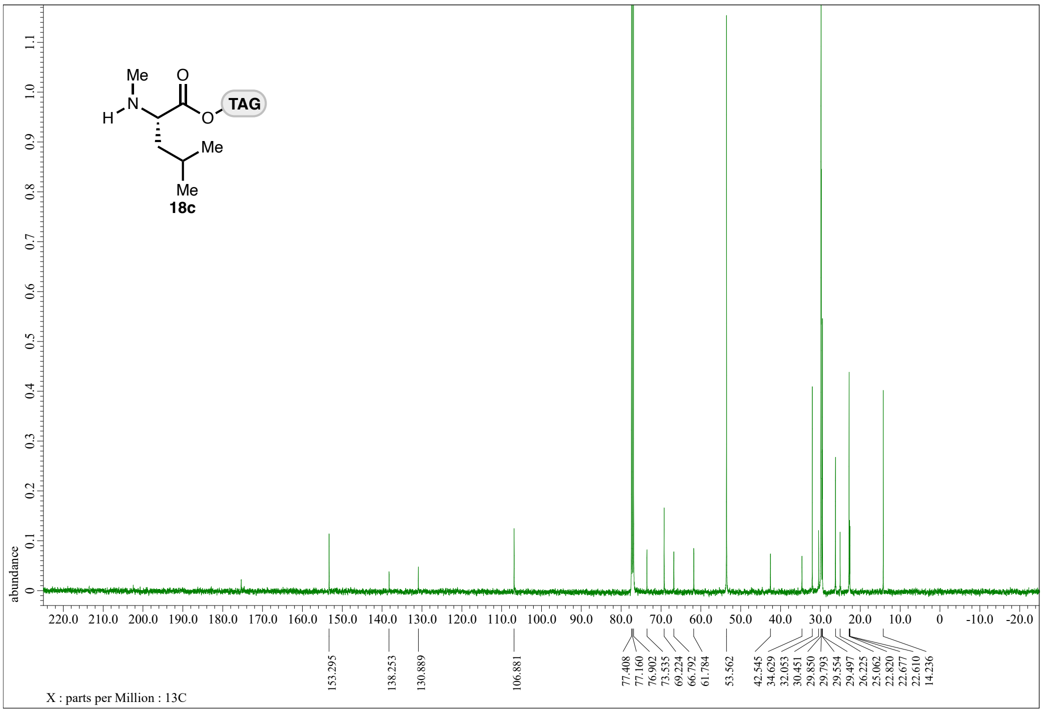

• H-L-ILe-OTAG (**18d**): ^{1}H NMR (500 MHz, $CDCl_3$), ^{13}C NMR (125 MHz, $CDCl_3$)

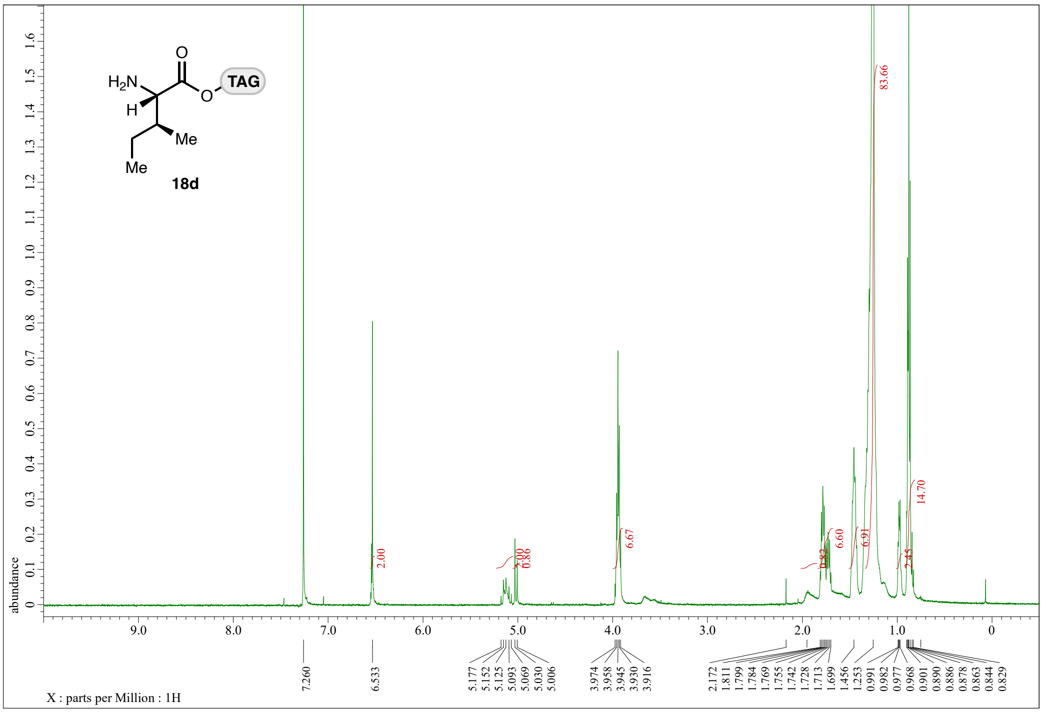

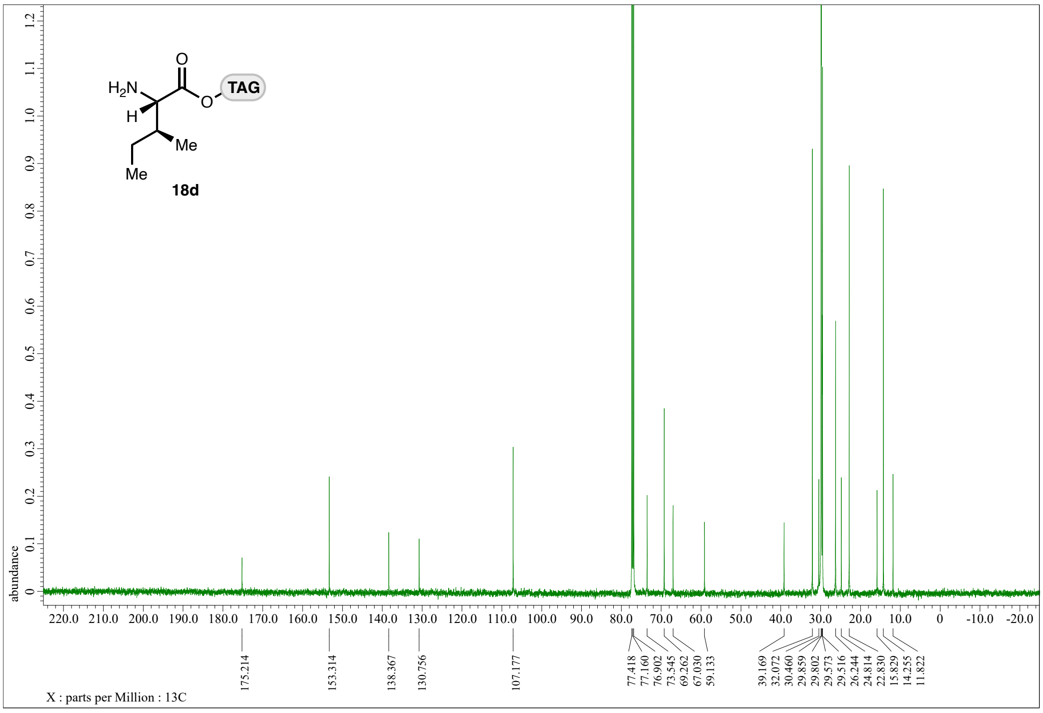

• H-L-Thr(^{t}Bu)-OTAG (**18e**): ^{1}H NMR (500 MHz, $CDCl_3$), ^{13}C NMR (125 MHz, $CDCl_3$)

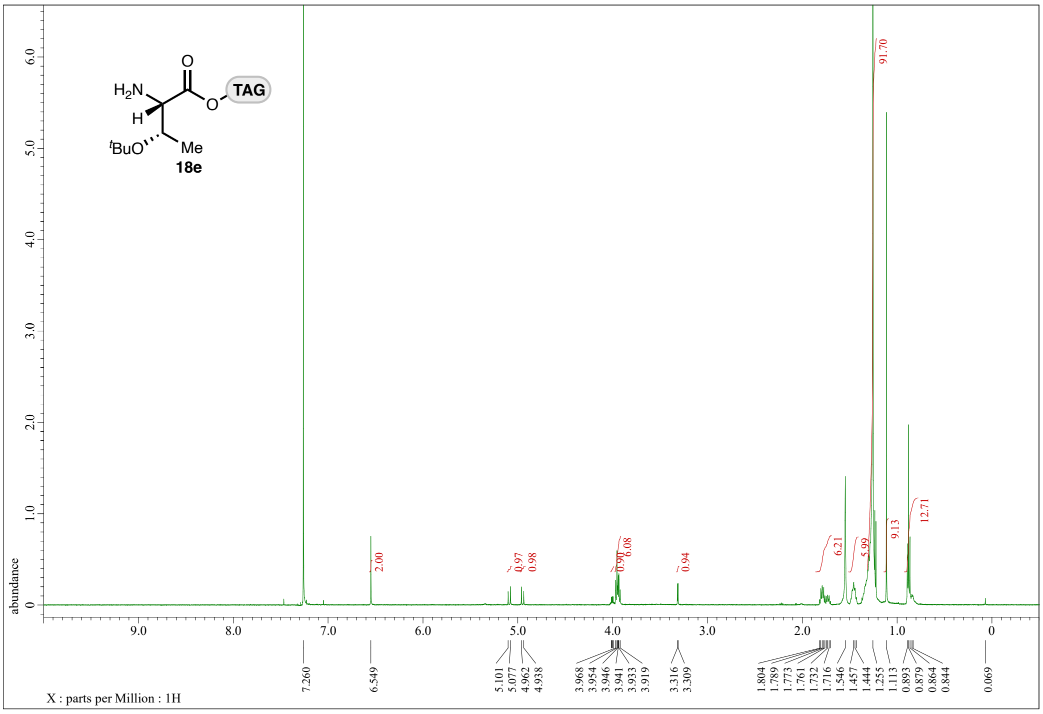

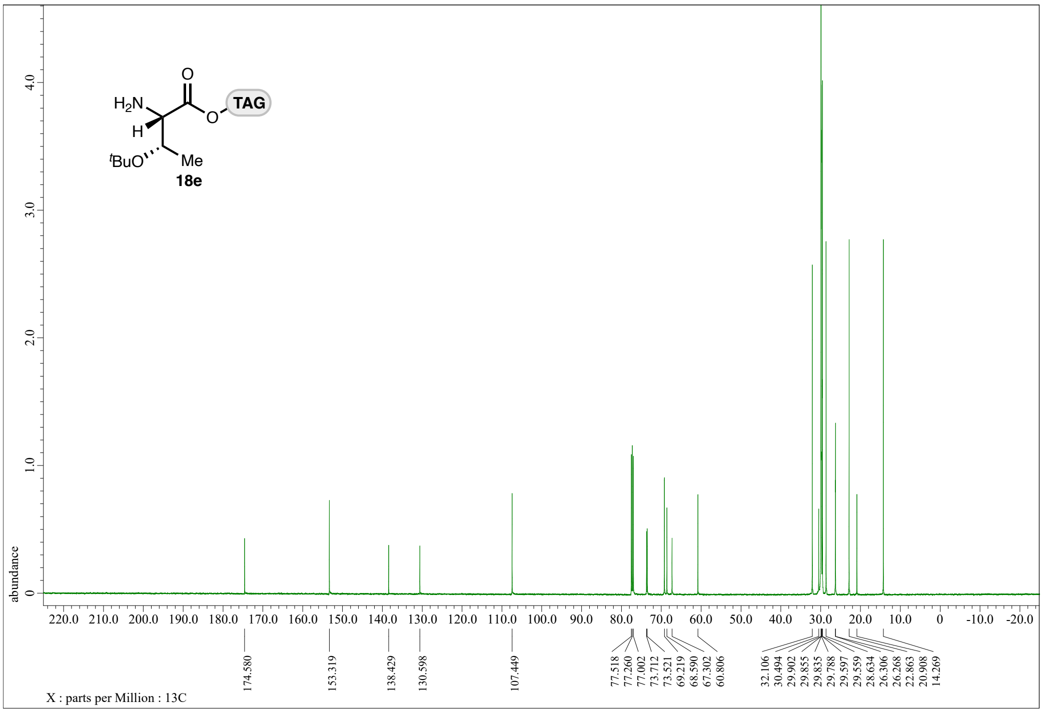

• H-L-Met-OTAG (**18f**): ^{1}H NMR (500 MHz, $CDCl_3$), ^{13}C NMR (125 MHz, $CDCl_3$)

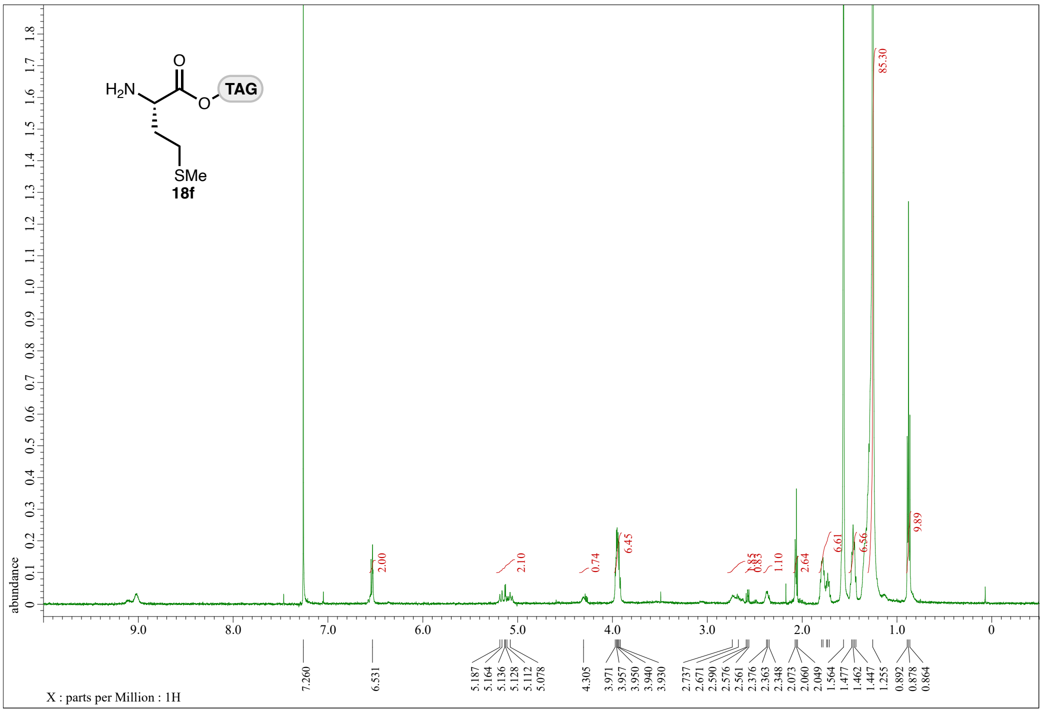

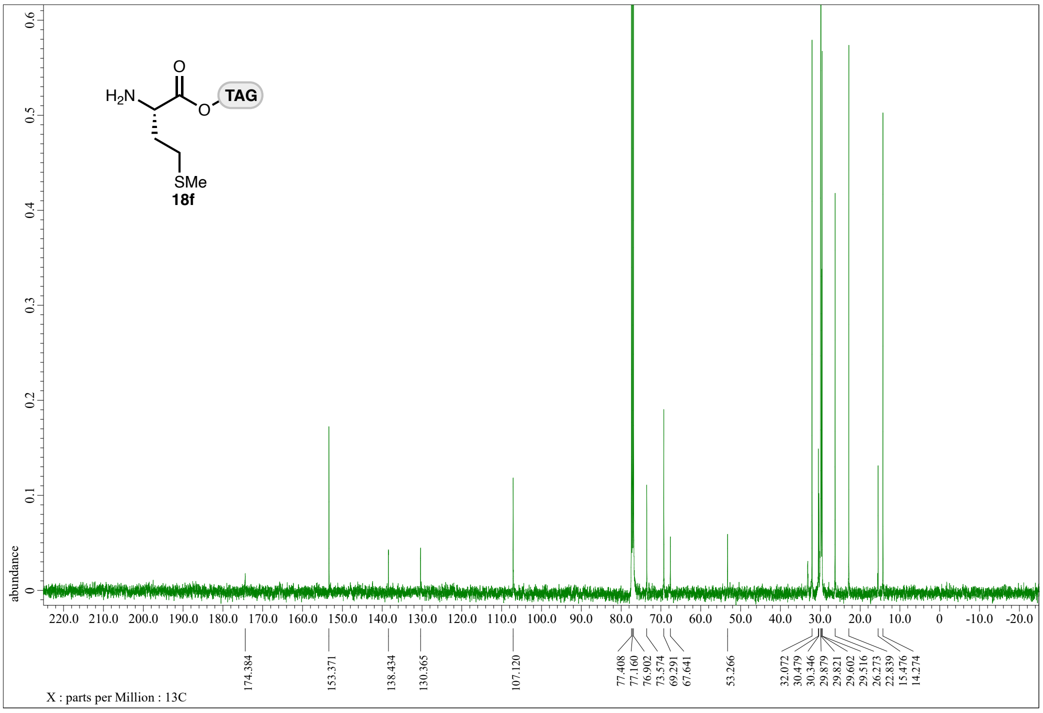

• H-L-Asp(^{t}Bu)-OTAG (**18g**): ^{1}H NMR (500 MHz, $CDCl_3$), ^{13}C NMR (125 MHz, $CDCl_3$)

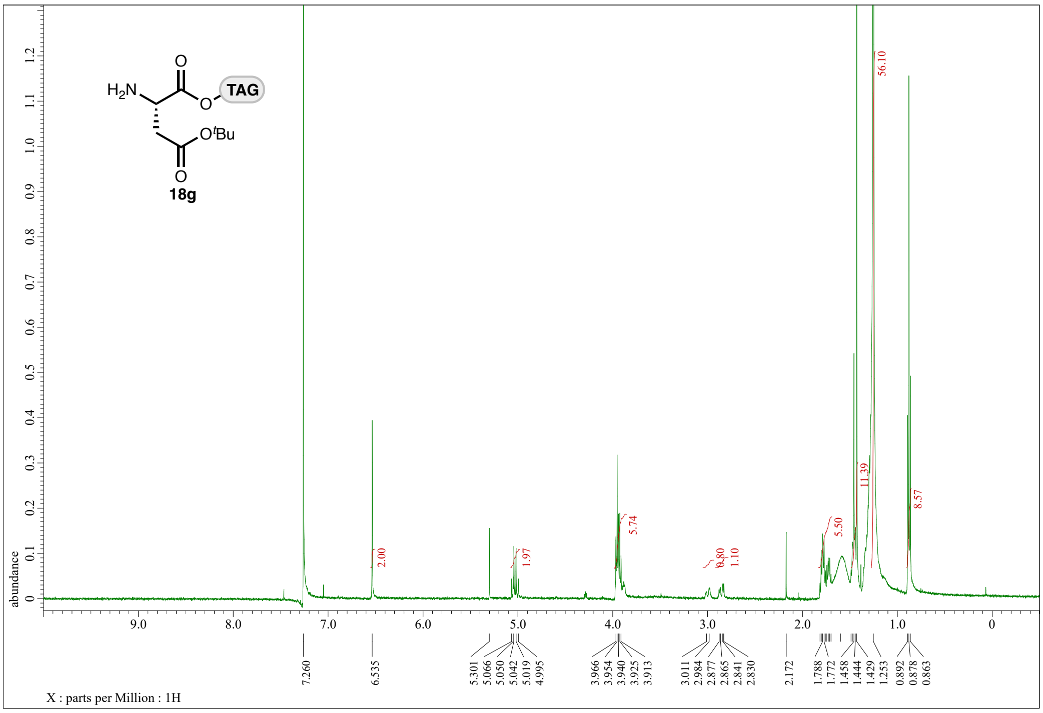

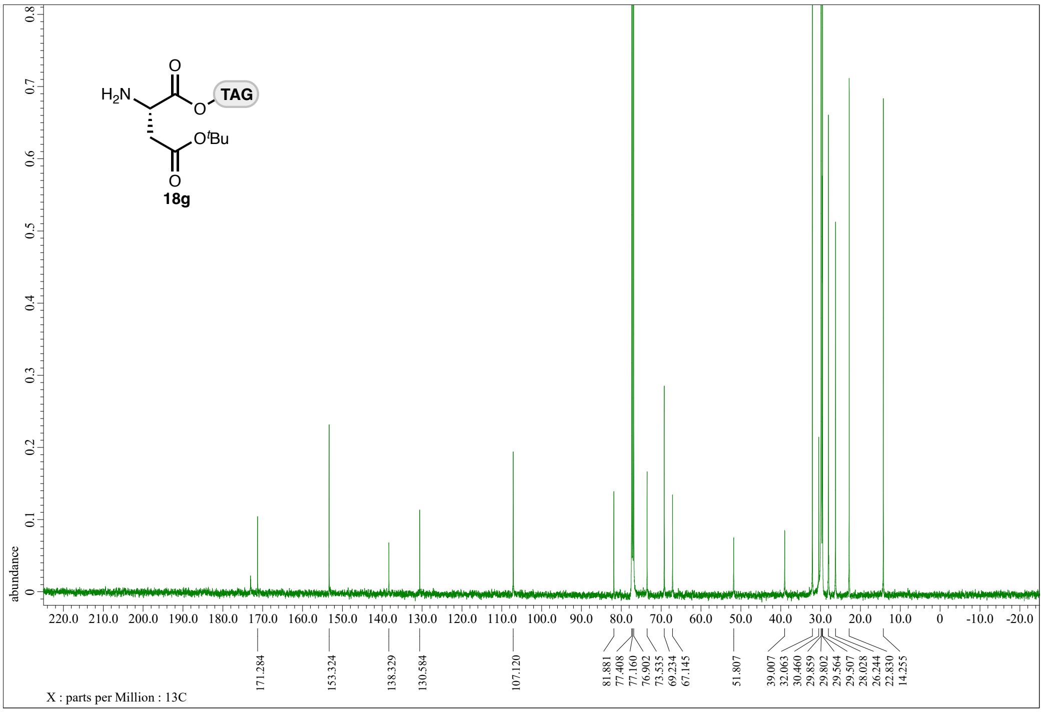

• H-L-Orn(Boc)-OTAG (**18h**): ^{1}H NMR (500 MHz, $CDCl_3$), ^{13}C NMR (125 MHz, $CDCl_3$)

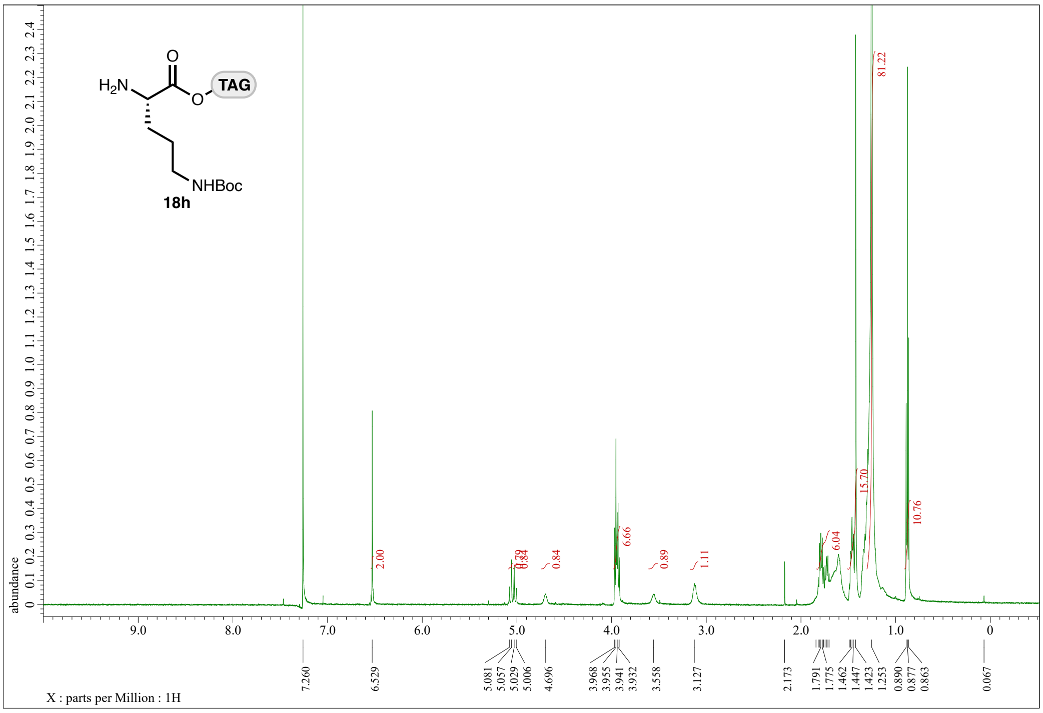

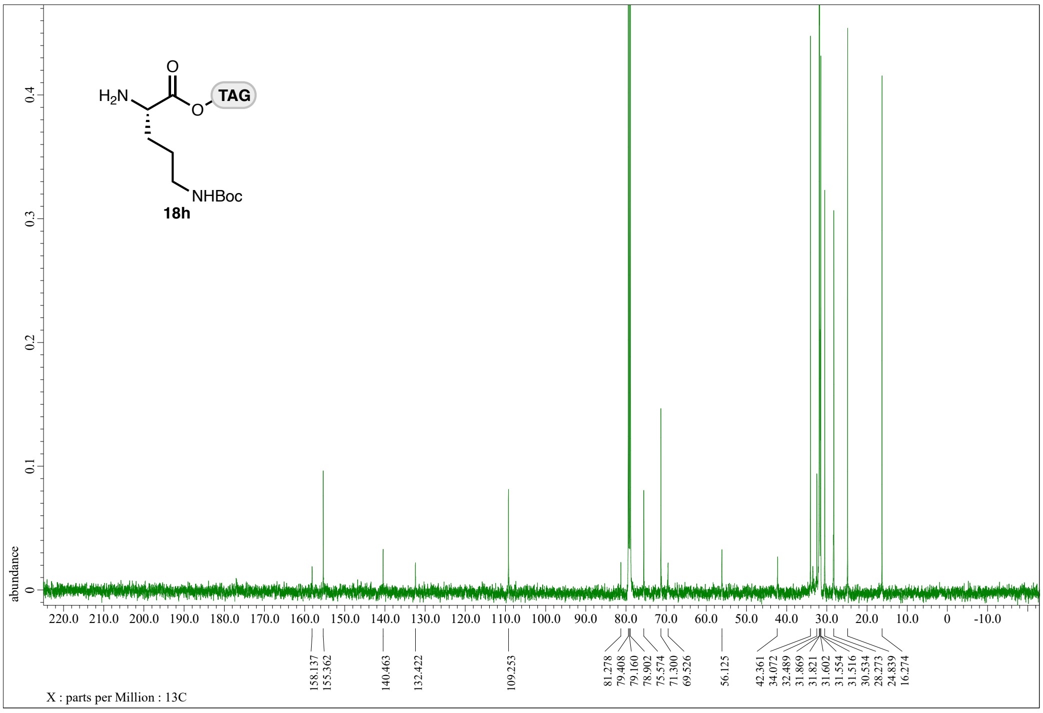

• H-L-His(Boc)-OTAG (**18i**): ^{1}H NMR (500 MHz, $CDCl_3$), ^{13}C NMR (125 MHz, $CDCl_3$)

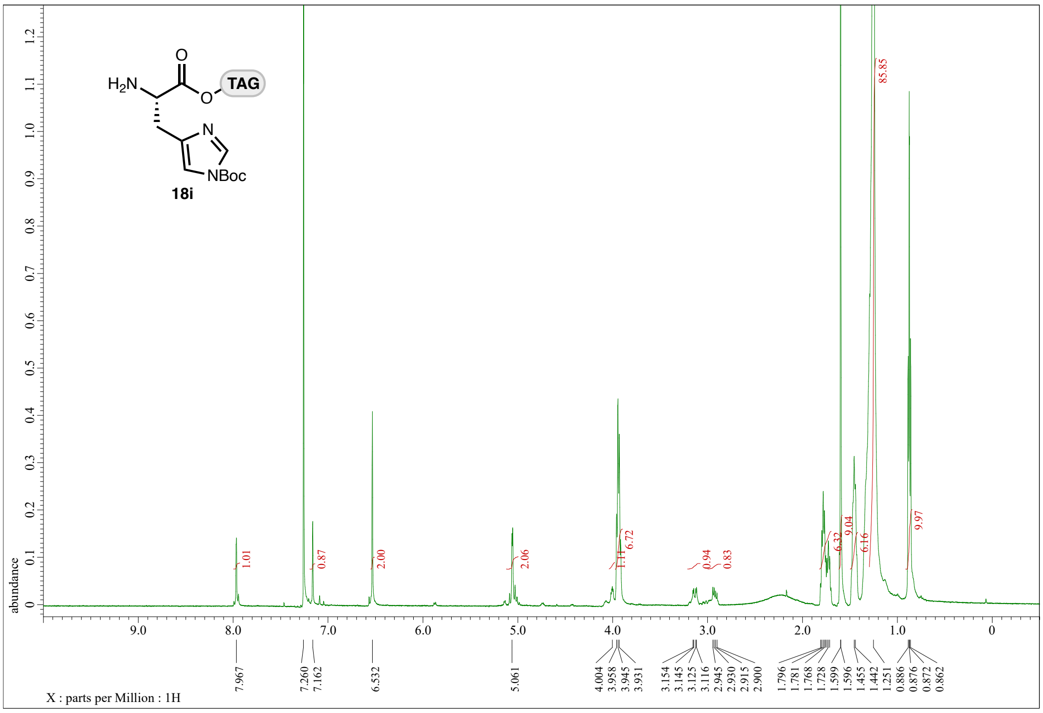

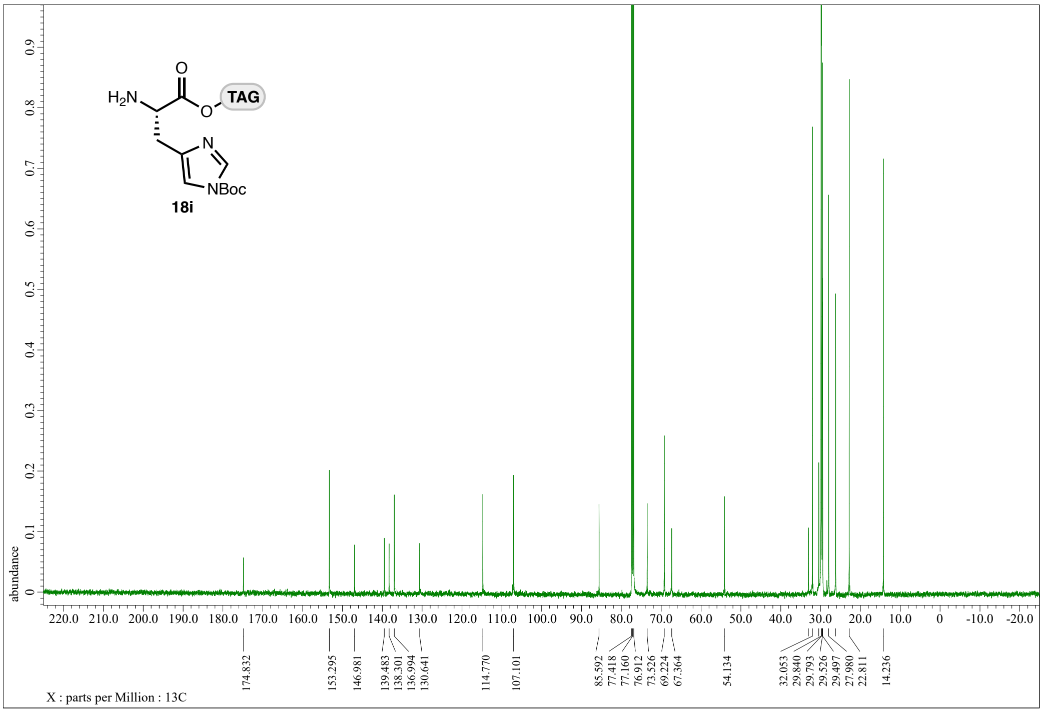

• H-*N*-Me-L-Phe-OTAG (**18j**): ^{1}H NMR (500 MHz, CDCl$_3$), ^{13}C NMR (125 MHz, CDCl$_3$)

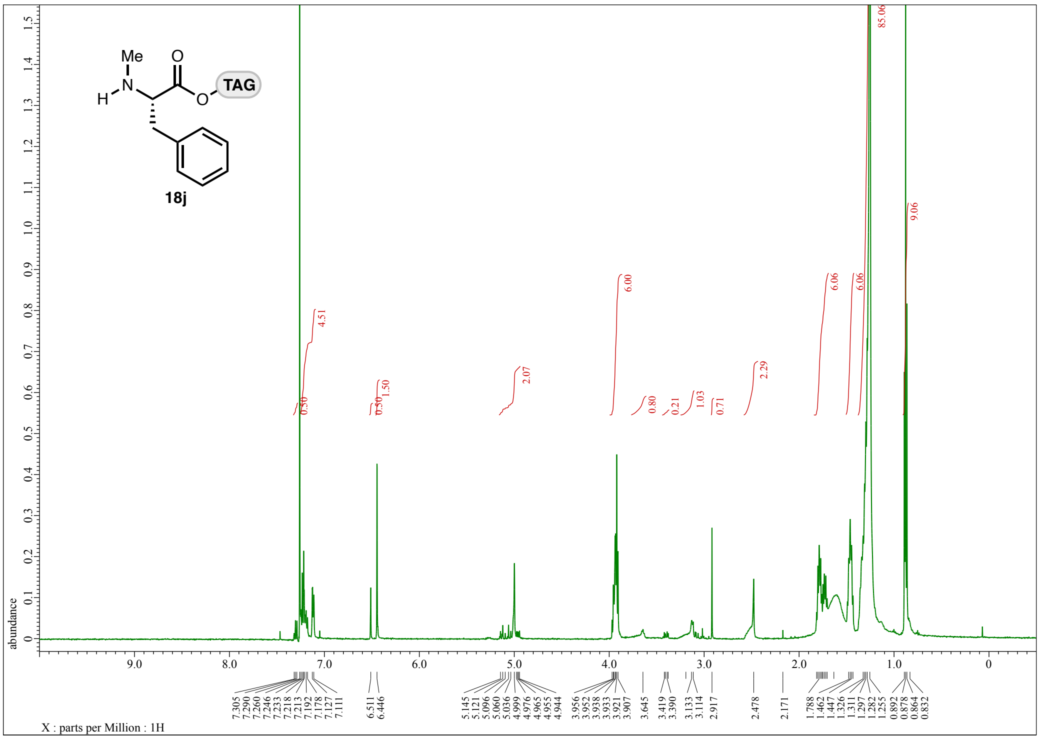

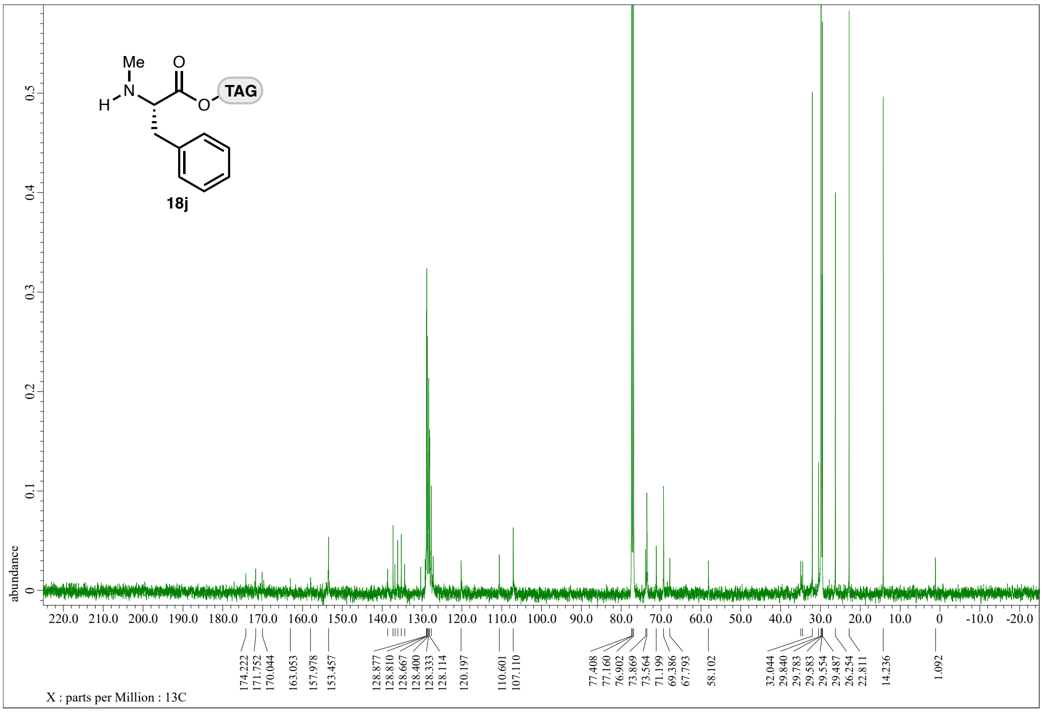

• H-L-Tyr(^{t}Bu)-OTAG (**18k**): ^{1}H NMR (500 MHz, CDCl$_3$), ^{13}C NMR (125 MHz, CDCl$_3$)

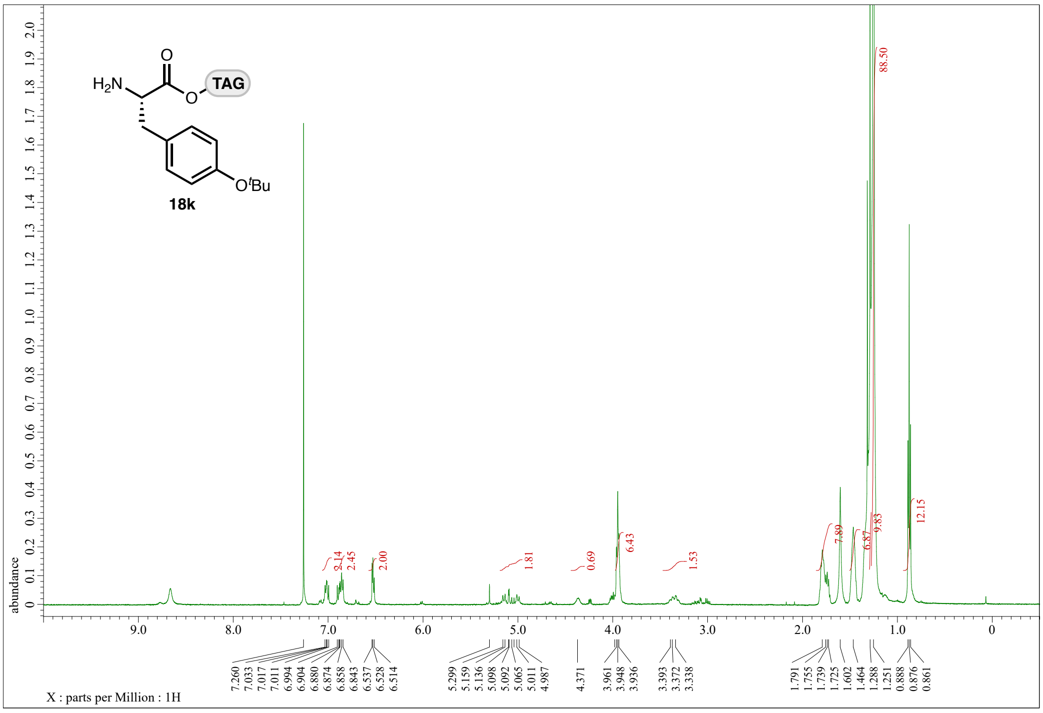

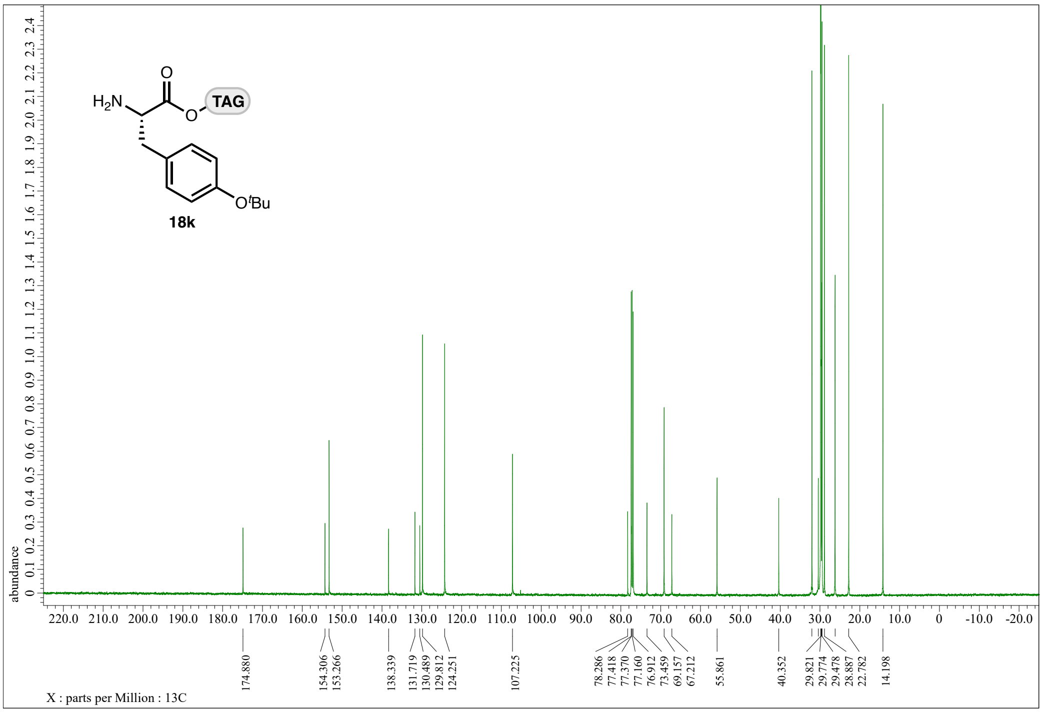

• H-L-4-F-Phe-OTAG (**18l**): ^{1}H NMR (500 MHz, $CDCl_3$), ^{13}C NMR (125 MHz, $CDCl_3$)

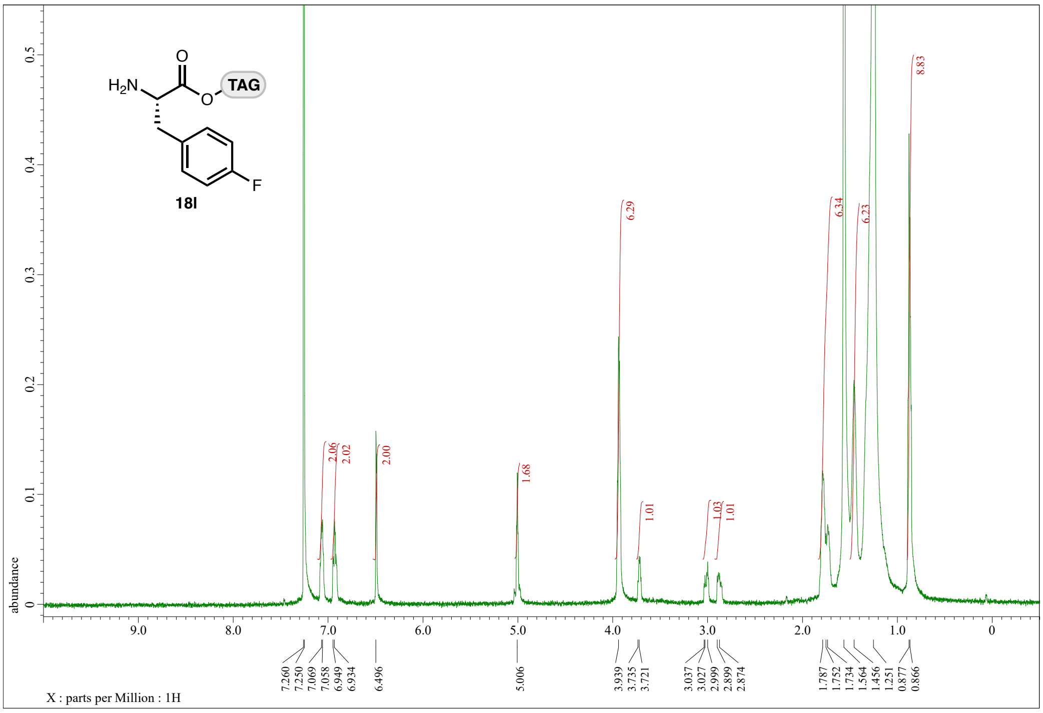

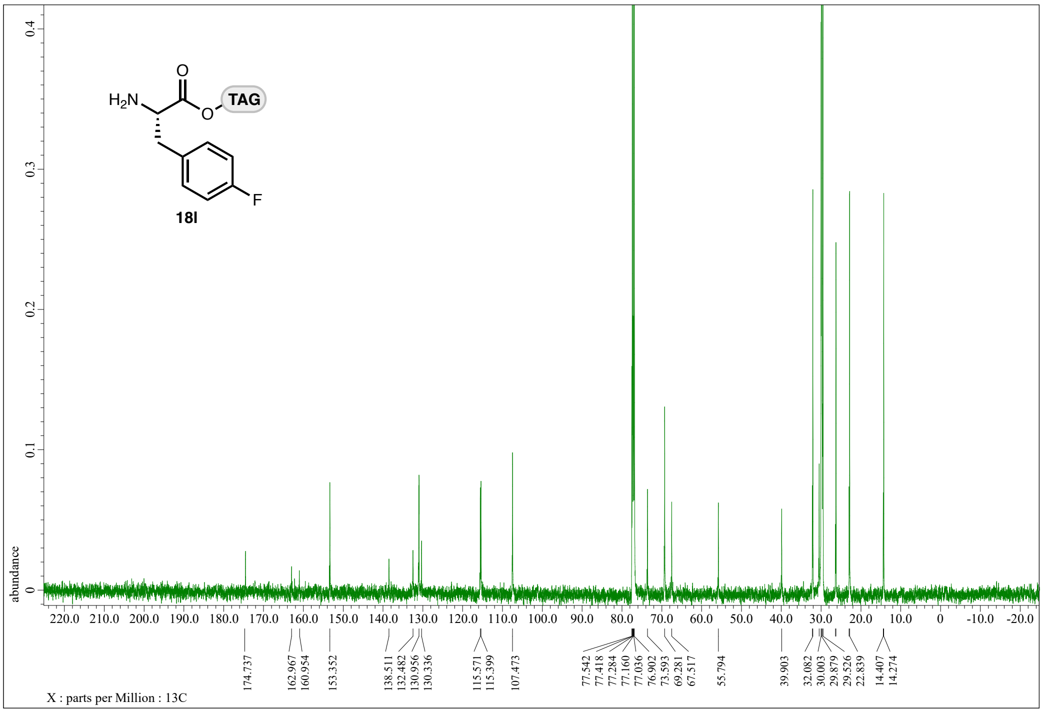

• H-L-Trp(Boc)-OTAG (**18m**): ^{1}H NMR (500 MHz, $CDCl_3$), ^{13}C NMR (125 MHz, $CDCl_3$)

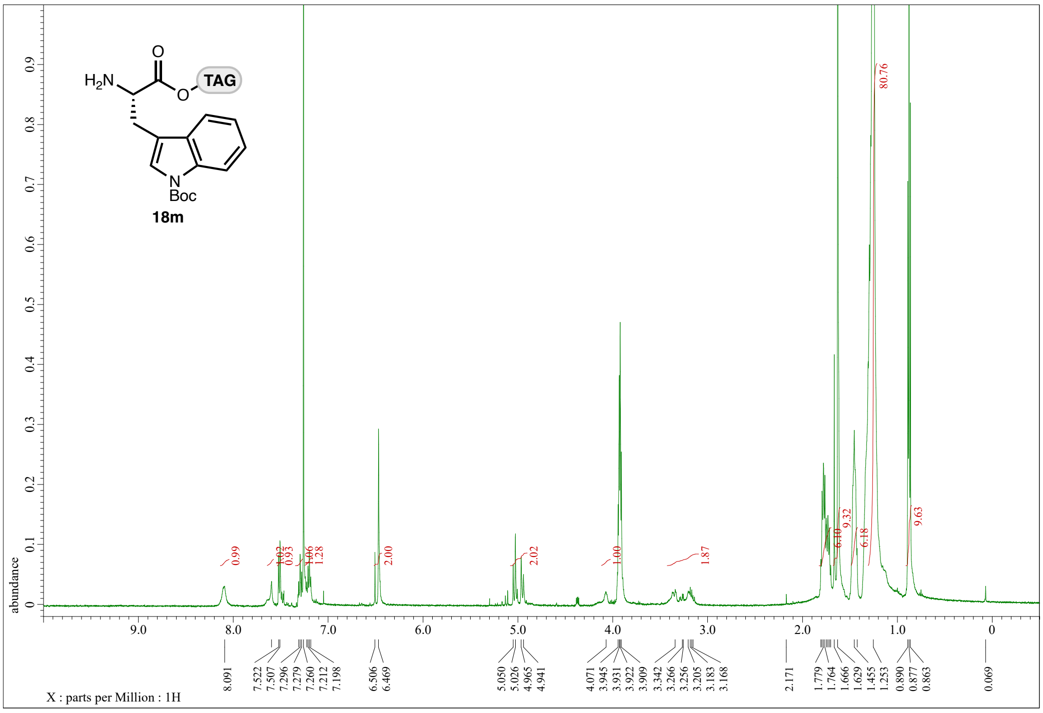

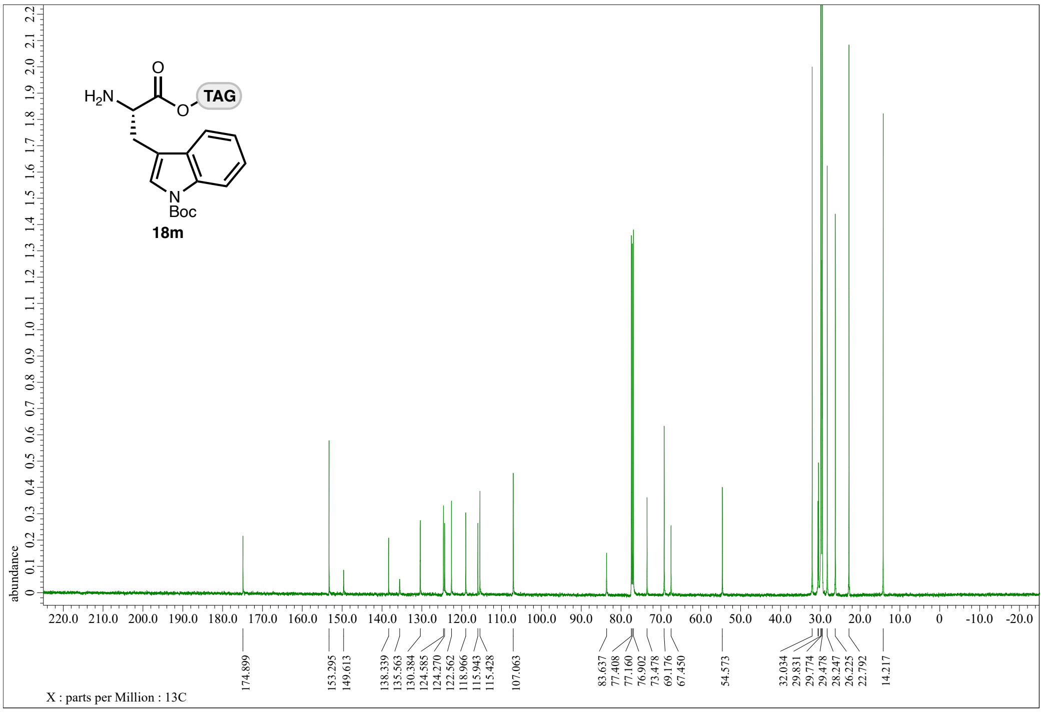

• BnO-tropolone-D-Val-OTAG (**19a**): ^{1}H NMR (500 MHz, $CDCl_3$), ^{13}C NMR (125 MHz, $CDCl_3$)

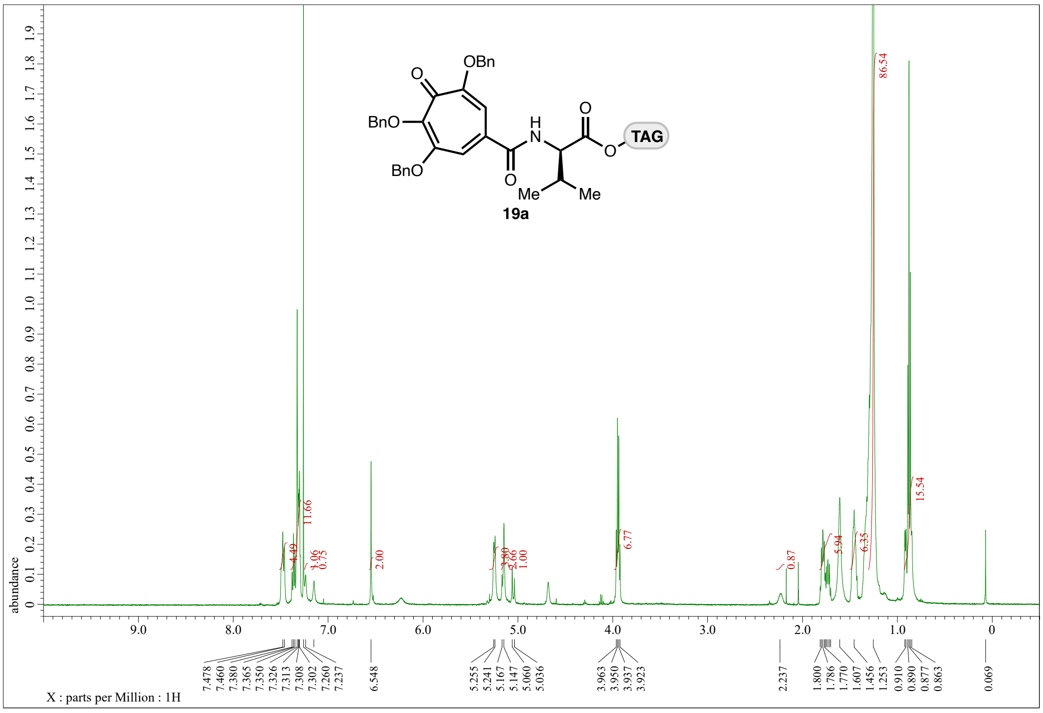

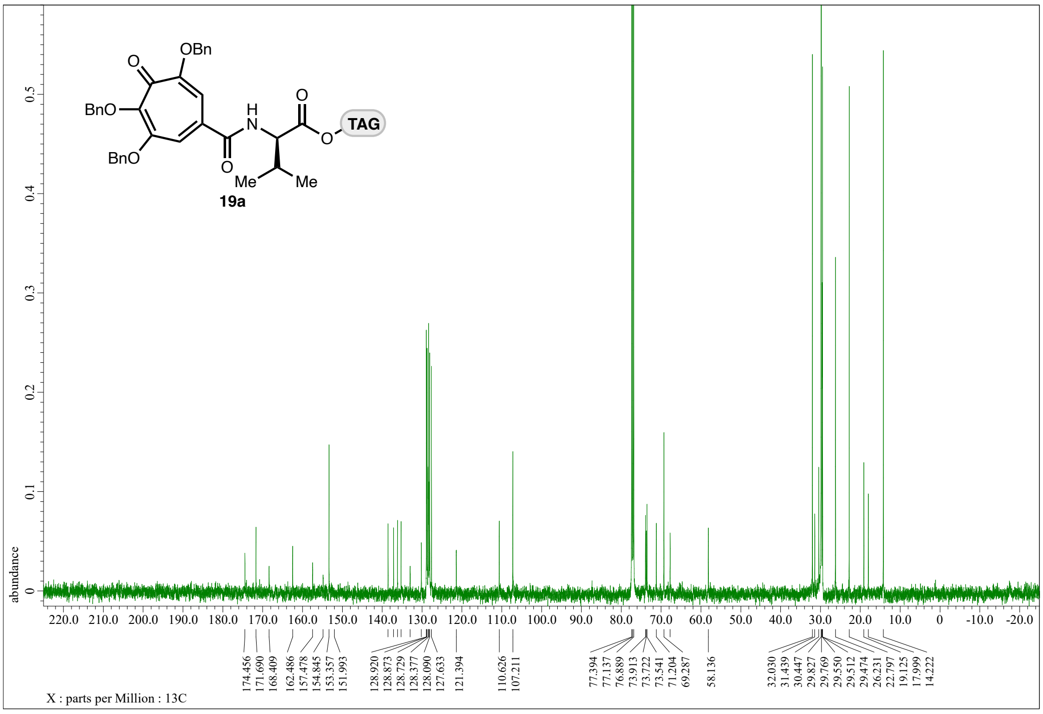

• BnO-tropolone-L-Leu-OTAG (**19b**): ^{1}H NMR (500 MHz, $CDCl_3$), ^{13}C NMR (125 MHz, $CDCl_3$)

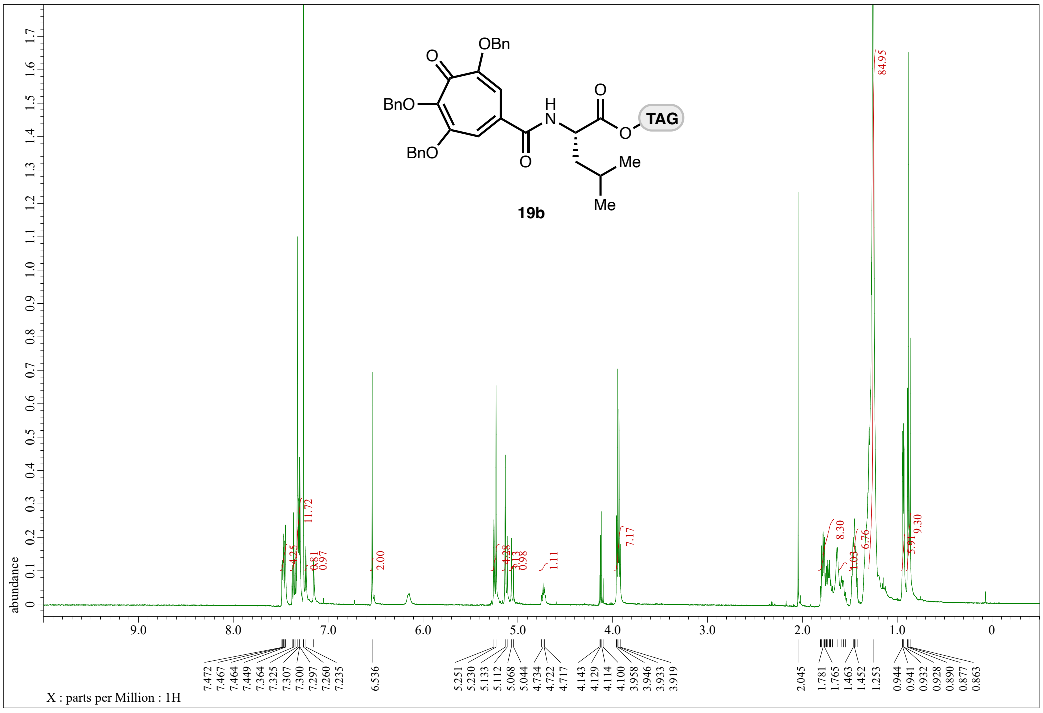

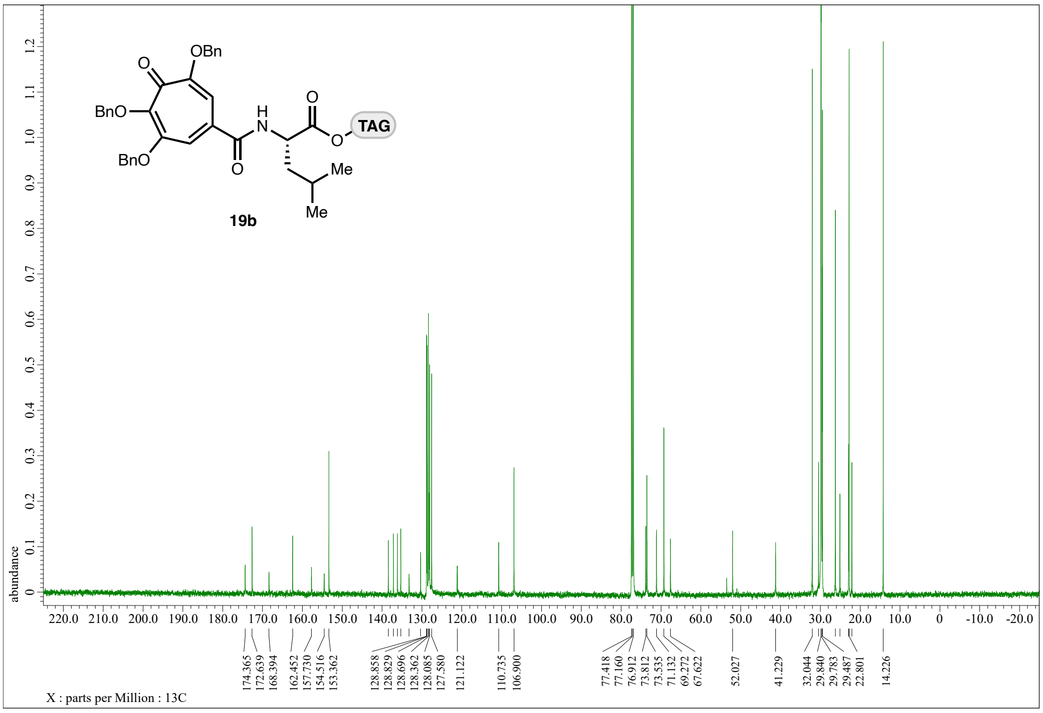

• BnO-tropolone-*N*-Me-L-Leu-OTAG (**19c**): ^{1}H NMR (500 MHz, $CDCl_3$), ^{13}C NMR (125 MHz, $CDCl_3$)

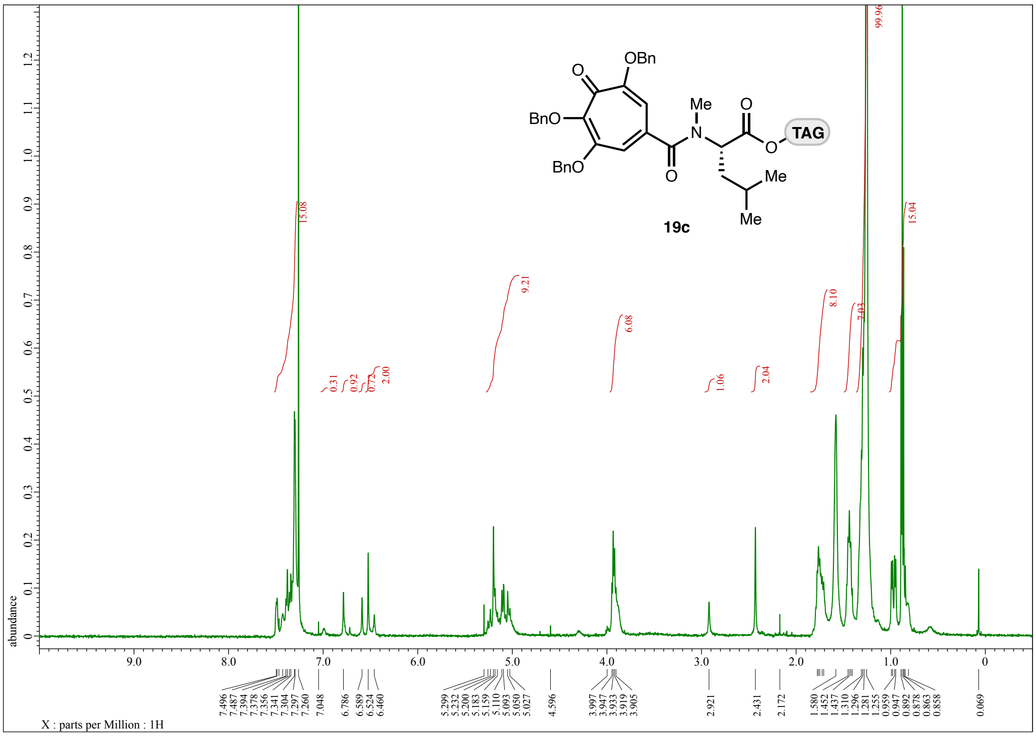

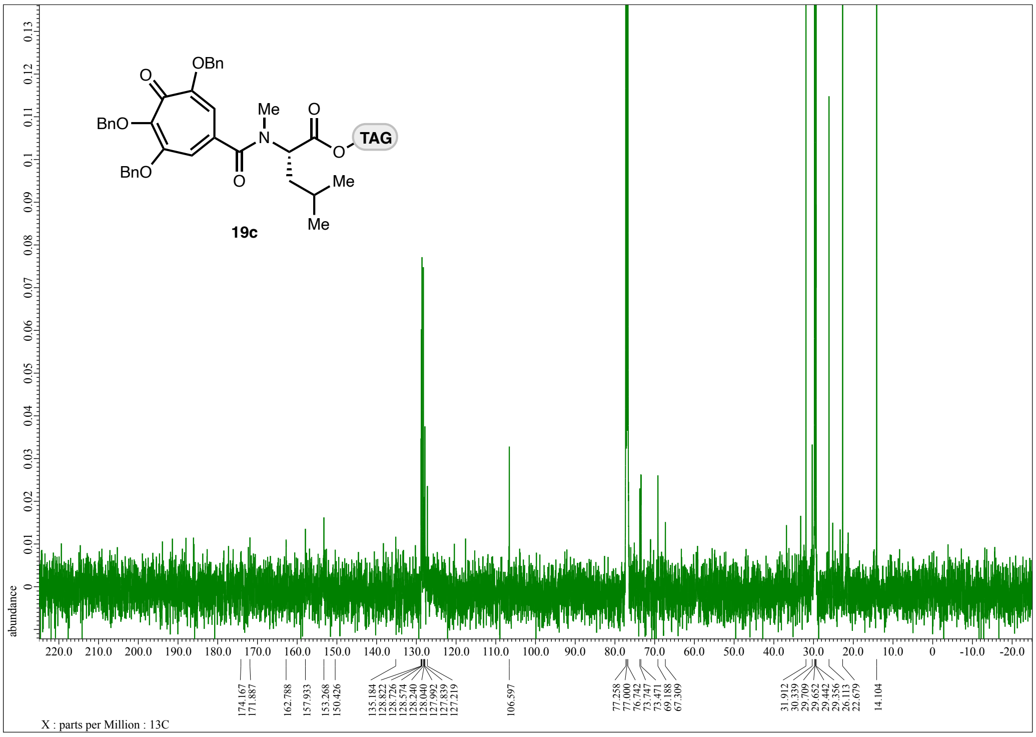

• BnO-tropolone-L-ILe-OTAG (**19d**): ^{1}H NMR (500 MHz, CDCl$_3$), ^{13}C NMR (125 MHz, CDCl$_3$)

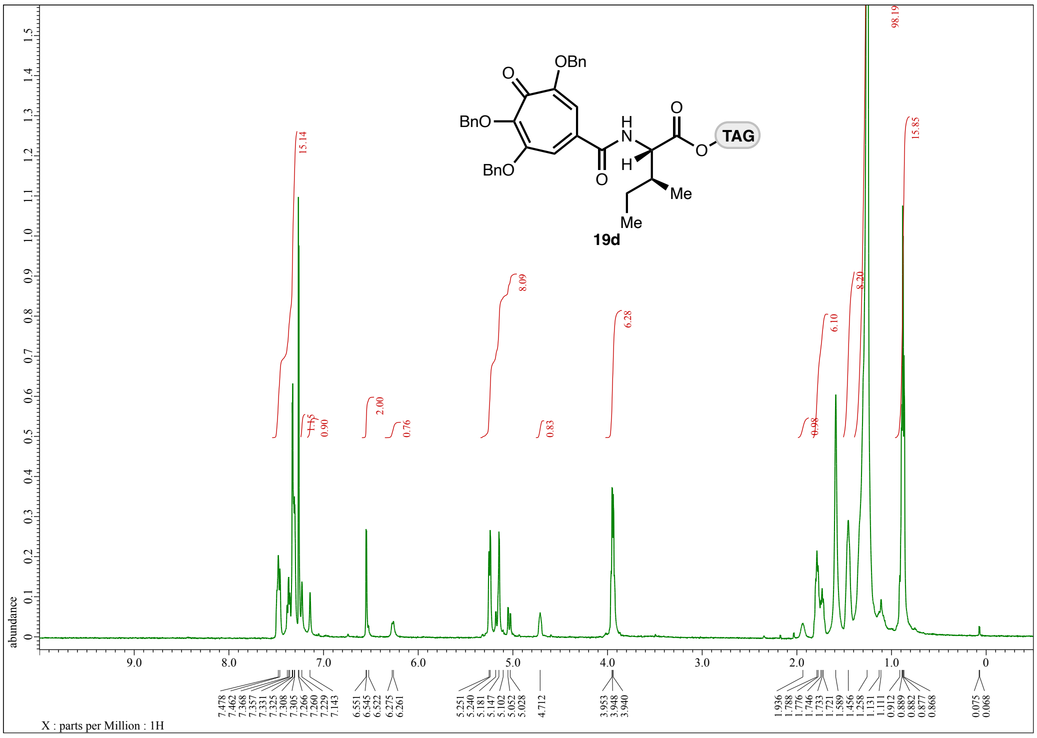

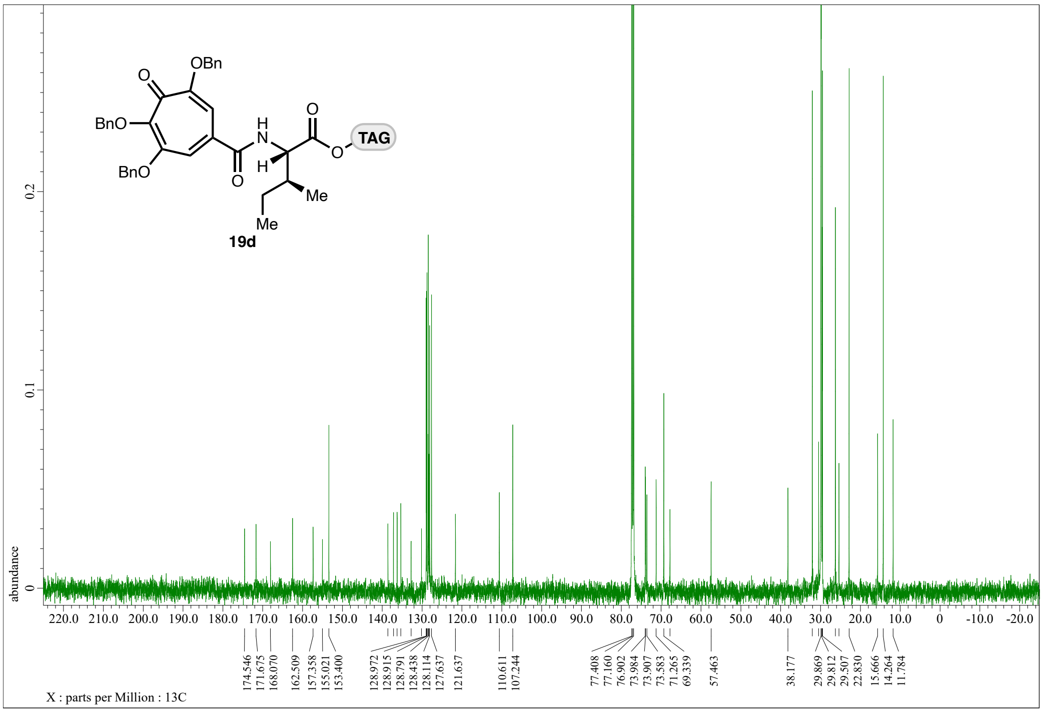

• BnO-tropolone-L-Thr(^{t}Bu)-OTAG (**19e**): ^{1}H NMR (500 MHz, $CDCl_3$), ^{13}C NMR (125 MHz, $CDCl_3$)

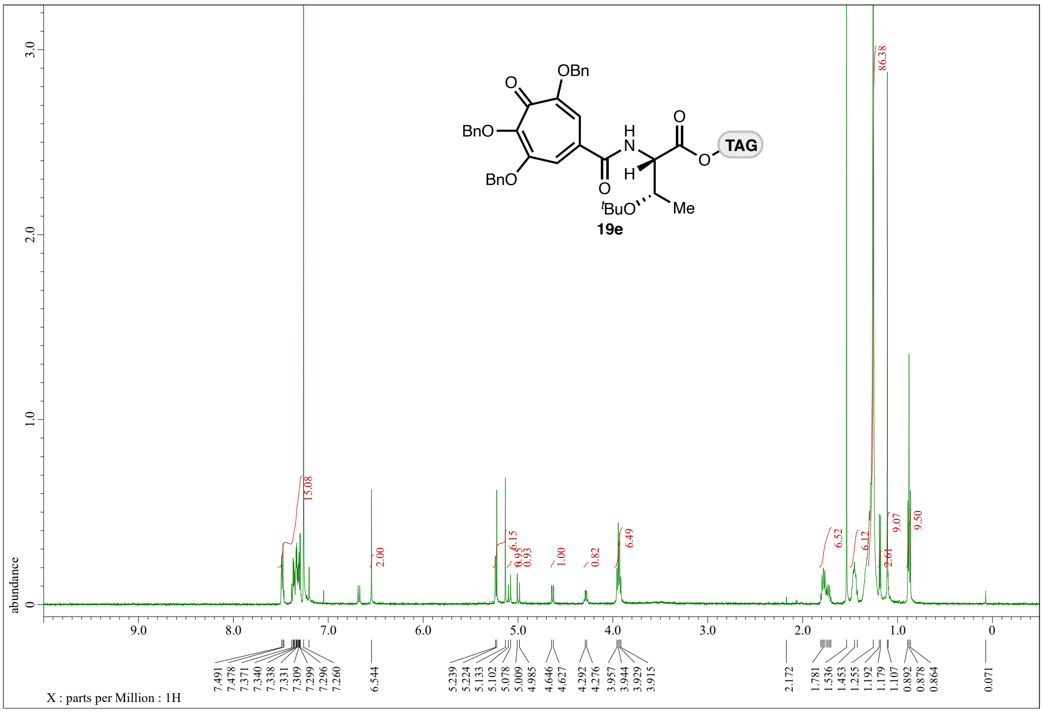

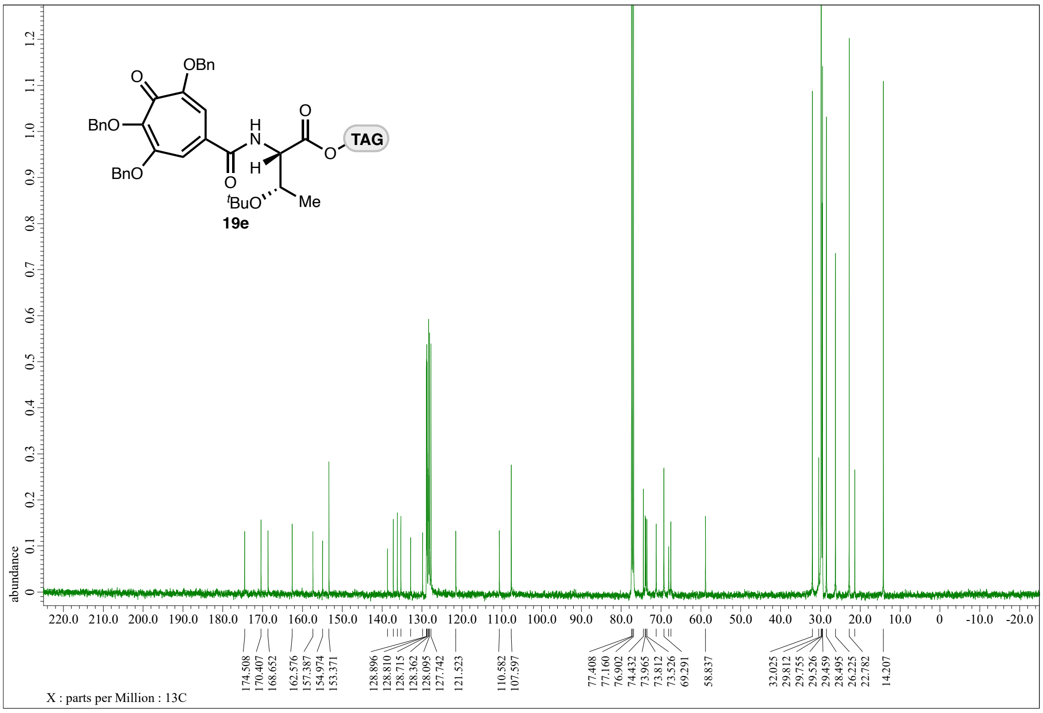

• BnO-tropolone-L-Met-OTAG (**19f**): ^{1}H NMR (500 MHz, $CDCl_3$), ^{13}C NMR (125 MHz, $CDCl_3$)

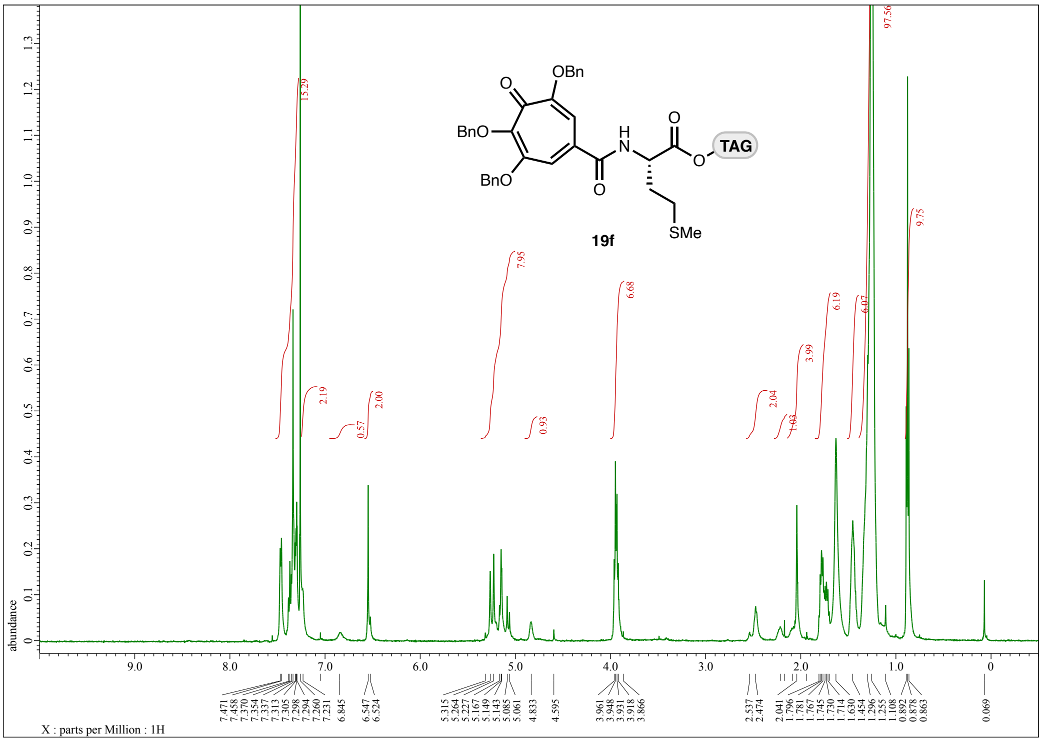

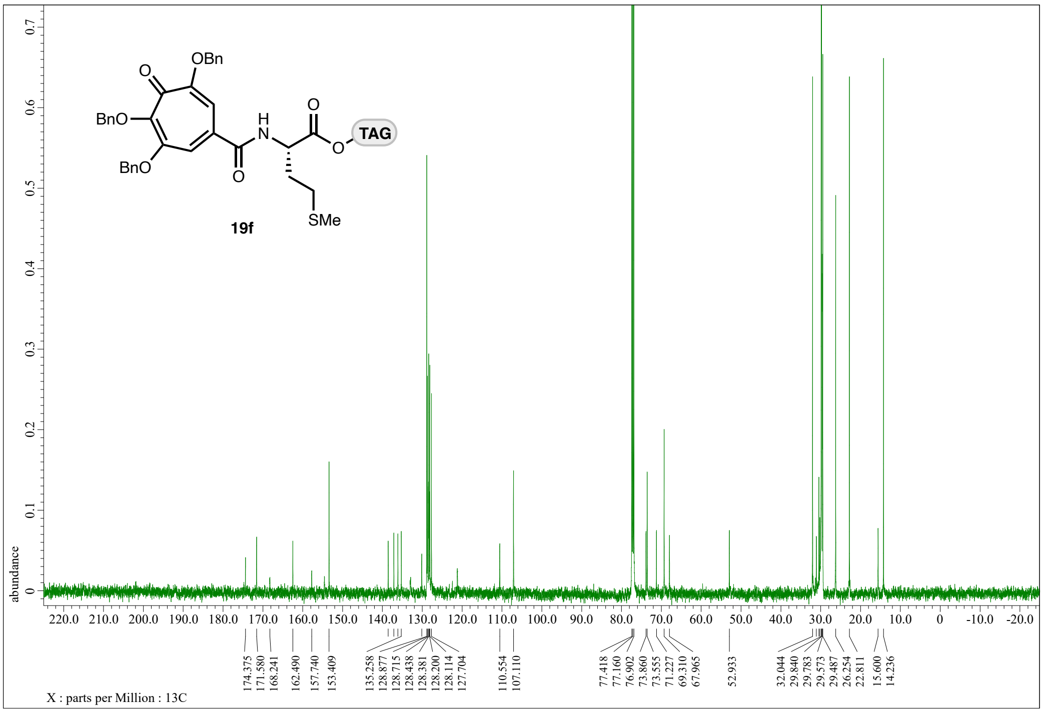

• BnO-tropolone-L-Asp(^{t}Bu)-OTAG (**19g**): ^{1}H NMR (500 MHz, CDCl$_3$), ^{13}C NMR (125 MHz, CDCl$_3$)

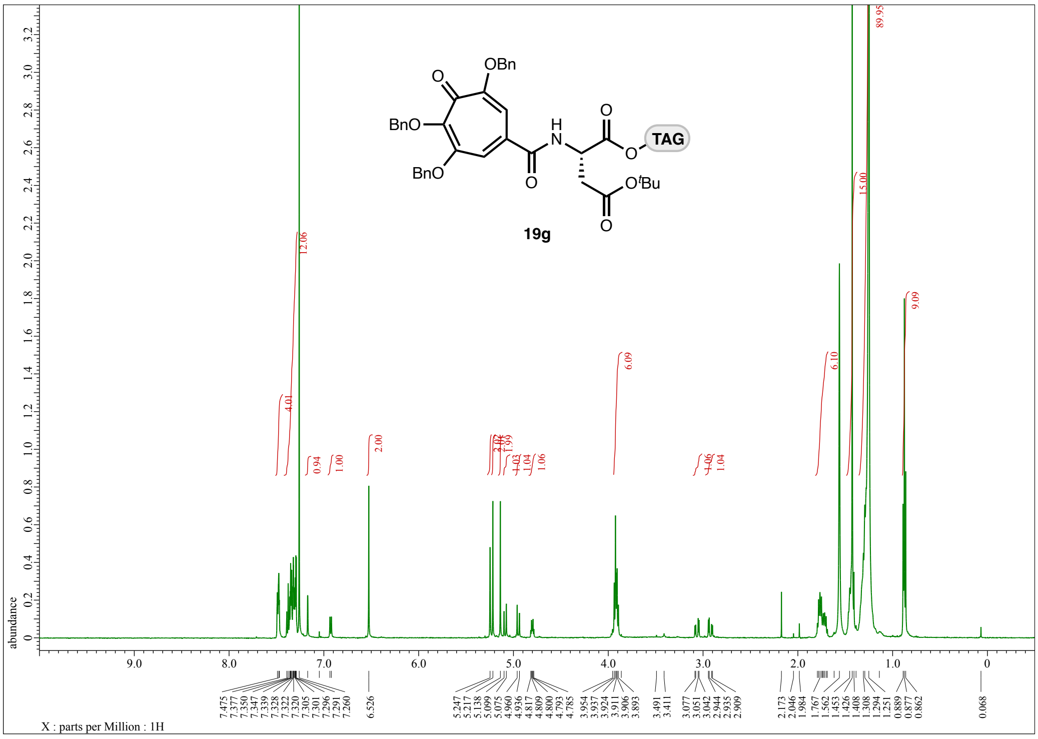

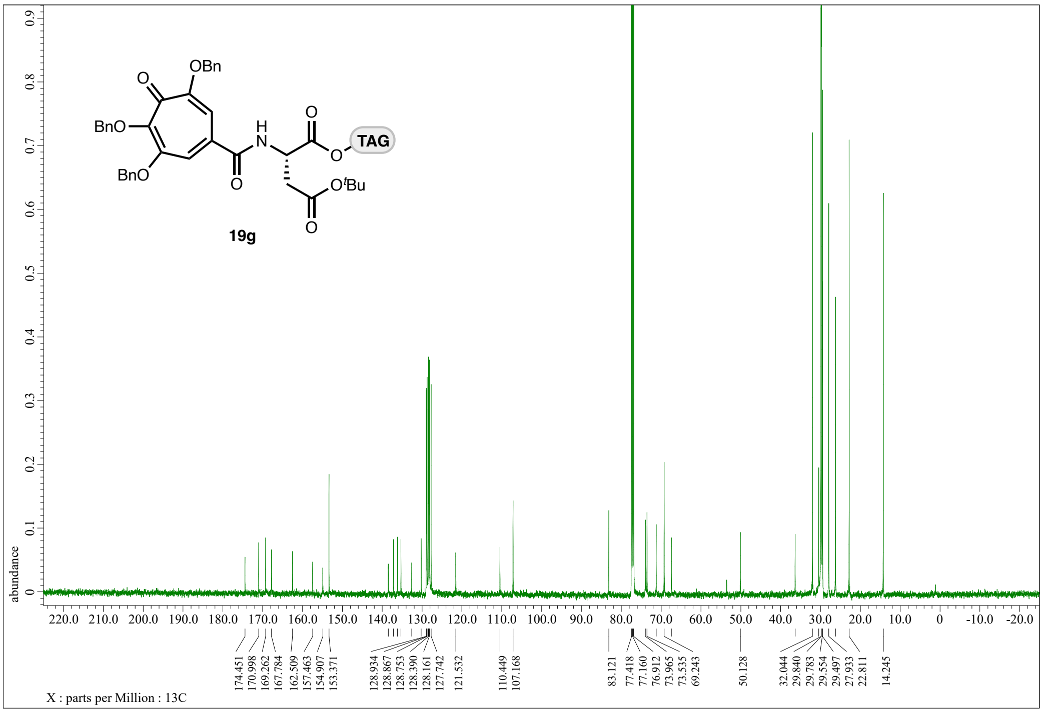

• BnO-tropolone-L-Orn(Boc)-OTAG (**19h**): ^{1}H NMR (500 MHz, CDCl$_3$), ^{13}C NMR (125 MHz, CDCl$_3$)

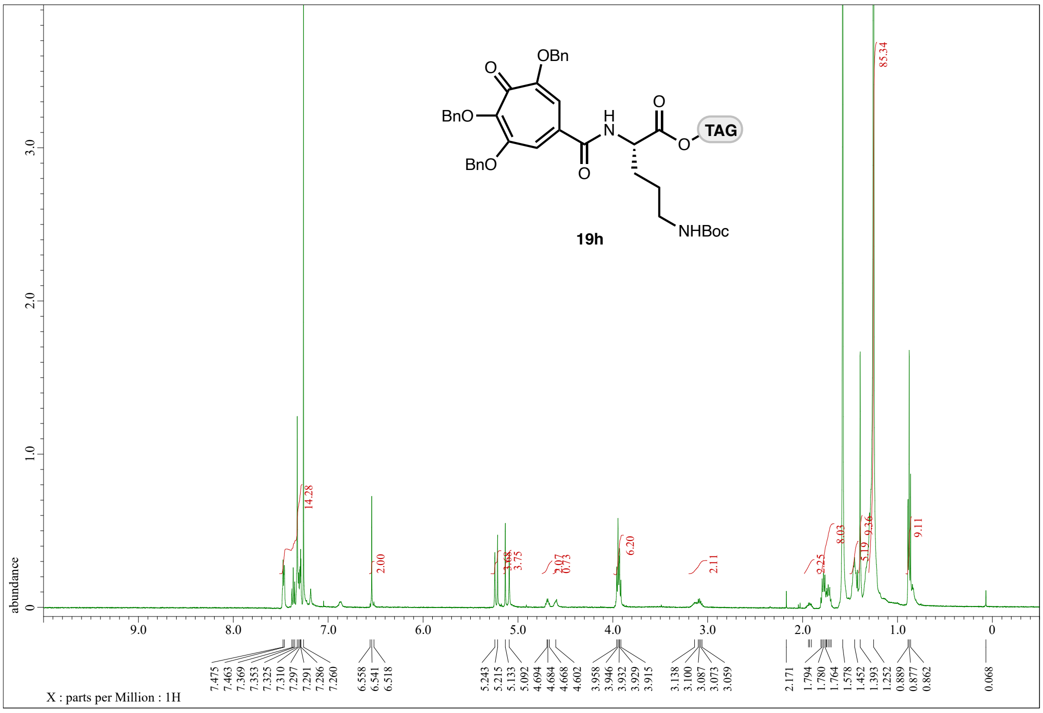

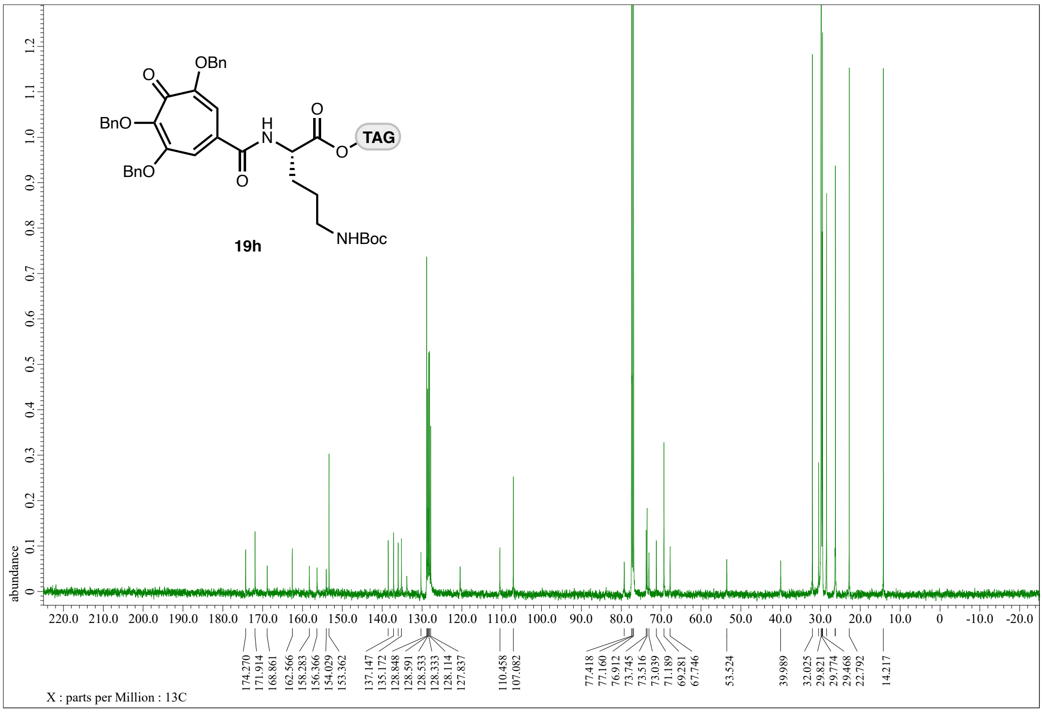

• BnO-tropolone-L-His(Boc)-OTAG (**19i**): ^{1}H NMR (500 MHz, $CDCl_3$), ^{13}C NMR (125 MHz, $CDCl_3$)

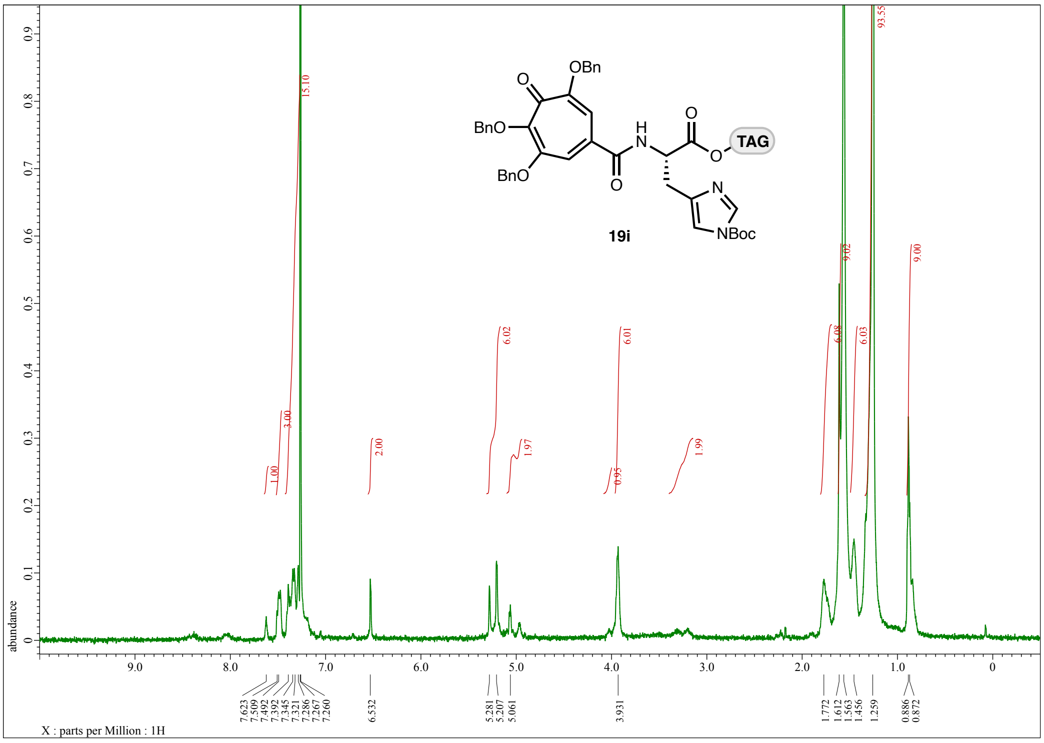

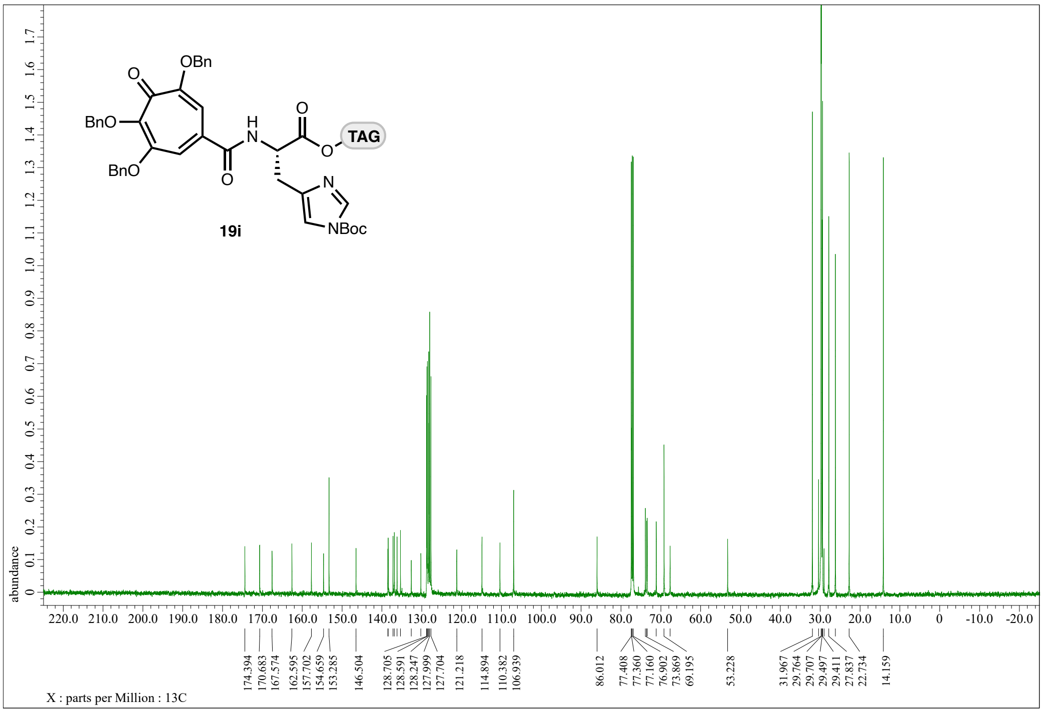

• BnO-tropolone-*N*-Me-L-Phe-OTAG (**19j**): ^{1}H NMR (500 MHz, CDCl$_3$), ^{13}C NMR (125 MHz, CDCl$_3$)

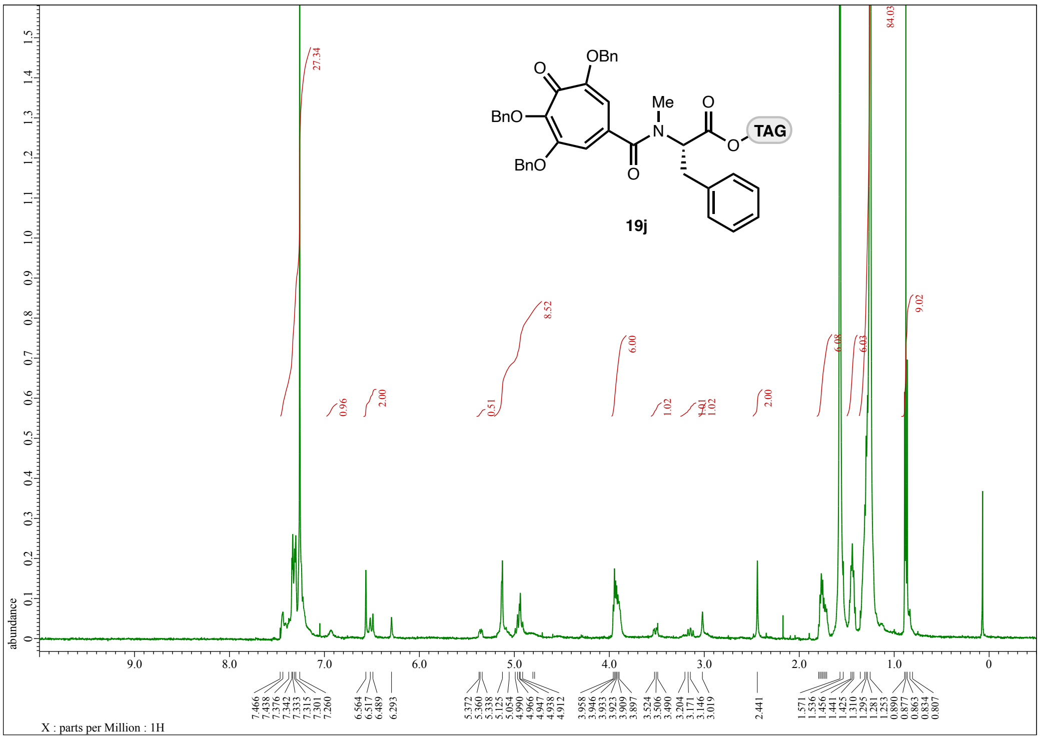

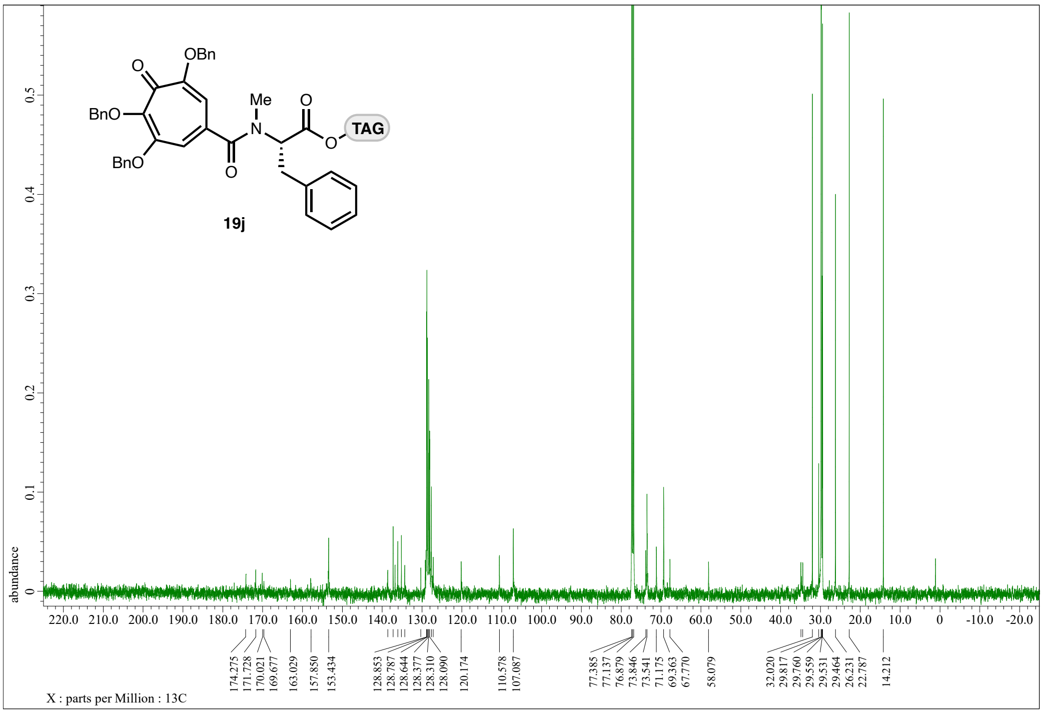

• BnO-tropolone-L-Tyr(^{t}Bu)-OTAG (**19k**): ^{1}H NMR (500 MHz, $CDCl_3$), ^{13}C NMR (125 MHz, $CDCl_3$)

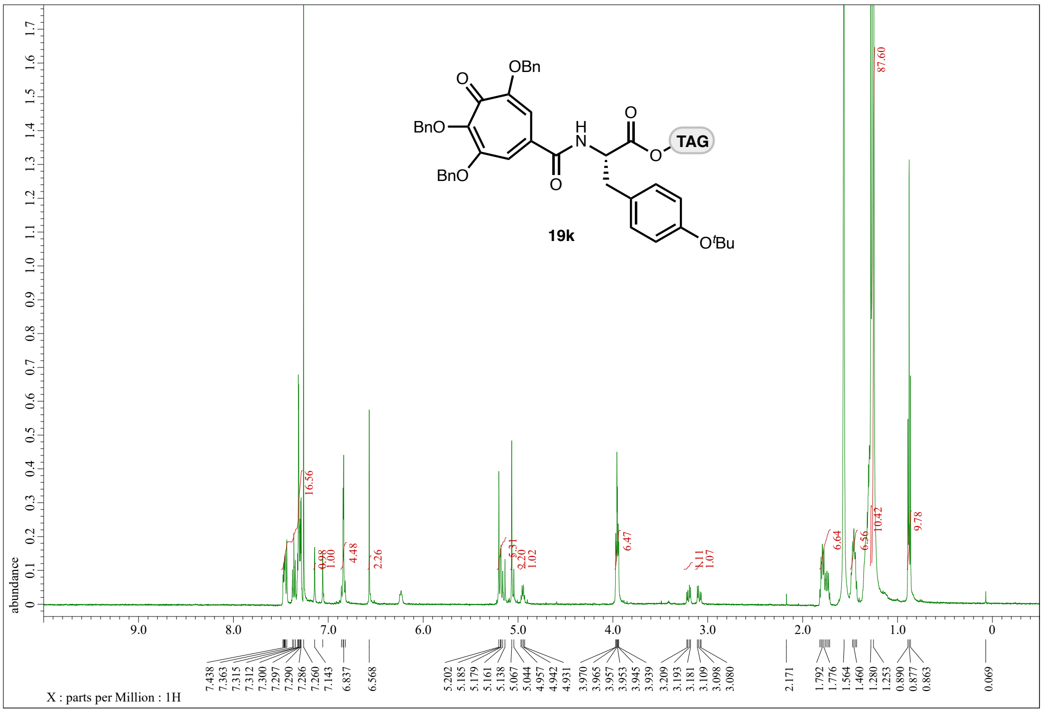

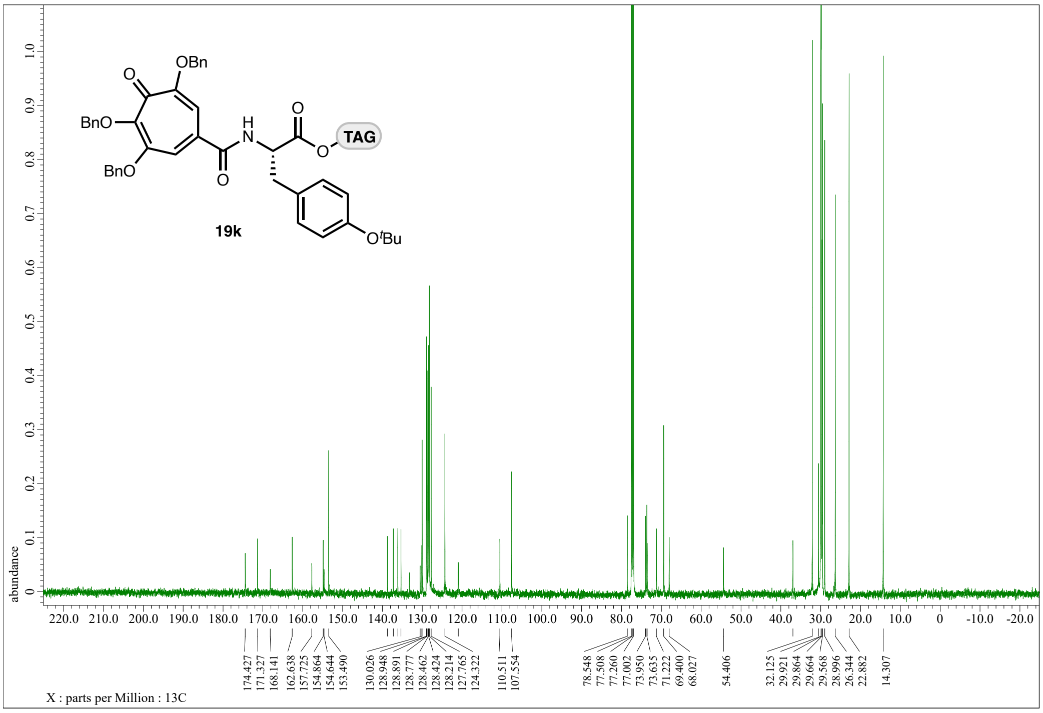

• BnO-tropolone-L-4-F-Phe-OTAG (**19l**): ^{1}H NMR (500 MHz, $CDCl_3$), ^{13}C NMR (125 MHz, $CDCl_3$)

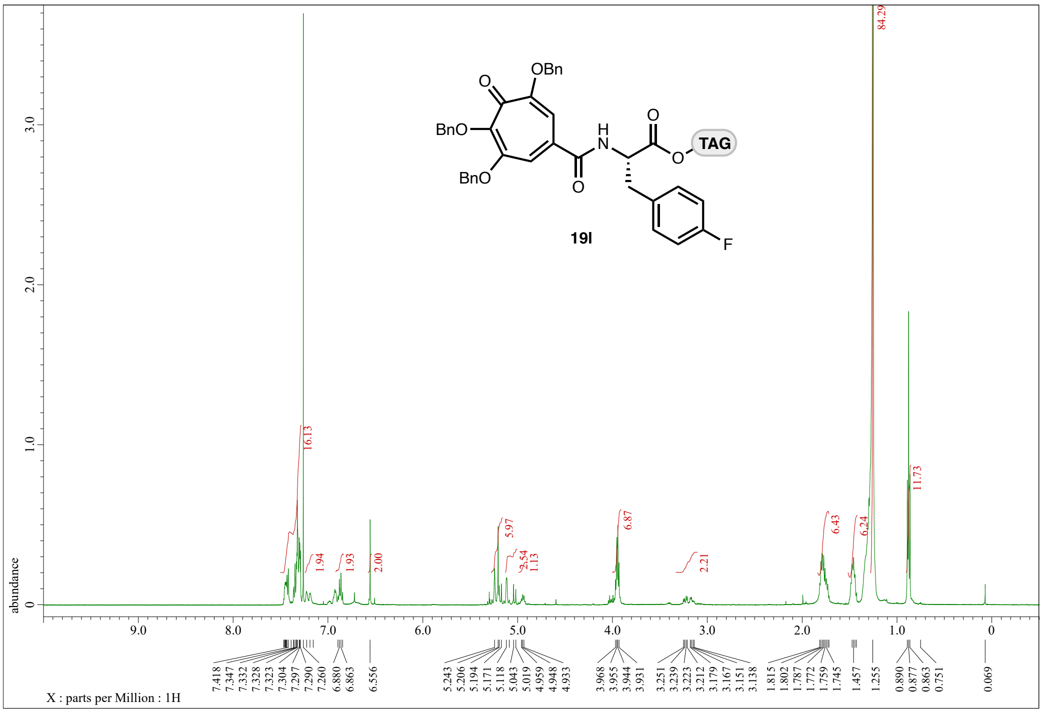

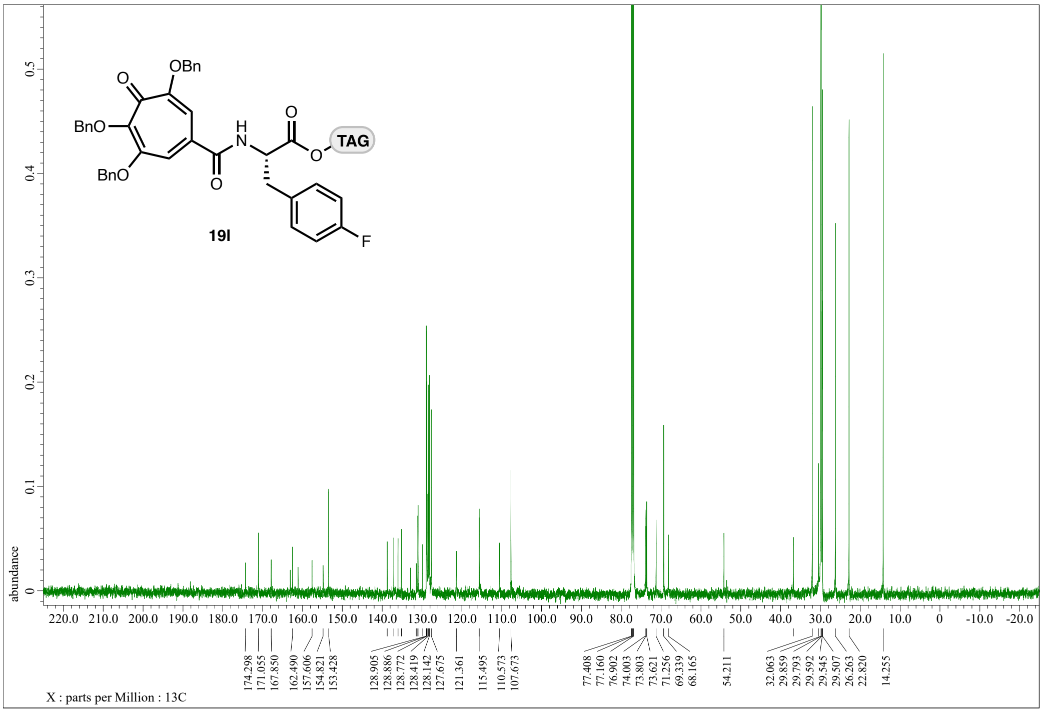

• BnO-tropolone-L-Trp(Boc)-OTAG (**19m**): ^{1}H NMR (500 MHz, $CDCl_3$), ^{13}C NMR (125 MHz, $CDCl_3$)

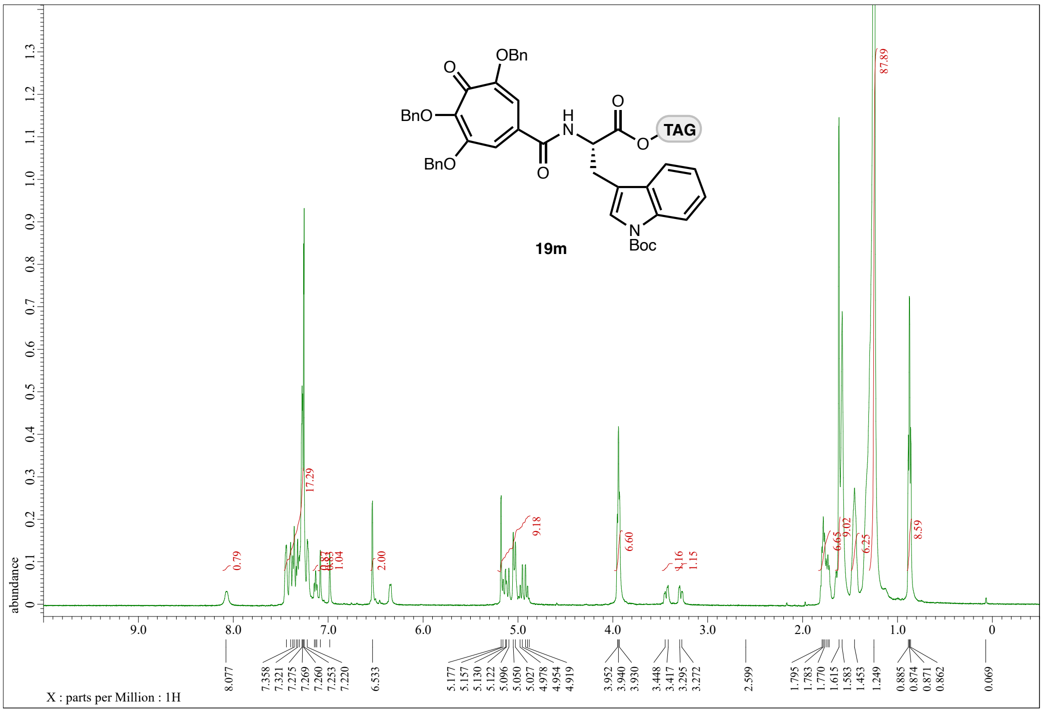

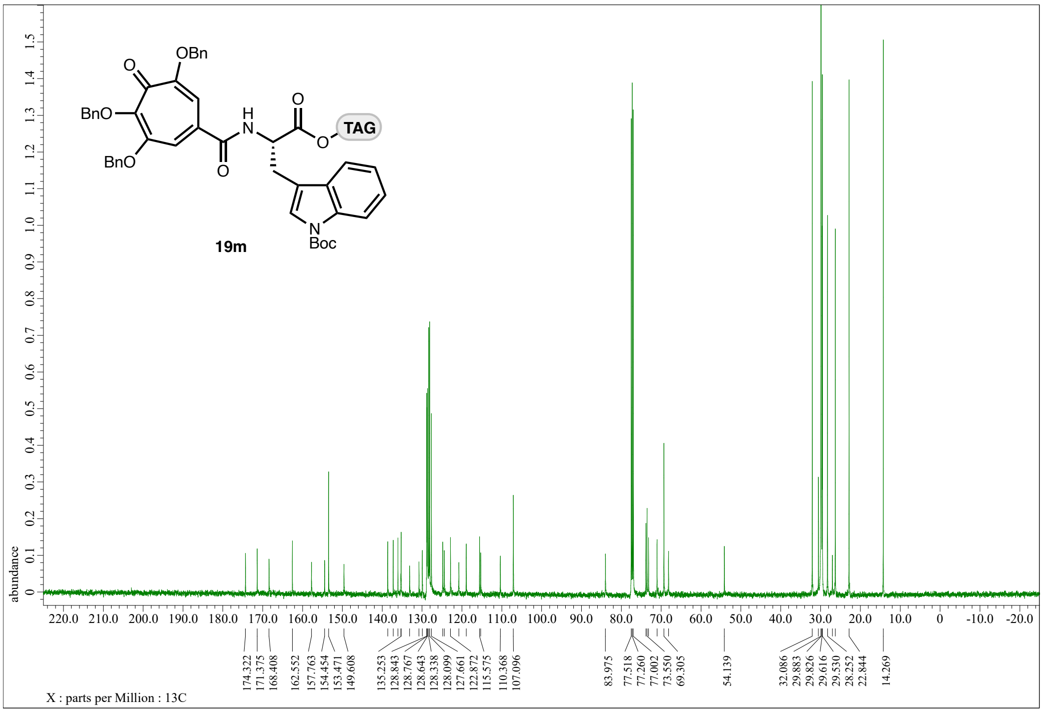

• Tropolone-D-Val-OH (**20**): ^{1}H NMR (500 MHz, CD_3OD), ^{13}C NMR (125 MHz, CD_3OD)

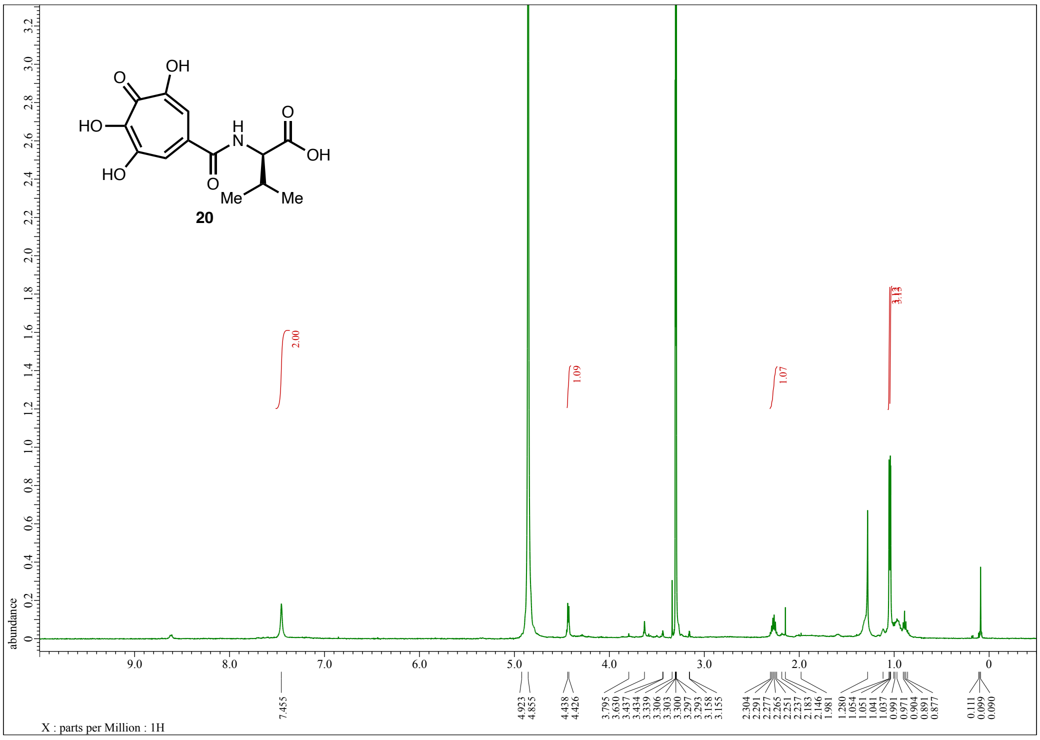

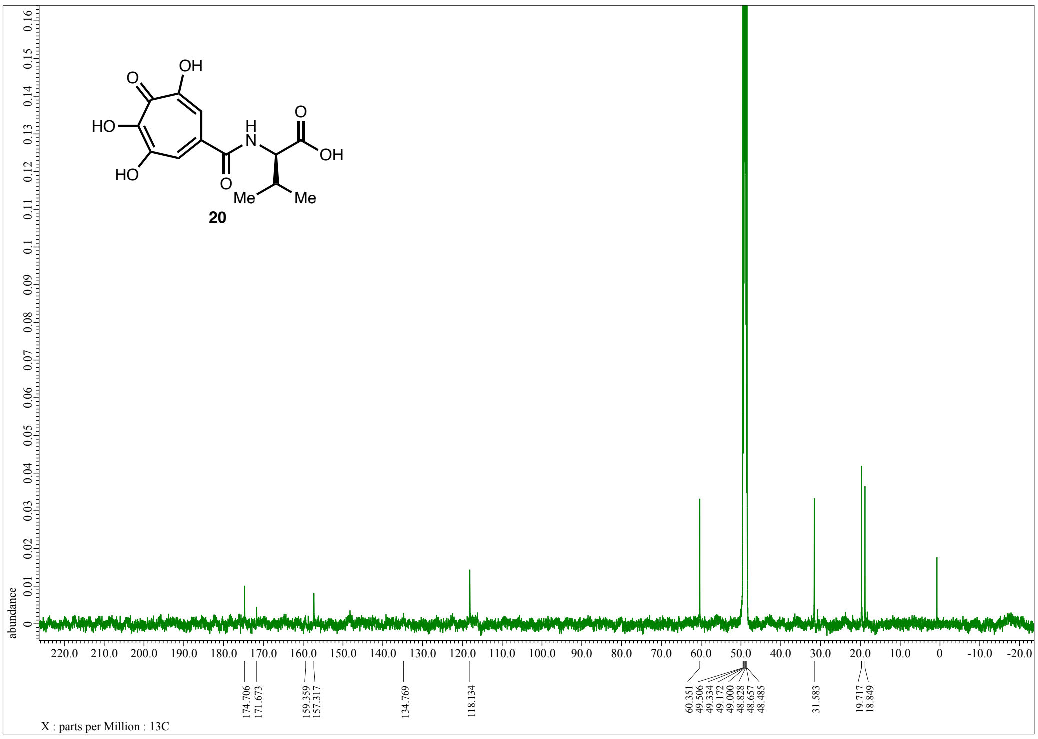

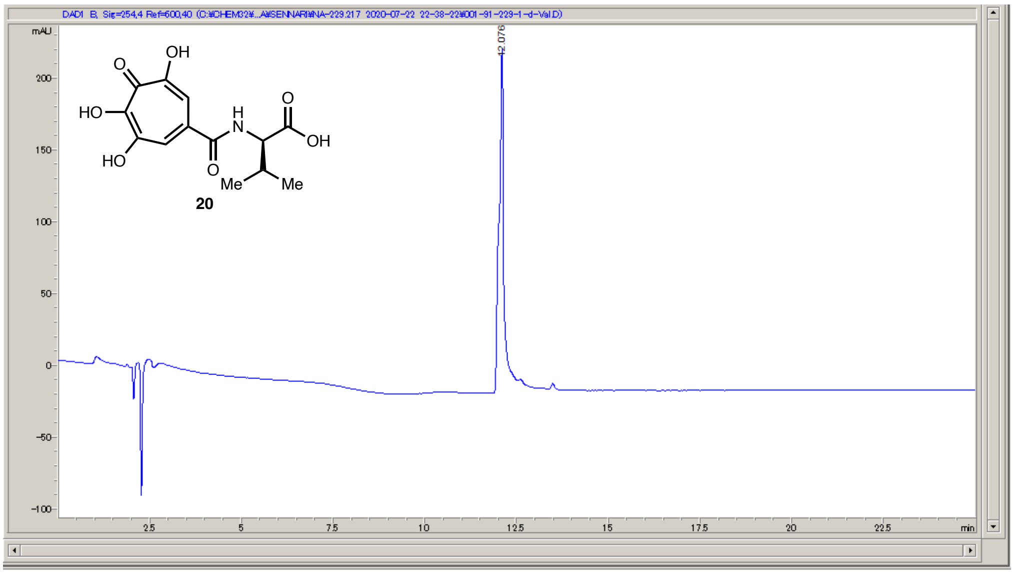

Retention time: 12.076 min

LC/UV method

Measuring equipment: Agilent 1260 Infinity LC

Column: Waters symmetry C18 Column (3.5 µm, 2.1 × 150 mm)

Mobile phase A: H_2O (containing 0.05% H_3PO_4)

Mobile phase B: MeCN (containing 0.05% H_3PO_4)

Linear gradient: A:B = 95:5 to 0:100 (0 – 25 min)

Flow late: 0.2 mL/min

Detect: UV 254 nm

Temperature: 40 ˚C

• Tropolone-L-Leu-OH (**21**): ^{1}H NMR (500 MHz, CD_3OD), ^{13}C NMR (125 MHz, CD_3OD)

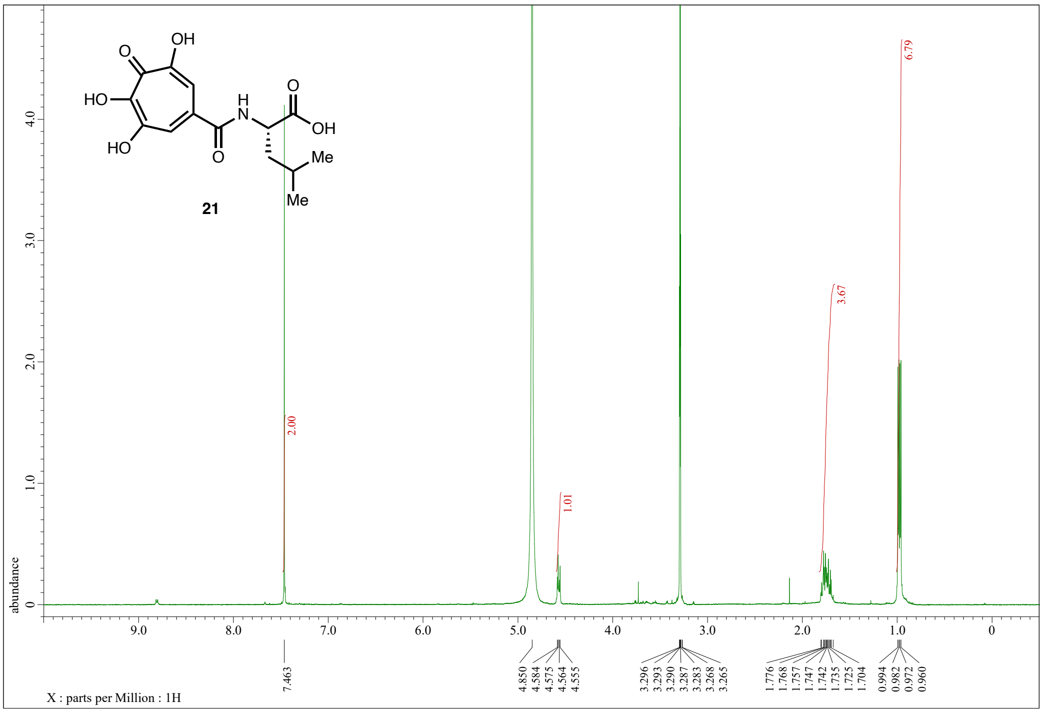

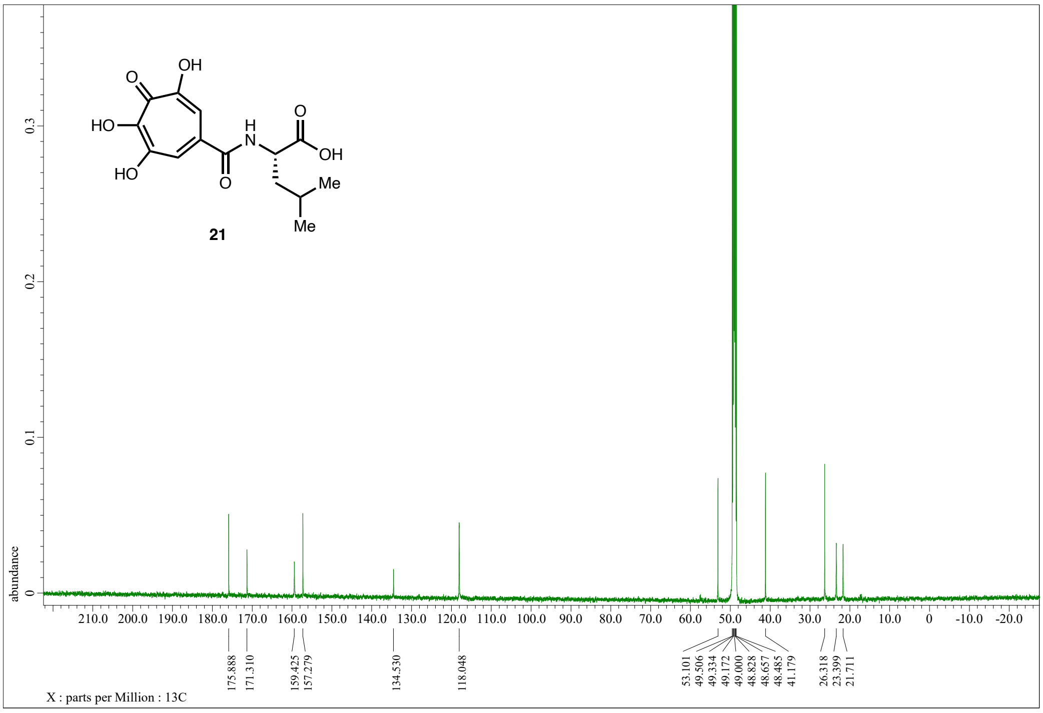

• Tropolone-*N*-Me-L-Leu-OH (**22**): ^{1}H NMR (500 MHz, CD_3OD), ^{13}C NMR (125 MHz, CD_3OD)

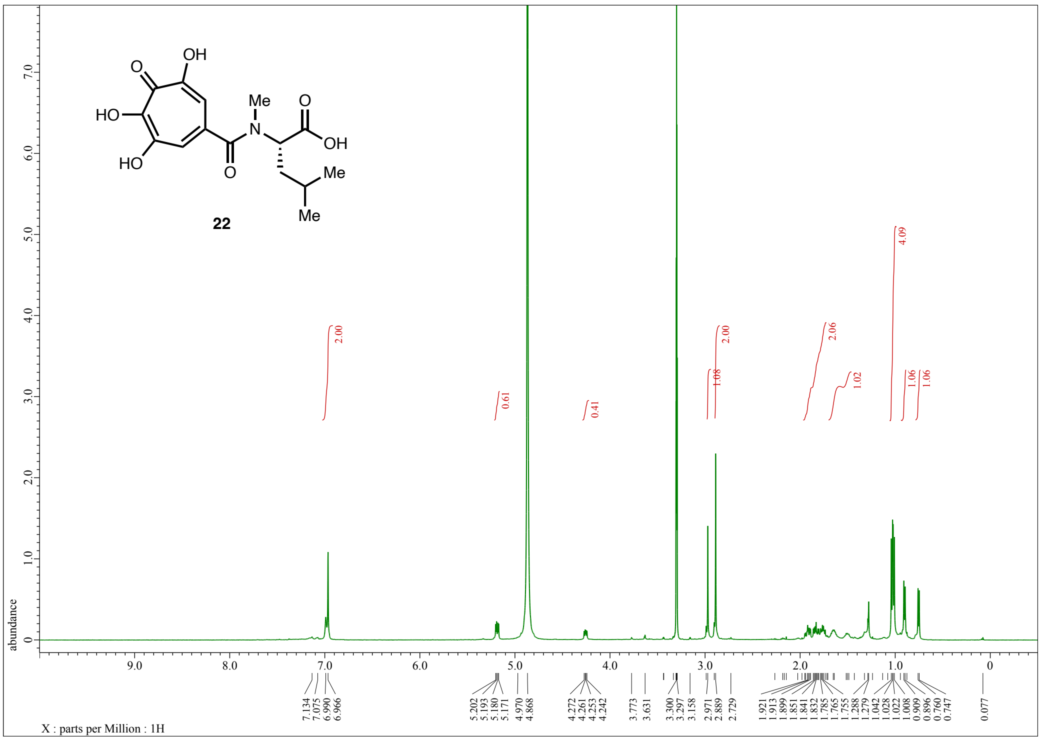

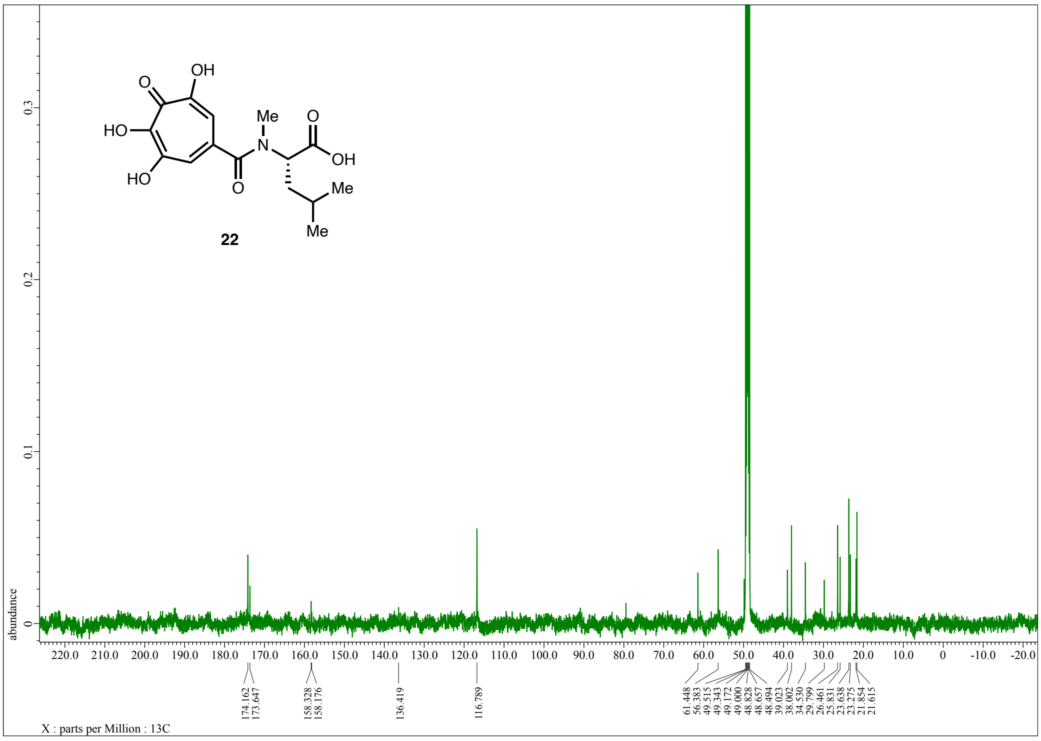

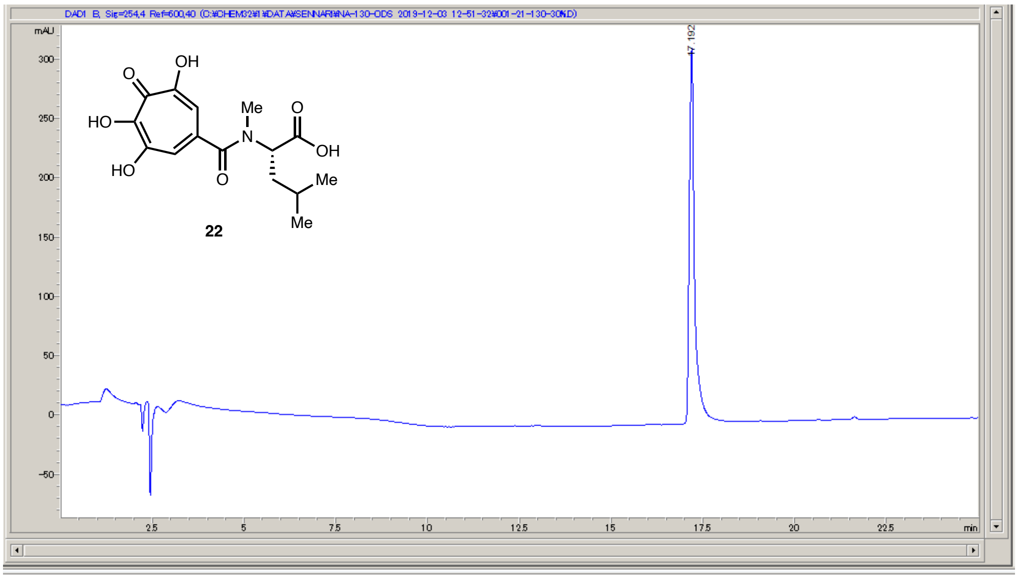

Retention time: 17.192 min

LC/UV method

Measuring equipment: Agilent 1260 Infinity LC

Column: Waters symmetry C18 Column (3.5 µm, 2.1 × 150 mm)

Mobile phase A: H_2O (containing 0.05% H_3PO_4)

Mobile phase B: MeCN (containing 0.05% H_3PO_4)

Linear gradient: A:B = 95:5 to 0:100 (0 – 25 min)

Flow late: 0.2 mL/min

Detect: UV 254 nm

Temperature: 40 ˚C

• Tropolone-L-Ile-OH (**23**): ^{1}H NMR (500 MHz, CD_3OD), ^{13}C NMR (125 MHz, CD_3OD)

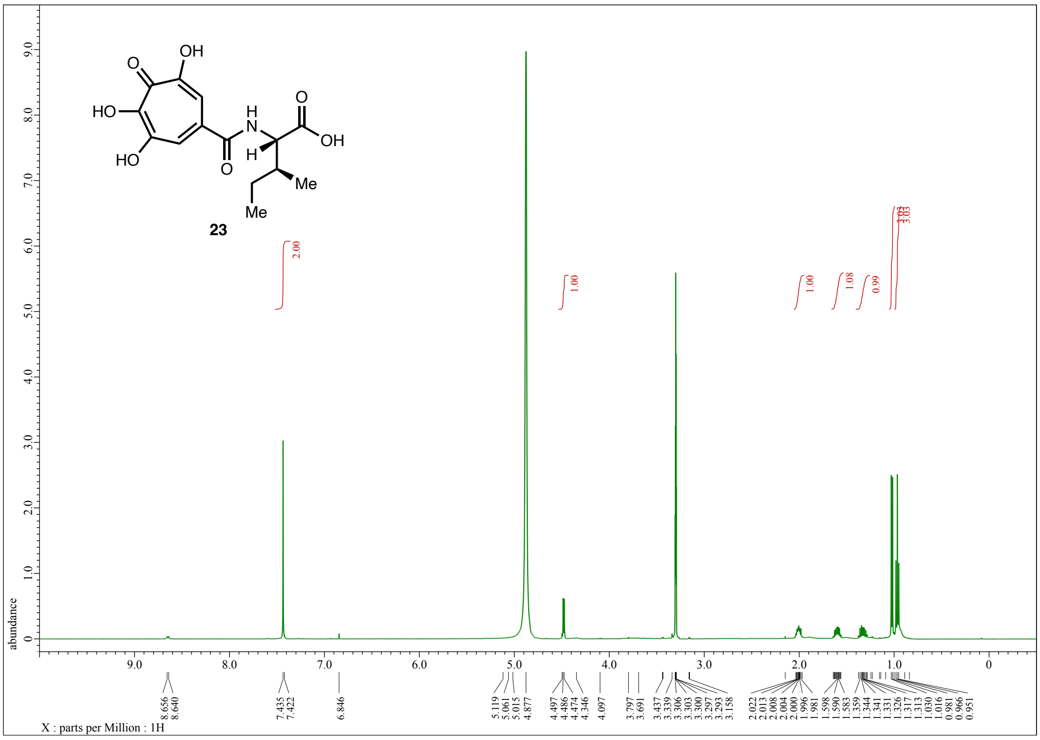

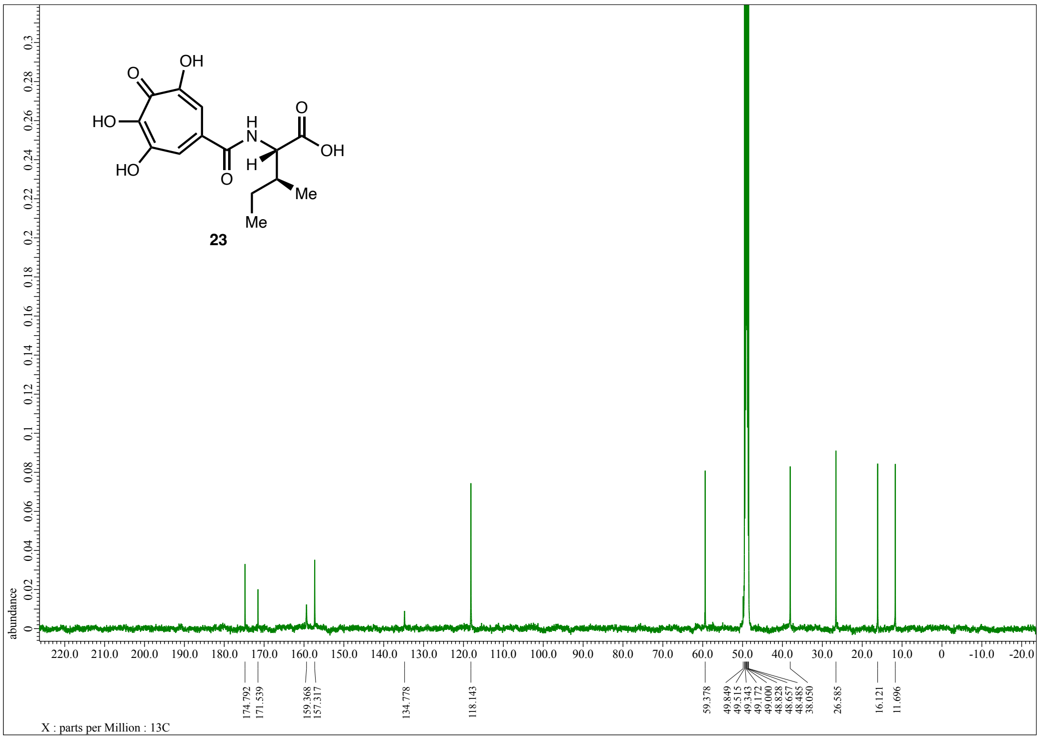

• Tropolone-L-Thr-OH (**24**): ^{1}H NMR (500 MHz, CD_3OD), ^{13}C NMR (125 MHz, CD_3OD)

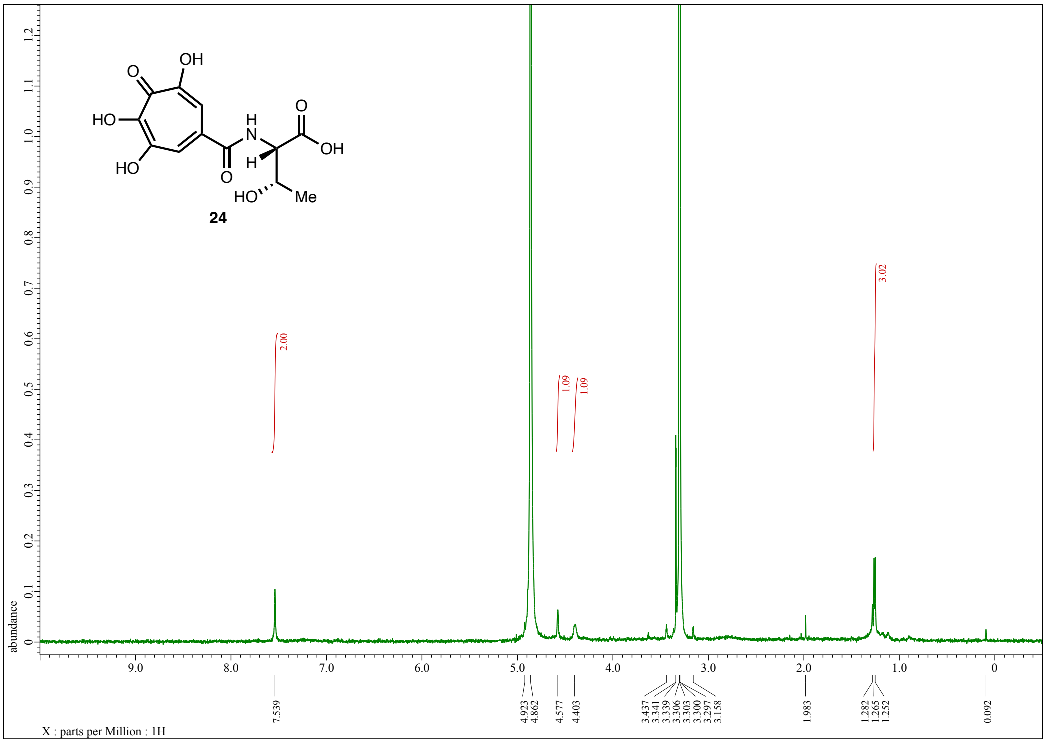

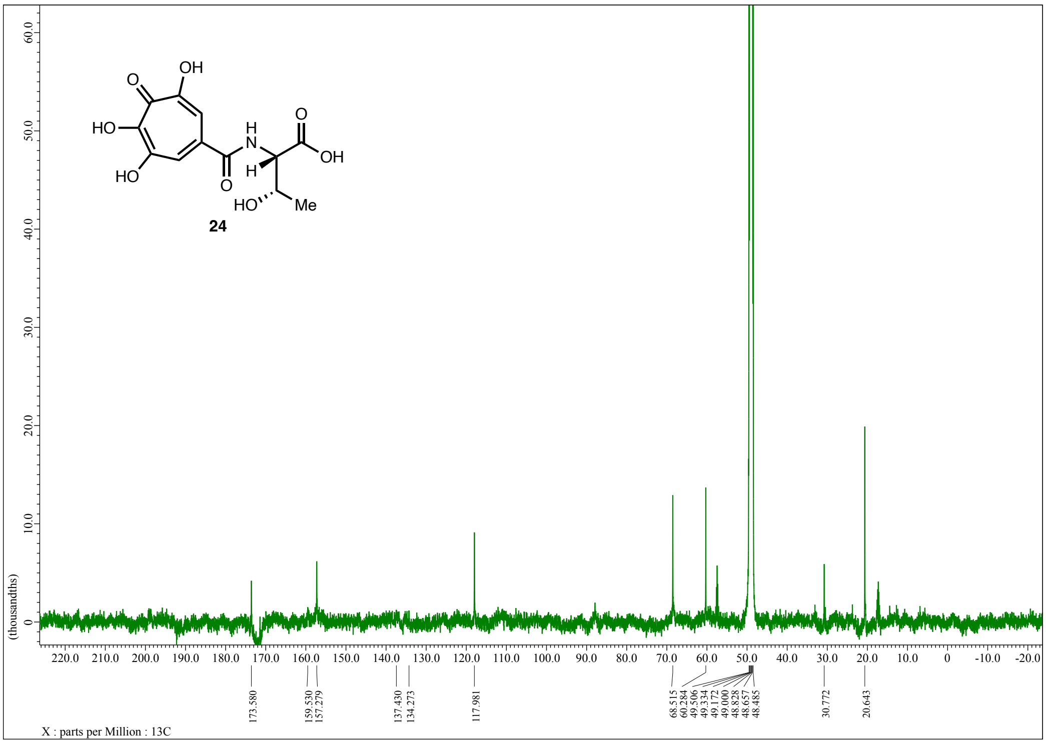

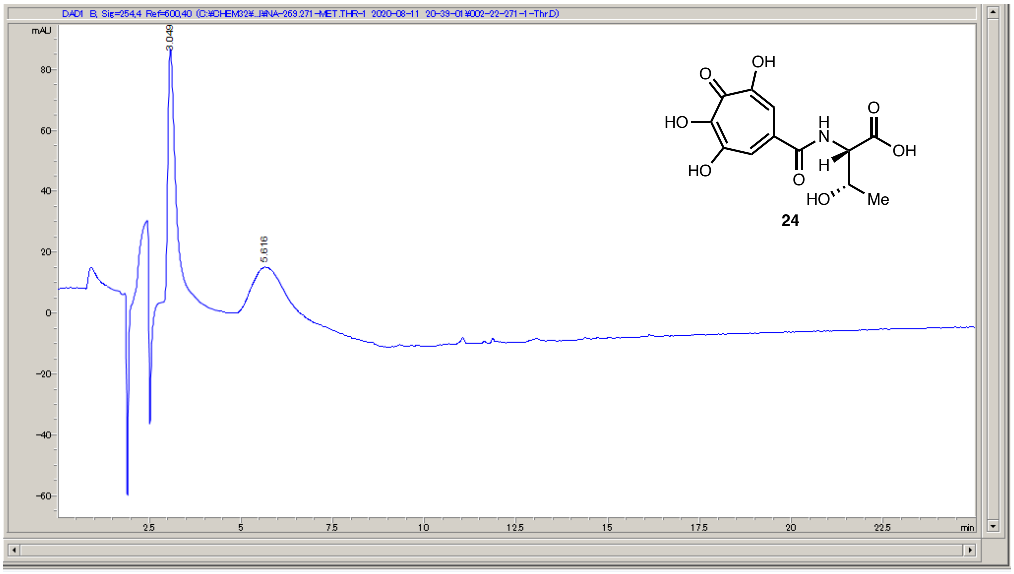

Retention time: 5.616 min

<u>LC/UV method</u>

Measuring equipment: Agilent 1260 Infinity LC

Column: Waters symmetry C18 Column (3.5 μm, 2.1 × 150 mm)

Mobile phase A: H_2O (containing 0.05% H_3PO_4)

Mobile phase B: MeCN (containing 0.05% H_3PO_4)

Linear gradient: A:B = 95:5 to 0:100 (0 – 25 min)

Flow late: 0.2 mL/min

Detect: UV 254 nm

Temperature: 40 ˚C

• Tropolone-L-Met-OH (**25**): ^{1}H NMR (500 MHz, CD_3OD), ^{13}C NMR (125 MHz, CD_3OD)

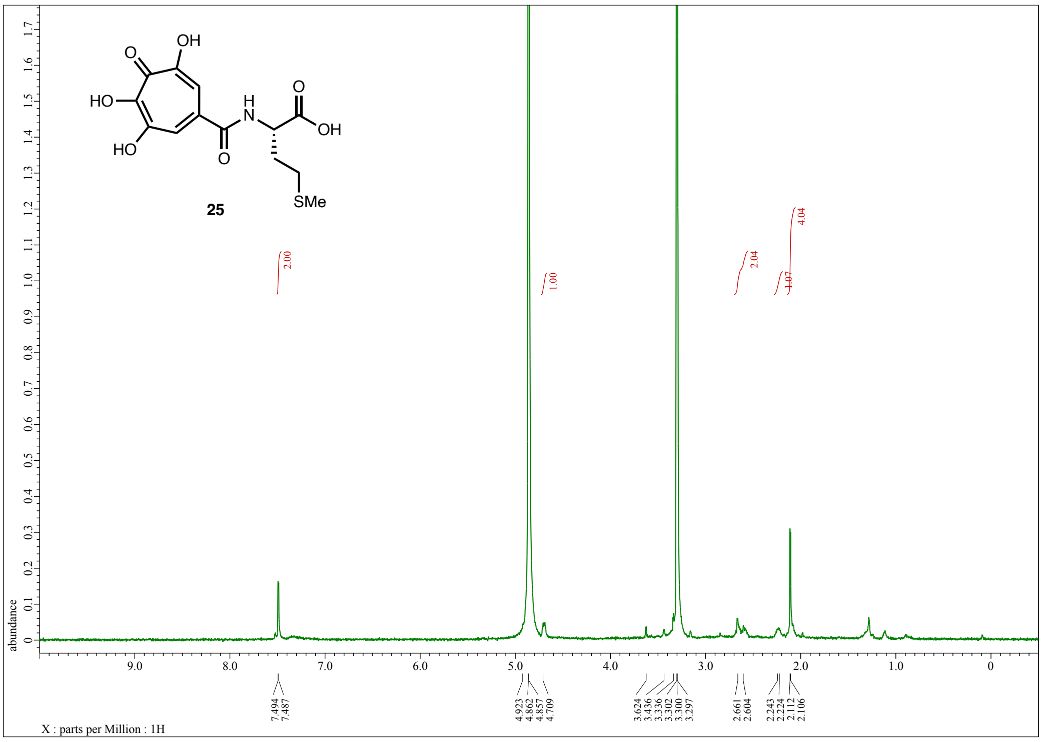

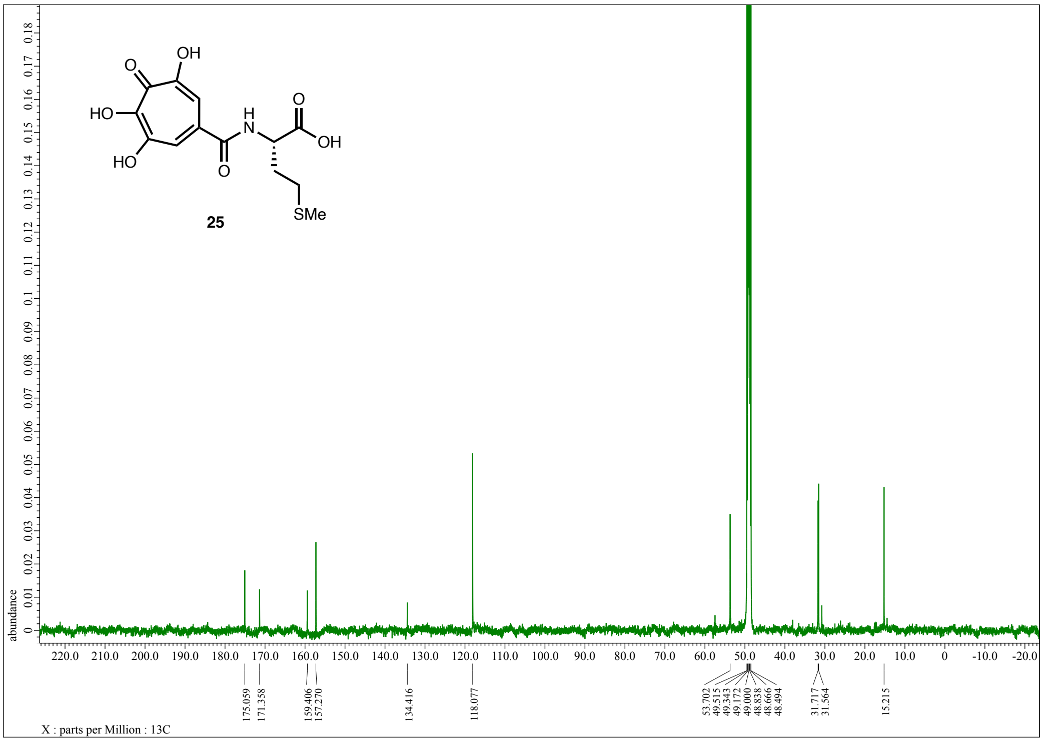

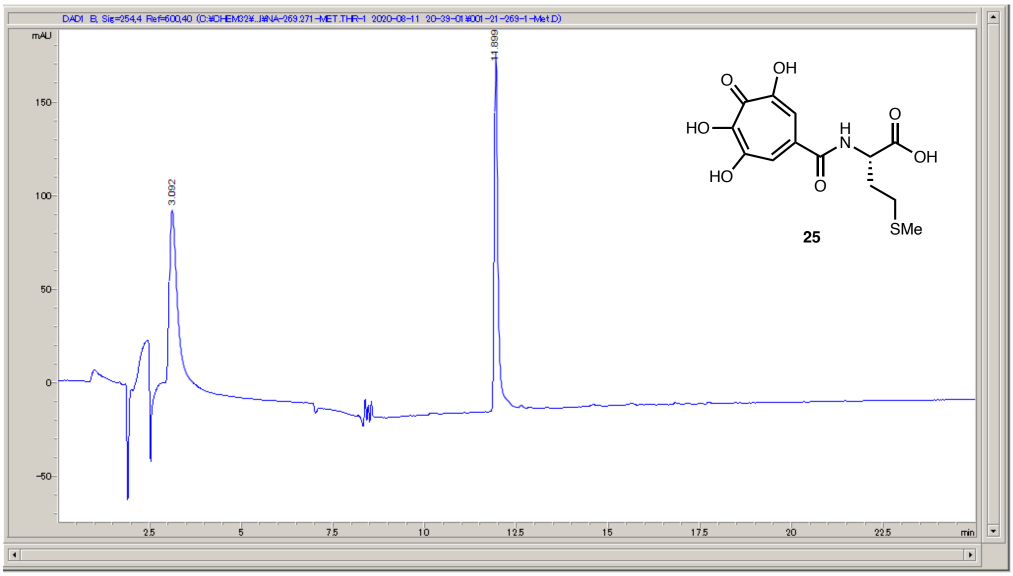

Retention time: 11.999 min

LC/UV method

Measuring equipment: Agilent 1260 Infinity LC

Column: Waters symmetry C18 Column (3.5 µm, 2.1 × 150 mm)

Mobile phase A: H_2O (containing 0.05% H_3PO_4)

Mobile phase B: MeCN (containing 0.05% H_3PO_4)

Linear gradient: A:B = 95:5 to 0:100 (0 – 25 min)

Flow late: 0.2 mL/min

Detect: UV 254 nm

Temperature: 40 ˚C

• Tropolone-L-Asp-OH (**26**): ^{1}H NMR (500 MHz, CD_3OD), ^{13}C NMR (125 MHz, CD_3OD)

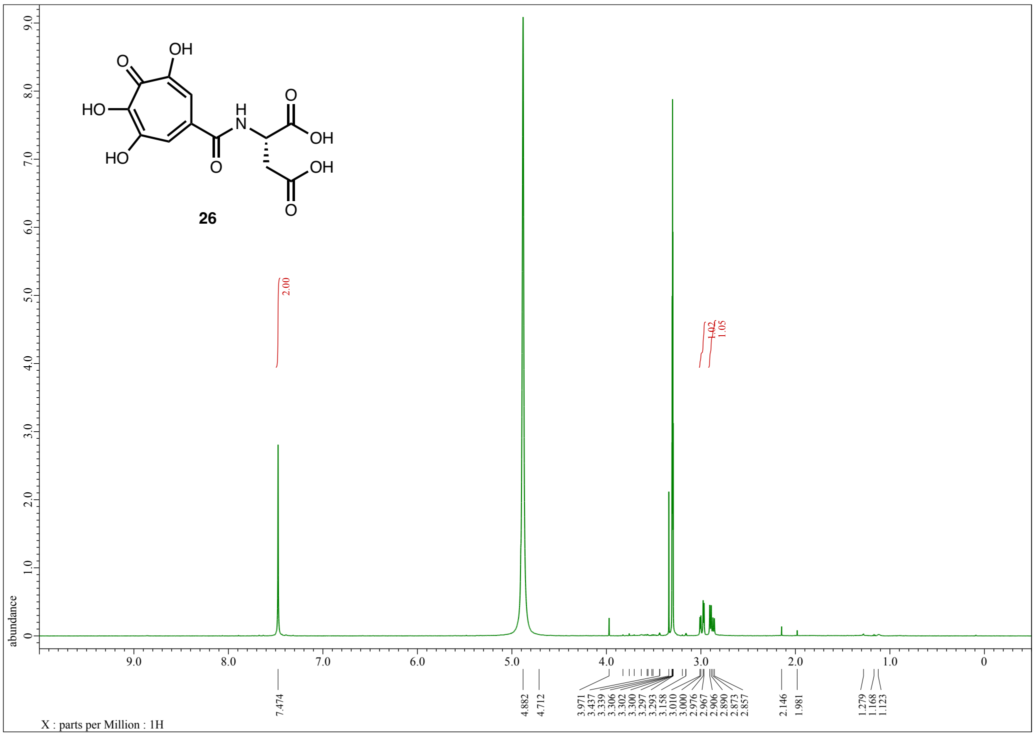

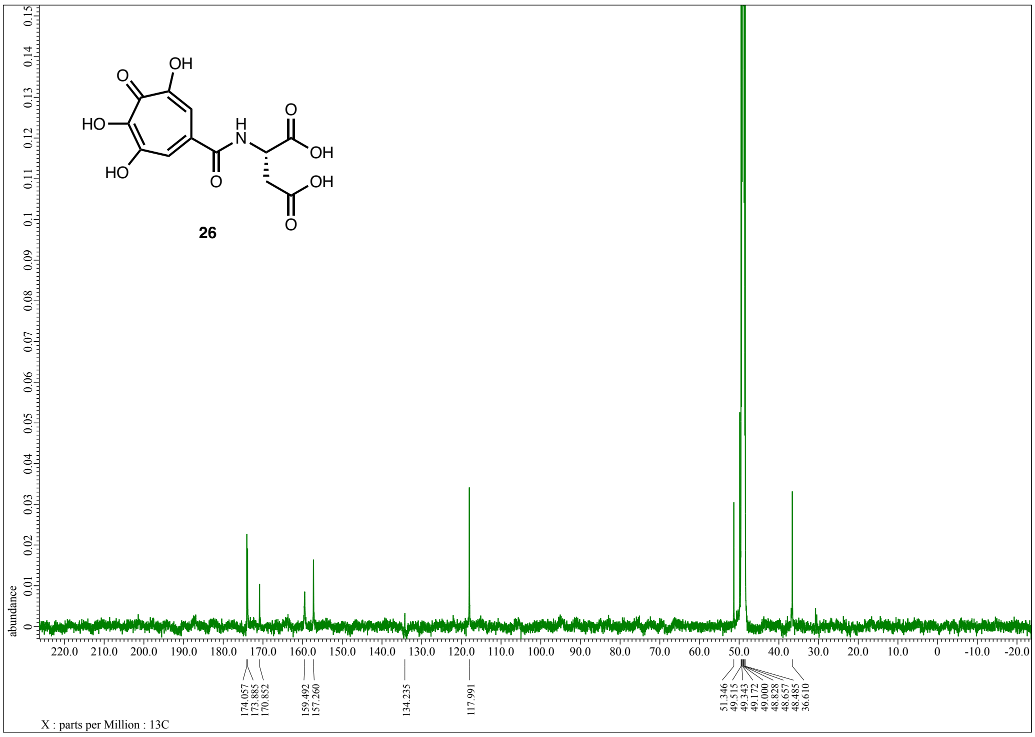

• Tropolone-L-Orn-OH (**27**): ^{1}H NMR (500 MHz, CD_3OD), ^{13}C NMR (125 MHz, CD_3OD)

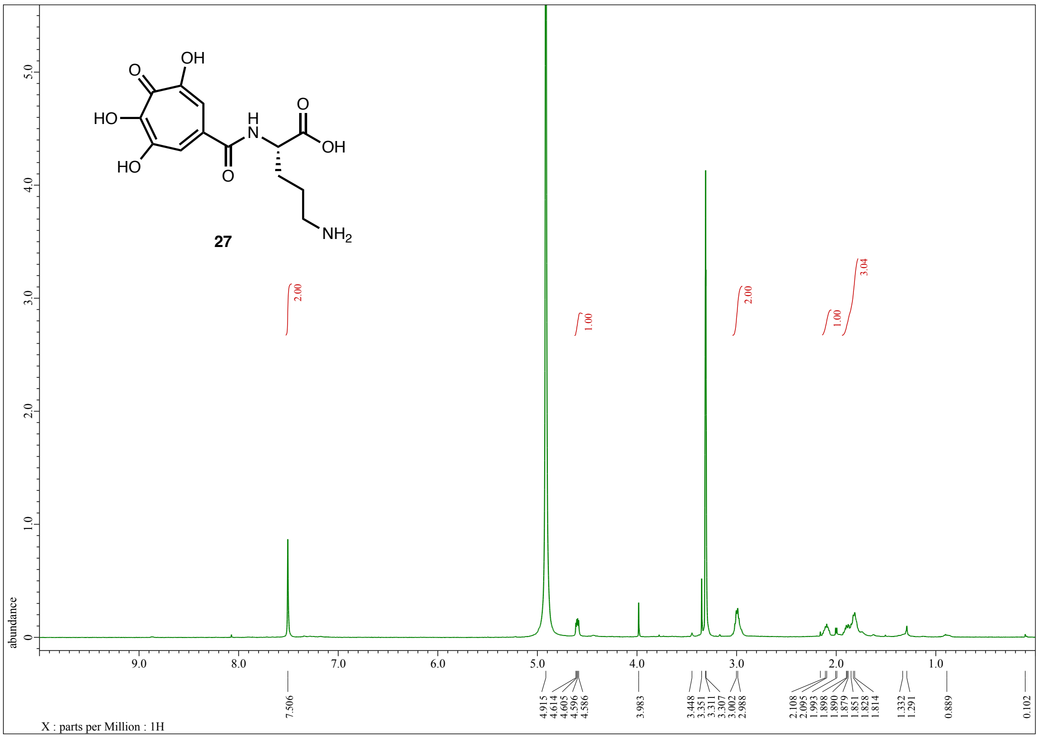

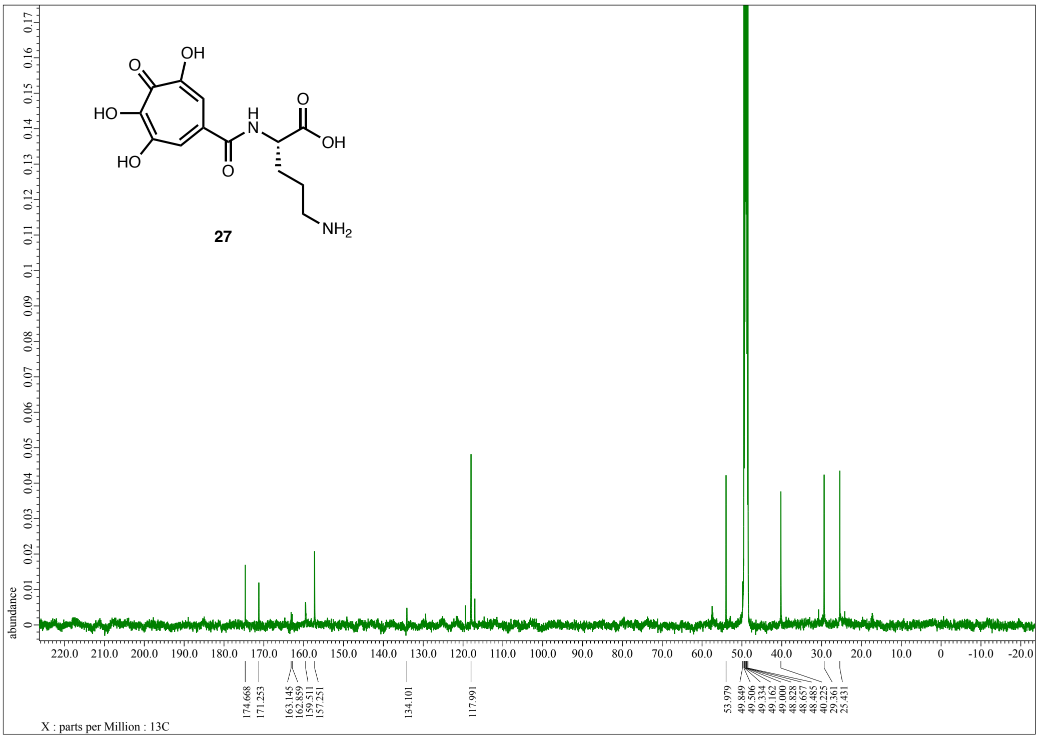

• Tropolone-L-His-OH (**28**): ^{1}H NMR (500 MHz, CD_3OD), ^{13}C NMR (125 MHz, CD_3OD)

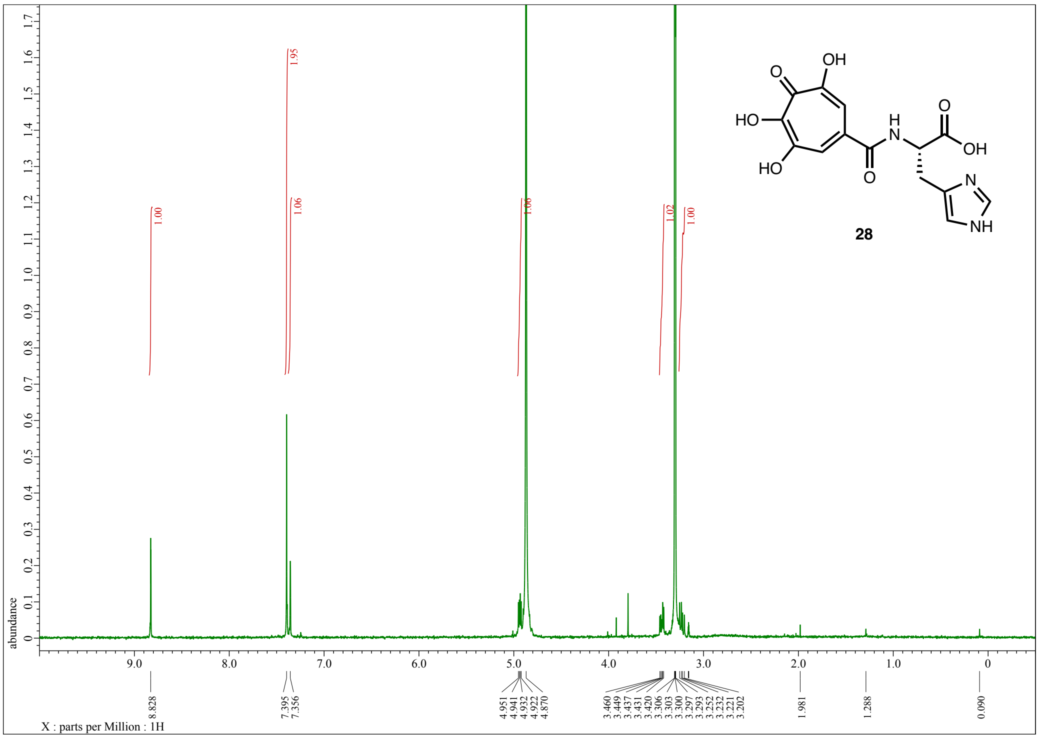

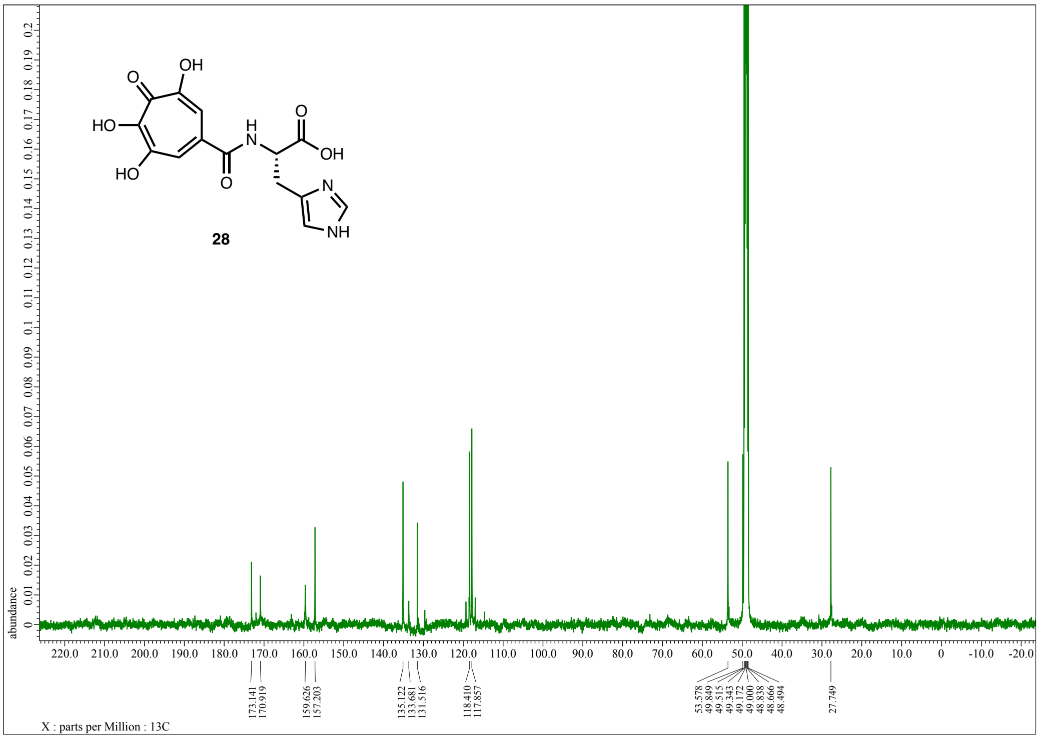

• Tropolone-*N*-Me-L-Phe-OH (**29**): ^{1}H NMR (500 MHz, CD_3OD), ^{13}C NMR (125 MHz, $(CD_3)_2CO$)

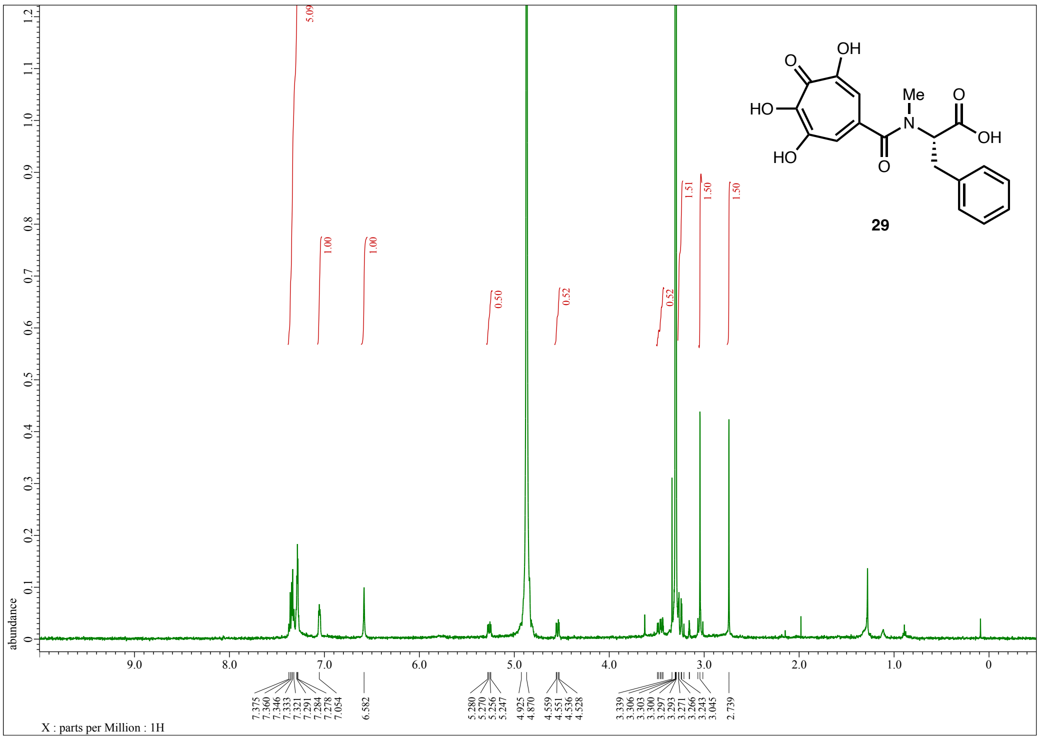

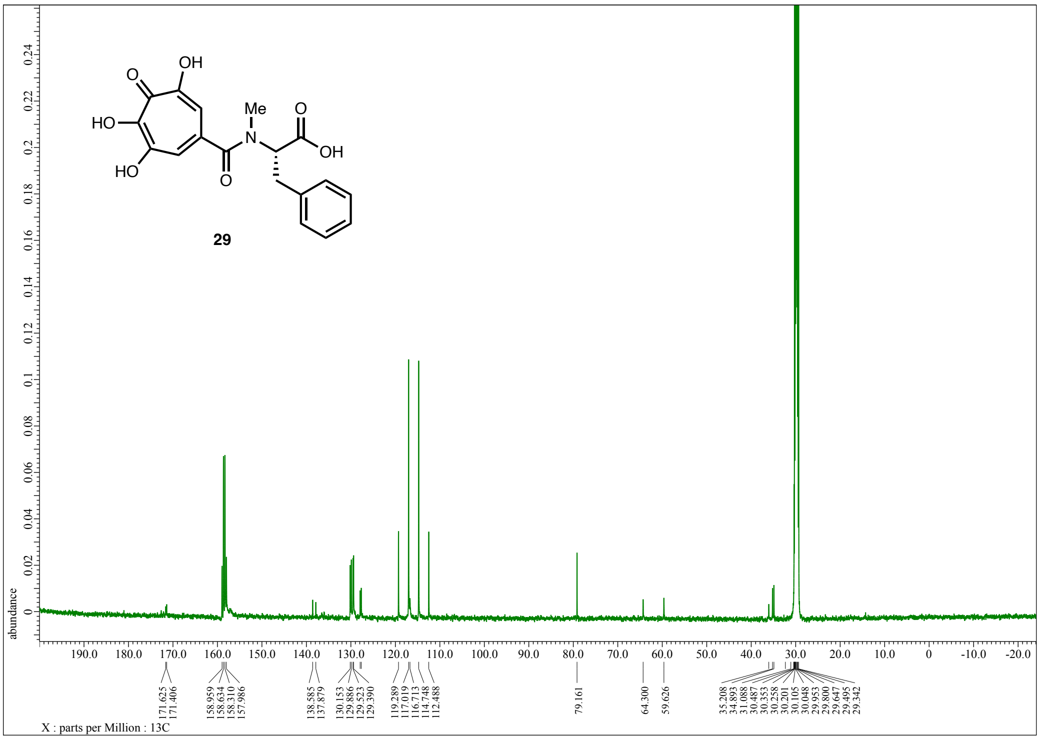

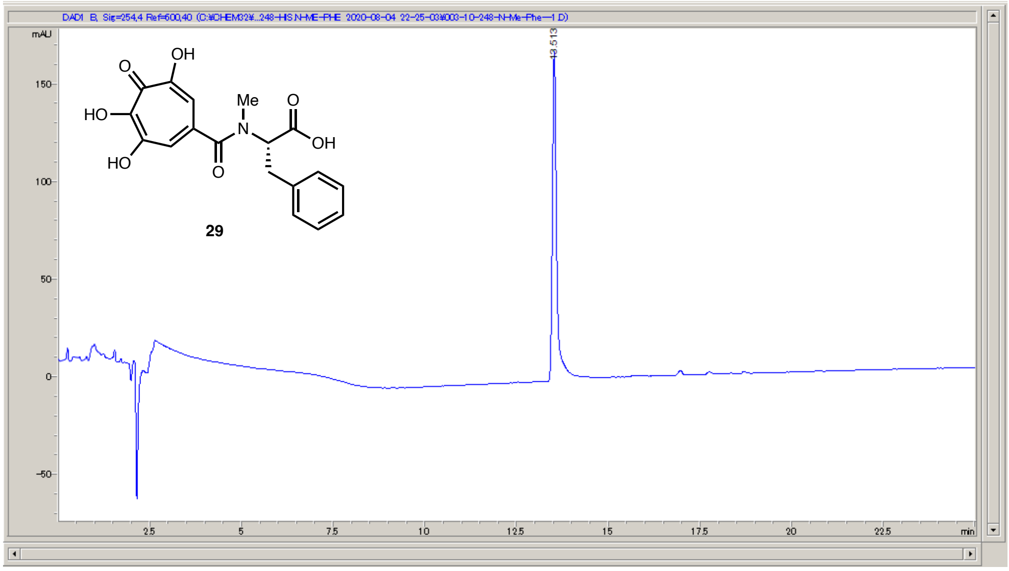

Retention time: 13.531 min

LC/UV method

Measuring equipment: Agilent 1260 Infinity LC

Column: Waters symmetry C18 Column (3.5 µm, 2.1 × 150 mm)

Mobile phase A: H_2O (containing 0.05% H_3PO_4)

Mobile phase B: MeCN (containing 0.05% H_3PO_4)

Linear gradient: A:B = 95:5 to 0:100 (0 – 25 min)

Flow late: 0.2 mL/min

Detect: UV 254 nm

Temperature: 40 ˚C

• Tropolone-L-Tyr-OH (**30**): ^{1}H NMR (500 MHz, CD_3OD), ^{13}C NMR (125 MHz, CD_3OD)

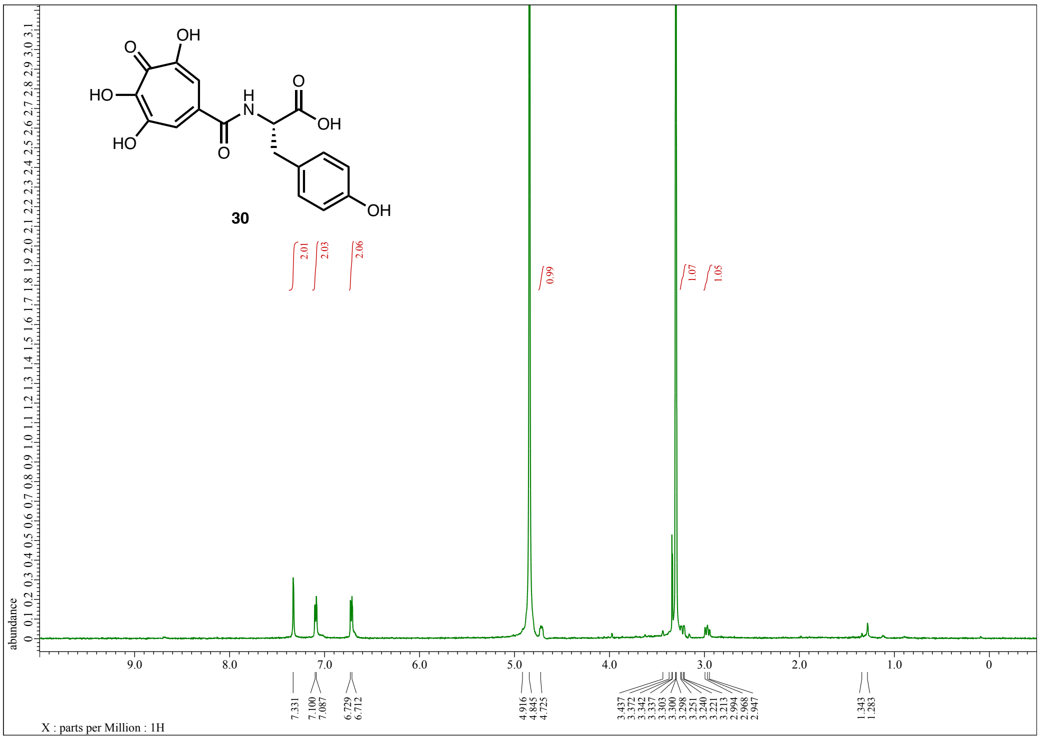

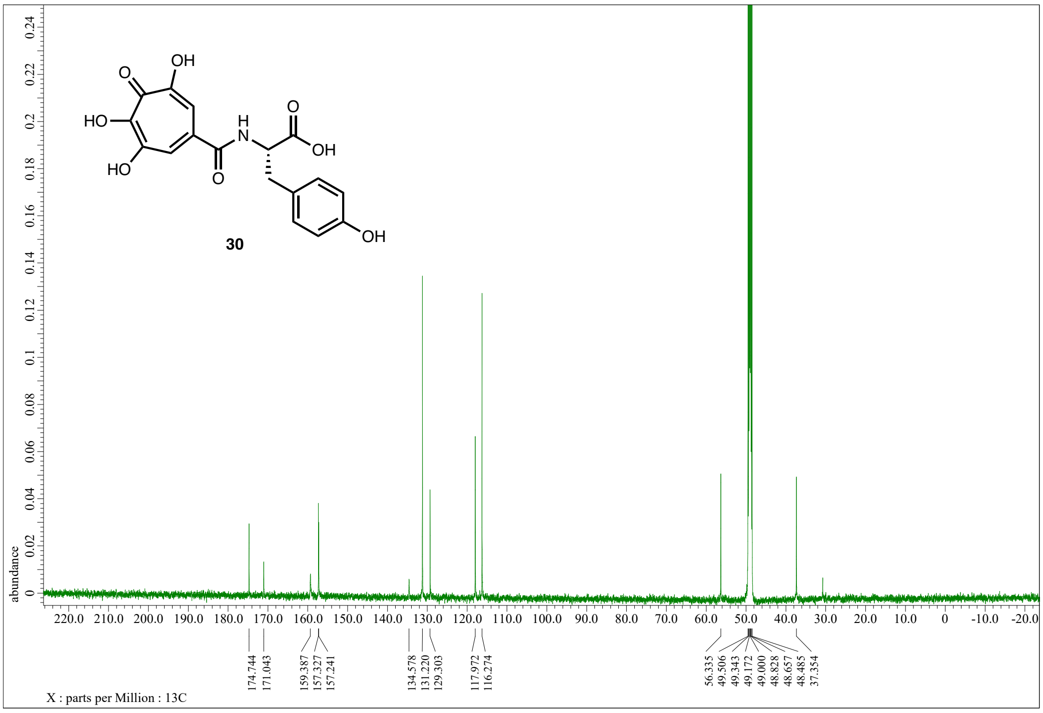

• Tropolone-L-4-Phe-OH (**31**): ^{1}H NMR (500 MHz, CD_3OD), ^{13}C NMR (125 MHz, CD_3OD)

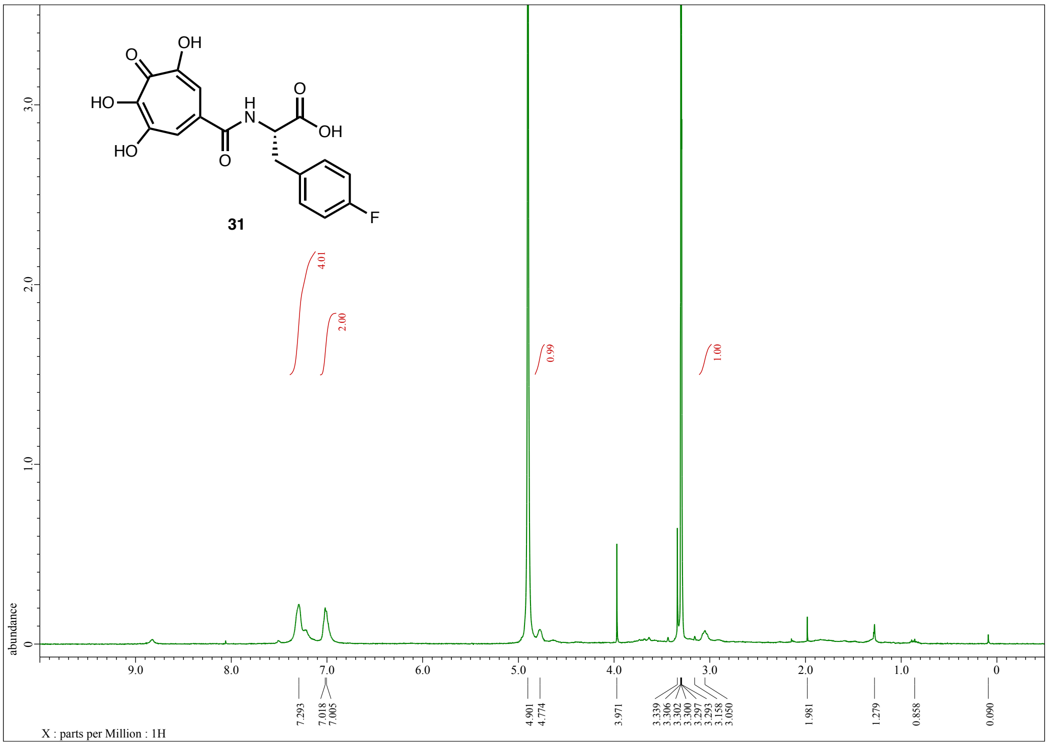

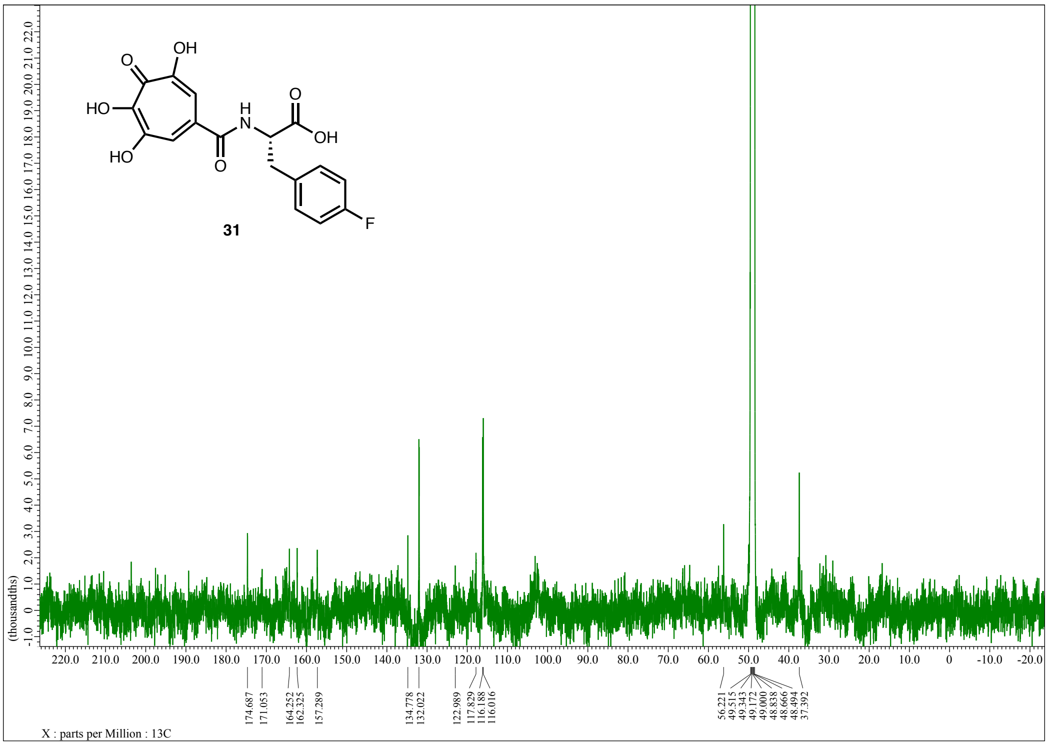

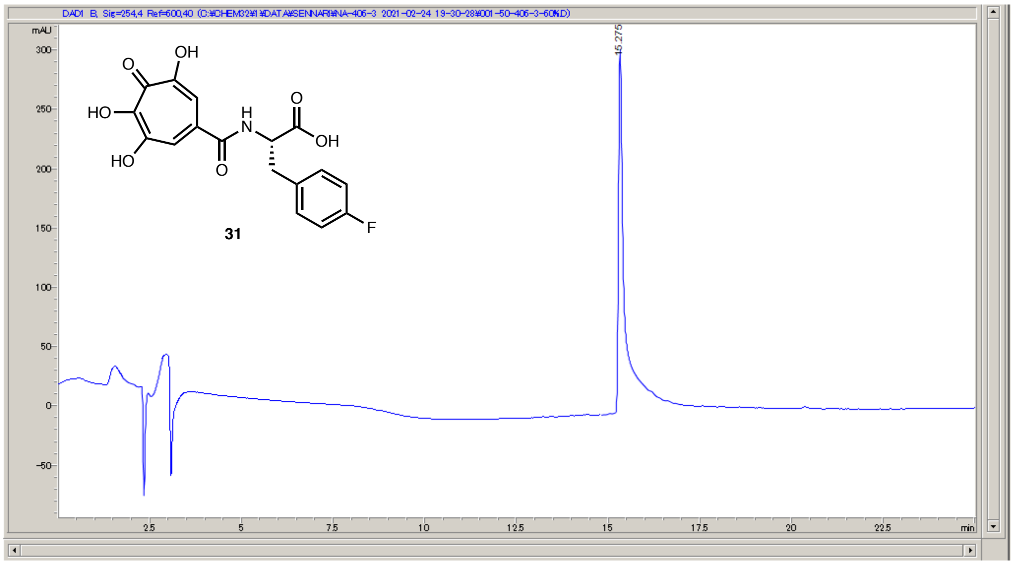

Retention time: 15.275 min

<u>LC/UV method</u>

Measuring equipment: Agilent 1260 Infinity LC

Column: Waters symmetry C18 Column (3.5 μm, 2.1 × 150 mm)

Mobile phase A: H_2O (containing 0.05% H_3PO_4)

Mobile phase B: MeCN (containing 0.05% H_3PO_4)

Linear gradient: A:B = 95:5 to 0:100 (0 – 25 min)

Flow late: 0.2 mL/min

Detect: UV 254 nm

Temperature: 40 ˚C

• Tropolone-L-Trp-OH (**32**): ^{1}H NMR (500 MHz, $(CD_3)_2CO$ containing with 1% TFA), ^{13}C NMR (125 MHz, $(CD_3)_2CO$ containing with 1% TFA)

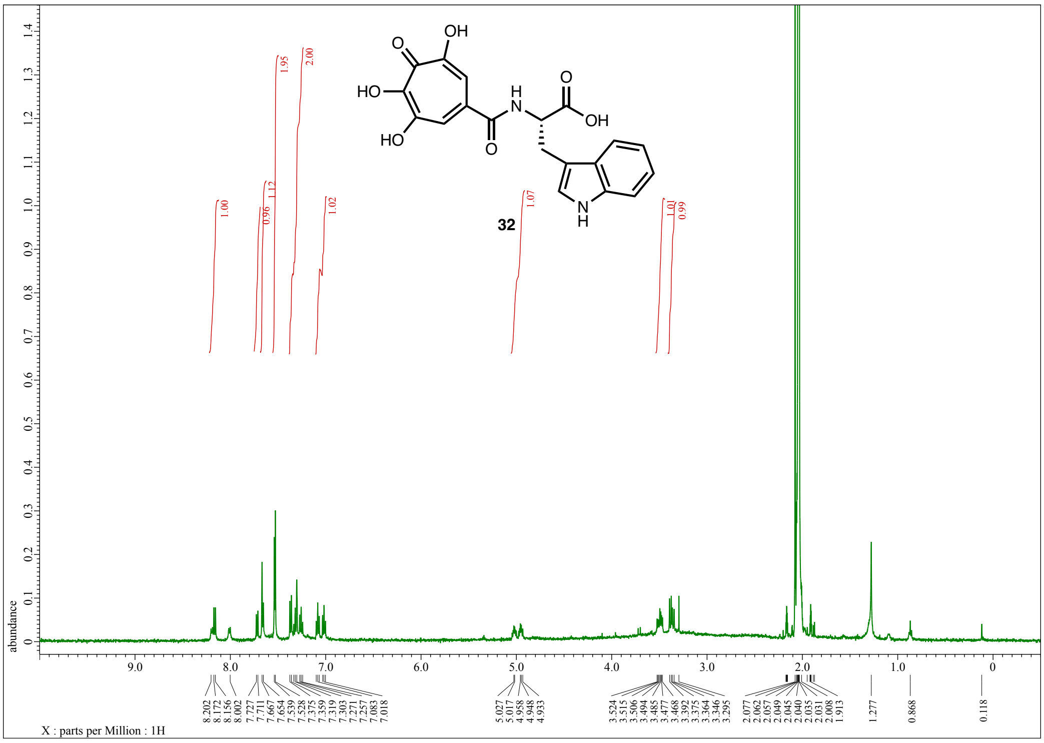

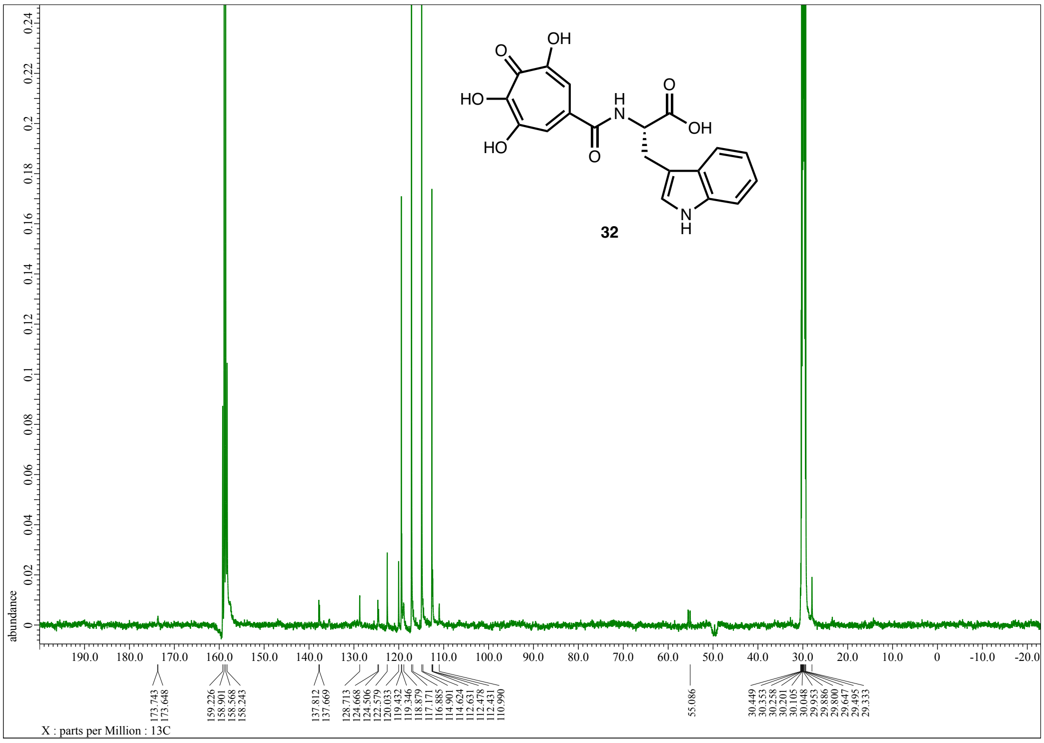

• Fmoc-L-Phe-OTAG (**S-6**): ^{1}H NMR (500 MHz, $CDCl_3$), ^{13}C NMR (125 MHz, $CDCl_3$)

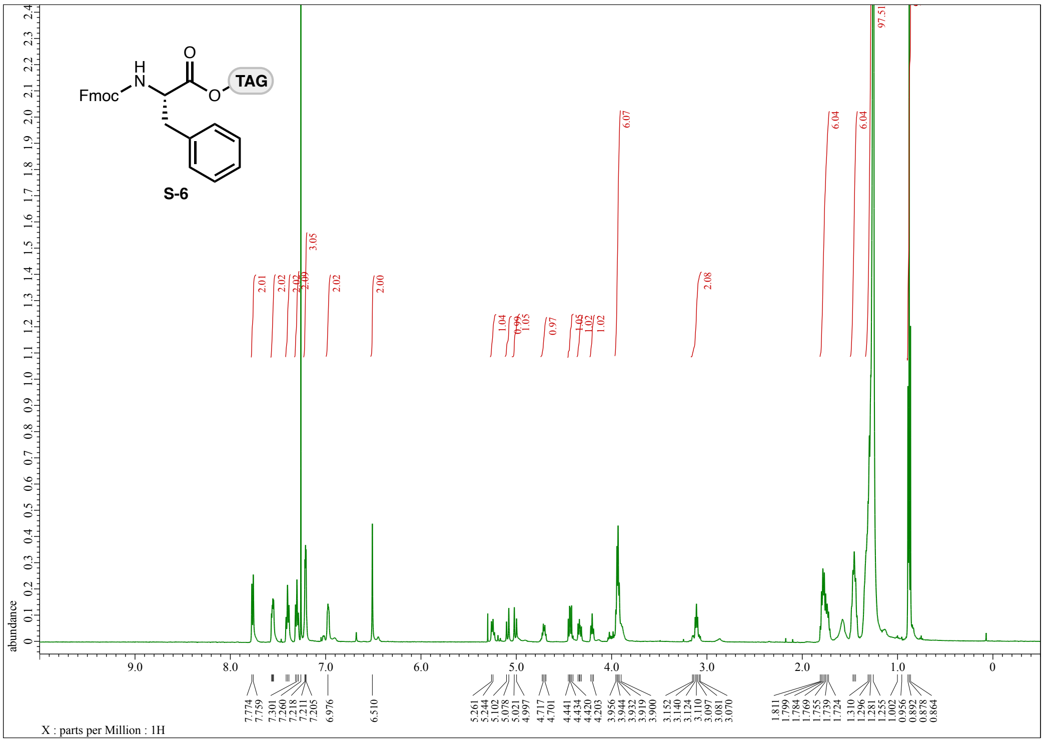

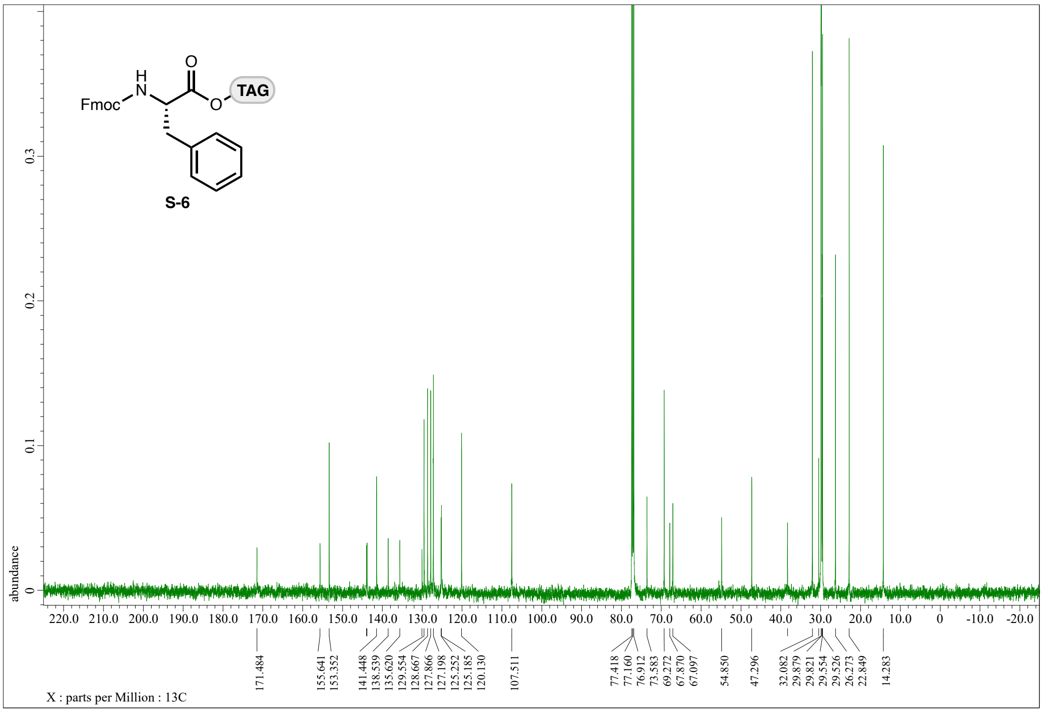

• H-L-Phe-OTAG (**S-7**): ^{1}H NMR (500 MHz, $CDCl_3$), ^{13}C NMR (125 MHz, $CDCl_3$)

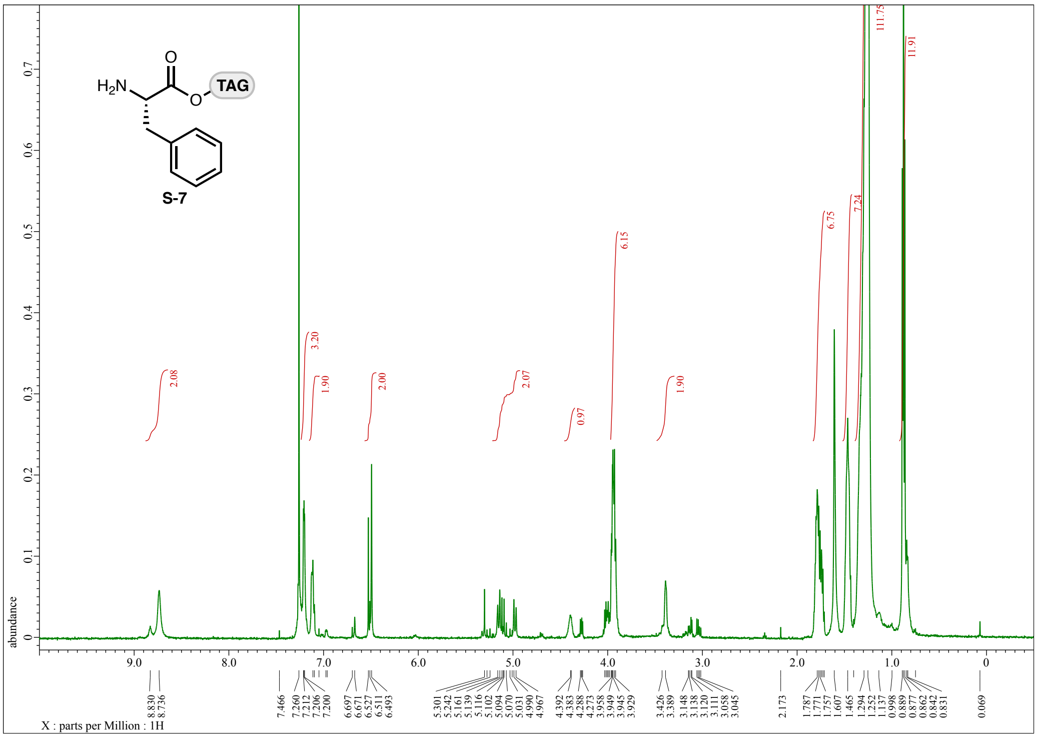

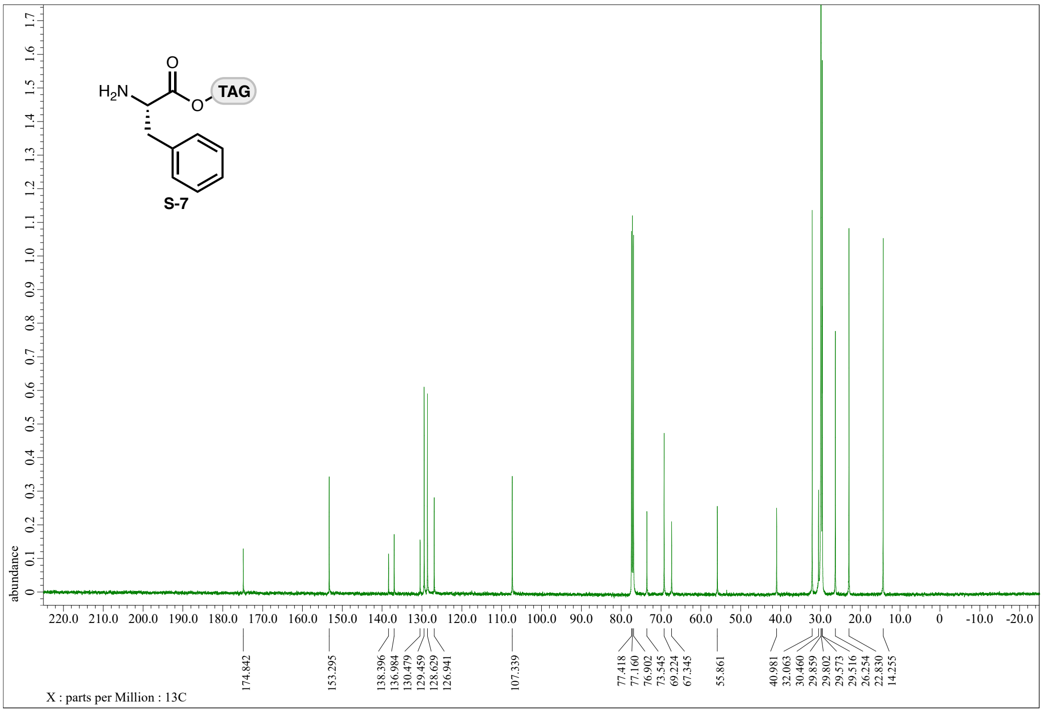

• Fmoc-*N*-Me-L-Leu-L-Phe-OTAG (**S-8**): ^{1}H NMR (500 MHz, $CDCl_3$), ^{13}C NMR (125 MHz, $CDCl_3$)

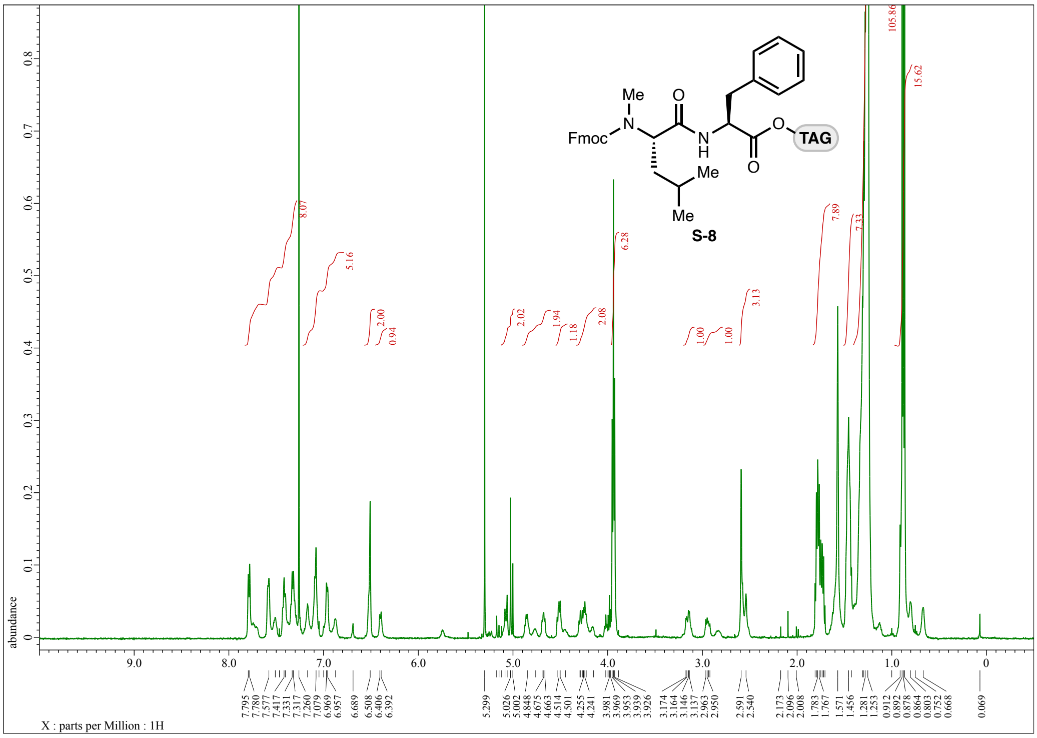

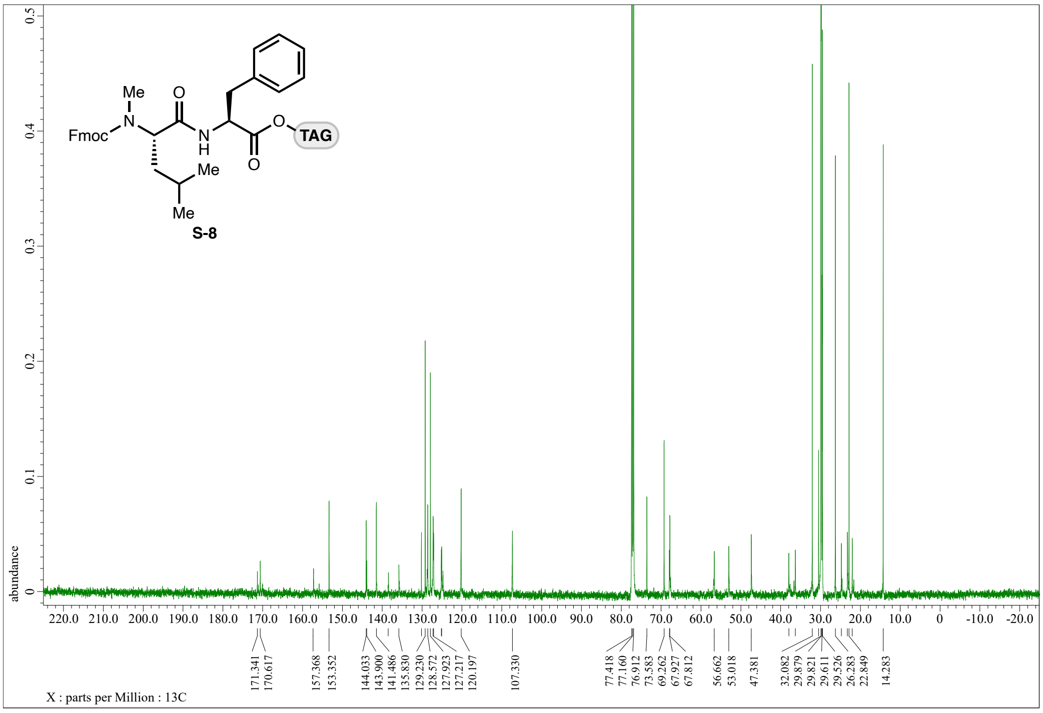

• H-*N*-Me-L-Leu-L-Phe-OTAG (**S-9**): ^{1}H NMR (500 MHz, $CDCl_3$), ^{13}C NMR (125 MHz, $CDCl_3$)

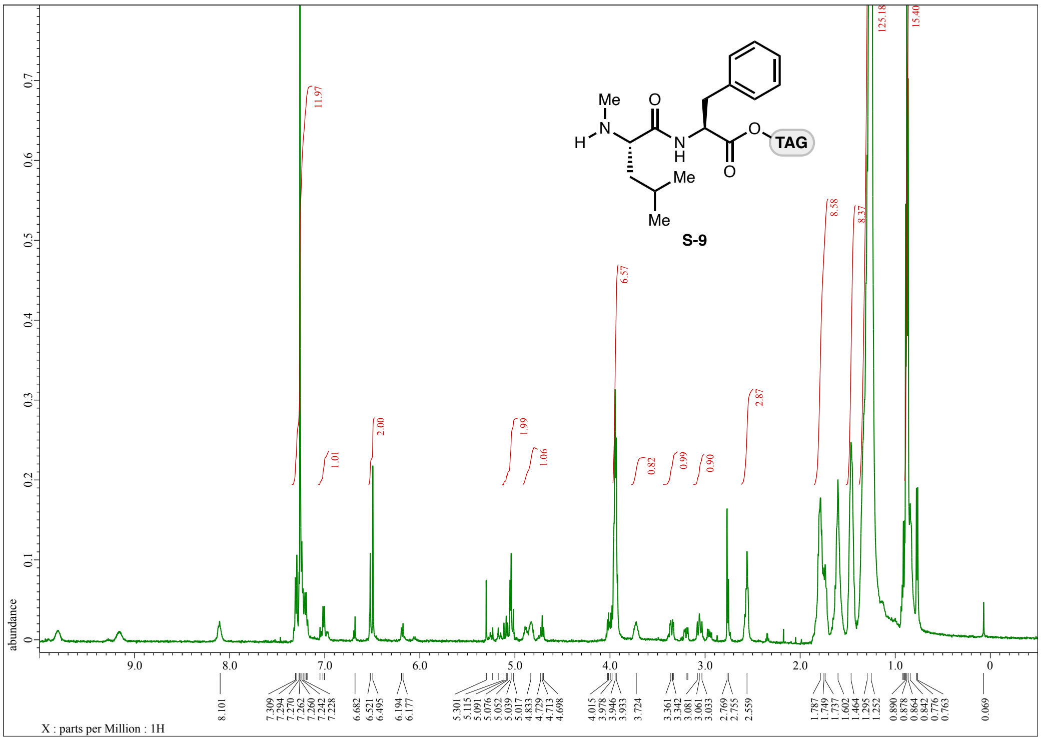

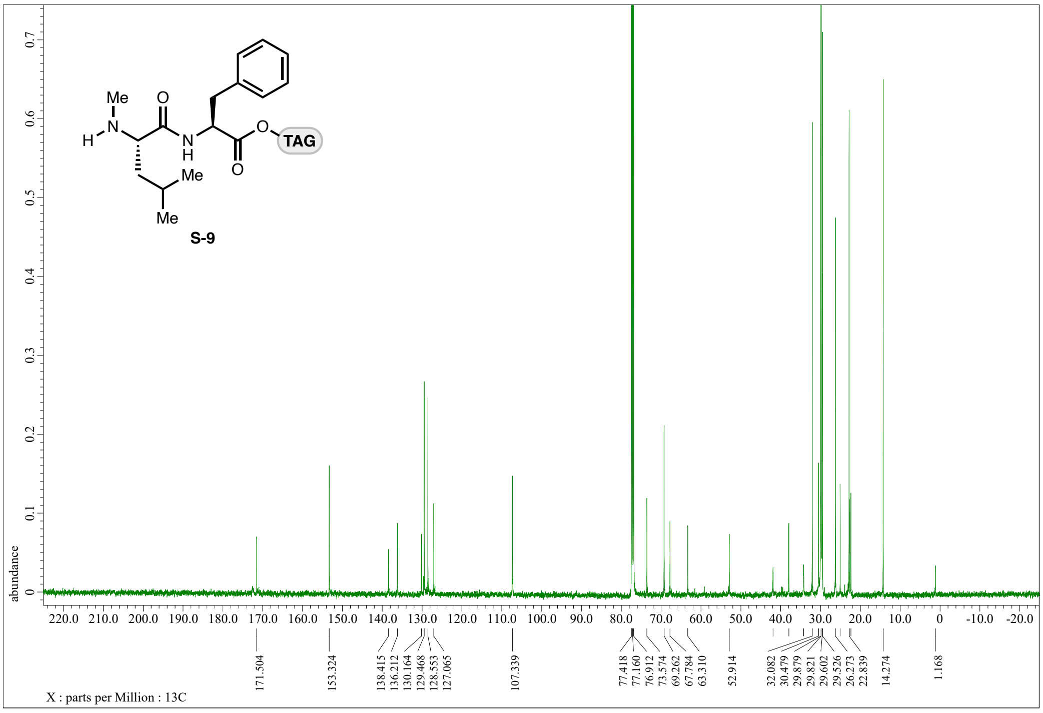

• BnO-tropolone-*N*-Me-L-Leu-L-Phe-OTAG (**S-10**): ^{1}H NMR (500 MHz, $CDCl_3$), ^{13}C NMR (125 MHz, $CDCl_3$)

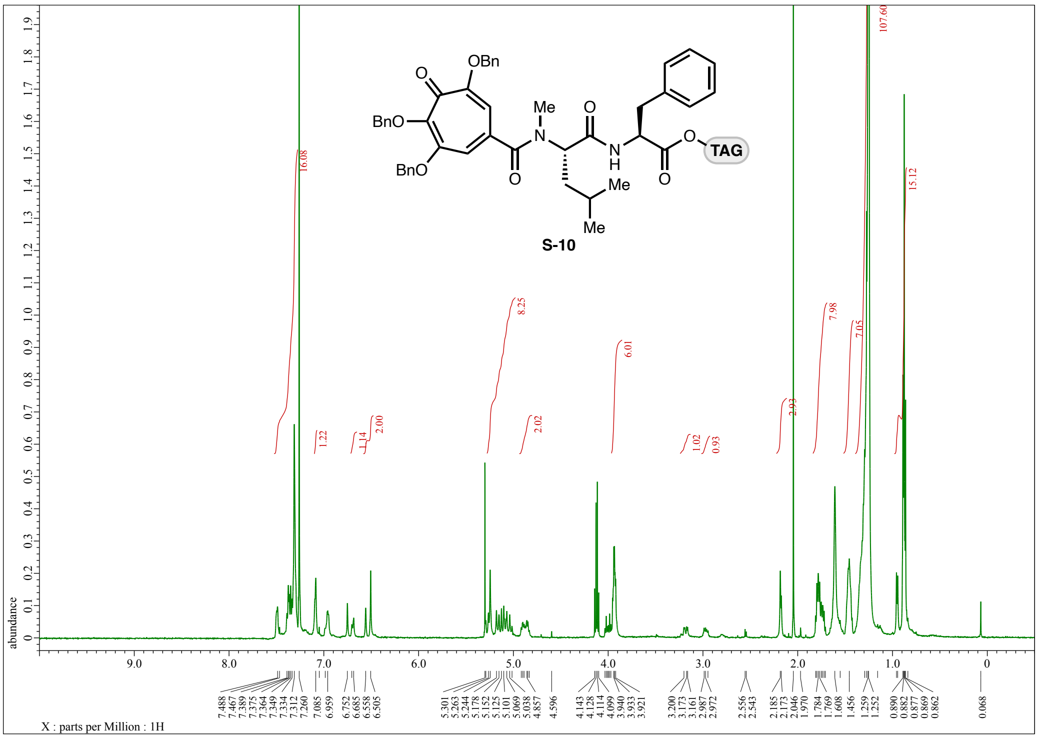

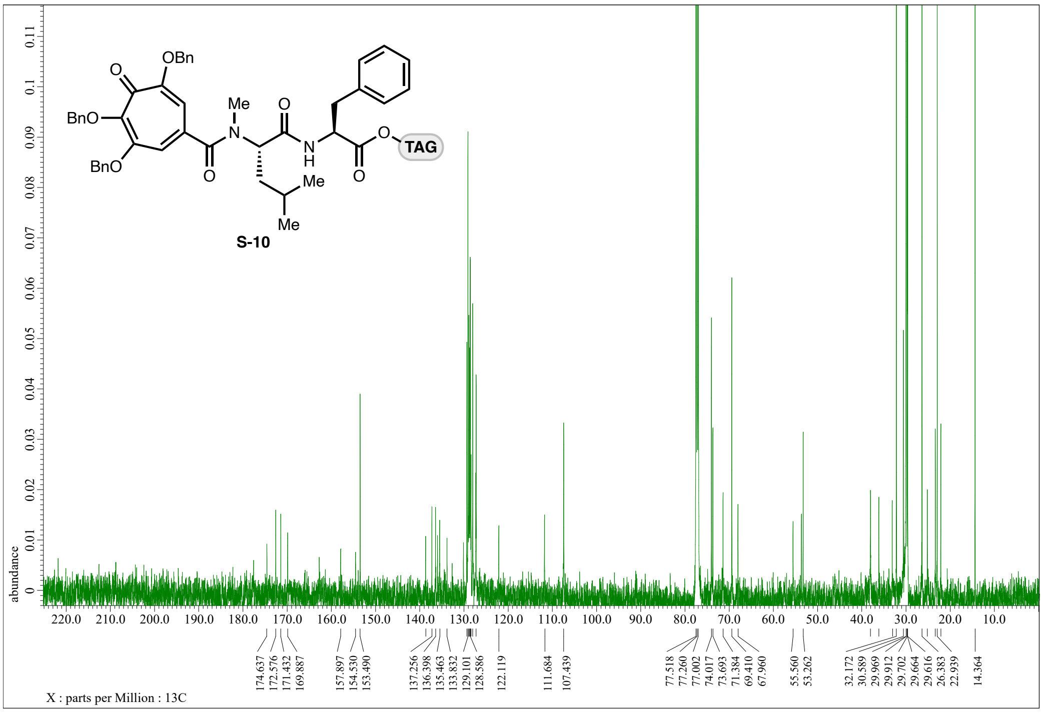

• Tropolone-*N*-Me-L-Leu-L-Phe-OH (**33**): ^{1}H NMR (500 MHz, CD_3OD), ^{13}C NMR (125 MHz, CD_3OD)

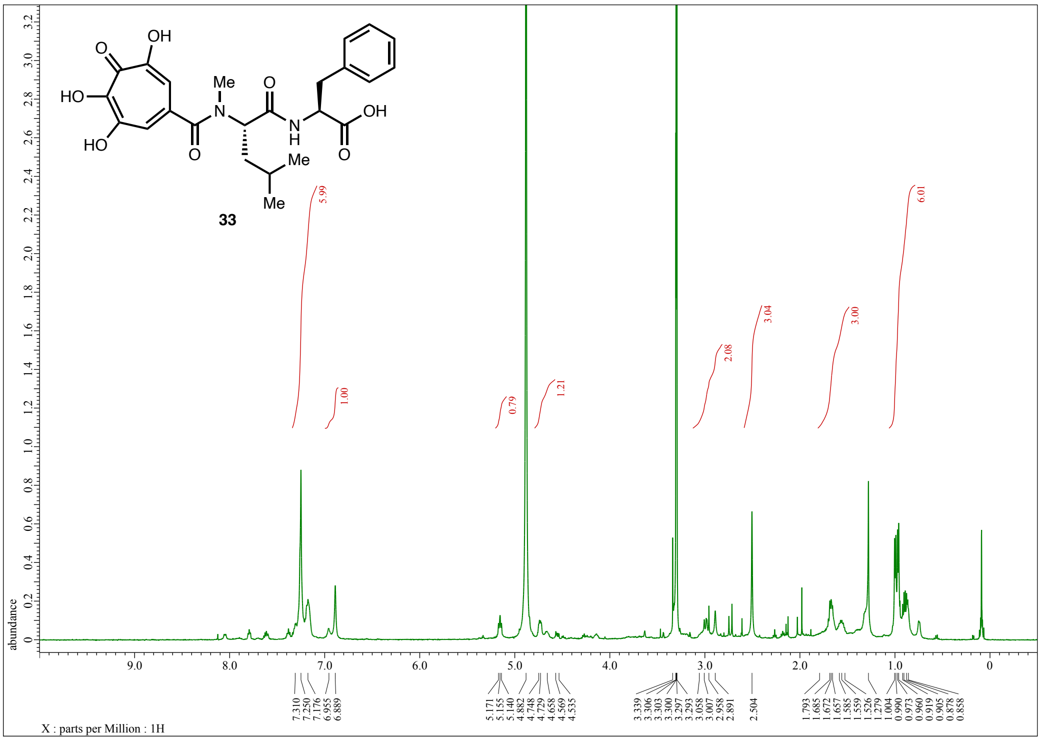

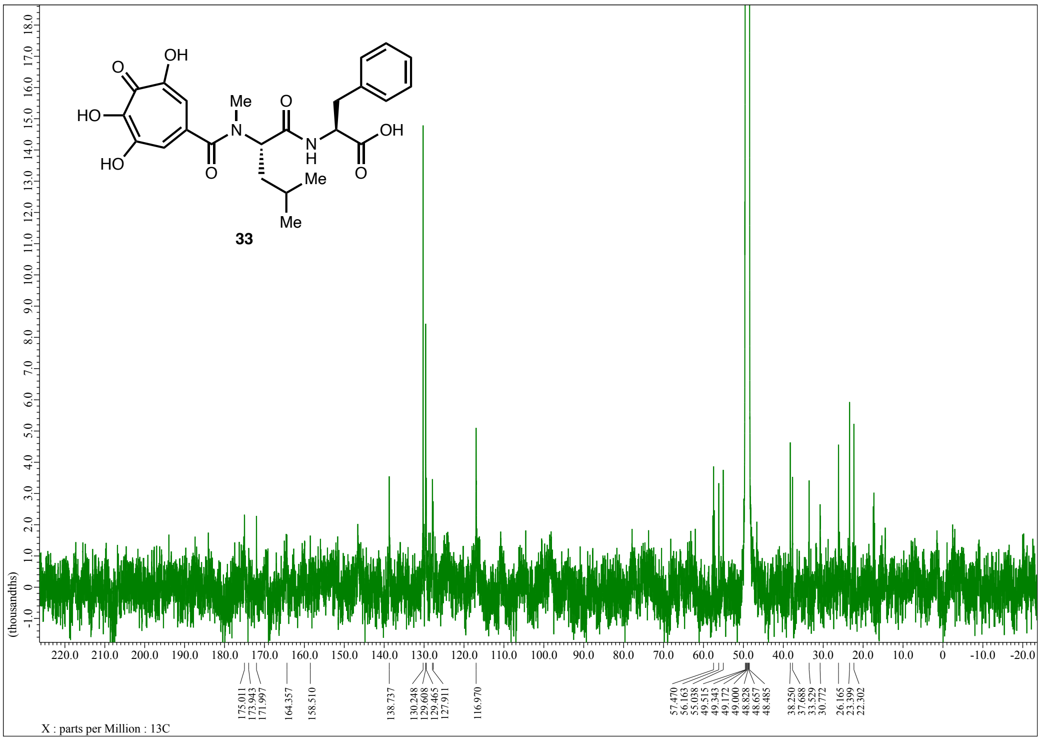

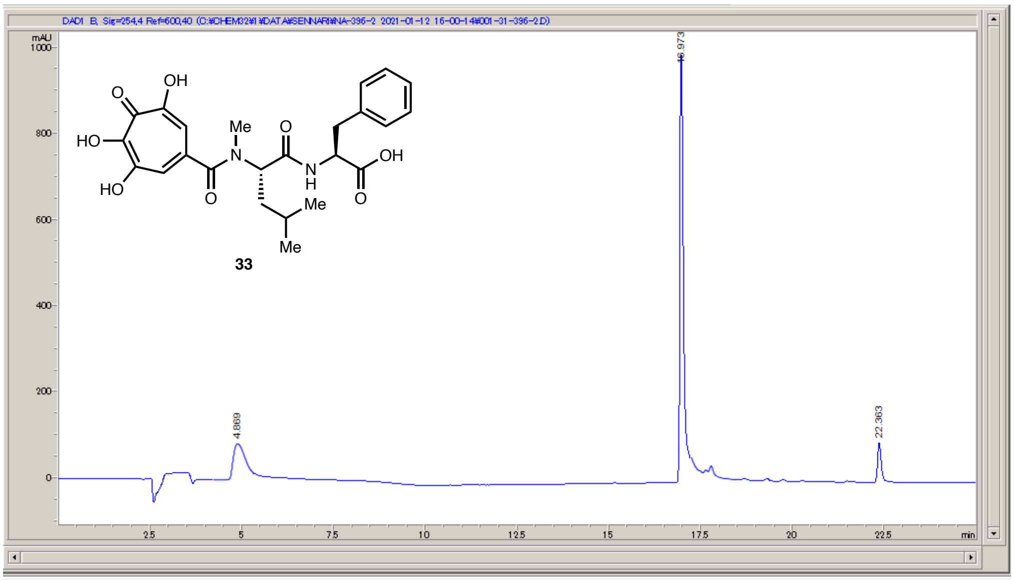

Retention time: 16.973 min

<u>LC/UV method</u>

Measuring equipment: Agilent 1260 Infinity LC

Column: Waters symmetry C18 Column (3.5 μm, 2.1 × 150 mm)

Mobile phase A: H_2O (containing 0.05% H_3PO_4)

Mobile phase B: MeCN (containing 0.05% H_3PO_4)

Linear gradient: A:B = 95:5 to 0:100 (0 – 25 min)

Flow late: 0.2 mL/min

Detect: UV 254 nm

Temperature: 40 ˚C
